# Analogue-based approaches in anti-cancer compound modelling: the relevance of QSAR models

**DOI:** 10.1186/2191-2858-1-3

**Published:** 2011-07-18

**Authors:** Mohammed Hussaini Bohari, Hemant Kumar Srivastava, Garikapati Narahari Sastry

**Affiliations:** 1 Molecular Modelling Group, Indian Institute of Chemical Technology, Taranaka, Hyderabad 500 607, India

**Keywords:** Analogue-based design, Anti-cancer cell lines, Anti-cancer drugs, Quantum chemical descriptors, QSAR, Docking

## Abstract

**Background:**

QSAR is among the most extensively used computational methodology for analogue-based design. The application of various descriptor classes like quantum chemical, molecular mechanics, conceptual density functional theory (DFT)- and docking-based descriptors for predicting anti-cancer activity is well known. Although *in vitro *assay for anti-cancer activity is available against many different cell lines, most of the computational studies are carried out targeting insufficient number of cell lines. Hence, statistically robust and extensive QSAR studies against 29 different cancer cell lines and its comparative account, has been carried out.

**Results:**

The predictive models were built for 266 compounds with experimental data against 29 different cancer cell lines, employing independent and least number of descriptors. Robust statistical analysis shows a high correlation, cross-validation coefficient values, and provides a range of QSAR equations. Comparative performance of each class of descriptors was carried out and the effect of number of descriptors (1-10) on statistical parameters was tested. Charge-based descriptors were found in 20 out of 39 models (approx. 50%), valency-based descriptor in 14 (approx. 36%) and bond order-based descriptor in 11 (approx. 28%) in comparison to other descriptors. The use of conceptual DFT descriptors does not improve the statistical quality of the models in most cases.

**Conclusion:**

Analysis is done with various models where the number of descriptors is increased from 1 to 10; it is interesting to note that in most cases 3 descriptor-based models are adequate. The study reveals that quantum chemical descriptors are the most important class of descriptors in modelling these series of compounds followed by electrostatic, constitutional, geometrical, topological and conceptual DFT descriptors. Cell lines in nasopharyngeal (2) cancer average *R*^2 ^= 0.90 followed by cell lines in melanoma cancer (4) with average *R*^2 ^= 0.81 gave the best statistical values.

## Background

Cancer has been seriously threatening the health and life of humans for a long period and has become the leading disease-related cause of deaths of human population [1]. Radiation therapy and surgery as a means of treatment are only successful when the cancer is found at early-localized stage. However, chemotherapy in contrast is the mainstay in treatment of malignancies because of its ability to cure widespread or metastatic cancers. Natural products are the chemical agents that have been the major source of anti-cancer drugs. According to a review on new chemical entities, approximately 74% of anti-cancer drugs were either natural products or natural product-related synthetic compounds or their mimetics [2]. Computational methodologies have emerged as an indispensible tool for any drug discovery program, playing key role from hit identification to lead optimization. The QSPR/QSAR is among the most practical tool used in analogue/ligand-based drug design and has been extensively reviewed for prediction of various properties like ADME [3], toxicity [4,5], carcinogenicity [6], retention time [7] stability [8] and other physicochemical properties apart from the biological activity [9-12]. This theoretical method follows the axiom that the variance in the activities or physicochemical properties of chemical compounds is determined by the variance in their molecular structures [13-15].

Computational methods aids in not only the design and interpretation of hypothesis-driven experiments in the field of cancer research but also in the rapid generation of new hypotheses. The QSAR has widely been applied for the activity prediction of diverse series of biological and/or chemical compounds including anti-cancer drugs [16-21]. A number of quantum chemical descriptors (such as charge, molecular orbital, dipole moment, etc.) and molecular property descriptors (such as steric, hydrophobic coefficient, etc.) have been successfully applied to establish 2D QSAR models for predicting activities of compounds [22-24]. Density functional theory (DFT)-based descriptors have found immense usefulness in the prediction of reactivity of atoms and molecules, and its application in the development of QSAR has been recently reviewed [25-30]. QSAR has been instrumental in the development of various popular drugs, and it has been discussed in detail earlier [31].

For a cancer type, there are a number of cell lines available, on which *in vitro *evaluation of biological activity can be performed, but the results of this evaluation varies based on the cell line employed for assay. Therefore, it becomes difficult for computational chemist to choose experimental data from a pool of available biological activity for a single scaffold type, so as to proceed for analogue-based design. Although *in vitro *assay for anti-cancer activity is available against many different cell lines, most of the computational studies are carried out targeting any one particular cell line, which may not be a good approach to rely upon. The study considering all the available experimental data to build predictive models, will guide medicinal chemist to more reliably design new and potent compounds. Also, analyzing the obtained descriptors for models against all the cell lines, may suggest the importance of a particular class of descriptor in modelling anti-cancer activity against a cancer type. Such statistically robust and extensive QSAR studies against many different cancer cell lines have not been reported yet. Hence, we performed comprehensive QSAR modelling studies on 266 anti-cancer compounds against 29 different cancer cell lines. Descriptor analysis of all the QSAR models was performed to derive commonality among various cell lines belonging to a cancer type. The experimental data considered in the study was from *in vitro *cell line-based assays, and it is difficult to get reliable target-based information from such studies, unless meticulously validated. Since the aim of the present study was to evaluate the potentials of simple 2D-based descriptors in anti-cancer compound modelling, the biological target-related aspects were not considered. This study provides one of the most comprehensive accounts of the structure-activity relationship of a large number of molecules against 29 different cancer cell lines. Besides being statistically significant, the aim of this study is to assess the role and relevance of computationally demanding conceptual-DFT descriptors compared with the conventional descriptors. The strengths and limitations of QSAR models on treating a complex area such as the development of anti-cancer compounds are important to notice, and the present study shows a systematic way of developing and applying QSAR equations effectively. Table 1 shows the name of scaffolds considered, different cell lines [32-41], number of molecules corresponding to cell lines and the target of action or the molecular mechanism of scaffolds.

**Table 1:** Details of scaffolds considered in the study and the cell lines against which their anticancer activity was reported along with the number of molecules in each cell lines and its molecular target/mechanism of action if studied.

No.	Scaffold name	Cell lines	Cancer type	# of comp.	Comments	Ref.
S1	Naphthalimides	LoVo	Colon	23	DNA intercalators	[31]
		A549	Lung	27		
		Hs468	Glioblastoma	23		
		U373-MG	Glioblastoma	29		
		HCT-15	Colon	25		
		MCF-7	Breast	20		
						
S2	Aryl thiazolyl benzamide	MB-231	Breast	27	Nek2 mitotic pathway	[32]
		MB-468	Breast	25		
		K562	Blood	25		
						
S3	Procaspase activators	U937	Lymphoma	19	Enhance procaspase-3 activity	[33]
						
S4	Tylophorine analogues	KB	Nasopharyngeal	21	NF-kB signalling pathway	[34]
		A549	Lung	21		
		DU-145	Prostate	21		
						
S5	Parthenin analogues	HL-60	Blood	37	TopoisomeraseII inhibition	[35]
		HeLa	Cervical	37		
						
S6	Arylthiazolidine-4-acid amides	A375	Melanoma	33	-	[36]
		B16F1	Melanoma	33		
		DU-145	Prostate	32		
		LNCaP	Prostate	35		
		PC-3	Prostate	31		
		PPC-1	Prostate	33		
		WM-164	Melanoma	32		
		Fibroblast	Fibroblast	27		
		RH7777	Prostate	32		
						
S7	Hydroxyl benzofuranones	LNCaP	Prostate	22	Selective inhibitor of the mammalian target of rapamycin	[37]
						
S8	Arylthiazole-4-acid amides	B16F1	Melenoma	20	Tubulin polymerization inhibition	[38]
		A375	Melanoma	20		
		DU-145	Prostate	16		
		PC-3	Prostate	17		
		LNCaP	Prostate	17		
		PPC-1	Prostate	18		
						
S9	Estradiol 3,17-*O*,*O*-bis-sulfamates	DU-145	Prostate	29	Disruption of the tubulin-microtubule equilibrium	[39]
		MB-231	Breast	22		
						
S10	Aromathecins	SF-539	CNS	29	Inhibitors of human topoisomerase I	[40]
		HOP-62	Lung	28		
		HCT-116	Colon	28		
		UACC-62	Melanoma	27		
		SN12C	Renal	26		
		MCF-7	Breast	29		
		DU-145	Prostate	23		

## Results and discussion

**Scheme 1:**
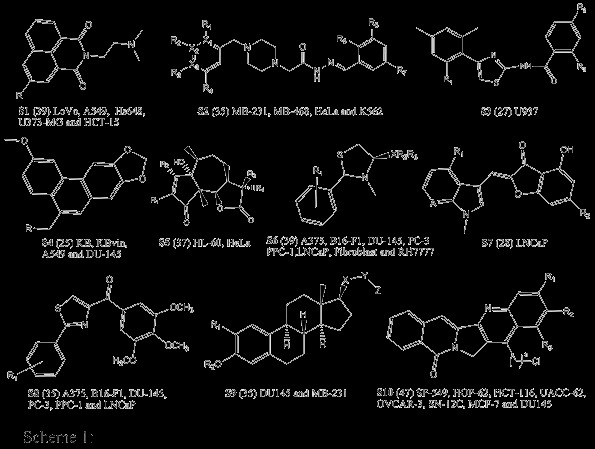
**266 compounds which have IC_50 _values represented into different scaffolds (S1-S10), the number of compounds in each scaffold in parenthesis and different cell lines against which the cytotoxicity values were reported (please see Tables S1-10 in Additional file **1** for structure of all the compounds with their *in vitro *IC_50 _values against various cell lines).**

Two different schemes were opted to develop statistically significant QSAR models. In the first scheme, 10 QSAR models were developed for the 10 scaffolds used in this study (i.e. scaffold-based QSAR models), whereas in the second scheme 29 different QSAR models were developed based on the availability of IC_50 _values against 29 cancer cell lines by combining all the scaffolds (i.e. cell lines-based QSAR models). The parent structure of all the scaffolds with a number of compounds and name of cell lines are represented in Scheme 1.

**Figure 1:**
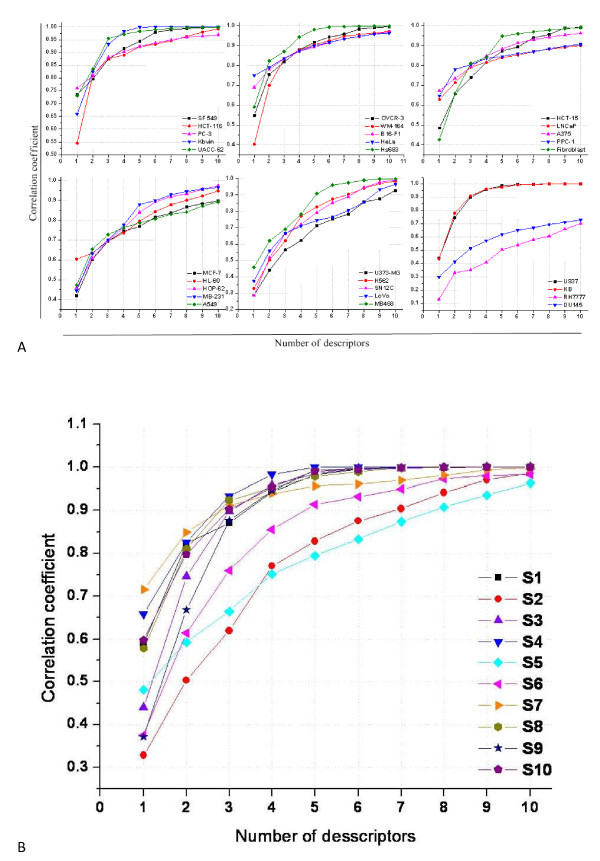
**Effect of number of descriptors on the correlation coefficient of (a) cell line-based QSAR models, (b) scaffold-based QSAR models**.

It is vitally necessary to avoid the oversimplification of the QSAR modelling process and employ statistically robust approaches for the model development. The selection of the best model was based on the values of correlation coefficient obtained from the correlation of approximately 300 descriptors (constitutional, geometrical, topological, electrostatic and quantum chemical, etc.) in different combinations. In one hand, the uniqueness of a compound and its total chemical information cannot be described by very few descriptors while on the other hand large number of descriptors will create confusions and reduce the statistical robustness and predictive ability of the model. The effect of a number of descriptors on the correlation coefficient values for all the models were tested on training set by correlating 1-10 descriptors separately and presented in Figure 1a (for cell lines-based models) and b (for scaffold-based models). We observed that in various models, three descriptors are sufficient for getting a good correlation and using more than three descriptors make only small effect on the statistical quality of the models in most cases. Although more than six descriptor-based models may provide high correlation and cross-validation coefficient values, however, this may be false and thus may not be very useful for the further prediction of IC_50 _values. Before the division of training and test set of compounds three, four and five, descriptor-based models were selected. While comparing the statistical performance of the selected models, three descriptor-based models were found to be optimum as they provide very acceptable correlation in most cases.

All the models were then divided into training and test set by randomly selecting around 20% of the compounds in the test set. Two independent test sets were constructed to rule out chance correlation (statistical data for the second test set is reported in Additional file 1 Table S83). Both the test sets showed the similar statistical performance indicating that the developed models are adequate. Final QSAR models were generated within the training set, and they were used to predict the activity of test set of compounds. The lower average residual obtained in both the training and test set of compounds in all the models indicate that the developed models are valuable and have capability to establish the relationship between the structure and activity for various anti-cancer scaffolds used in this study.

**Figure 2:**
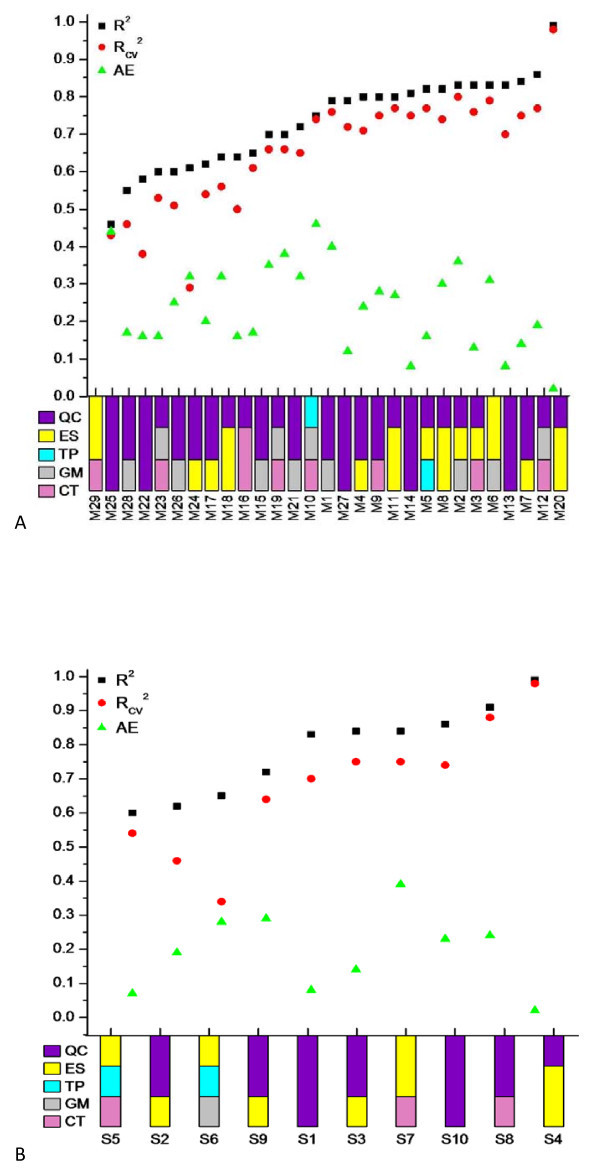
**Regression summary (correlation coefficient *R*^2^, cross-validation coefficient *R*_CV_^2 ^and average residual AE values) for (a) cell line-based QSAR models, (b) scaffold-based QSAR models**.

In order to assess and compare the predictive power and the stability of the QSAR models, several statistical and other parameters are reported and widely applied like *R*^2^, *R*_cv_^2^, *s*^2^, *F*, and AE (for details about these parameters, see footnote to Table 2). Table 2 contains the regression summary for cell lines-based QSAR models along with regression equation, name of the cell lines and types of cancer. Most of the cell lines-based QSAR models where the activity range is broad (M1, M2, M4, M5, M6, M8, M9, M11, M12 and M20) show higher statistical quality (*R*^2 ^~ 0.80, *R*_cv_^2 ^~ 0.75) and seems valuable for the current class of compounds. The statistical quality of few other cell line-based models (M10, M15, M19 and M21) is also reasonable (*R*^2 ^~ 0.75, *R*_cv_^2 ^~ 0.70), and these models can be used for the prediction. However, the statistical qualities of M17, M23 and M26 models, which are lower (*R*^2 ^~ 0.60, *R*_cv_^2 ^~ 0.50), show that extra care is required before utilizing these models for the prediction. However, M29 cannot be used for the prediction because of the insignificant statistical results obtained for this model (*R*^2 ^= 0.46, *R*_cv_^2 ^= 0.43). The reason for poor result in M29 is probably due to involvement of 118 compounds and 5 different scaffolds in this model. The increase in the number of descriptors for M29 is not much improving the quality of the model (with 10 descriptors *R*^2 ^~ 0.7) and indicates that the currently used descriptors are not good enough for developing the structure-activity relationship for this model, and one needs to try or develop additional descriptors. However, the involvement of single scaffolds in this model provides a good statistical quality (DU145/S10 in Table 3). The models (M3, M7, M13, M14, M16, M18, M22, M24, M25, M27 and M28), for which the activity range was narrow were moved to the end of Table 2 and will not be very reliable for predictions. Some of these models (M3, M7, M13, M14 and M27) show higher correlation values (*R*^2 ^~ 0.80, *R*_cv_^2 ^~ 0.75) while other six models show moderate correlation values (*R*^2 ^~ 0.65, *R*_cv_^2 ^~ 0.60) although the residuals are lower in all the 11 models as per expectations. The statistical details and descriptor types for cell line-based QSAR models are depicted in Figure 2a.

**Table 2:** Cell line with type of cancer in parenthesis, scaffolds involved, regression summary and number of compounds in various cell lines based QSAR models.

No	Cell line (Type)	Scf	Regression equation	R^2^	R_cv_^2^	AE	O	s^2^	F	# Comp.		
										TR	TS	PD
M1	A375 (melanoma)	**S6**, **S8**	= 20.6264* MiVH -6.19186* ZXS/ZXR -9719.75*MiNRC -10.9946	0.79	0.76	0.40	1	0.308	43.24	38	12	19
M2	B16-F1 (melanoma)	**S6**, **S8**	= -91.8353*MaPCH-4.3472* ZXS/ZXR-96.017*Ma1ERN +10.0303	0.83	0.80	0.36	1	0.255	55.71	38	12	19
M4	KB (nasopharyngeal)	**S4**	= 769.472* HC-2/Tz +9193.37* Mi1ERN +68.531* MiNACH -7.67576	0.80	0.71	0.24	0	0.065	40.02	17	4	0
M5	WM-164 (melanoma)	**S6**	P = 0.172982*PS-3A_Z_-0.968448* KHI_3_-1205.47* MiNRN+7.6019	0.81	0.77	0.16	1	0.062	33.70	24	6	7
M6	PC-3 (prostate)	**S6**, **S8**	= -3.14901* ZXS/ZXR -95.5552* MaPCH -2.37816*FS-2P_z_+ 9.10613	0.83	0.79	0.31	0	0.136	77.55	29	12	23
M8	UACC-62 (melanoma)	**S10**	= -256.732* MaPCN +13.6563 *MaPC+6.61641 *MaVO-34.0055	0.81	0.74	0.30	1	0.094	51.96	21	5	6
M9	SF-539 (CNS)	**S10**	= 0.000240276*GI_AP_+ 0.113696*TPCCMD +13.1633*MaBOO-20.9902	0.81	0.75	0.28	0	0.120	37.46	21	6	3
M10	LNCaP (prostate)	**S6**, **S7**, **S8**	= -0.0396034* ZXS +0.412216* SIC_0_-24.4713* RNN +0.80884	0.75	0.74	0.46	1	0.370	67.18	58	15	24
M11	PPC-1 (prostate)	**S6**, **S8**	= 0.00211384* PS-1_Z_-17.6992* RPCG_Z _-11.5927* MaNACH +8.51731	0.80	0.77	0.27	1	0.150	48.02	37	12	20
M12	HCT-116 (colon)	**S10**	= -7.48415* ZXS/ZXR+ 0.157414 *TPCCMD-0.0635789* RNCS_Q_+ 7.09755	0.86	0.77	0.19	1	0.157	17.06	24	9	5
M15	MB-231 (breast)	**S2**, **S9**	= -0.031465*YZS+ 5.30324* FBCS_q _+1.16981* MaPBO+1.40089	0.70	0.66	0.35	1	0.191	25.79	37	11	21
M17	A549 (lung)	**S1**, **S4**	= -0.505821* RPCS_Z _-3.59234* MiVO +2.58951* MiBOO + 9.37614	0.64	0.56	0.32	1	0.135	30.22	38	9	12
M19	HOP-62 (lung)	**S10**	= -12.0428* ZXS/ZXR -4.44967* RPCS_Q _-0.819861* NF +12.2371	0.70	0.66	0.38	0	0.278	14.69	23	5	2
M20	KBvin (nasopharyngeal)	**S4**	= -5.01349**HC-1/T -5.01844* PP/SD -0.768924* MaNACC +3.76504	0.99	0.97	0.02	0	0.047	31.28	17	4	0
M21	MCF-7 (breast)	**S1, S10**	= -5.62149*ZXS/ZXR-64.0123* MiNRO-100.36*Mi1ERC+5.76988	0.72	0.65	0.32	1	0.138	31.51	39	9	36
M23	SN12C (renal)	**S10**	= -0.339628*NN-8.49682* XYS/XYR-1.57052* MiVC+ 14.5423	0.60	0.51	0.25	1	0.048	17.50	17	8	5
M26	OVCR-3 (ovarian)	**S1, S10**	= -0.00524177* MSA -0.300618*THCMD +2.31159* MaVO -0.441172	0.63	0.51	0.25	2	0.072	25.39	18	8	18
M29	DU 145 (prostate)	**S4**, **S6**, **S8**, **S9**, **S10**	= 15.0725* RNO +0.00985941* HS-1_Z_-25.5879* H-HC-2/ST +2.19779	0.46	0.43	0.44	1	0.391	28.73	99	18	36
M3	HeLa (cervical)	**S2**, **S5**	= 0.298986*NN+0.00213416*W-1wP+0.849867*MiVC-2.24538	0.83	0.76	0.13	1	0.043	71.93	44	16	11
M7	U937 (lymphoma)	**S3**	= -22.0891* MiBOH +8.67391* MiVN -52.0125* H-HD-2/T -7.6190	0.84	0.75	0.14	0	0.029	32.41	15	4	8
M13	Hs-638 (glioblas toma)	**S1**	= 9.68671* MaVC -1671.96* A1ERC -2.78721*MiVO -30.7528	0.83	0.70	0.08	1	0.009	29.13	17	5	16
M14	HCT-15 (colon)	**S1**	= 2.33664* AVN -2.02577* MiNACN +9.95155* MaVC -46.34	0.86	0.77	0.08	0	0.021	14.25	17	8	14
M16	HL-60 (blood)	**S5**	= -7.57298*RNH-2.67981*RNO+ 3.66509*MaVO-2.2655	0.65	0.61	0.17	0	0.053	18.98	29	8	0
M18	MB468 (breast)	**S2**	= -0.184603* RPCS_Z _+0.0249929* RNCS_Z_-2.74917*MiBOH+4.0713	0.64	0.50	0.16	0	0.044	11.23	19	6	10
M22	LoVo (colon)	**S1**	= 106.594* MaERC +5.20066* MaBOO-47.3247*MaVH +37.9454	0.60	0.53	0.16	0	0.038	13.92	25	6	8
M24	K562 (blood)	**S2**	= 0.034093* H-HC-1_Q _-0.273258* RPCS_Z _-24886* MiERC +1.64689	0.62	0.54	0.20	0	0.061	8.70	19	6	10
M25	U373-MG (glioblas toma)	**S1**	= -27.0375* ANRN -0.297945* H-1E -0.195554*MaBON	0.55	0.46	0.17	0	0.043	8.18	23	6	10
M27	Fibroblast (fibroblast)	**S6**	= 20.3816* MiVN +0.783874* L1E-70.268548*THCMD-59.2997	0.79	0.72	0.12	0	0.046	22.98	20	5	7
M28	RH7777 (prostate)	**S6**	= -43.2403*MI-A-1.24807*MaBOC-69.6817*AERN+4.96796	0.58	0.38	0.16	0	0.057	3.45	23	6	7

*R*^2 ^is the square of the correlation coefficient and represents the statistical significance of the model. *Rcv^2 ^*is the cross-validated *R*^2^, a measure of the quality of the QSAR model. O is the number of outlier for the model. *s^2 ^*is the standard deviation. *F *is the Fischer statistics, the ratio between explained and unexplained variance for a given number of degrees of freedom, thereby indicating a factual correlation or the significance level for QSAR models. AE is the average of absolute difference between experimental and predicted IC_50 _values. TR is number of molecules in training set, TE is test set molecules, PD is number of molecules for which activity was not reported, and the QSAR model predicted it.

**Table 3:** Cell line with type of cancer in parenthesis, scaffolds involved, regression summary and number of compounds in various scaffolds based QSAR models developed for the prediction of IC_50 _values.

No	Cell lines (Type)	Regression equation	R^2^	R_CV_^2^	AE	O	s^2^	F	# Comp.		
									TR	TE	PD
S3	U937 (lymphoma)	= -22.0891* MiBOH +8.67391* MiVN -52.0125* H-HD-2/T -7.61902	0.84	0.75	0.14	0	0.029	32.41	15	4	8
S4	KBvin (nasopharyngeal)	= -5.01349**HC-1/T -5.01844* PP/SD -0.768924* MaNACC +3.76504	0.99	0.98	0.02	0	0.047	31.28	17	4	0
S7	LNCaP (prostate)	= -18.4821*RNC-0.0467594*PS-3A_z_-22.7663*HS-1/T+15.7067	0.84	0.75	0.39	1	0.175	51.03	17	4	5
S8	A375 (melanoma)	= -126.706*Mi1ERS-48.08*RNN -49.070* MaERN +5.9514	0.91	0.88	0.24	0	0.164	48.73	16	4	13
S9	MB231 (breast)	= 32.1529* ABC -64.8239* H-HD-2/T -0.546856* HE -31.4458	0.71	0.58	0.33	1	0.099	30.56	17	4	13
S10	DU-145 (prostate)	= 10.2264*MaBOC+0.17954*TPCCMD-1904.46*MiERO-14.72	0.86	0.74	0.23	0	0.091	36.82	16	5	7
S1	Hs-638 (glioblastoma)	= 9.68671* MaVC -1671.96* A1ERC -2.78721*MiVO -30.7528	0.83	0.70	0.08	1	0.009	29.13	17	5	16
S2	K562 (blood)	= 0.039572* H-HC-1_Q_-0.264148* RPCS_Z_-28043.6*MiERC+1.5088	0.62	0.46	0.19	0	0.061	8.70	19	6	10
S5	HeLa (cervical)	= 0.306112* ACI_2_+ 5.47295*MiPCO +0.533647* RNAB +1.85607	0.57	0.51	0.07	1	0.006	15.88	27	9	0
S6	B16-F1 (melanoma)	= -0.236646*KHI_3_-9.21013*MV/X-128.278*MaPCH+13.064	0.65	0.34	0.28	0	0.096	22.23	25	6	6

Please refer to the footnote of Table 2 for definition of the statistical parameters as well as other abbreviations.

Regression summary for scaffold-based QSAR models along with regression equation, name of the cell lines and types of cancer is given in Table 3. We observed a good statistical quality with higher regression coefficient values in all the scaffold-based QSAR models probably because of the involvement of lesser number of compounds and only one scaffold in the development of these models. The range of activity of compounds in four models (S1, S2, S5 and S6) is narrow, so these models were moved to the end of Table 3 and these models will not be very reliable. The models with narrow activity range compounds show lower regression coefficient values compared with the ones with broad activity range compounds. All the scaffold-based models with broad activity range compounds seem reasonable and can be used for the prediction. The statistical details and descriptor types for scaffold-based QSAR models are depicted in Figure 2b.

**Figure 3:**
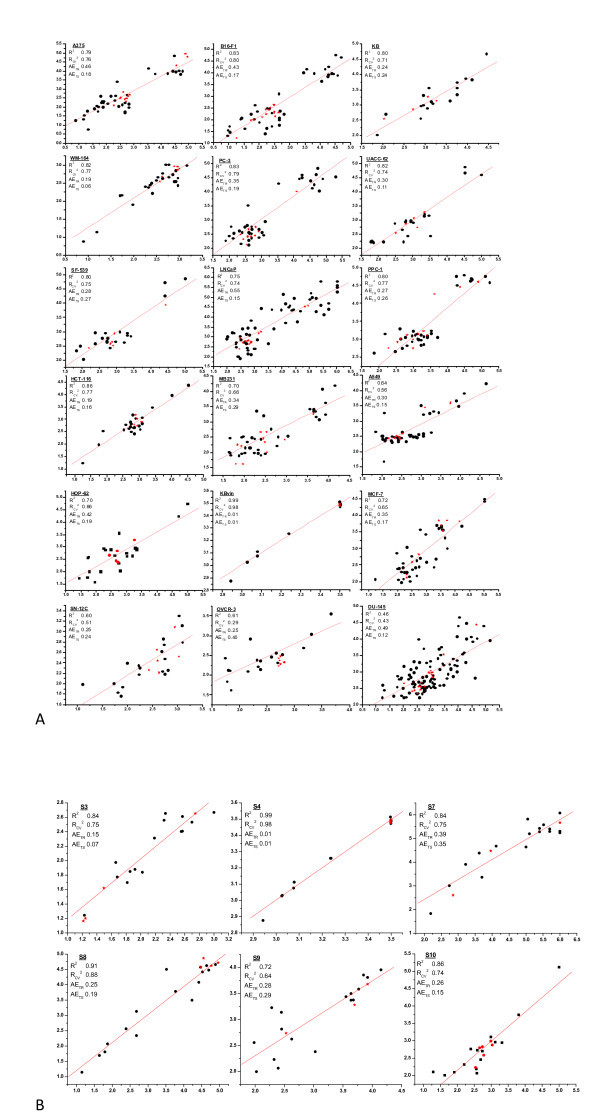
**Plot between experimental and predicted IC_50 _values with correlation coefficient, cross-validation coefficient and average residual for training and test set of molecules separately for** **(a)** **Cell line-based QSAR,** **(b)** **scaffold-based QSAR models.**

The observed and predicted activity with residuals and descriptors values for all the developed models are presented in Additional file 1 (Tables S12 to S46). Outliers are those compounds which are unable to fit in the developed QSAR models. Although most of these QSAR models do not have any outlier, however, in some cases maximum of one outlier is present because of its higher deviation between the observed and predicted activities. The occurrence of outliers is not only due to the possibility that the compounds may act by different mechanisms or interact with the receptor in different binding modes but also due to the intrinsic noise associated with both the original data and methodological aspects opted for the construction of models. Figure 3a,b represents the plot between the experimental and predicted IC_50 _values for cell line- and scaffold-based QSAR, respectively, (the plot for 11 cell line- and 4 scaffold-based models, which has narrow activity range, is presented in Figure S1a,b, respectively, of the Additional file 1). The average residual for test and training set compounds presented in this figure clearly shows the compounds of test set are closer to the line compared with the compounds of training set. Rigorous validation for the applicability of generated QSAR models was done by dividing another independent test set. As per our expectations, the statistical performance of the second test set is similar to that of the first test set. The observed and predicted activity with residuals and descriptors values for all the developed models for the second test set of compounds are presented in Additional file 1 (Tables S48-S82).

**Figure 4:**
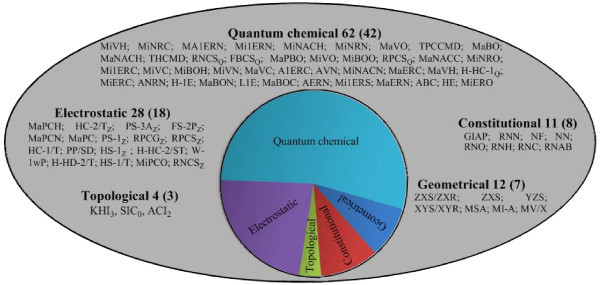
**Classification of various descriptors involved in QSAR model**. Numbers in parenthesis indicates the number of descriptors from one group while numbers outside parenthesis indicates the occurrence of a particular type of descriptor in the models (see Additional file 1 Table S11 for the details of all the descriptors).

In the developed QSAR models, 78 descriptors (42 quantum chemical, 18 electrostatic, 8 constitutional, 7 geometrical and 3 topological) were used in different combinations. Figure 4 depicts the details of all the 78 descriptors, its type and occurrence in the models. The inter-correlation of the descriptors appeared in all the developed models were taken into account, and the descriptors were found to be reasonably orthogonal (see Additional file 1 Table S47 for details). Frequent occurrence of quantum chemical descriptors was found in general in the developed QSAR models. Charge-based descriptors (such as Maximum partial charge for a H atom, Minimum net atomic charge for a H atom, Relative positive charged surface area, Maximum net atomic charge for a C atom etc.) were present in 20 of 39 models (approx. 50%) thereby sharing a major proportion of overall descriptor space. This was followed by valency-based descriptors (such as Minimum valency of O atom, Minimum valency of a C atom, Average valency of a N atom, Maximum valency of a H atom, etc.) present in 14 models (approx. 36%). This was later followed by bond order-based descriptors (such as Minimum (>0.1) bond order of a H atom, Maximum bond order of a N atom, Average bond order of a C atom, Maximum PI-PI bond order, etc.) present in 11 models (~28%). This indicates the role of charge-based, valency-based and bond order-based descriptors in modelling of the present set of compounds. We have tested the conceptual DFT descriptors on all the above models and found that these descriptors are not important for this class of compounds.

Cell lines considered in the current study correspond to 14 different cancer types (Additional file 1 Table S84). Among them, eight cancer types have experimental data with more than one cell line. Thus, comparative statistical significance of various types of cancer has been analysed (see Additional file 1 Table S84 for details). It is interesting to note that nasopharyngeal (*R*^2 ^= 0.90, *R*_cv_^2 ^= 0.84), lymphoma (*R*^2 ^= 0.84, *R*_cv_^2 ^= 0.75), cervical (*R*^2 ^= 0.83, *R*_cv_^2 ^= 0.76), melanoma (*R*^2 ^= 0.81, *R*_cv_^2 ^= 0.77), CNS (*R*^2 ^= 0.81, *R*_cv_^2 ^= 0.75), fibroblast (*R*^2 ^= 0.79, *R*_cv_^2 ^= 0.72) and colon (*R*^2 ^= 0.77, *R*_cv_^2 ^= 0.69) types of cancer show better statistical performance (average *R*^2 ^= 0.82 and average *R*_cv_^2 ^= 0.75) compared with other types of cancer (glioblastoma, prostate, breast, lung, blood, ovarian and renal; average *R*^2 ^= 0.65 and average *R*_cv_^2 ^= 0.57).

## Conclusions

Within the present study, we assessed the predictive power of QSAR approaches to model anti-cancer compounds. A total of 39 QSAR models, 10 for different scaffolds and 29 for different cell lines, were built to assess the predictive power of QSAR models for anti-cancer activity. Although analysis is done with various models where the number of descriptors is increased from 1 to 10, it is interesting to note that in most cases 3 descriptor-based models are adequate. The study reveals that quantum chemical descriptors are the most important class of descriptors followed by electrostatic, constitutional, geometrical, topological and conceptual DFT descriptors. Charge-based descriptors prevailed among the rest, followed by valency-based and bond order-based descriptors. Thus, the current study highlights the importance of analogue-based designing approaches in modelling anti-cancer compounds. Considerably, we did not make any assumptions about the site of interaction or mechanism of action of these compounds yet were able to develop statistically robust models for all experimentally tested compounds where the correlation coefficient (*R*^2^) and cross-validation coefficient (*R*_cv_^2^) values are higher and average residuals (AE) are lower in most cases. Cell lines in nasopharyngeal (2) cancer average *R*^2 ^= 0.90 followed by cell lines in melanoma cancer (4) with average *R*^2 ^= 0.81 gave the best statistical values.

## Methods

**Scheme 2:**
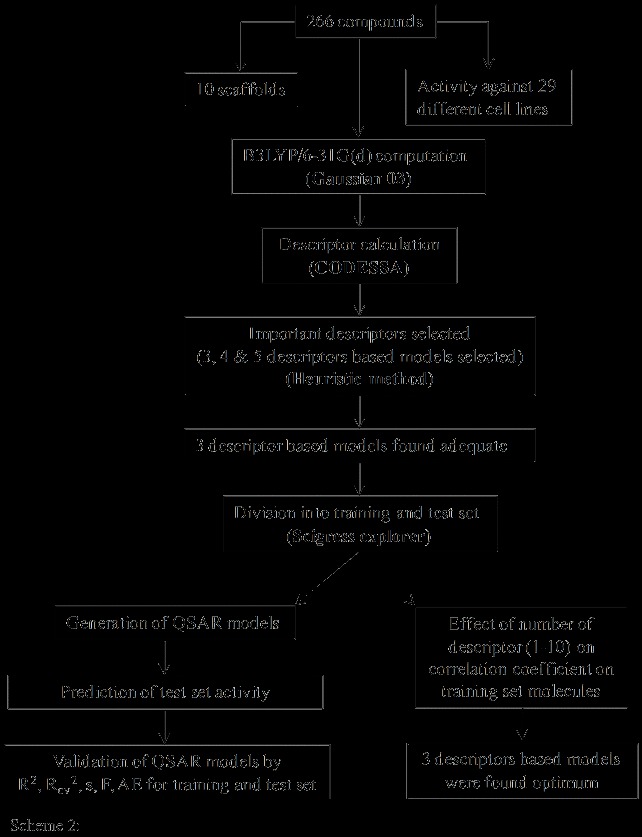
**Flowchart of methodology adopted for building and validating QSAR models**.

Details of the scaffold considered in the study along with the cell lines against which experimental IC_50 _values is reported with number of compounds in each cell line is given in Table 1. Two different schemes (scaffold- and cell line-based) were followed for performing QSAR studies. Scaffold-based QSAR studies were carried out based on the availability of compounds in various scaffolds (**S1**-**S10**) collected from ten different studies. The cell line that provided the best regression summary was used for making scaffold-based QSAR models. See Tables S1-S10 in Additional file 1 for the structure and the corresponding activity values of all the compounds. Scheme 2 provides a schematic illustration of workflow adopted in the manuscript for building and validating various QSAR models. A total of 266 compounds are collected along with their anti-cancer activity against 29 cancer cell lines which belong to 10 different chemical scaffolds (Scheme 1). All the structures were initially optimized using semi-empirical AM1 procedure and later subjected to energy evaluations at B3LYP/6-31G(d) level on AM1 geometries [42]. Important descriptors were obtained using these B3LYP calculations by using the CODESSA [43] program in conjunction with the Gaussian output files. The 300 descriptors obtained using the CODESSA program can be divided into different classes such as constitutional, topological, geometrical, quantum chemical and thermodynamic. For each compound these descriptors were calculated, and non-significant descriptors were identified by heuristic method and eliminated. The inter-correlation of the descriptors in all the models was tested. Then, models where the descriptors are highly inter-correlated were replaced and refined so that the descriptors employed in a given model are virtually orthogonal to each other. In order to find out the minimum number of descriptors defining activity, we systematically developed 3, 4 and 5 descriptor-based models for all sets of compounds, using heuristic method. It was found that three descriptor-based models are fairly satisfactory. Then all the compounds were divided into two independent tests (approx. 20%) and training set (approx. 80%) using Project Leader application associated with Scigress explorer [44]. The statistical quality of the model was assessed by various parameters like *R*^2^, *R*^2^_cv_, AE, *s*, *F*, for both test and training set. The validation of QSAR models was done by examining the prediction of activity on test set i.e. *R*^2^, *R*^2^_cv _and AE. Also, the effect of the number of descriptors on the correlation coefficient was examined on the training set of molecules by running heuristic method at 1-10 descriptors. Two different training and test sets were developed to rule out chance correlation. Scheme 2 illustrates the steps taken for developing the final QSAR models in a schematic fashion.

## Abbreviation

DFT: density functional theory.

## Competing interests

The authors declare that they have no competing interests.

## Supplementary Material

Additional file 1 **The additional data file available with the online version of the article contains following information**: (a) Structure of all the compounds used in this study (Tables S1-S10); (b) Full name of all the descriptors involved in the study (Table S11); (c) The predicted activity and descriptors values for all the models, the first test set (Tables S12-S46); (d) Inter-correlation analysis of the descriptors (Table S47); (e) The predicted activity and descriptors values for all the models, the second test set (Tables S48-S82); (f) Regression summary for cell- line-based and scaffold-based QSAR models pertaining to the second test set (Table S83a and S83b); (g) Comparative statistical significance of various cancer types (Table S84); (h) Figure of plot between the experimental and predicted IC_50 _values for the QSAR models where activity range was narrow, based on cell lines and scaffold (Figure S1a,b). Click here for file
