# Supplementary material for: Analogue-based approaches in anti-cancer compound modelling: the relevance of QSAR models
Source: Org Med Chem Lett. 2011 Jul 18;1:3. doi: 10.1186/2191-2858-1-3 (PMC3279142; doi:10.1186/2191-2858-1-3)
Supplement: Additional file 1 — The additional data file available with the online version of the article contains following information: (a) Structure of all the compounds used in this study (Tables S1-S10); (b) Full name of all the descriptors involved in the study (Table S11); (c) The predicted activity and descriptors values for all the models, the first test set (Tables S12-S46); (d) Inter-correlation analysis of the descriptors (Table S47); (e) The predicted activity and descriptors values for all the models, the second test set (Tables S48-S82); (f) Regression summary for cell- line-based and scaffold-based QSAR models pertaining to the second test set (Table S83a and S83b); (g) Comparative statistical significance of various cancer types (Table S84); (h) Figure of plot between the experimental and predicted IC50 values for the QSAR models where activity range was narrow, based on cell lines and scaffold (Figure S1a,b). [file 2191-2858-1-3-S1.DOC]

**ADDITIONAL INFORMATION FILE**

**Analogue Based Approaches in Anti-Cancer Compound Modeling:**

**The Relevance of QSAR Models**

**Mohammed Bohari, Hemant Kumar Srivastava^*^ and G. Narahari Sastry^*^**

Molecular Modelling Group, Indian Institute of Chemical Technology,

Taranaka, Hyderabad 500 607, INDIA

Phone: +91 40 27193016, Fax: +91 40 27160512

Email: hemantkrsri@gmail.com; gnsastry@gmail.com

TABLE OF CONTENTS:

| **Table S1** | Structure and activity against various cancer cell lines for scaffold 1 | 9 |
| --- | --- | --- |
| **Table S2** | Structure and activity against various cancer cell lines for scaffold 2 | 13 |
| **Table S3** | Structure and activity against various cancer cell lines for scaffold 3 | 17 |
| **Table S4** | Structure and activity against various cancer cell lines for scaffold 4 | 19 |
| **Table S5** | Structure and activity against various cancer cell lines for scaffold 5 | 21 |
| **Table S6** | Structure and activity against various cancer cell lines for scaffold 6 | 23 |
| **Table S7** | Structure and activity against various cancer cell lines for scaffold 7 | 25 |
| **Table S8** | Structure and activity against various cancer cell lines for scaffold 8 | 27 |
| **Table S9** | Structure and activity against various cancer cell lines for scaffold 9 | 29 |
| **Table S10** | Structure and activity against various cancer cell lines for scaffold 10 | 30 |
| **Table S11** | Details of the descriptors involved in the different combination in QSAR studies | 34 |
| **Table S12** | Descriptors, experimental and predicted pIC_50_ values and their residuals for cell line based QSAR model against A375 | 37 |
| **Table S13** | Descriptor, experimental and predicted pIC_50_ values and their residuals for test set 1compounds in cell line based QSAR model against A549 | 39 |
| **Table S14** | Descriptor, experimental and predicted pIC_50_ values and their residuals for test set 1compounds in cell line based QSAR model against B16-F1 | 41 |
| **Table S15** | Descriptor, experimental and predicted pIC_50_ values and their residuals for test set 1compounds in cell line based QSAR model against DU145 | 43 |
| **Table S16** | Descriptor, experimental and predicted pIC_50_ values and their residuals for test set 1compounds in cell line based QSAR model against Fibroblast | 47 |
| **Table S17** | Descriptor, experimental and predicted pIC_50_ values and their residuals for test set 1compounds in cell line based QSAR model against HCT-15 | 48 |
| **Table S18** | Descriptor, experimental and predicted pIC_50_ values and their residuals for test set 1compounds in cell line based QSAR model against HOP-62 | 49 |
| **Table S19** | Descriptor, experimental and predicted pIC_50_ values and their residuals for test set 1compounds in cell line based QSAR model against HCT-116 | 50 |
| **Table S20** | Descriptor, experimental and predicted pIC_50_ values and their residuals for test set 1compounds in cell line based QSAR model against HeLA | 51 |
| **Table S21** | Descriptor, experimental and predicted pIC_50_ values and their residuals for test set 1compounds in cell line based QSAR model against HL-60 | 53 |
| **Table S22** | Descriptor, experimental and predicted pIC_50_ values and their residuals for test set 1compounds in cell line based QSAR model against HS-638 | 54 |
| **Table S23** | Descriptor, experimental and predicted pIC_50_ values and their residuals for test set 1compounds in cell line based QSAR model against KB | 55 |
| **Table S24** | Descriptor, experimental and predicted pIC_50_ values and their residuals for test set 1compounds in cell line based QSAR model against KBvin | 56 |
| **Table S25** | Descriptor, experimental and predicted pIC_50_ values and their residuals for test set 1compounds in cell line based QSAR model against LNCaP | 57 |
| **Table S26** | Descriptor, experimental and predicted pIC_50_ values and their residuals for test set 1compounds in cell line based QSAR model against LoVo | 60 |
| **Table S27** | Descriptor, experimental and predicted pIC_50_ values and their residuals for test set 1compounds in cell line based QSAR model against MB231 | 61 |
| **Table S28** | Descriptor, experimental and predicted pIC_50_ values and their residuals for test set 1compounds in cell line based QSAR model against MB468 | 63 |
| **Table S29** | Descriptor, experimental and predicted pIC_50_ values and their residuals for test set 1compounds in cell line based QSAR model against MCF-7 | 64 |
| **Table S30** | Descriptor, experimental and predicted pIC_50_ values and their residuals for test set 1compounds in cell line based QSAR model against OVCR-3 | 66 |
| **Table S31** | Descriptor, experimental and predicted pIC_50_ values and their residuals for test set 1compounds in cell line based QSAR model against PC-3 | 68 |
| **Table S32** | Descriptor, experimental and predicted pIC_50_ values and their residuals for test set 1compounds in cell line based QSAR model against PPC-1 | 70 |
| **Table S33** | Descriptor, experimental and predicted pIC_50_ values and their residuals for test set 1compounds in cell line based QSAR model against RH7777 | 72 |
| **Table S34** | Descriptor, experimental and predicted pIC_50_ values and their residuals for test set 1compounds in cell line based QSAR model against SF-539 | 73 |
| **Table S35** | Descriptor, experimental and predicted pIC_50_ values and their residuals for test set 1compounds in cell line based QSAR model against SN12C | 75 |
| **Table S36** | Descriptor, experimental and predicted pIC_50_ values and their residuals for test set 1compounds in cell line based QSAR model against U937 | 76 |
| **Table S37** | Descriptor, experimental and predicted pIC_50_ values and their residuals for test set 1compounds in cell line based QSAR model against UACC-62 | 77 |
| **Table S38** | Descriptor, experimental and predicted pIC_50_ values and their residuals for test set 1compounds in cell line based QSAR model against WM-164 | 78 |
| **Table S39** | Descriptor, experimental and predicted pIC_50_ values and their residuals for test set 1compounds in cell line based QSAR model against U373-MG | 79 |
| **Table S40** | Descriptor, experimental and predicted pIC_50_ values and their residuals for test set 1compounds in cell line based QSAR model against K562 | 80 |
| **Table S41** | Descriptor, experimental and predicted pIC_50_ values and their residuals for test set 1compounds in scaffold based QSAR model S5 | 81 |
| **Table S42** | Descriptor, experimental and predicted pIC50 values and their residuals for test set 1compounds in scaffold based QSAR model S6 | 82 |
| **Table S43** | Descriptor, experimental and predicted pIC50 values and their residuals for test set 1compounds in scaffold based QSAR model S7 | 83 |
| **Table S44** | Descriptor, experimental and predicted pIC50 values and their residuals for test set 1compounds in scaffold based QSAR model S8 | 84 |
| **Table S45** | Descriptor, experimental and predicted pIC50 values and their residuals for test set 1compounds in scaffold based QSAR model S9 | 85 |
| **Table S46** | Descriptor, experimental and predicted pIC50 values and their residuals for test set 1compounds in scaffold based QSAR model S10 | 86 |
| **Table S47** | Analysis of Inter-correlation of the descriptors | 87 |
|  |  |  |
| **Table S48** | Descriptors, experimental and predicted pIC_50_ values and their residuals for test set 2 compounds in cell line based QSAR model against A375 | 91 |
| **Table S49** | Descriptor, experimental and predicted pIC_50_ values and their residuals for test set 2 compounds in cell line based QSAR model against A549 | 93 |
| **Table S50** | Descriptor, experimental and predicted pIC_50_ values and their residuals for test set 2 compounds in cell line based QSAR model against B16-F1 | 95 |
| **Table S51** | Descriptor, experimental and predicted pIC_50_ values and their residuals for test set 2 compounds in cell line based QSAR model against DU145 | 97 |
| **Table S52** | Descriptor, experimental and predicted pIC_50_ values and their residuals for test set 2 compounds in cell line based QSAR model against Fibroblast | 101 |
| **Table S53** | Descriptor, experimental and predicted pIC_50_ values and their residuals for test set 2 compounds in cell line based QSAR model against HCT-15 | 102 |
| **Table S54** | Descriptor, experimental and predicted pIC_50_ values and their residuals for test set 2 compounds in cell line based QSAR model against HOP-62 | 103 |
| **Table S55** | Descriptor, experimental and predicted pIC_50_ values and their residuals for test set 2 compounds in cell line based QSAR model against HCT-116 | 104 |
| **Table S56** | Descriptor, experimental and predicted pIC_50_ values and their residuals for test set 2 compounds in cell line based QSAR model against HeLA | 105 |
| **Table S57** | Descriptor, experimental and predicted pIC_50_ values and their residuals for test set 2 compounds in cell line based QSAR model against HL-60 | 107 |
| **Table S58** | Descriptor, experimental and predicted pIC_50_ values and their residuals for test set 2 compounds in cell line based QSAR model against HS-638 | 108 |
| **Table S59** | Descriptor, experimental and predicted pIC_50_ values and their residuals for test set 2 compounds in cell line based QSAR model against KB | 109 |
| **Table S60** | Descriptor, experimental and predicted pIC_50_ values and their residuals for test set 2 compounds in cell line based QSAR model against KBvin | 110 |
| **Table S61** | Descriptor, experimental and predicted pIC_50_ values and their residuals for test set 2 compounds in cell line based QSAR model against LNCaP | 111 |
| **Table S62** | Descriptor, experimental and predicted pIC_50_ values and their residuals for test set 2 compounds in cell line based QSAR model against LoVo | 114 |
| **Table S63** | Descriptor, experimental and predicted pIC_50_ values and their residuals for test set 2 compounds in cell line based QSAR model against MB231 | 115 |
| **Table S64** | Descriptor, experimental and predicted pIC_50_ values and their residuals for test set 2 compounds in cell line based QSAR model against MB468 | 117 |
| **Table S65** | Descriptor, experimental and predicted pIC_50_ values and their residuals for test set 2 compounds in cell line based QSAR model against MCF-7 | 118 |
| **Table S66** | Descriptor, experimental and predicted pIC_50_ values and their residuals for test set 2 compounds in cell line based QSAR model against OVCR-3 | 120 |
| **Table S67** | Descriptor, experimental and predicted pIC_50_ values and their residuals for test set 2 compounds in cell line based QSAR model against PC-3 | 122 |
| **Table S68** | Descriptor, experimental and predicted pIC_50_ values and their residuals for test set 2 compounds in cell line based QSAR model against PPC-1 | 124 |
| **Table S69** | Descriptor, experimental and predicted pIC_50_ values and their residuals for test set 2 compounds in cell line based QSAR model against RH7777 | 126 |
| **Table S70** | Descriptor, experimental and predicted pIC_50_ values and their residuals for test set 2 compounds in cell line based QSAR model against SF-539 | 127 |
| **Table S71** | Descriptor, experimental and predicted pIC_50_ values and their residuals for test set 2 compounds in cell line based QSAR model against SN12C | 129 |
| **Table S72** | Descriptor, experimental and predicted pIC_50_ values and their residuals for test set 2 compounds in cell line based QSAR model against U937 | 130 |
| **Table S73** | Descriptor, experimental and predicted pIC_50_ values and their residuals for test set 2 compounds in cell line based QSAR model against UACC-62 | 131 |
| **Table S74** | Descriptor, experimental and predicted pIC_50_ values and their residuals for test set 2 compounds in cell line based QSAR model against WM-164 | 132 |
| **Table S75** | Descriptor, experimental and predicted pIC_50_ values and their residuals for test set 2 compounds in cell line based QSAR model against U373-MG | 133 |
| **Table S76** | Descriptor, experimental and predicted pIC_50_ values and their residuals for test set 2 compounds in cell line based QSAR model against K562 | 134 |
| **Table S77** | Descriptor, experimental and predicted pIC_50_ values and their residuals for test set 2 compounds in scaffold based QSAR model S5 | 135 |
| **Table S78** | Descriptor, experimental and predicted pIC50 values and their residuals for test set 2 compounds in scaffold based QSAR model S6 | 136 |
| **Table S79** | Descriptor, experimental and predicted pIC50 values and their residuals for test set 2 compounds in scaffold based QSAR model S7 | 137 |
| **Table S80** | Descriptor, experimental and predicted pIC50 values and their residuals for test set 2 compounds in scaffold based QSAR model S8 | 138 |
| **Table S81** | Descriptor, experimental and predicted pIC50 values and their residuals for test set 2 compounds in scaffold based QSAR model S9 | 139 |
| **Table S82** | Descriptor, experimental and predicted pIC50 values and their residuals for test set 2 compounds in scaffold based QSAR model S10 | 141 |
| **Table S83a** | Cell line with type of cancer in parenthesis, scaffolds involved, regression summary (regression equation, correlation coefficient R^2^, cross validation coefficient R_cv_^2^, average residual AE and number of outliers O) and number of compounds (training set TR, test set TS and predicted set PD) in various cell lines based QSAR models for the second test set. | 142 |
| **Table S83b** | Cell line with type of cancer in parenthesis, scaffolds involved, regression summary (regression equation, correlation coefficient R^2^, cross validation coefficient R_cv_^2^, average residual AE and number of outliers O) and number of compounds (training set TR, test set TS and predicted set PD) in various scaffolds based QSAR models for the second test set. | 144 |
| **Table S84** | Comparative statistical significance (correlation coefficient R^2^, cross validation coefficient R_cv_^2^) of various type of cancer involved in the cell line based QSAR study | 145 |
| **Figure S1a** | Plot between experimental and predicted IC_50_ values for 11-cell line based QSAR models with name of cell lines, correlation coefficient, cross validation coefficient, average residual of training set and average residual of test set of molecules | 148 |
| **Figure S1b** | Plot between experimental and predicted IC_50_ values for 4 scaffold based QSAR models with name of cell lines, correlation coefficient, cross validation coefficient, average residual of training set and average residual of test set of molecules | 149 |

**Table S1: Structure and activity against various cancer cell lines for scaffold 1.**

| **No.** | **Structure** | **IC_50_** | | | | | |
| --- | --- | --- | --- | --- | --- | --- | --- |
|  |  | **Hs468** | **U373-MG** | **HCT-15** | **LoVo** | **A549** | **MCF-7** |
|  |  |  |  |  |  |  |  |
| 1 | NO_2_ | 3.7 | 1.3 | 2.6 | 0.4 | 2.4 | - |
| 2 | NH_2_ | 3.9 | 3.5 | 5.2 | 2.7 | 3.8 | 5.8 |
| 3 | H | 6.9 | 7.5 | 9.2 | 4.7 | 5.6 | 10 |
| 4 |  | >10 | 9 | 7.8 | >10 | >10 | >10 |
| 5 |  | 4.8 | 4.4 | 4.3 | 6.6 | 3.9 | 7.4 |
| 6 |  | >10 | >10 | >10 | >10 | >10 | >10 |
| 7 |  | >10 | >10 | >10 | 10 | >10 | >10 |
| 8 |  | 7 | 5.5 | 5.5 | 4.8 | 4.7 | 4.8 |
| 9 |  | >10 | >10 | >10 | >10 | >10 | >10 |
| 10 |  | 4.2 | 2.7 | 2.8 | 1.3 | 3.2 | 2.2 |
| 11 |  | >10.0 | 8.9 | >10.0 | 6.4 | 10 | >10 |
| 12 |  | 8.7 | 8.1 | >10 | 4.5 | 9.5 | 9.6 |
| 13 |  | 5.6 | 6 | 8.4 | 3.7 | 8.5 | >10 |
| 14 |  | 3.3 | 4.2 | 7.2 | 4.1 | 5.9 | >10 |
| 15 |  | 3 | 3.3 | 4.5 | 2.9 | 4.1 | 4.9 |
| 16 |  | 0.8 | 0.9 | 0.9 | 0.9 | 0.9 | 1.8 |
| 17 |  | 3 | 3.4 | 3.4 | 2.1 | 2 | 3.6 |
| 18 |  | 3.6 | 4.1 | 4.4 | 7.7 | 4.9 | 6.3 |
| 19 |  | 2.8 | 3.6 | 3.1 | 2 | 1.9 | 2.4 |
| 20 |  | 5 | 0.9 | 9.7 | 4.2 | 3.5 | 3.1 |
| 21 |  | 4.6 | 1 | 5.6 | 1.8 | 3 | 3.9 |
| 22 |  | 2.1 | 2.1 | 7.4 | 1.5 | 1.6 | 3.1 |
| 23 |  | 5.8 | 1.4 | 7.7 | 4.4 | 5.5 | 6.6 |
| 24 |  | 2.6 | 3.1 | 5.8 | 3.2 | 3.1 | 6.5 |
| 25 |  | 5 | 3.6 | 4.6 | 2.8 | 3.6 | 4.8 |
| 26 |  | >10 | >10 | 9 | 8.3 | >10 | >10 |
| 27 |  | >10 | >10 | >10 | >10 | >10 | >10 |
| 28 |  | >10 | 7.8 | >10 | 8.4 | 9.1 | >10 |
| 29 |  | >10 | 6.4 | >10 | 5 | 6.7 | >10 |
| 30 |  | >10 | 4.2 | 9.9 | 4.1 | 7.7 | 7.4 |
| 31 |  | >10 | 5 | >10 | >10 | >10 | >10 |
| 32 |  | 9.8 | >10 | 8.2 | 4.8 | 8.5 | >10 |
| 33 |  | >10 | >10 | >10 | >10 | >10 | >10 |
| 34 |  | >10 | >10 | >10 | 8.1 | >10 | >10 |
| 35 |  | 5.5 | 7.1 | 6.7 | 4 | 9.7 | 8.9 |
| 36 |  | 6.4 | 3.6 | 5.9 | 4 | 4.3 | 4.8 |
| 37 |  | >10 | >10 | >10 | >10 | >10 | >10 |
| 38 |  | >10 | >10 | >10 | 8.7 | >10 | >10 |
| 39 |  | >10 | 9.4 | >10 | >10 | >10 | >10 |

**Table S2: Structure and activity against various cancer cell lines for scaffold 2.**

| **No.** | **Structure** | **IC_50_** | | | |
| --- | --- | --- | --- | --- | --- |
|  |  | **MB-231** | **MB-468** | **HeLa** | **K562** |
| 40 |  | 8.6 | 10.5 | 8.8 | 11.7 |
| 41 |  | 9.7 | 11.2 | 13.6 | 9.5 |
| 42 |  | >40 | >40 | >40 | >40 |
| 43 |  | >40 | >40 | >40 | >40 |
| 44 |  | 23.4 | >40 | >40 | >40 |
| 45 |  | 1.7 | 2.1 | 2.4 | 2.5 |
| 46 |  | >40 | >40 | >40 | >40 |
| 47 |  | 5.9 | 4.1 | 10.6 | 7.5 |
| 48 |  | 7.5 | 6.2 | 14.5 | 13.3 |
| 49 |  | 4.1 | 2.3 | 5.2 | 4.9 |
| 50 |  | 4.1 | 5.5 | 5.8 | 8.2 |
| 51 |  | 3.2 | 3 | 3.6 | 3.9 |
| 52 |  | 1.1 | 3.4 | 1.6 | 1.2 |
| 53 |  | >25 | >25 | >25 | >25 |
| 54 |  | 3.2 | 4.7 | 5.7 | 5.9 |
| 55 |  | >25 | >25 | >25 | >25 |
| 56 |  | 6.8 | 10.7 | 11.2 | 10.1 |
| 57 |  | 2.9 | 6.2 | 7.4 | 10.1 |
| 58 |  | 4.7 | 11.8 | 11.2 | 14.5 |
| 59 |  | 5.8 | 4 | 4.7 | 42 |
| 60 |  | 1.6 | 4.2 | 2.5 | 3.8 |
| 61 |  | >40 | >40 | >40 | >40 |
| 62 |  | >40 | >40 | >40 | >40 |
| 63 |  | 13.5 | 16 | 20.2 | 14.8 |
| 64 |  | 11.6 | 10.8 | 14.1 | 9.2 |
| 65 |  | 10.6 | 18.7 | >40 | 15.5 |
| 66 |  | 22.1 | 21 | 22 | 23.7 |
| 67 |  | 15.8 | 20.2 | 22.2 | 20.2 |
| 68 |  | >40 | >40 | >40 | >40 |
| 69 |  | 11.8 | 14.4 | 14.4 | 14.8 |
| 70 |  | 22.5 | 21.3 | >40 | >40 |
| 71 |  | 7.2 | 10.7 | 10.6 | 7.3 |
| 72 |  | 11.8 | >40 | 13.1 | 11.9 |
| 73 |  | 14.4 | 16 | 18.6 | 19.2 |
| 74 |  | 21.1 | 19.6 | 18.6 | 22.9 |

**Table S3: Structure and activity against various cancer cell lines for scaffold 3.**

| **No.** | **Structure** | **IC_50_ U937** |  | **No.** | **Structure** | **IC_50_ U937** |
| --- | --- | --- | --- | --- | --- | --- |
| 75 |  | 4.8 |  | 89 |  | 21 |
| 76 |  | 15.3 |  | 90 |  | 2 |
| 77 |  | 61 |  | 91 |  | 1 |
| 78 |  | >100 |  | 92 |  | 2.7 |
| 79 |  | >100 |  | 93 |  | 4.6 |
| 80 |  | >100 |  | 94 |  | 12 |
| 81 |  | 57 |  | 95 |  | 2.8 |
| 82 |  | >100 |  | 96 |  | 6.5 |
| 83 |  | >100 |  | 97 |  | 2.7 |
| 84 |  | >100 |  | 98 |  | 59 |
| 85 |  | 1.8 |  | 99 |  | 22 |
| 86 |  | 32 |  | 100 |  | >100 |
| 87 |  | 9.5 |  | 101 |  | >100 |
| 88 |  | 14 |  |  |  |  |

**Table S4:** Structure and activity against various cancer cell lines for scaffold 4.

| **No.** | **Structure** | **IC_50_** | | | |
| --- | --- | --- | --- | --- | --- |
|  |  | **KB** | **A549** | **DU-145** | **KBvin** |
|  |  |  |  |  |  |
| 102 |  | 0.73 | 0.62 | 0.82 | 0.84 |
| 103 |  | 1.11 | 1.19 | 1.09 | 1.3 |
| 104 |  | 13.85 | 14.58 | 17.23 | 18.09 |
| 105 |  | 8.69 | 8.8 | 9.6 | 8.87 |
| 106 |  | 0.86 | 0.88 | 0.93 | 1.06 |
| 107 |  | 0.55 | 0.36 | 0.78 | 0.66 |
| 108 |  | 9.3 | 6.57 | 12.56 | 7.48 |
| 109 |  | 1.12 | 0.88 | 1.1 | 1.15 |
| 110 |  | 0.11 | 0.11 | 0.11 | 0.14 |
| 111 |  | 1.26 | 1.38 | 1.85 | 1.97 |
| 112 |  | 0.66 | 1.02 | 1.02 | 1.08 |
| 113 |  | 1.94 | 1.86 | 2.68 | 3.04 |
| 114 |  | 1.3 | 1.3 | 1.5 | 1.42 |
| 115 |  | 0.26 | 0.49 | 0.35 | - |
| 116 |  | 0.08 | 0.08 | 0.11 | - |
| 117 |  | 0.83 | 0.8 | 1.07 | - |
| 118 |  | 0.18 | 0.13 | 0.16 | - |
| 119 |  | 0.18 | 0.18 | 0.26 | - |
| 120 |  | 0.9 | 0.56 | 0.84 | - |
| 121 |  | 0.18 | 0.18 | 0.24 | 0.21 |
| 122 |  | 0.036 | 0.022 | 0.025 | 0.025 |

**Table S5: Structure and activity against various cancer cell lines for scaffold 5.**

| **No.** | **Structure** | **IC_50_** | |  | **No.** | **Structure** | **IC_50_** | |
| --- | --- | --- | --- | --- | --- | --- | --- | --- |
|  |  | **HL-60** | **HeLa** |  |  |  | **HL-60** | **HeLa** |
| 123 |  | 60 | 17 |  | 144 |  | 44 | 9 |
| 124 |  | 70 | 17 |  | 145 |  | 68 | 50 |
| 125 |  | 65 | 16 |  |  |  |  |  |
|  |  |  |  |  | 146 | R = CH_3_ | 68 | 32 |
| 126 | R =H | 65 | 49 |  | 147 | R = C_2_H_5_ | 76 | 60 |
| 127 | R = CH_3_ | 70 | 41 |  | 148 | R = C_3_H_7_ | 74 | 18 |
| 128 | R = C_2_H_5_ | 65 | 38 |  | 149 | R = C_4_H_9_ | 70 | 42 |
|  |  |  |  |  | 150 | R = C_2_H_4_OH | 72 | 44 |
| 129 | R = C_2_H_5_ | 73 | 45 |  | 151 |  | 72 | 55 |
| 130 | R = C_3_H_7_ | 68 | 42 |  | 152 |  | 76 | 44 |
| 131 9 | R = C_5_H_11_ | 47 | 24 |  | 153 |  | 67 | 12 |
|  |  |  |  |  | 154 |  | 76 | 13 |
| 132 | R = phenyl | 31 | 6 |  | 155 |  | 61 | 8 |
| 134 | R = *p*-chlorophenyl | 12 | 3 |  | 156 |  | 64 | 12 |
| 135 | R = 3,4-methylene  dioxyphenyl | 57 | 5 |  | 157 |  | 67 | 18 |
| 136 | R = styryl | 25 | 7 |  | 158 |  | 64 | 48 |
| 137 |  | 72 | 46 |  | 159 |  | 74 | 48 |
| 138 | R = 3,4-dimethoxy  phenyl | 32 | 6 |  | 160 |  | 74 | 16 |
| 139 | R = furfuryl | 68 | 5 |  |  |  |  |  |
|  |  |  |  |  |  |  |  |  |
| 140 | R = phenyl | 58 | 9 |  |  |  |  |  |
| 141 | R = *p*-chlorophenyl | 70 | 17 |  |  |  |  |  |
| 142 | R = 3,4-methylene  dioxyphenyl | 73 | 18 |  |  |  |  |  |
| 143 | R = styryl | 78 | 46 |  |  |  |  |  |

**Table S6: Structure and activity against various cancer cell lines for scaffold 6.**

| **No.** | **Structure** | **IC_50_** | | | | | | | | |
| --- | --- | --- | --- | --- | --- | --- | --- | --- | --- | --- |
|  |  | **A375** | **B16F1** | **DU 145** | **LNCaP** | **PC-3** | **PPC-1** | **WM-164** | **Fib^*^** | **RH7^**^** |
|  |  |  |  |  |  |  |  |  |  |  |
| 161 | X =CON,  R^1^=3,4,5 Trimethoxy  R^2^ = *n*-C_18_H_37_ | 4.6 | 5.9 | 4.1 | 1.6 | 4.9 | 1 | 3 | 4.7 | 14 |
| 162 | X =CON  R1=3,4,5  Trimethoxy  R2 =(Z)-Octadec-8-enyl | 2.4 | 4.7 | 1.2 | 0.7 | 1.5 | 0.5 | 1.3 | 4.8 | 8.4 |
| 163 | X =CON  R1=3,4,5  Trimethoxy  R2 =(E)-Octadec-8-enyl | 1.8 | 3.2 | 0.9 | 0.6 | 1.2 | 0.3 | 1.1 | 4 | 6.8 |
| 164 | X =CON  R1=3,4,5  Trimethoxy  R2 = n-C16H33 | 1.4 | 1.6 | - | - | - | - | 0.7 | 2.4 | 11.8 |
| 165 | X =CON  R1 = 3,5-Dimethoxy  R2 = n-C18H37 | 6.9 | 14.3 | 5.5 | 2.4 | 4.1 | 0.9 | 2.7 | 20.3 | 10 |
| 166 | X =CON  R1 =3,5- Dimethoxy  R2 =(Z)-Octadec-8-enyl | 1.6 | 3.3 | 2 | 1.3 | 2.6 | 0.6 | 1.2 | 18.3 | 9.4 |
| 167 | X =CON  R1 = 3,5-Dimethoxy  R2 =(E)-Octadec-8-enyl | 1.4 | 3.2 | 1.5 | 0.8 | 2 | 0.3 | 1.1 | 16.3 | 17.2 |
| 168 | X =CON  R1 = 2-OMe  R2 = n-C16H33 | 3 | 4.3 | 6.5 | 3.6 | 7.4 | 1.7 | 2.4 | 7.2 | 3.6 |
| 169 | X =CON  R1 = 3-OMe  R2 = n-C16H33 | 1.8 | 3 | 1.1 | 0.5 | 1 | 0.2 | 1.2 | 2.5 | 8.4 |
| 170 | X =CON  R1 = 4-OMe  R2 = n-C16H33 | 1.5 | 2.3 | 2.2 | 1.1 | 2.7 | 0.4 | 1 | 8.1 | 10 |
| 171 | X =CON  R1=NMe  R2 = n-C16H33 | 1.8 | 6.7 | 3.2 | 1.4 | 6 | 0.4 | 1.5 | 21 | 13.2 |
| 172 | X =CON  R1 =2-NHAc  R2 = n-C16H33 | 9.3 | 8 | 4.6 | 3.3 | 2.7 | 1.5 | 3.9 | 27.8 | 4.9 |
| 173 | X =CON  R1 =3-NHAc  R2 = n-C16H33 | 1.5 | 2.2 | 2.3 | 1.4 | 1.6 | 0.5 | 1.1 | 6.3 | 9.2 |
| 174 | X =CON  R1 =4-NHAc  R2 = n-C18H37 | 20.6 | 18.9 | 3.9 | 1.8 | 3.7 | 0.8 | 10.7 | >100 | >20 |
| 175 | X =CON  R1 =4-NHAc  R2 =1-Admantanyl | 137.5 | 96.5 | >20 | 5.7 | >20 | >20 | 127.5 | >100 | >20 |
| 176 | X =CON  R1 =4-NHAc  R2 =2-Admantanyl | 66 | 108.2 | >20 | 9.7 | >20 | >20 | 64.4 | >100 | 5.4 |
| 177 | X =CON  R1 =4-NHAc  R2 =9H-FLuoren-2yl | 2.1 | 3.9 | 1.9 | 3.5 | 2.1 | 1.56 | 1.7 | 28.9 | 2.6 |
| 178 | X =CON  R1 =4-NHAc  R2 =Anthracene-2-yl | 3.1 | 5.6 | 0.9 | 1.7 | 0.8 | 0.7 | 1.4 | 13.5 | 9.5 |
| 179 | X =CON  R1 =2-NHAc  R2 =4-Biphenyl | 6.2 | 5.7 | 5 | 3.5 | 7.6 | 2.9 | 4.1 | 33.4 | >20 |
| 180 | X =CON  R1 =4-NHAc  R2 =2-Benzothiazolyl | 45.9 | 55.3 | >20 | >20 | >20 | >20 | >100 | >100 | 7.6 |
| 181 | X =CON  R1 =4-NHAc  R2 =(Z)-Hexadec-9-enyl | 1.9 | 2.1 | 2.4 | 1.8 | 2.5 | 1.1 | 2 | 14 | 6.6 |
| 182 | X =CON  R1 =4-NHAc  R2 =Nonadec-10-ynyl | 2.7 | 1.6 | 1.8 | 2 | 2.1 | 0.7 | 1.3 | 7 | 8.3 |
| 183 | X =CON  R1= H  R2 =(Z)-Octadec-8-enyl | 4.9 | 6 | 1.8 | 1.6 | 2.4 | 1 | 2.4 | 11.6 | 8.9 |
| 184 | X =CON  R1= H  R2 =(E)-Octadec-8-enyl | 5.3 | 10.1 | 1.5 | 1.4 | 2.1 | 0.6 | 2.4 | 12.4 | 13 |
| 185 | X =CON  R1 =4-NHAc  R2 = n-C16H33 | 12.6 | 15 | 14.9 | 10.6 | 9 | 6.8 | 20.4 | 57.7 | >20 |
| 186 | X =CON  R1 =4-NHAc  R2 = n-C16H33 | 28 | 41.9 | >20 | >20 | >20 | >20 | 18.5 | >100 | 6.2 |
| 187 | X =CH2N  R1= H  R2 = n-C16H33 | 13 | 4.7 | 3.1 | 3.9 | 3.4 | 2.5 | 4.9 | 60 | 7.9 |
| 188 | X =CH2N  R1 =4-NHAc  R2 = n-C16H33 | 14.4 | 11.9 | 4.7 | 4.7 | 4.5 | 2.4 | 3.2 | 84.5 | 7.3 |
| 189 | X =CON  R1=4-NHSO3CH3  R2 = n-C16H33 | 50.6 | 113.6 | >20 | 4.8 | >20 | 1.1 | 18.6 | >100 | 9.4 |
| 190 | X =CON  R1=4-NHCOCH2Cl  R2 = n-C16H33 | 20.4 | 93.3 | 0.7 | 2 | 0.9 | 0.4 | 5.7 | 25.4 | >20 |
| 191 | X =CON  R1=4-NHCONH2  R2 = n-C16H33 | 3.6 | 6.5 | 3.2 | 1.8 | 2.5 | 0.5 | 1.7 | 72 | 9.4 |
| 192 | X =CON  R1 =H  R2 = n-C16H33 | 15 | 15.5 | 10.8 | 4.2 | 10 | 2.4 | 4.4 | 29.8 | 6.7 |
| 193 | X =CON  R1 =4-NHAc  R2 = n-C16H33 | 2.1 | 2.2 | 1.7 | 1 | 1.2 | 0.4 | 1.1 | 16 | >20 |
| 194 |  | >100 | >100 | - | - | - | - | >100 | >100 | 6.8 |
|  |  |  |  |  |  |  |  |  |  |  |
| 195 | R =n-C8H17 | - | - | 17.3 | 2.7 | >20 | 14 | - | - | >20 |
| 196 | R =n-C10H21(4R) | - | - | 4.1 | 2.2 | 4.4 | 2.1 | - | - | 6 |
| 197 | R =n-C12H25(4R) | - | - | 2.8 | 1.4 | 2.9 | 1 | - | - | 6.5 |
| 198 | R =n-C14H29 (4R) | - | - | 2.2 | 2 | 2.6 | 0.6 | - | - | 6.1 |
| 199 | R =n-C14H29 (4S) | - | - | 2.1 | 1.9 | 2.9 | 0.7 | - | - | 5.8 |

^*^ Fibroblast ^**^ RH7777

**Table S7:** Structure and activity against various cancer cell lines for scaffold 7.

| **No.** | **Structure** | **IC_50_** |  | **No.** | **Structure** | **IC_50_** |
| --- | --- | --- | --- | --- | --- | --- |
|  |  | **LNCaP** |  |  |  | **LNCaP** |
|  |  |  |  | 214 | A | 0.001 |
| 200 | R =H, A = CH | - |  | 215 | B | 0.001 |
| 201 | R =Ph, A = CH | 1.4 |  |  |  |  |
| 202 | R =H, A = N | 6.5 |  | 216 | Positions = *m-* | 0.6 |
|  |  |  |  | 217 | Positions = *o-* | >60 |
| 203 | X = OH, Y = OH | 0.077 |  | 218 |  | 0.004 |
| 204 | X = OH, Y = H | 0.2 |  |  |  |  |
| 205 | X = H, Y = OH | 1.8 |  | 219 | R = -C(O)NH_2_ | 0.01 |
|  | A  B |  |  | 220 | R = -CH_2_NH_2_ | 0.075 |
| 206 | R = NMe_2_ A | 0.004 |  | 221 | R = NH_2_ | 0.24 |
| 207 | R = NMe_2_ B | 0.001 |  |  |  |  |
| 208 | A | 0.001 |  | 222 |  | 0.005 |
| 209 | B | <0.001 |  | 223 |  | 0.002 |
| 210 | A | 0.008 |  | 224 |  | 0.002 |
| 211 | B | 0.001 |  | 225 |  | 0.003 |
| 212 | A | 0.009 |  | 226 |  | 0.11 |
| 213 | B | <0.001 |  |  |  |  |
|  |  |  |  |  |  |  |

**Table S8:** Structure and activity against various cancer cell lines for scaffold 8.

| **No.** | **Structure** | **IC_50_** | | | | | |
| --- | --- | --- | --- | --- | --- | --- | --- |
|  |  | **B16-F1** | **A375** | **DU-145** | **LNCaP** | **PC-3** | **PPC-1** |
|  |  |  |  |  |  |  |  |
| 227 | A= *p-*NHAc-Ph  B = TZD  C = C_16_H_33_ | 2.2 | 2.1 | 1.7 | 1 | 1.2 | 0.4 |
| 228 | A= *p-*NHAc-Ph  B = TZD  C = 9H-fluoren-1-yl | 3.9 | 2.1 | 1.9 | 3.5 | 2.1 | 1.6 |
| 229 | A= Ph  B = TZD  C = 3,4,5-trimethoxy-Ph | >100 | >100 | >20 | >20 | >20 | >20 |
| 230 | A = 3,4,5-trimethoxy-Ph  B = TZD  C = 3,4,5-trimethoxy-Ph | >100 | >100 | >20 | >20 | >20 | >20 |
| 231 | A= Ph  B = TZL  C = 3,4,5-trimethoxy-Ph | 38.3 | 22.8 | >20 | >20 | >20 | 5.3 |
| 232 | A= Ph  B = TZL  C = 3,4,5-trimethoxy-Ph | 30.4 | 13.6 | >20 | 16.8 | 13.2 | 3.4 |
| 233 | A= Ph  B = TZ  C = 3,4,5-trimethoxy-Ph | >100 | >100 | >20 | >20 | >20 | >20 |
| 234 | A= Ph  B = TZ  C = 3,4,5-trimethoxy-Ph | 0.055 | 0.028 | 0.071 | 0.028 | 0.021 | 0.043 |
|  |  |  |  |  |  |  |  |
| 235 | Ph | >100 | >100 | >20 | >20 | >20 | >20 |
| 236 | 4-methoxy-Ph | >100 | >100 | >20 | >20 | >20 | >20 |
| 237 | 3-methoxy-Ph | >100 | >100 | >20 | >20 | >20 | >20 |
| 238 | 2-methoxy-Ph | 59.4 | 70.3 | >20 | >20 | >20 | >20 |
| 239 | 3,4-dimethoxy-Ph | >100 | >100 | >20 | >20 | >20 | >20 |
| 240 | 3,5-dimethoxy-Ph | 0.35 | 0.17 | 0.424 | 0.323 | 0.301 | 0.242 |
| 241 | 2-Fluoro-Ph | >100 | >100 | >20 | >20 | >20 | >20 |
| 242 | Hexadecyl | 18.6 | 16 | >20 | >20 | >20 | >20 |
|  |  |  |  |  |  |  |  |
| 243 | 4-Methyl-Ph | 0.021 | 0.011 | 0.007 | 0.006 | 0.005 | 0.006 |
| 244 | 2-Fluoro-Ph | 0.027 | 0.03 | 0.114 | 0.053 | 0.082 | 0.052 |
| 245 | 3-Fluoro-Ph | 0.287 | 0.304 | 0.035 | 0.011 | 0.024 | 0.021 |
| 246 | 4-Fluoro-Ph | 0.043 | 0.033 | 0.012 | 0.006 | 0.013 | 0.008 |
| 247 | 3,4-dimethoxy-Ph | 0.161 | 0.034 | 0.102 | 0.038 | 0.069 | 0.056 |
| 248 | 4-Nitro-Ph | 0.056 | 0.038 | 0.095 | 0.039 | 0.056 | 0.034 |
| 249 | 4-Cyano-Ph | 0.053 | 0.059 | 0.052 | 0.015 | 0.03 | 0.019 |
| 250 | 4-Trifluoromethyl-Ph | 0.092 | 0.023 | 0.05 | 0.094 | 0.058 | 0.76 |
| 251 | 4-Bromo-Ph | 0.032 | 0.013 | 0.021 | 0.044 | 0.018 | 0.021 |
| 252 | 4-Ethyl-Ph | 0.07 | 0.017 | 0.031 | 0.06 | 0.027 | 0.022 |
| 253 | 4-Pyridine | >100 | >100 | >20 | >20 | >20 | >20 |
| 254 | 2-Pyrimidine | 2.3 | 4.1 | 2.813 | 2.37 | 2.657 | 1.186 |
| 255 | 2-Thienyl | 0.038 | 0.02 | 0.022 | 0.009 | 0.017 | 0.013 |
| 256 | H | >100 | >100 | >20 | >20 | >20 | >20 |
| 257 | 4-Carboxylic acid-Ph | >100 | >100 | >20 | >20 | >20 | >20 |
| 258 | 4-Methylcarboxylate-Ph | >100 | >100 | >20 | >20 | >20 | >20 |
| 259 |  | >100 | >100 | >20 | >20 | >20 | >20 |
| 260 |  | >100 | - | - | - | - | - |

**Table S9: Structure and activity against various cancer cell lines for scaffold 9.**

| **No.** | **Structure** | **IC_50_** | |
| --- | --- | --- | --- |
|  |  | **DU-145** | **MB-231** |
|  |  |  |  |
| 261 | R_1_= MeO, R_2_ = H X = OH, Y = NA, Z = NA | 1.22 | 0.94 |
| 262 | R_1_= MeO, R_2_ = SO_2_NH_2_ X = O, Y = SO_2_, Z = NH_2_ | 0.34 | 0.28 |
| 263 | R_1_= Et, R_2_ = SO_2_NH_2_ X = O, Y = SO_2_, Z = NH_2_ | 0.21 | 0.21 |
| 264 | R_1_= MeO, R_2_ = SO_2_NH_2_ X = CH_2_, Y = CN, Z = NA | 0.062 | 0.071 |
| 265 | R_1_= Et, R_2_ = SO_2_NH_2_ X = CH_2_, Y = CN, Z = NA | 0.054 | 0.141 |
| 266 | R_1_= MeO, R_2_ = SO_2_NH_2_ X = =O, Y = NA, Z = NA | 0.46 | 0.12 |
| 267 | R_1_= MeO, R_2_ = H X = O, Y = SO_2_, Z = CH_3_ | 4.2 | 2.37 |
| 268 | R_1_= MeO, R_2_ = SO_2_NH_2_ X = O, Y = SO_2_, Z = CH_3_ | 0.52 | 0.23 |
| 269 | R_1_= Et, R_2_ = H X = O, Y = SO_2_, Z = CH_3_ | 5.58 | - |
| 270 | R_1_= Et, R_2_ = SO_2_NH_2_ X = O, Y = SO_2_, Z = CH_3_ | 0.57 | 0.2 |
| 271 | R_1_= Et, R_2_ = SO_2_NH_2_ X = CH_2_, Y = SO_2_, Z = CH_3_ | 0.19 | 0.23 |
| 272 | R_1_= MeO, R_2_ = H X = NH_2_, Y = NA, Z = NA | 2.64 | 3.56 |
| 273 | R_1_= MeO, R_2_ = H X = NH, Y = SO_2_, Z = NH_2_ | 58.6 | - |
| 274 | R_1_= MeO, R_2_ = SO_2_NH_2_ X = NH, Y = SO_2_, Z = NH_2_ | 6.13 | 5.6 |
| 275 | R_1_= Et, R_2_ = H X = NH_2_, Y = NA, Z = NA | 60.8 | - |
| 276 | R_1_= Et, R_2_ = H X = NH, Y = SO_2_, Z = CH_3_ | >100 | - |
| 277 | R_1_= MeO, R_2_ = H X = NH, Y = SO_2_, Z = CH_3_ | >100 | - |
| 278 | R_1_= MeO, R_2_ = SO_2_NH_2_ X = NH, Y = SO_2_, Z = CH_3_ | 9.9 | 5.2 |
| 279 | R_1_= Et, R_2_ = H X = NH, Y = SO_2_, Z = CH_3_ | >100 | - |
| 280 | R_1_= Et, R_2_ = SO_2_NH_2_ X = NH, Y = SO_2_, Z = CH_3_ | 0.84 | 3.52 |
| 281 | R_1_= Et, R_2_ = H X = NA, Y = SO_2_, Z = CH_3_ | >100 | >100 |
| 282 | R_1_= Et, R_2_ = SO_2_NH_2_ X = NA, Y = SO_2_, Z = CH_3_ | 0.11 | 0.23 |
| 283 | R_1_= Et, R_2_ = H X = (CH_2_)_2_, Y= NMe_2_, Z = NA | 7.96 | 4.74 |
| 284 | R_1_= Et, R_2_ = SO_2_NH_2_ X = (CH_2_)_2_, Y= NMe_2_, Z = NA | 3.54 | 2.96 |
| 285 | R_1_= Et, R_2_ = H X = CH_2_, Y= NMe_2_, Z = NA | 10.1 | 9.5 |
| 286 | R_1_= Et, R_2_ = SO_2_NH_2_ X = CH_2_, Y= NMe_2_, Z = NA | 3.05 | 4.03 |
| 287 | R_1_= Et, R_2_ = H X = CH_2_, Y= NO_2_, Z = NA | 12.2 | 10.4 |
| 288 | R_1_= Et, R_2_ = SO_2_NH_2_ X = CH_2_, Y= NO_2_, Z = NA | 0.14 | 0.17 |
| 289 | R_1_= MeO, R_2_ = H X = CH_2_, Y= CN, Z = NA | 0.49 | 0.12 |
| 290 | R_1_= Me, R_2_ = SO_2_NH_2_ X = O, Y = SO_2_, Z = NH_2_ | 0.38 | - |
| 291 | R_1_= *^n^*Pr, R_2_ = SO_2_NH_2_ X = O, Y = SO_2_, Z = NH_2_ | 3.4 | - |
| 292 | R_1_= MeO, R_2_ = SO_2_NH_2_ X = OH, Y = NA, Z = NA | 0.19 | - |
| 293 | R_1_= Et, R_2_ = SO_2_NH_2_ X = OH, Y = NA, Z = NA | <0.01 | - |
| 294 | R_1_= MeO, R_2_ = SO_2_NH_2_ X = =O, Y = NA, Z = NA | >10 | - |
| 295 | R_1_= MeO, R_2_ = H X = O, Y = SO_2_, Z = NH_2_ | 0.34 | - |

**Table S10: Structure and activity against various cancer cell lines for scaffold 10.**

| **No.** | **Structure** | **IC_50_** | | | | | | | | |
| --- | --- | --- | --- | --- | --- | --- | --- | --- | --- | --- |
|  |  | **SF-539** | **HOP-62** | **HCT-116** | **UACC-62** | **OVCAR-3** | **SN12C** | **MCF-7** | | **DU-145** |
| 296 |  | 0.01 | 0.01 | 0.03 | 0.01 | 0.22 | 2 | 0.01 | 0.01 | |
| 297 |  | 0.04 | 0.02 | 0.1 | 0.03 | 0.5 | <0.01 | <0.01 | <0.01 | |
| 298 |  | 0.04 | 1.78 | 1.15 | 0.03 | 74.1 | 0.813 | 0.37 | 0.155 | |
| 299 |  | 0.037 | <0.01 | <0.01 | <0.01 | 0.085 | <0.01 | 0.01 | <0.01 | |
| 300 |  | >100 | >100 | 57.3 | >100 | >100 | >100 | 60.3 | >100 | |
| 301 |  | - | - | - | - | - | - | - | - | |
| 302 |  | 2.69 | 3.31 | 1.17 | 1.66 | 2.19 | 2.51 | 0.3 | 2.19 | |
| 303 |  | 4.37 | 19.5 | >100 | 21.4 | 18.6 | 81.3 | 0.3 | >100 | |
| 304 |  | - | - | - | - | - | - | - | - | |
| 305 |  | 0.81 | 3.8 | 1.33 | 0.99 | 1.7 | 2.57 | 0.3 | 0.93 | |
| 306 |  | 4.26 | 5.69 | 2.63 | 1.16 | 1.95 | 5.75 | 0.44 | 2.04 | |
| 307 |  | 0.49 | 1.01 | 0.82 | 0.46 | 1.84 | 0.94 | 0.27 | 1.02 | |
| 308 |  | - | - | - | - | - | - | - | - | |
| 309 |  | - | - | - | - | - | - | - | - | |
| 310 |  | - | - | - | - | - | - | - | - | |
| 311 |  | 0.49 | 1.01 | 0.82 | 0.46 | 1.84 | 0.94 | 0.27 | 1.02 | |
| 312 |  | 1.95 | 0.57 | 1.95 | 0.93 | 7.08 | 12.6 | 0.19 | 2.88 | |
| 313 |  | 1.15 | 0.54 | 1.42 | 1.15 | 1.84 | 1.13 | 0.06 | 0.76 | |
| 314 |  | - | 2.14 | - | - | - | - | - | - | |
| 315 |  | 1.86 | 17 | 1.95 | 1.625 | 1.95 | 1.86 | 1.05 | 1.82 | |
| 316 |  | 11.7 | 0.66 | 1.45 | 1.62 | 16.2 | 2.34 | 0.38 | 51.3 | |
| 317 |  | 1.35 | - | 0.95 | 0.47 | 1.55 | 1.91 | 0.28 | 1.82 | |
| 318 |  | - | 0.48 | - | - | - | - | - | - | |
| 319 |  | 0.6 | 5.01 | 0.85 | 0.35 | >100 | 0.79 | 0.07 | - | |
| 320 |  | 2.14 | - | 1.32 | 3.39 | 4.26 | 5.89 | 0.48 | 2.57 | |
| 321 |  | - | - | - | - | - | - | - | - | |
| 322 |  | - | - | - | - | - | - | - | - | |
| 323 |  | - | - | - | - | - | - | - | - | |
| 324 |  | - | - | - | - | - | - | - | - | |
| 325 |  | - | - | - | - | - | - | - | - | |
| 326 |  | 6.46 | 20.1 | 1.8 | - | 14.6 | 16 | 1.8 | 2.63 | |
| 327 |  | - | - | - | 16.2 | - | - | - | - | |
| 328 |  | 1.1 | 36.7 | 3.05 | >100 | 4.68 | 1.8 | 2.21 | 6.24 | |
| 329 |  | 9.23 | 11.2 | 18.4 | 14.2 | 16.8 | 20.2 | 1.62 | 12.2 | |
| 330 |  | - | - | - | - | - | - | - | - | |
| 331 |  | - | - | - | - | - | - | - | - | |
| 332 |  | 1.58 | 2.29 | 0.15 | 1.41 | 1.51 | 1.78 | 3.31 | 2.75 | |
| 333 |  | 1.26 | 1.05 | 1.1 | 0.74 | 1.86 | - | 0.37 | 1.66 | |
| 334 |  | 4.36 | 7.24 | 2.14 | 2.04 | 6.46 | 10.2 | 0.23 | - | |
| 335 |  | 0.39 | 0.46 | 0.4 | 0.31 | 0.69 | 1.54 | 0.19 | 0.48 | |
| 336 |  | 1.86 | 2 | 1.82 | 1.91 | 2.04 | 2.09 | 1.66 | - | |
| 337 |  | 15.1 | 18.1 | 13.5 | 17 | 15.1 | 19 | 10.9 | 23.4 | |
| 338 |  | - | - | - | - | - | - | - | - | |
| 339 |  | - | - | - | - | - | - | - | - | |
| 340 |  | 1.51 | 1.86 | 1.41 | 15.5 | 4.17 | 13.8 | 1 | 9.77 | |
| 341 |  | 1.95 | 1.66 | 1.55 | 3.09 | 2.63 | 3.71 | 0.37 | 3.98 | |
| 342 |  | 1.9 | 2.51 | 1.58 | 7.24 | 4.9 | 6.03 | 0.55 | 1.02 | |

**Table S11: Details of the descriptors involved in the different combination in QSAR studies**

| **No.** | **Descriptors** | **Full Name of the Descriptors** | **Nature** | **QSAR Model** |
| --- | --- | --- | --- | --- |
| 1 | MiVH | Min valency of a H atom | Quantum chemical | M1 |
| 2 | ZXS/ZXR | ZX Shadow/ZX rectangle | Geometrical | M1, M2, M6, M12, M19, M21 |
| 3 | MiNRC | Min nucleoph. react. index for a C atom | Quantum chemical | M1 |
| 4 | MaPCH | Max partial charge for a H atom [Zefirov's PC] | Electrostatic | M2, M6, **S6** |
| 5 | MA1ERN | Max 1-electron react. index for a N atom | Quantum chemical | M2 |
| 6 | HC-2/T_Z_ | HACA-2/TMSA [Zefirov's PC] | Electrostatic | M4 |
| 7 | Mi1ERN | Min 1-electron react. index for a N atom | Quantum chemical | M4 |
| 8 | MiNACH | Min net atomic charge for a H atom | Quantum chemical | M4 |
| 9 | PS-3A_Z_ | PPSA-3 Atomic charge weighted PPSA [Zefirov's PC] | Electrostatic | M5, **S7** |
| 10 | KHI_3_ | Kier&Hall index (order 3) | Topological | M5, **S6** |
| 11 | MiNRN | Min nucleoph. react. index for a N atom | Quantum chemical | M5 |
| 12 | FS-2P_Z_ | FNSA-2 Fractional PNSA (PNSA-2/TMSA) [Zefirov's PC] | Electrostatic | M6 |
| 13 | MaPCN | Max partial charge for a N atom [Zefirov's PC] | Electrostatic | M8 |
| 14 | MaPC | Max partial charge (Q_MAX_) | Electrostatic | M8 |
| 15 | MaVO | Max valency of a O atom | Quantum chemical | M8, M26, M16 |
| 16 | GI_AP_ | Gravitation index (all pairs) | Constitutional | M9 |
| 17 | TPCCMD | Tot point-charge comp. of the molecular dipole | Quantum chemical | M9, **S10**, M12 |
| 18 | MaBO | Max bond order of a O atom | Quantum chemical | M9 |
| 19 | ZXS | ZX Shadow | Geometrical | M10 |
| 20 | SCI_0_ | Structural Information content (order 0) | Topological | M10 |
| 21 | RNN | Relative number of N atoms | Constitutional | M10, S8 |
| 22 | PS-1_Z_ | PPSA-1 Partial positive surface area [Zefirov's PC] | Electrostatic | M11 |
| 23 | RPCG_Z_ | RPCG Relative positive charge (QMPOS/QTPLUS) [Zefirov's PC] | Electrostatic | M11 |
| 24 | MaNACH | Max net atomic charge for a H atom | Quantum chemical | M11 |
| 25 | THCMD | Tot hybridization comp. of the molecular dipole | Quantum chemical | M26, M27 |
| 26 | RNCS_Q_ | RNCS Relative negative charged SA (SAMNEG^*^RNCG) [Quantum-Chemical PC] | Quantum chemical | M12 |
| 27 | YZS | YZ Shadow | Geometrical | M15 |
| 28 | FBCS_Q_ | FHBCA Fractional HBSA (HBSA/TMSA) [Quantum-Chemical PC] | Quantum chemical | M15 |
| 29 | MaPBO | Max PI-PI bond order | Quantum chemical | M15 |
| 30 | RPCS_Z_ | RPCS Relative positive charged SA (SAMPOS^*^RPCG) [Zefirov's PC] | Electrostatic | M17, M18, M24**, S2** |
| 31 | MiVO | Min valency of a O atom | Quantum chemical | M17, M13, **S1** |
| 32 | MiBOO | Min (>0.1) bond order of a O atom | Quantum chemical | M17, M22 |
| 33 | RPCS_Q_ | RPCS Relative positive charged SA (SAMPOS^*^RPCG) [Quantum-Chemical PC] | Quantum chemical | M19 |
| 34 | NF | Number of F atom | Constitutional | M19 |
| 35 | HC-1/T | HACA-1/TMSA [Zefirov's PC] | Electrostatic | M20, **S4** |
| 36 | PP/SD | Polarity parameter / square distance | Electrostatic | M20, **S4** |
| 37 | MaNACC | Max net atomic charge for a C atom | Quantum chemical | M20, **S4** |
| 38 | MiNRO | Min nucleoph. react. index for a O atom | Quantum chemical | M21 |
| 39 | Mi1ERC | Min 1-electron react. index for a C atom | Quantum chemical | M21 |
| 40 | NN | Number of N atom | Constitutional | M23, M3 |
| 41 | XYS/XYR | XY Shadow/XY rectangle | Geometrical | M23 |
| 42 | MiVC | Min valency of a C atom | Quantum chemical | M23, M3 |
| 43 | MSA | Molecular surface area | Geometrical | M26 |
| 44 | RNO | Relative number of O atoms | Constitutional | M29, M16 |
| 45 | HS-1_Z_ | HASA-1 [Zefirov's PC] | Electrostatic | M29 |
| 46 | H-HC-2/ST | HA dependent HDCA-2/SQRT(TMSA) [Zefirov's PC] | Electrostatic | M29 |
| 47 | W-1wP | WNSA-1 Weighted PNSA (PNSA1^*^TMSA/1000) [Zefirov's PC] | Electrostatic | M3 |
| 48 | MiBOH | Min (>0.1) bond order of a H atom | Quantum chemical | M7, M18, **S3** |
| 49 | MiVN | Min valency of a N atom | Quantum chemical | M7, M27, **S3** |
| 50 | H-HD-2/T | HA dependent HDSA-2/TMSA [Zefirov's PC] | Electrostatic | M7,**S9, S3** |
| 51 | MaVC | Max valency of a C atom | Quantum chemical | M13, M14, **S1** |
| 52 | A1ERC | Avg 1-electron react. index for a C atom | Quantum chemical | M13, **S1** |
| 53 | AVN | Avg valency of a N atom | Quantum chemical | M14 |
| 54 | MiNACN | Min net atomic charge for a H atom | Quantum chemical | M14 |
| 55 | RNH | Relative number of H atoms | Constitutional | M16 |
| 56 | MaERC | Max electroph. react. index for a C atom | Quantum chemical | M22 |
| 57 | MaVH | Max valency of a H atom | Quantum chemical | M22 |
| 58 | H-HC-1_Q_ | HA dependent HDCA-1 [Quantum-Chemical PC] | Quantum chemical | M24, **S2** |
| 59 | MiERC | Min electroph. react. index for a C atom | Quantum chemical | M24, **S2** |
| 60 | ANRN | Avg nucleoph. react. index for a N atom | Quantum chemical | M25 |
| 61 | H-1E | HOMO-1 energy | Quantum chemical | M25 |
| 62 | MaBON | Max bond order of a N atom | Quantum chemical | M25 |
| 63 | L1E | LUMO+1 energy | Quantum chemical | M27 |
| 64 | MI-A | Moment of inertia | Geometrical | M28 |
| 65 | MaBOC | Max bond order of C atom | Quantum chemical | M28, **S10** |
| 66 | AERN | Avg electroph. react. index for a N atom | Quantum chemical | M28 |
| 67 | RNC | Relative number of C atom | Constitutional | **S7** |
| 68 | HS-1/T | HASA-1/TMSA [Zefirov's PC] | Electrostatic | **S7** |
| 69 | Mi1ERS | Min 1-electron react. index for a S atom | Quantum chemical | **S8** |
| 70 | MaERN | Max electroph. react. index for a N atom | Quantum chemical | **S8** |
| 71 | ABC | Avg bond order of a C atom | Quantum chemical | **S9** |
| 72 | HE | HOMO energy | Quantum chemical | **S9** |
| 73 | MiERO | Min electroph. react. index for a O atom | Quantum chemical | **S10** |
| 74 | ACI_2_ | Average Complementary Information content (order 2) | Topological | **S5** |
| 75 | MiPCO | Min partial charge for a O atom [Zefirov's PC] | Electrostatic | **S5** |
| 76 | RNAB | Relative number of aromatic bonds | Constitutional | **S5** |
| 77 | MV/X | Molecular volume / XYZ Box | Geometrical | **S6** |
| 78 | RNCS_Z_ | RNCS Relative negative charged SA (SAMNEG^*^RNCG) [Zefirov's PC] | Electrostatic | M18 |

**Table S12: Descriptors, experimental and predicted pIC_50_ values and their residuals for test set 1 compounds in cell line based QSAR model against A375**

| **No.** | **MiVH** | **ZXS/ZXR** | **MiNRC** | **Exp.** | **Pred.** | **Res.** |
| --- | --- | --- | --- | --- | --- | --- |
| 191 | 8.29E-01 | 6.25E-01 | 0.00E+00 | 1.824 | 2.24 | -0.41 |
| 192 | 8.19E-01 | 5.89E-01 | 1.49E-11 | 2.678 | 2.25 | 0.43 |
| 197 | 8.35E-01 | 7.19E-01 | 1.22E-08 |  | 1.78 |  |
| 196 | 8.35E-01 | 7.25E-01 | 5.03E-08 |  | 1.74 |  |
| 195 | 8.35E-01 | 7.15E-01 | 5.11E-08 |  | 1.80 |  |
| 194 | 8.35E-01 | 7.29E-01 | 2.09E-07 |  | 1.71 |  |
| 198 | 8.49E-01 | 6.66E-01 | 2.12E-10 |  | 2.39 |  |
| 165 | 8.30E-01 | 5.68E-01 | 0.00E+00 | 2.161 | 2.61 | -0.45 |
| 170 | 8.29E-01 | 6.33E-01 | 3.10E-11 | 2.824 | 2.19 | 0.64 |
| 172 | 8.27E-01 | 6.24E-01 | 2.87E-10 | 2.032 | 2.20 | -0.17 |
| 173 | 8.19E-01 | 6.32E-01 | 1.73E-10 | 2.824 | 1.99 | 0.84 |
| 174 | 8.19E-01 | 6.66E-01 | 0.00E+00 | 1.686 | 1.78 | -0.09 |
| 175 | 8.17E-01 | 7.36E-01 | 3.49E-06 | 0.862 | 1.27 | -0.40 |
| 176 | 8.19E-01 | 7.01E-01 | 6.08E-08 | 1.181 | 1.56 | -0.38 |
| 177 | 8.17E-01 | 6.75E-01 | 5.37E-08 | 2.678 | 1.68 | 1.00 |
| 180 | 8.17E-01 | 6.18E-01 | 1.30E-04 | 1.338 | 0.77 | 0.57 |
| 182 | 8.19E-01 | 6.07E-01 | 0.00E+00 | 2.569 | 2.14 | 0.43 |
| 185 | 8.18E-01 | 5.71E-01 | 4.40E-11 | 1.900 | 2.34 | -0.44 |
| 186 | 8.18E-01 | 6.40E-01 | 0.00E+00 | 1.553 | 1.92 | -0.36 |
| 187 | 8.49E-01 | 6.90E-01 | 1.08E-09 | 1.886 | 2.25 | -0.36 |
| 188 | 8.19E-01 | 5.78E-01 | 2.26E-10 | 1.842 | 2.32 | -0.48 |
| 189 | 8.05E-01 | 6.17E-01 | 4.45E-11 | 1.296 | 1.79 | -0.49 |
| 190 | 8.15E-01 | 6.11E-01 | 4.37E-11 | 1.690 | 2.03 | -0.34 |
| 191 | 8.12E-01 | 5.98E-01 | 2.98E-11 | 2.444 | 2.05 | 0.39 |
| 195 | 8.09E-01 | 6.81E-01 | 5.44E-04 |  | -3.81 |  |
| 229 | 8.17E-01 | 6.18E-01 | 1.39E-07 |  | 2.03 |  |
| 230 | 8.17E-01 | 6.62E-01 | 8.08E-08 |  | 1.76 |  |
| 231 | 8.19E-01 | 5.42E-01 | 4.76E-05 | 1.642 | 2.08 | -0.44 |
| 233 | 8.16E-01 | 6.79E-01 | 2.82E-05 |  | 1.36 |  |
| 235 | 9.19E-01 | 7.01E-01 | 1.47E-05 |  | 3.48 |  |
| 236 | 9.20E-01 | 7.46E-01 | 6.04E-07 |  | 3.36 |  |
| 237 | 9.19E-01 | 5.66E-01 | 2.86E-05 |  | 4.18 |  |
| 239 | 9.19E-01 | 6.92E-01 | 1.17E-05 |  | 3.56 |  |
| 241 | 9.18E-01 | 6.37E-01 | 5.60E-05 |  | 3.45 |  |
| 242 | 9.19E-01 | 8.62E-01 | 1.38E-10 | 1.796 | 2.62 | -0.83 |
| 244 | 9.24E-01 | 6.61E-01 | 6.51E-07 | 4.523 | 3.97 | 0.56 |
| 245 | 9.27E-01 | 6.37E-01 | 1.46E-06 | 3.517 | 4.17 | -0.65 |
| 247 | 9.27E-01 | 5.29E-01 | 8.59E-08 | 4.469 | 4.85 | -0.38 |
| 248 | 9.26E-01 | 6.39E-01 | 1.39E-05 | 4.420 | 4.01 | 0.41 |
| 257 | 7.48E-01 | 7.80E-01 | 8.13E-06 |  | -0.48 |  |
| 240 | 9.26E-01 | 6.23E-01 | 3.96E-05 | 3.770 | 3.86 | -0.09 |
| 250 | 9.27E-01 | 6.50E-01 | 7.91E-06 | 4.638 | 4.02 | 0.61 |
| 252 | 9.28E-01 | 6.68E-01 | 1.45E-06 | 4.770 | 4.00 | 0.77 |
| 253 | 9.26E-01 | 5.48E-01 | 7.45E-06 |  | 4.64 |  |
| 254 | 9.19E-01 | 6.94E-01 | 2.46E-05 | 2.387 | 3.43 | -1.04 |
| 255 | 9.27E-01 | 6.91E-01 | 3.64E-07 | 4.699 | 3.84 | 0.86 |
| 256 | 9.28E-01 | 6.97E-01 | 1.28E-06 |  | 3.82 |  |
| 259 | 7.40E-01 | 7.41E-01 | 3.29E-04 |  | -3.52 |  |
| 227 | 8.18E-01 | 6.66E-01 | 3.39E-10 | 2.678 | 1.75 | 0.92 |
| 163 | 8.30E-01 | 5.56E-01 | 0.00E+00 | 2.745 | 2.68 | 0.06 |
| 249 | 9.26E-01 | 6.34E-01 | 3.10E-05 | 4.229 | 3.88 | 0.35 |
| 246 | 9.27E-01 | 5.29E-01 | 8.59E-08 | 4.482 | 4.85 | -0.37 |
| 183 | 8.29E-01 | 6.04E-01 | 0.00E+00 | 2.310 | 2.37 | -0.06 |
| 238 | 9.29E-01 | 7.31E-01 | 2.37E-04 | 1.153 | 1.34 | -0.18 |
| 232 | 8.17E-01 | 6.08E-01 | 8.84E-06 | 1.867 | 2.01 | -0.14 |
| 161 | 8.30E-01 | 5.65E-01 | 0.00E+00 | 2.337 | 2.63 | -0.29 |
| 179# | 8.18E-01 | 7.11E-01 | 4.04E-05 | 2.208 | 1.08 | 1.13 |
| 234* | 9.20E-01 | 5.70E-01 | 1.57E-05 | 4.553 | 4.30 | 0.25 |
| 184* | 8.29E-01 | 6.49E-01 | 0.00E+00 | 2.276 | 2.09 | 0.19 |
| 228* | 8.17E-01 | 5.52E-01 | 7.54E-07 | 2.678 | 2.43 | 0.25 |
| 168* | 8.32E-01 | 5.90E-01 | 9.16E-11 | 2.523 | 2.51 | 0.01 |
| 178* | 8.16E-01 | 5.41E-01 | 1.33E-06 | 2.509 | 2.47 | 0.04 |
| 169* | 8.30E-01 | 5.89E-01 | 4.49E-11 | 2.745 | 2.48 | 0.27 |
| 181* | 8.19E-01 | 5.76E-01 | 0.00E+00 | 2.721 | 2.33 | 0.39 |
| 251* | 9.27E-01 | 5.07E-01 | 2.27E-06 | 4.886 | 4.97 | -0.08 |
| 166* | 8.30E-01 | 5.68E-01 | 0.00E+00 | 2.796 | 2.61 | 0.19 |
| 243* | 9.28E-01 | 5.39E-01 | 1.59E-06 | 4.959 | 4.79 | 0.17 |
| 167* | 8.30E-01 | 5.53E-01 | 0.00E+00 | 2.854 | 2.70 | 0.15 |
| 162* | 8.30E-01 | 5.31E-01 | 0.00E+00 | 2.620 | 2.84 | -0.22 |

**Table S13: Descriptor, experimental and predicted pIC_50_ values and their residuals for test set 1 compounds in cell line based QSAR model against A549**

| **No.** | **RPCS _Z_** | **MiVO** | **MiBOO** | **Exp.** | **Pred.** | **Res.** |
| --- | --- | --- | --- | --- | --- | --- |
| 2 | 3.71E-01 | 2.00E+00 | 1.24E-01 | 2.420 | 2.33 | 0.10 |
| 4 | 2.56E-01 | 1.99E+00 | 1.23E-01 |  | 2.42 |  |
| 1 | 3.05E-01 | 2.01E+00 | 1.27E-01 | 2.620 | 2.33 | 0.29 |
| 3 | 4.26E-01 | 2.00E+00 | 1.26E-01 | 2.252 | 2.30 | -0.05 |
| 5 | 2.60E-01 | 2.00E+00 | 1.24E-01 | 2.409 | 2.38 | 0.03 |
| 6 | 1.64E-01 | 1.98E+00 | 1.25E-01 |  | 2.50 |  |
| 7 | 2.77E-01 | 1.99E+00 | 1.25E-01 |  | 2.41 |  |
| 9 | 2.26E-01 | 2.00E+00 | 1.25E-01 |  | 2.40 |  |
| 10 | 1.99E-01 | 1.99E+00 | 1.24E-01 | 2.495 | 2.45 | 0.05 |
| 11 | 0.00E+00 | 2.00E+00 | 1.24E-01 | 2.000 | 2.51 | -0.51 |
| 12 | 0.00E+00 | 2.00E+00 | 1.24E-01 | 2.022 | 2.51 | -0.49 |
| 13 | 2.38E-01 | 2.00E+00 | 1.24E-01 | 2.071 | 2.39 | -0.32 |
| 14 | 4.11E-01 | 1.98E+00 | 1.18E-01 | 2.229 | 2.36 | -0.13 |
| 15 | 2.93E-01 | 1.96E+00 | 1.05E-01 | 2.387 | 2.46 | -0.07 |
| 16 | 0.00E+00 | 2.00E+00 | 1.24E-01 | 3.046 | 2.51 | 0.53 |
| 17 | 0.00E+00 | 1.99E+00 | 1.25E-01 | 2.699 | 2.55 | 0.15 |
| 18 | 7.22E-02 | 1.99E+00 | 1.08E-01 | 2.310 | 2.47 | -0.16 |
| 19 | 2.92E-01 | 1.96E+00 | 1.20E-01 | 2.721 | 2.50 | 0.22 |
| 24 | 0.00E+00 | 1.96E+00 | 1.20E-01 | 2.509 | 2.65 | -0.14 |
| 22 | 3.02E-01 | 1.96E+00 | 1.19E-01 | 2.796 | 2.49 | 0.31 |
| 23 | 2.86E-01 | 1.96E+00 | 1.18E-01 | 2.260 | 2.50 | -0.24 |
| 26 | 2.52E-01 | 2.00E+00 | 1.25E-01 |  | 2.39 |  |
| 27 | 2.26E+00 | 1.92E+00 | 1.25E-01 |  | 1.66 |  |
| 28 | 2.29E+00 | 1.91E+00 | 1.25E-01 | 2.041 | 1.68 | 0.36 |
| 30 | 1.31E-01 | 2.00E+00 | 1.25E-01 | 2.114 | 2.45 | -0.34 |
| 31 | 2.25E-01 | 2.00E+00 | 1.25E-01 |  | 2.40 |  |
| 32 | 2.90E-01 | 2.00E+00 | 1.24E-01 | 2.071 | 2.37 | -0.30 |
| 33 | 1.21E+00 | 1.78E+00 | 1.24E-01 |  | 2.69 |  |
| 34 | 2.18E-01 | 2.00E+00 | 1.24E-01 |  | 2.40 |  |
| 35 | 1.88E-01 | 2.00E+00 | 1.24E-01 | 2.013 | 2.42 | -0.40 |
| 37 | 2.62E-01 | 2.00E+00 | 1.25E-01 |  | 2.38 |  |
| 38 | 1.66E-01 | 2.00E+00 | 1.25E-01 |  | 2.43 |  |
| 39 | 1.69E-01 | 2.00E+00 | 1.25E-01 |  | 2.43 |  |
| 102 | 1.87E+00 | 2.09E+00 | 9.02E-01 | 3.208 | 3.26 | -0.05 |
| 103 | 1.80E-01 | 1.96E+00 | 1.24E-01 | 2.925 | 2.57 | 0.36 |
| 104 | 0.00E+00 | 1.96E+00 | 1.24E-01 | 1.836 | 2.66 | -0.82 |
| 105 | 1.85E+00 | 2.09E+00 | 9.02E-01 | 2.056 | 3.27 | -1.21 |
| 106 | 1.20E-01 | 1.96E+00 | 1.24E-01 | 3.056 | 2.60 | 0.46 |
| 107 | 1.82E+00 | 2.09E+00 | 9.02E-01 | 3.444 | 3.28 | 0.16 |
| 109 | 1.95E+00 | 2.09E+00 | 9.01E-01 | 3.056 | 3.22 | -0.16 |
| 110 | 1.91E+00 | 2.02E+00 | 9.01E-01 | 3.959 | 3.49 | 0.47 |
| 111 | 5.30E-02 | 2.03E+00 | 1.65E-01 | 2.860 | 2.48 | 0.38 |
| 112 | 8.64E-01 | 1.61E+00 | 1.40E-01 | 2.994 | 3.52 | -0.52 |
| 113 | 0.00E+00 | 2.02E+00 | 1.62E-01 | 2.731 | 2.54 | 0.19 |
| 114 | 1.11E-01 | 2.02E+00 | 1.56E-01 | 2.886 | 2.47 | 0.42 |
| 115 | 1.75E+00 | 2.09E+00 | 9.02E-01 | 3.310 | 3.32 | -0.01 |
| 116 | 2.02E+00 | 1.79E+00 | 7.66E-01 | 4.097 | 3.91 | 0.19 |
| 118 | 2.57E+00 | 1.78E+00 | 7.66E-01 | 3.886 | 3.67 | 0.22 |
| 120 | 5.81E-02 | 1.96E+00 | 1.24E-01 | 3.252 | 2.63 | 0.63 |
| 122 | 2.03E-01 | 2.05E+00 | 8.99E-01 | 4.658 | 4.24 | 0.42 |
| 12# | 0.00E+00 | 2.09E+00 | 9.01E-01 | 2.182 | 4.20 | -2.02 |
| 8* | 2.19E-01 | 1.99E+00 | 1.24E-01 | 2.328 | 2.44 | -0.11 |
| 29* | 1.67E-01 | 2.00E+00 | 1.25E-01 | 2.174 | 2.43 | -0.26 |
| 36* | 0.00E+00 | 2.00E+00 | 1.25E-01 | 2.367 | 2.52 | -0.15 |
| 20* | 1.80E-01 | 1.99E+00 | 1.02E-01 | 2.456 | 2.40 | 0.06 |
| 21* | 1.73E-01 | 1.95E+00 | 1.18E-01 | 2.523 | 2.59 | -0.07 |
| 121* | 2.65E+00 | 1.78E+00 | 7.62E-01 | 3.745 | 3.62 | 0.13 |
| 25* | 2.45E-01 | 1.97E+00 | 1.17E-01 | 2.444 | 2.48 | -0.04 |
| 119* | 2.74E+00 | 1.78E+00 | 7.66E-01 | 3.745 | 3.58 | 0.17 |
| 117* | 1.93E+00 | 2.03E+00 | 9.02E-01 | 3.097 | 3.44 | -0.35 |

**Table S14: Descriptor, experimental and predicted pIC_50_ values and their residuals for test set 1 compounds in cell line based QSAR model against B16-F1**

| No. | MaPCH | ZXS/ZXR | Mi1ERN | Exp. | Pred. | Res. |
| --- | --- | --- | --- | --- | --- | --- |
| 193 | 5.52E-02 | 5.89E-01 | 3.48E-03 | 2.658 | 2.07 | 0.59 |
| 198 | 5.37E-02 | 7.19E-01 | 4.21E-05 |  | 1.97 |  |
| 197 | 5.37E-02 | 7.25E-01 | 4.21E-05 |  | 1.94 |  |
| 196 | 5.37E-02 | 7.15E-01 | 3.39E-05 |  | 1.99 |  |
| 195 | 5.37E-02 | 7.29E-01 | 4.26E-05 |  | 1.93 |  |
| 199 | 4.62E-02 | 6.66E-01 | -2.78E-03 |  | 3.16 |  |
| 165 | 5.37E-02 | 5.68E-01 | 5.18E-05 | 1.845 | 2.63 | -0.78 |
| 173 | 5.52E-02 | 6.32E-01 | 2.42E-03 | 2.658 | 1.98 | 0.68 |
| 175 | 5.52E-02 | 7.36E-01 | 4.00E-04 | 1.016 | 1.72 | -0.71 |
| 176 | 5.52E-02 | 7.01E-01 | 3.75E-03 | 0.966 | 1.55 | -0.59 |
| 177 | 5.64E-02 | 6.75E-01 | 2.06E-03 | 2.409 | 1.72 | 0.69 |
| 178 | 5.64E-02 | 5.41E-01 | 8.40E-05 | 2.252 | 2.49 | -0.24 |
| 179 | 5.63E-02 | 7.11E-01 | 1.36E-03 | 2.244 | 1.64 | 0.61 |
| 181 | 5.52E-02 | 5.76E-01 | 8.77E-04 | 2.678 | 2.37 | 0.31 |
| 182 | 5.52E-02 | 6.07E-01 | 8.57E-04 | 2.796 | 2.24 | 0.56 |
| 184 | 5.37E-02 | 6.49E-01 | 4.01E-03 | 1.996 | 1.89 | 0.10 |
| 185 | 5.52E-02 | 5.71E-01 | 4.82E-03 | 1.824 | 2.02 | -0.19 |
| 186 | 5.52E-02 | 6.40E-01 | 1.70E-03 | 1.378 | 2.02 | -0.64 |
| 187 | 4.68E-02 | 6.90E-01 | 2.64E-03 | 2.328 | 2.48 | -0.15 |
| 188 | 5.52E-02 | 5.78E-01 | 8.97E-04 | 1.925 | 2.36 | -0.44 |
| 189 | 6.39E-02 | 6.17E-01 | 1.65E-03 | 0.945 | 1.32 | -0.38 |
| 190 | 5.74E-02 | 6.11E-01 | 6.54E-03 | 1.030 | 1.48 | -0.45 |
| 191 | 5.84E-02 | 5.98E-01 | 6.88E-03 | 2.187 | 1.41 | 0.78 |
| 194 | 5.83E-02 | 6.81E-01 | 2.16E-02 |  | -0.36 |  |
| 229 | 5.80E-02 | 6.18E-01 | 1.38E-03 |  | 1.89 |  |
| 230 | 5.80E-02 | 6.62E-01 | 1.26E-03 |  | 1.71 |  |
| 231 | 5.83E-02 | 5.42E-01 | 2.50E-03 | 1.417 | 2.08 | -0.66 |
| 232 | 5.89E-02 | 6.08E-01 | 3.69E-03 | 1.517 | 1.62 | -0.11 |
| 233 | 5.91E-02 | 6.79E-01 | 1.16E-03 |  | 1.54 |  |
| 235 | 3.50E-02 | 7.01E-01 | 2.70E-03 |  | 3.51 |  |
| 236 | 3.50E-02 | 7.46E-01 | -1.57E-03 |  | 3.72 |  |
| 239 | 3.51E-02 | 6.92E-01 | -1.81E-03 |  | 3.97 |  |
| 234 | 3.52E-02 | 5.70E-01 | 5.99E-04 | 4.260 | 4.26 | 0.00 |
| 240 | 3.51E-02 | 6.23E-01 | 8.49E-04 | 3.456 | 4.02 | -0.56 |
| 241 | 3.54E-02 | 6.37E-01 | 2.24E-03 |  | 3.80 |  |
| 24 | 3.47E-02 | 8.62E-01 | 9.01E-03 | 1.731 | 2.23 | -0.50 |
| 243 | 3.57E-02 | 5.39E-01 | -2.77E-03 | 4.678 | 4.68 | 0.00 |
| 244 | 3.87E-02 | 6.61E-01 | -3.14E-03 | 4.569 | 3.90 | 0.66 |
| 245 | 3.67E-02 | 6.37E-01 | -2.89E-03 | 3.542 | 4.17 | -0.63 |
| 246 | 3.66E-02 | 5.29E-01 | 4.09E-03 | 4.367 | 3.98 | 0.39 |
| 247 | 3.66E-02 | 5.29E-01 | 4.09E-03 | 3.793 | 3.98 | -0.18 |
| 248 | 3.62E-02 | 6.39E-01 | 8.07E-04 | 4.252 | 3.85 | 0.40 |
| 249 | 3.59E-02 | 6.34E-01 | 5.37E-04 | 4.276 | 3.93 | 0.35 |
| 257 | 9.76E-02 | 7.80E-01 |  | |  |  |
| 258 | 3.46E-02 | 7.54E-01 |  | |  |  |
| 250 | 3.62E-02 | 6.50E-01 | 2.41E-03 | 4.036 | 3.65 | 0.39 |
| 251 | 3.60E-02 | 5.07E-01 | -2.64E-03 | 4.495 | 4.77 | -0.28 |
| 252 | 3.57E-02 | 6.68E-01 | -3.39E-03 | 4.155 | 4.17 | -0.02 |
| 253 | 3.63E-02 | 5.48E-01 | 4.02E-04 |  | 4.28 |  |
| 254 | 4.15E-02 | 6.94E-01 | 9.81E-04 | 2.638 | 3.11 | -0.47 |
| 255 | 3.73E-02 | 6.91E-01 | -3.76E-03 | 4.420 | 3.96 | 0.46 |
| 256 | 3.35E-02 | 6.97E-01 | 6.09E-04 |  | 3.87 |  |
| 259 | 9.14E-02 | 7.41E-01 |  | |  |  |
| 227 | 5.52E-02 | 6.66E-01 | 3.02E-03 | 2.658 | 1.78 | 0.88 |
| 228 | 5.67E-02 | 5.52E-01 | 1.43E-03 | 2.409 | 2.29 | 0.12 |
| 238# | 3.52E-02 | 7.31E-01 | 7.88E-03 | 1.226 | 2.86 | -1.64 |
| 161* | 5.37E-02 | 5.65E-01 | 4.29E-03 | 2.229 | 2.23 | 0.00 |
| 162* | 5.37E-02 | 5.31E-01 | 3.85E-03 | 2.328 | 2.42 | -0.09 |
| 192* | 5.37E-02 | 6.25E-01 | 4.08E-03 | 1.810 | 1.99 | -0.18 |
| 163* | 5.37E-02 | 5.56E-01 | 4.12E-03 | 2.495 | 2.29 | 0.21 |
| 166* | 5.37E-02 | 5.68E-01 | 5.18E-05 | 2.482 | 2.63 | -0.14 |
| 168* | 5.37E-02 | 5.90E-01 | 1.45E-04 | 2.367 | 2.52 | -0.15 |
| 167* | 5.37E-02 | 5.53E-01 | 3.77E-03 | 2.495 | 2.33 | 0.16 |
| 174* | 5.52E-02 | 6.66E-01 | 1.09E-03 | 1.724 | 1.96 | -0.24 |
| 180* | 5.89E-02 | 6.18E-01 | 7.49E-03 | 1.257 | 1.22 | 0.04 |
| 170* | 5.37E-02 | 6.33E-01 | 3.86E-04 | 2.638 | 2.31 | 0.33 |
| 172* | 5.56E-02 | 6.24E-01 | 9.83E-04 | 2.097 | 2.12 | -0.02 |
| 183* | 5.37E-02 | 6.04E-01 | 4.03E-04 | 2.222 | 2.43 | -0.21 |
| 169* | 5.37E-02 | 5.89E-01 | 4.23E-03 | 2.523 | 2.13 | 0.39 |

**Table S15: Descriptor, experimental and predicted pIC_50_ values and their residuals for test set 1 compounds in cell line based QSAR model against DU145**

| **No.** | **RNO** | **HS-1z** | **H-HC-2/ST** | **Exp.** | **Pred.** | **Res.** |
| --- | --- | --- | --- | --- | --- | --- |
| 102 | 5.56E-02 | 1.16E+01 | 2.30E-02 | 3.086 | 2.56 | 0.53 |
| 104 | 7.58E-02 | 1.83E+00 | 1.86E-02 | 1.764 | 2.88 | -1.12 |
| 107 | 5.88E-02 | 1.40E+01 | 2.25E-02 | 3.108 | 2.65 | 0.46 |
| 108 | 5.77E-02 | 0.00E+00 | 1.08E-02 | 1.901 | 2.79 | -0.89 |
| 109 | 6.00E-02 | 6.41E+01 | 2.20E-02 | 2.959 | 3.17 | -0.21 |
| 110 | 8.33E-02 | 4.95E+01 | 2.28E-02 | 3.959 | 3.36 | 0.60 |
| 111 | 1.11E-01 | 3.89E+01 | 2.14E-02 | 2.733 | 3.71 | -0.97 |
| 113 | 9.09E-02 | 6.09E-01 | 1.07E-02 | 2.572 | 3.30 | -0.73 |
| 114 | 9.09E-02 | 0.00E+00 | 1.12E-02 | 2.824 | 3.28 | -0.46 |
| 115 | 5.56E-02 | 1.40E+01 | 2.19E-02 | 3.456 | 2.61 | 0.84 |
| 116 | 8.00E-02 | 2.92E+01 | 2.65E-02 | 3.959 | 3.01 | 0.95 |
| 117 | 8.33E-02 | 4.78E+01 | 2.30E-02 | 2.971 | 3.34 | -0.37 |
| 118 | 7.55E-02 | 2.57E+01 | 2.83E-02 | 3.796 | 2.87 | 0.93 |
| 119 | 7.55E-02 | 2.27E+01 | 2.94E-02 | 3.585 | 2.81 | 0.78 |
| 120 | 7.25E-02 | 6.09E+00 | 1.73E-02 | 3.076 | 2.91 | 0.17 |
| 122 | 5.77E-02 | 3.04E+00 | 6.34E-03 | 3.620 | 2.94 | 0.69 |
| 121 | 7.55E-02 | 2.15E+01 | 2.84E-02 | 4.602 | 2.82 | 1.78 |
| 192 | 1.25E-02 | 4.70E+01 | 1.34E-02 | 1.967 | 2.51 | -0.54 |
| 161 | 4.35E-02 | 4.77E+01 | 1.31E-02 | 2.387 | 2.99 | -0.60 |
| 198 | 1.61E-02 | 4.58E+01 | 1.37E-02 | 2.658 | 2.54 | 0.12 |
| 196 | 1.79E-02 | 4.60E+01 | 1.47E-02 | 2.387 | 2.55 | -0.16 |
| 195 | 2.00E-02 | 4.65E+01 | 1.59E-02 | 1.762 | 2.55 | -0.79 |
| 199 | 0.00E+00 | 4.22E+01 | 1.06E-02 | 2.678 | 2.34 | 0.34 |
| 165 | 3.41E-02 | 4.83E+01 | 1.34E-02 | 2.260 | 2.85 | -0.59 |
| 166 | 3.41E-02 | 4.83E+01 | 1.34E-02 | 2.699 | 2.85 | -0.15 |
| 167 | 3.49E-02 | 5.05E+01 | 1.38E-02 | 2.824 | 2.87 | -0.05 |
| 168 | 2.56E-02 | 5.30E+01 | 1.23E-02 | 2.187 | 2.79 | -0.60 |
| 169 | 2.56E-02 | 4.98E+01 | 1.37E-02 | 2.959 | 2.72 | 0.24 |
| 172 | 2.47E-02 | 4.88E+01 | 1.34E-02 | 2.337 | 2.71 | -0.37 |
| 174 | 2.22E-02 | 5.15E+01 | 1.62E-02 | 2.409 | 2.63 | -0.22 |
| 175 | 3.51E-02 | 3.86E+01 | 2.49E-02 |  | 2.47 |  |
| 176 | 3.51E-02 | 5.00E+01 | 2.06E-02 |  | 2.69 |  |
| 177 | 3.70E-02 | 4.30E+01 | 2.56E-02 | 2.721 | 2.52 | 0.20 |
| 178 | 3.64E-02 | 5.16E+01 | 2.27E-02 | 3.046 | 2.67 | 0.37 |
| 179 | 3.77E-02 | 5.21E+01 | 2.49E-02 | 2.301 | 2.64 | -0.34 |
| 180 | 4.26E-02 | 1.00E+02 | 3.95E-02 |  | 2.82 |  |
| 182 | 2.27E-02 | 4.76E+01 | 2.29E-02 | 2.745 | 2.42 | 0.32 |
| 183 | 1.28E-02 | 4.78E+01 | 1.43E-02 | 2.745 | 2.50 | 0.25 |
| 184 | 1.28E-02 | 5.13E+01 | 1.39E-02 | 2.824 | 2.54 | 0.28 |
| 185 | 3.49E-02 | 4.68E+01 | 1.57E-02 | 1.827 | 2.78 | -0.96 |
| 186 | 3.75E-02 | 5.04E+01 | 1.50E-02 |  | 2.88 |  |
| 187 | 0.00E+00 | 5.40E+01 | 1.44E-02 | 2.509 | 2.36 | 0.15 |
| 189 | 3.66E-02 | 4.81E+01 | 2.05E-02 |  | 2.70 |  |
| 190 | 2.47E-02 | 5.20E+01 | 2.20E-02 | 3.155 | 2.52 | 0.64 |
| 191 | 2.50E-02 | 6.16E+01 | 3.29E-02 | 2.495 | 2.34 | 0.16 |
| 194 | 4.35E-02 | 5.41E+01 | 5.75E-02 |  | 1.92 |  |
| 229 | 8.33E-02 | 3.82E+01 | 1.48E-02 |  | 3.45 |  |
| 230 | 1.17E-01 | 3.76E+01 | 1.39E-02 |  | 3.98 |  |
| 231 | 8.70E-02 | 3.67E+01 | 1.87E-02 |  | 3.39 |  |
| 232 | 8.70E-02 | 4.99E+01 | 1.76E-02 |  | 3.55 |  |
| 233 | 9.09E-02 | 4.27E+01 | 2.15E-02 |  | 3.44 |  |
| 235 | 3.33E-02 | 4.40E+00 | 0.00E+00 |  | 2.74 |  |
| 236 | 5.88E-02 | 4.40E+00 | 7.09E-03 |  | 2.95 |  |
| 237 | 5.88E-02 | 4.40E+00 | 6.30E-03 |  | 2.97 |  |
| 238 | 5.88E-02 | 8.67E+01 | 1.68E-02 |  | 3.51 |  |
| 239 | 7.89E-02 | 3.77E+00 | 4.88E-03 |  | 3.30 |  |
| 234 | 9.52E-02 | 6.92E+00 | 5.93E-03 | 4.149 | 3.55 | 0.60 |
| 240 | 7.89E-02 | 8.92E+01 | 1.96E-02 | 3.373 | 3.77 | -0.39 |
| 241 | 3.33E-02 | 3.79E+01 | 0.00E+00 |  | 3.07 |  |
| 242 | 1.47E-02 | 3.68E+01 | 6.16E-03 |  | 2.63 |  |
| 243 | 8.51E-02 | 9.03E+01 | 1.62E-02 | 5.155 | 3.96 | 1.20 |
| 244 | 9.09E-02 | 9.66E+01 | 1.71E-02 | 3.943 | 4.08 | -0.14 |
| 245 | 9.09E-02 | 9.60E+01 | 1.75E-02 | 4.456 | 4.07 | 0.39 |
| 246 | 1.15E-01 | 8.87E+01 | 1.56E-02 | 4.921 | 4.41 | 0.51 |
| 247 | 1.15E-01 | 8.87E+01 | 1.56E-02 | 3.991 | 4.41 | -0.42 |
| 248 | 1.30E-01 | 9.41E+01 | 1.65E-02 | 4.022 | 4.66 | -0.64 |
| 249 | 8.89E-02 | 1.58E+02 | 2.37E-02 | 4.284 | 4.49 | -0.21 |
| 257 | 1.30E-01 | 2.45E+01 | 1.73E-02 |  | 3.96 |  |
| 258 | 1.22E-01 | 0.00E+00 | 0.00E+00 |  | 4.04 |  |
| 250 | 8.51E-02 | 9.63E+01 | 1.66E-02 | 4.301 | 4.01 | 0.30 |
| 251 | 9.09E-02 | 8.87E+01 | 1.65E-02 | 4.678 | 4.02 | 0.66 |
| 252 | 8.00E-02 | 9.60E+01 | 1.66E-02 | 4.509 | 3.93 | 0.58 |
| 253 | 9.30E-02 | 1.14E+02 | 2.39E-02 |  | 4.11 |  |
| 254 | 9.52E-02 | 1.24E+02 | 3.33E-02 | 2.551 | 4.00 | -1.45 |
| 256 | 1.18E-01 | 1.08E+02 | 1.94E-02 |  | 4.55 |  |
| 259 | 1.18E-01 | 8.72E+01 | 5.22E-02 |  | 3.50 |  |
| 227 | 2.47E-02 | 4.24E+01 | 2.04E-02 | 2.770 | 2.47 | 0.30 |
| 228 | 3.70E-02 | 4.51E+01 | 2.56E-02 | 2.721 | 2.55 | 0.18 |
| 261 | 6.25E-02 | 3.52E+01 | 3.58E-02 | 2.914 | 2.57 | 0.34 |
| 263 | 1.00E-01 | 8.52E+00 | 2.16E-02 | 3.678 | 3.24 | 0.44 |
| 264 | 8.77E-02 | 6.71E+01 | 2.30E-02 | 4.208 | 3.59 | 0.62 |
| 265 | 6.78E-02 | 6.65E+01 | 2.26E-02 | 4.268 | 3.30 | 0.97 |
| 266 | 9.80E-02 | 4.67E+01 | 2.40E-02 | 3.337 | 3.52 | -0.18 |
| 267 | 9.26E-02 | 1.55E+01 | 1.65E-02 | 2.377 | 3.32 | -0.95 |
| 268 | 1.19E-01 | 4.26E+00 | 1.29E-02 | 3.284 | 3.70 | -0.42 |
| 269 | 7.14E-02 | 2.27E+01 | 1.60E-02 | 2.253 | 3.09 | -0.84 |
| 270 | 9.84E-02 | 4.26E+00 | 1.22E-02 | 3.244 | 3.41 | -0.17 |
| 271 | 7.94E-02 | 3.65E+00 | 1.22E-02 | 3.721 | 3.12 | 0.60 |
| 273 | 7.41E-02 | 3.29E+01 | 4.05E-02 | 1.232 | 2.60 | -1.37 |
| 274 | 1.02E-01 | 7.92E+00 | 3.49E-02 | 2.213 | 2.92 | -0.71 |
| 275 | 1.96E-02 | 3.79E+01 | 2.48E-02 | 1.216 | 2.23 | -1.02 |
| 276 | 5.36E-02 | 2.57E+01 | 3.98E-02 |  | 2.24 |  |
| 277 | 7.27E-02 | 2.87E+01 | 2.29E-02 |  | 2.99 |  |
| 278 | 1.00E-01 | 4.87E+00 | 2.23E-02 | 2.004 | 3.18 | -1.18 |
| 279 | 5.26E-02 | 2.21E+01 | 2.22E-02 |  | 2.64 |  |
| 281 | 5.45E-02 | 2.33E+01 | 1.65E-02 |  | 2.83 |  |
| 282 | 8.33E-02 | 4.26E+00 | 1.21E-02 | 3.959 | 3.19 | 0.77 |
| 283 | 1.59E-02 | 2.64E+01 | 1.81E-02 | 2.099 | 2.24 | -0.14 |
| 284 | 4.41E-02 | 9.74E+00 | 2.33E-02 | 2.451 | 2.36 | 0.09 |
| 285 | 1.67E-02 | 2.82E+01 | 1.95E-02 | 1.996 | 2.23 | -0.23 |
| 287 | 5.56E-02 | 2.21E+01 | 1.62E-02 | 1.914 | 2.84 | -0.93 |
| 288 | 8.47E-02 | 4.87E+00 | 1.22E-02 | 3.854 | 3.21 | 0.64 |
| 289 | 3.92E-02 | 9.03E+01 | 2.34E-02 | 3.310 | 3.08 | 0.23 |
| 290 | 1.05E-01 | 1.10E+01 | 2.46E-02 | 3.420 | 3.26 | 0.16 |
| 291 | 9.52E-02 | 9.74E+00 | 2.35E-02 | 2.469 | 3.13 | -0.66 |
| 292 | 9.43E-02 | 2.34E+01 | 2.98E-02 | 3.721 | 3.09 | 0.63 |
| 293 | 7.27E-02 | 2.46E+01 | 2.85E-02 |  | 2.81 |  |
| 294 | 8.77E-02 | 4.18E+01 | 1.14E-02 |  | 3.64 |  |
| 295 | 9.43E-02 | 3.59E+01 | 2.98E-02 | 3.469 | 3.21 | 0.26 |
| 297 | 1.23E-01 | 2.09E+01 | 3.76E-02 |  | 3.30 |  |
| 297 | 1.15E-01 | 6.09E-01 | 2.09E-02 | 3.810 | 3.40 | 0.41 |
| 298 | 1.09E-01 | 2.39E+01 | 2.57E-02 |  | 3.42 |  |
| 301 | 5.88E-02 | 1.69E+01 | 2.28E-02 | 2.660 | 2.67 | -0.01 |
| 302 | 6.00E-02 | 4.21E+01 | 3.00E-02 |  | 2.75 |  |
| 304 | 7.14E-02 | 1.13E+01 | 2.23E-02 | 3.032 | 2.82 | 0.22 |
| 305 | 5.88E-02 | 1.63E+01 | 2.27E-02 | 2.690 | 2.66 | 0.03 |
| 306 | 7.14E-02 | 2.10E+01 | 3.72E-02 | 2.991 | 2.53 | 0.46 |
| 310 | 6.90E-02 | 4.91E+01 | 3.69E-02 | 2.991 | 2.78 | 0.21 |
| 311 | 5.36E-02 | 4.27E+01 | 2.81E-02 | 2.541 | 2.71 | -0.17 |
| 312 | 6.45E-02 | 1.69E+01 | 2.14E-02 | 3.119 | 2.79 | 0.33 |
| 314 | 5.88E-02 | 2.84E+01 | 2.43E-02 | 2.740 | 2.74 | 0.00 |
| 315 | 5.56E-02 | 2.47E+01 | 3.21E-02 | 1.290 | 2.46 | -1.17 |
| 316 | 8.16E-02 | 4.36E+01 | 4.46E-02 | 2.740 | 2.72 | 0.02 |
| 318 | 7.55E-02 | 3.58E+01 | 4.41E-02 |  | 2.56 |  |
| 319 | 5.17E-02 | 4.64E+01 | 3.26E-02 | 2.590 | 2.60 | -0.01 |
| 325 | 1.85E-02 | 2.42E+01 | 1.89E-02 | 2.580 | 2.23 | 0.35 |
| 327 | 1.72E-02 | 4.46E+01 | 2.06E-02 | 2.205 | 2.37 | -0.17 |
| 328 | 4.08E-02 | 3.70E+01 | 3.46E-02 | 1.914 | 2.29 | -0.38 |
| 331 | 1.96E-02 | 2.23E+01 | 1.92E-02 | 2.561 | 2.22 | 0.34 |
| 332 | 4.00E-02 | 3.89E+01 | 2.93E-02 | 2.780 | 2.43 | 0.35 |
| 333 | 4.35E-02 | 3.83E+01 | 2.83E-02 |  | 2.51 |  |
| 334 | 2.27E-02 | 4.02E+01 | 1.31E-02 | 3.319 | 2.60 | 0.72 |
| 335 | 1.82E-02 | 4.33E+01 | 2.07E-02 |  | 2.37 |  |
| 336 | 1.96E-02 | 2.60E+01 | 1.85E-02 | 1.631 | 2.28 | -0.65 |
| 339 | 1.96E-02 | 2.60E+01 | 1.75E-02 |  | 2.30 |  |
| 296# | 9.52E-02 | 3.42E+01 | 2.93E-02 | 5.000 | 3.22 | 1.78 |
| 103* | 7.58E-02 | 3.04E+00 | 1.57E-02 | 2.963 | 2.97 | -0.01 |
| 105* | 5.88E-02 | 1.64E+01 | 2.28E-02 | 2.018 | 2.66 | -0.65 |
| 106* | 7.58E-02 | 4.26E+00 | 1.84E-02 | 3.032 | 2.91 | 0.12 |
| 112* | 8.77E-02 | 0.00E+00 | 1.85E-02 | 2.991 | 3.05 | -0.06 |
| 162* | 4.44E-02 | 4.77E+01 | 1.36E-02 | 2.921 | 2.99 | -0.07 |
| 193* | 2.47E-02 | 5.28E+01 | 2.06E-02 | 2.770 | 2.56 | 0.21 |
| 163* | 4.44E-02 | 4.77E+01 | 1.35E-02 | 3.046 | 2.99 | 0.05 |
| 197* | 1.79E-02 | 4.72E+01 | 1.47E-02 | 2.553 | 2.56 | 0.00 |
| 170* | 2.56E-02 | 4.77E+01 | 1.43E-02 | 2.658 | 2.69 | -0.03 |
| 173* | 2.47E-02 | 5.05E+01 | 2.03E-02 | 2.638 | 2.55 | 0.09 |
| 181* | 2.53E-02 | 4.82E+01 | 2.31E-02 | 2.620 | 2.46 | 0.16 |
| 188* | 1.22E-02 | 5.64E+01 | 2.06E-02 | 2.328 | 2.41 | -0.08 |
| 255* | 9.76E-02 | 1.42E+02 | 2.48E-02 | 4.658 | 4.43 | 0.22 |
| 262* | 1.21E-01 | 9.13E+00 | 2.28E-02 | 3.469 | 3.53 | -0.06 |
| 272* | 4.08E-02 | 4.21E+01 | 2.58E-02 | 2.578 | 2.57 | 0.01 |
| 280* | 8.06E-02 | 4.26E+00 | 2.37E-02 | 3.076 | 2.85 | 0.23 |
| 286* | 4.62E-02 | 7.31E+00 | 2.40E-02 | 2.516 | 2.35 | 0.16 |
| 340* | 4.00E-02 | 3.82E+01 | 2.88E-02 | 2.400 | 2.44 | -0.04 |

**Table S16: Descriptor, experimental and predicted pIC_50_ values and their residuals for test set 1 compounds in cell line based QSAR model against Fibroblast**

| **No.** | **MiVN** | **L1E** | **THCMD** | **Exp.** | **Pred.** | **Res.** |
| --- | --- | --- | --- | --- | --- | --- |
| 192* | 2.98E+00 | 7.73E-02 | -1.41E+00 | 1.526 | 1.88 | -0.35 |
| 161* | 2.99E+00 | 3.01E-01 | -1.99E+00 | 2.328 | 2.41 | -0.08 |
| 162 | 2.99E+00 | 3.02E-01 | -2.00E+00 | 2.319 | 2.42 | -0.10 |
| 163* | 2.99E+00 | 3.04E-01 | -1.91E+00 | 2.398 | 2.39 | 0.01 |
| 165 | 2.98E+00 | 9.69E-02 | -6.70E-01 | 1.690 | 1.69 | 0.00 |
| 168 | 2.99E+00 | 3.52E-01 | -1.44E+00 | 2.143 | 2.30 | -0.16 |
| 169 | 2.98E+00 | 1.51E-01 | -1.56E+00 | 2.602 | 1.98 | 0.63 |
| 170 | 2.98E+00 | 2.10E-01 | -2.09E+00 | 2.092 | 2.16 | -0.07 |
| 173* | 2.98E+00 | -2.66E-01 | -2.41E+00 | 2.201 | 1.88 | 0.33 |
| 174 | 2.98E+00 | -3.01E-01 | -1.32E+00 |  | 1.56 |  |
| 175 | 2.99E+00 | -4.03E-01 | -1.06E+00 |  | 1.61 |  |
| 176 | 2.98E+00 | -2.54E-01 | -1.09E+00 |  | 1.53 |  |
| 177* | 2.98E+00 | -8.68E-01 | -2.99E+00 | 1.539 | 1.56 | -0.02 |
| 178 | 3.00E+00 | -8.13E-01 | -2.76E+00 | 1.870 | 1.95 | -0.08 |
| 179* | 3.00E+00 | -8.17E-01 | -7.80E-01 | 1.476 | 1.41 | 0.06 |
| 180 | 2.98E+00 | -4.08E-01 | -2.49E+00 |  | 1.79 |  |
| 181* | 2.98E+00 | -1.28E-01 | -2.32E+00 | 1.854 | 1.96 | -0.11 |
| 182 | 2.98E+00 | -1.26E-01 | -2.34E+00 | 2.155 | 1.97 | 0.19 |
| 183 | 2.98E+00 | 7.35E-02 | -1.42E+00 | 1.936 | 1.88 | 0.06 |
| 184 | 2.98E+00 | 7.54E-02 | -1.50E+00 | 1.907 | 1.90 | 0.01 |
| 185 | 2.98E+00 | -6.08E-01 | -1.45E+00 | 1.239 | 1.35 | -0.11 |
| 186 | 2.98E+00 | -4.34E-01 | -1.41E+00 |  | 1.48 |  |
| 187 | 2.96E+00 | 1.40E-01 | -2.09E-01 | 1.222 | 1.20 | 0.03 |
| 188 | 2.96E+00 | -2.36E-01 | -1.84E+00 | 1.073 | 1.34 | -0.27 |
| 189 | 2.93E+00 | -2.67E-01 | -8.14E-01 |  | 0.43 |  |
| 191 | 2.96E+00 | -3.23E-01 | -1.16E+00 | 1.143 | 1.09 | 0.06 |
| 194 | 3.04E+00 | 5.25E-01 | -1.81E+00 |  | 3.56 |  |

**Table S17: Descriptor, experimental and predicted pIC_50_ values and their residuals for test set 1 compounds in cell line based QSAR model against HCT-15**

| **No.** | **AVN** | **MiNACN** | **MaVC** | **Exp.** | **Pred.** | **Res.** |
| --- | --- | --- | --- | --- | --- | --- |
| 1 | 3.50E+00 | -5.14E-01 | 3.99E+00 | 2.585 | 2.59 | 0.00 |
| 5 | 3.26E+00 | -6.22E-01 | 3.99E+00 | 2.367 | 2.24 | 0.12 |
| 6 | 3.25E+00 | -6.40E-01 | 3.99E+00 |  | 2.26 |  |
| 7 | 3.25E+00 | -6.33E-01 | 3.99E+00 |  | 2.24 |  |
| 8 | 3.26E+00 | -6.21E-01 | 4.01E+00 | 2.260 | 2.44 | -0.18 |
| 9 | 3.25E+00 | -6.33E-01 | 3.99E+00 |  | 2.24 |  |
| 10 | 3.26E+00 | -6.22E-01 | 4.00E+00 | 2.553 | 2.34 | 0.21 |
| 11 | 3.26E+00 | -6.10E-01 | 4.06E+00 |  | 2.92 |  |
| 12 | 3.27E+00 | -6.07E-01 | 3.99E+00 |  | 2.24 |  |
| 13 | 3.25E+00 | -6.29E-01 | 3.99E+00 | 2.076 | 2.24 | -0.16 |
| 15 | 3.23E+00 | -6.90E-01 | 3.99E+00 | 2.347 | 2.31 | 0.04 |
| 16 | 3.23E+00 | -7.01E-01 | 4.06E+00 | 3.046 | 3.03 | 0.02 |
| 17 | 3.23E+00 | -7.06E-01 | 3.99E+00 | 2.469 | 2.34 | 0.13 |
| 18 | 3.22E+00 | -7.26E-01 | 3.99E+00 | 2.357 | 2.36 | 0.00 |
| 19 | 3.22E+00 | -6.59E-01 | 4.01E+00 | 2.509 | 2.42 | 0.08 |
| 21 | 3.20E+00 | -6.90E-01 | 3.99E+00 | 2.252 | 2.24 | 0.01 |
| 22 | 3.23E+00 | -6.58E-01 | 3.99E+00 | 2.131 | 2.25 | -0.12 |
| 23 | 3.22E+00 | -6.60E-01 | 3.99E+00 | 2.114 | 2.23 | -0.11 |
| 24 | 3.22E+00 | -6.59E-01 | 3.99E+00 | 2.237 | 2.23 | 0.01 |
| 25 | 3.24E+00 | -6.57E-01 | 3.99E+00 | 2.337 | 2.27 | 0.07 |
| 26 | 3.21E+00 | -5.13E-01 | 4.00E+00 | 2.046 | 2.01 | 0.04 |
| 27 | 3.22E+00 | -5.13E-01 | 4.00E+00 |  | 2.03 |  |
| 28 | 3.22E+00 | -5.13E-01 | 4.00E+00 |  | 2.03 |  |
| 29 | 3.21E+00 | -5.13E-01 | 4.00E+00 |  | 2.01 |  |
| 30 | 3.18E+00 | -5.13E-01 | 4.00E+00 | 2.004 | 1.94 | 0.07 |
| 31 | 3.41E+00 | -5.13E-01 | 4.00E+00 |  | 2.47 |  |
| 32 | 3.22E+00 | -6.13E-01 | 4.00E+00 | 2.086 | 2.23 | -0.15 |
| 33 | 3.21E+00 | -6.61E-01 | 3.99E+00 |  | 2.21 |  |
| 34 | 3.40E+00 | -6.25E-01 | 4.00E+00 |  | 2.68 |  |
| 35 | 3.22E+00 | -6.28E-01 | 4.00E+00 | 2.174 | 2.26 | -0.09 |
| 36 | 3.24E+00 | -6.30E-01 | 3.99E+00 | 2.229 | 2.21 | 0.02 |
| 37 | 3.24E+00 | -6.25E-01 | 4.00E+00 |  | 2.30 |  |
| 38 | 3.24E+00 | -6.30E-01 | 4.01E+00 |  | 2.41 |  |
| 39 | 3.20E+00 | -6.28E-01 | 4.00E+00 |  | 2.22 |  |
| 2* | 3.16E+00 | -8.20E-01 | 3.99E+00 | 2.284 | 2.41 | -0.13 |
| 4* | 3.26E+00 | -6.17E-01 | 3.99E+00 | 2.108 | 2.23 | -0.13 |
| 3* | 3.26E+00 | -5.13E-01 | 3.99E+00 | 2.037 | 2.02 | 0.01 |
| 14* | 3.25E+00 | -6.43E-01 | 3.99E+00 | 2.143 | 2.26 | -0.12 |
| 20* | 3.00E+00 | -6.53E-01 | 4.03E+00 | 2.013 | 2.10 | -0.08 |

**Table S18: Descriptor, experimental and predicted pIC_50_ values and their residuals for test set 1 compounds in cell line based QSAR model against HOP-62**

| **No.** | **ZXS/ZXR** | **RPCSQ** | **NF** | **EXP** | **PRED** | **RES** |
| --- | --- | --- | --- | --- | --- | --- |
| 296 | 5.61E-01 | 1.63E-01 | 0.00E+00 | 5.000 | 4.76 | 0.24 |
| 297 | 6.05E-01 | 1.61E-01 | 0.00E+00 | 4.699 | 4.24 | 0.46 |
| 298 | 6.42E-01 | 2.13E-01 | 0.00E+00 | 2.750 | 3.56 | -0.81 |
| 302 | 6.47E-01 | 3.48E-01 | 0.00E+00 | 2.480 | 2.90 | -0.42 |
| 303 | 6.39E-01 | 4.44E-01 | 0.00E+00 | 1.710 | 2.57 | -0.86 |
| 306 | 6.44E-01 | 3.48E-01 | 0.00E+00 | 2.245 | 2.93 | -0.69 |
| 307 | 6.74E-01 | 3.07E-01 | 0.00E+00 | 2.996 | 2.75 | 0.24 |
| 311 | 6.91E-01 | 2.92E-01 | 0.00E+00 | 2.996 | 2.62 | 0.38 |
| 315 | 7.03E-01 | 3.96E-01 | 0.00E+00 | 1.770 | 2.01 | -0.24 |
| 313 | 6.64E-01 | 2.88E-01 | 0.00E+00 | 3.268 | 2.96 | 0.31 |
| 316 | 6.55E-01 | 3.83E-01 | 0.00E+00 | 3.181 | 2.65 | 0.54 |
| 317 | 6.81E-01 | 4.10E-01 | 0.00E+00 |  | 2.21 |  |
| 318 | 6.42E-01 | 3.61E-01 | 0.00E+00 | 3.319 | 2.90 | 0.42 |
| 319 | 6.37E-01 | 3.81E-01 | 0.00E+00 | 2.300 | 2.87 | -0.57 |
| 326 | 6.58E-01 | 3.85E-01 | 1.00E+00 | 1.697 | 1.78 | -0.08 |
| 327 | 6.74E-01 | 3.81E-01 | 1.00E+00 |  | 1.61 |  |
| 328 | 6.93E-01 | 3.01E-01 | 1.00E+00 | 1.435 | 1.73 | -0.30 |
| 329 | 6.92E-01 | 3.40E-01 | 1.00E+00 | 1.951 | 1.57 | 0.38 |
| 334 | 7.05E-01 | 3.90E-01 | 0.00E+00 | 2.140 | 2.01 | 0.13 |
| 333 | 6.28E-01 | 4.34E-01 | 0.00E+00 | 2.979 | 2.74 | 0.24 |
| 342 | 6.96E-01 | 2.71E-01 | 0.00E+00 | 2.600 | 2.65 | -0.05 |
| 335 | 5.74E-01 | 5.37E-01 | 0.00E+00 | 3.337 | 2.94 | 0.40 |
| 337 | 6.40E-01 | 4.35E-01 | 0.00E+00 | 1.742 | 2.59 | -0.85 |
| 340 | 6.87E-01 | 3.63E-01 | 0.00E+00 | 2.731 | 2.35 | 0.38 |
| 341 | 6.60E-01 | 5.05E-01 | 0.00E+00 | 2.780 | 2.04 | 0.74 |
| 312* | 6.25E-01 | 3.20E-01 | 0.00E+00 | 3.244 | 3.29 | -0.04 |
| 332* | 6.51E-01 | 4.38E-01 | 0.00E+00 | 2.640 | 2.45 | 0.19 |
| 336* | 6.67E-01 | 4.06E-01 | 0.00E+00 | 2.699 | 2.40 | 0.30 |
| 314* | 6.44E-01 | 3.67E-01 | 0.00E+00 | 2.670 | 2.85 | -0.18 |
| 305* | 6.50E-01 | 3.91E-01 | 0.00E+00 | 2.420 | 2.67 | -0.25 |

**Table S19: Descriptor, experimental and predicted pIC_50_ values and their residuals for test set 1 compounds in cell line based QSAR model against HCT-116**

| **No.** | **ZXS/ZXR** | **TPCCMD** | **RNCSQ** | **Exp.** | **Pred.** | **Res.** |
| --- | --- | --- | --- | --- | --- | --- |
| 296 | 5.61E-01 | 1.03E+01 | 2.09E+00 | 4.523 | 4.39 | 0.14 |
| 297 | 6.05E-01 | 9.57E+00 | 1.69E+00 | 4.000 | 3.97 | 0.03 |
| 300 | 7.96E-01 | 4.30E+00 | 9.09E+00 | 1.242 | 1.24 | 0.00 |
| 301 | 7.44E-01 | 6.51E+00 | 6.95E+00 |  | 2.11 |  |
| 305 | 6.50E-01 | 7.25E+00 | 5.34E+00 | 2.876 | 3.04 | -0.16 |
| 306 | 6.44E-01 | 5.54E+00 | 5.64E+00 | 2.580 | 2.79 | -0.21 |
| 307 | 6.74E-01 | 6.80E+00 | 7.70E-01 | 3.086 | 3.08 | 0.01 |
| 308 | 6.62E-01 | 7.99E+00 | 7.13E-01 |  | 3.36 |  |
| 311 | 6.91E-01 | 6.94E+00 | 2.84E+00 | 3.086 | 2.84 | 0.25 |
| 312 | 6.25E-01 | 4.05E+00 | 5.45E+00 | 2.710 | 2.71 | 0.00 |
| 314 | 6.44E-01 | 5.37E+00 | 5.02E+00 |  | 2.80 |  |
| 315 | 7.03E-01 | 7.53E+00 | 1.97E+00 | 2.710 | 2.90 | -0.19 |
| 316 | 6.55E-01 | 3.56E+00 | 1.19E+00 | 2.839 | 2.68 | 0.16 |
| 317 | 6.81E-01 | 5.08E+00 | 3.26E+00 | 3.022 | 2.59 | 0.43 |
| 318 | 6.42E-01 | 3.59E+00 | 5.92E+00 |  | 2.48 |  |
| 326 | 6.58E-01 | 2.51E+00 | 9.26E-01 | 2.745 | 2.51 | 0.24 |
| 327 | 6.74E-01 | 5.16E+00 | 7.97E-01 |  | 2.82 |  |
| 328 | 6.93E-01 | 4.59E+00 | 6.56E-01 | 2.516 | 2.59 | -0.08 |
| 329 | 6.92E-01 | 1.47E+00 | 2.70E+00 | 1.735 | 1.98 | -0.24 |
| 333 | 6.28E-01 | 3.38E+00 | 2.97E+00 | 2.979 | 2.74 | 0.24 |
| 334 | 7.05E-01 | 6.33E+00 | 3.03E+00 | 2.670 | 2.63 | 0.05 |
| 335 | 5.74E-01 | 7.04E+00 | 6.57E+00 | 3.398 | 3.49 | -0.09 |
| 336 | 6.67E-01 | 4.63E+00 | 1.20E-01 | 2.740 | 2.83 | -0.09 |
| 337 | 6.40E-01 | 2.00E+00 | 1.41E+00 | 1.870 | 2.53 | -0.66 |
| 340 | 6.87E-01 | 3.54E+00 | 1.35E+00 | 2.851 | 2.43 | 0.42 |
| 341 | 6.60E-01 | 4.36E+00 | 2.97E+00 | 2.810 | 2.66 | 0.15 |
| 342 | 6.96E-01 | 8.36E+00 | 1.80E-01 | 2.801 | 3.19 | -0.39 |
| 298* | 6.42E-01 | 4.12E+00 | 3.05E+00 | 2.939 | 2.75 | 0.19 |
| 302* | 6.47E-01 | 7.18E+00 | 5.79E+00 | 2.932 | 3.02 | -0.09 |
| 313* | 6.64E-01 | 6.05E+00 | 4.73E+00 | 2.848 | 2.78 | 0.07 |
| 314* | 6.37E-01 | 4.57E+00 | 2.37E+00 | 3.071 | 2.90 | 0.17 |
| 320* | 6.96E-01 | 8.28E+00 | 2.65E-01 | 2.879 | 3.18 | -0.30 |
| 332# | 6.51E-01 | 2.75E+00 | 1.12E+00 | 3.824 | 2.59 | 1.24 |

**Table S20: Descriptor, experimental and predicted pIC_50_ values and their residuals for test set 1 compounds in cell line based QSAR model against HeLA**

|  | **NN** | **W-1wP** | **MiVC** | **Exp.** | **Pred.** | **Res.** |
| --- | --- | --- | --- | --- | --- | --- |
| 42 | 2.00E+00 | 6.01E+01 | 3.83E+00 |  | 1.74 |  |
| 43 | 2.00E+00 | 6.27E+01 | 3.83E+00 |  | 1.74 |  |
| 44 | 2.00E+00 | 1.32E+02 | 3.72E+00 |  | 1.80 |  |
| 46 | 2.00E+00 | 1.28E+02 | 3.72E+00 |  | 1.79 |  |
| 48 | 3.00E+00 | 1.26E+02 | 3.83E+00 | 1.840 | 2.18 | -0.34 |
| 49 | 3.00E+00 | 1.29E+02 | 3.83E+00 | 2.280 | 2.18 | 0.10 |
| 50 | 3.00E+00 | 1.33E+02 | 3.83E+00 | 2.240 | 2.19 | 0.05 |
| 51 | 3.00E+00 | 1.24E+02 | 3.83E+00 | 2.440 | 2.17 | 0.27 |
| 52 | 3.00E+00 | 1.32E+02 | 3.83E+00 | 2.800 | 2.19 | 0.61 |
| 53 | 4.00E+00 | 1.24E+02 | 3.83E+00 |  | 2.47 |  |
| 55 | 4.00E+00 | 1.26E+02 | 3.75E+00 |  | 2.41 |  |
| 56 | 4.00E+00 | 1.36E+02 | 3.75E+00 | 1.950 | 2.43 | -0.48 |
| 57 | 3.00E+00 | 1.34E+02 | 3.83E+00 | 2.130 | 2.19 | -0.06 |
| 58 | 3.00E+00 | 1.39E+02 | 3.83E+00 | 1.950 | 2.20 | -0.25 |
| 59 | 3.00E+00 | 1.27E+02 | 3.80E+00 | 2.330 | 2.15 | 0.18 |
| 60 | 3.00E+00 | 1.19E+02 | 3.80E+00 | 2.600 | 2.14 | 0.47 |
| 61 | 2.00E+00 | 1.64E+02 | 3.83E+00 |  | 1.96 |  |
| 62 | 2.00E+00 | 1.71E+02 | 3.83E+00 |  | 1.97 |  |
| 63 | 2.00E+00 | 1.45E+02 | 3.76E+00 | 1.690 | 1.86 | -0.17 |
| 65 | 2.00E+00 | 1.25E+02 | 3.72E+00 |  | 1.78 |  |
| 66 | 2.00E+00 | 1.30E+02 | 3.73E+00 | 1.660 | 1.80 | -0.14 |
| 67 | 2.00E+00 | 1.70E+02 | 3.77E+00 | 1.650 | 1.92 | -0.27 |
| 68 | 2.00E+00 | 1.69E+02 | 3.81E+00 |  | 1.95 |  |
| 69 | 2.00E+00 | 1.39E+02 | 3.72E+00 | 1.840 | 1.81 | 0.03 |
| 70 | 2.00E+00 | 1.43E+02 | 3.83E+00 |  | 1.91 |  |
| 71 | 2.00E+00 | 1.28E+02 | 3.73E+00 | 1.970 | 1.80 | 0.17 |
| 72 | 2.00E+00 | 1.40E+02 | 3.83E+00 | 1.880 | 1.91 | -0.03 |
| 74 | 2.00E+00 | 1.39E+02 | 3.83E+00 | 1.730 | 1.90 | -0.17 |
| 123 | 0.00E+00 | 6.68E+01 | 3.82E+00 | 1.220 | 1.14 | 0.08 |
| 125 | 0.00E+00 | 8.28E+01 | 3.80E+00 | 1.190 | 1.16 | 0.03 |
| 126 | 0.00E+00 | 6.73E+01 | 3.69E+00 | 1.190 | 1.03 | 0.16 |
| 128 | 0.00E+00 | 7.55E+01 | 3.76E+00 | 1.190 | 1.11 | 0.08 |
| 129 | 0.00E+00 | 9.20E+01 | 3.82E+00 | 1.140 | 1.20 | -0.06 |
| 131 | 0.00E+00 | 1.06E+02 | 3.82E+00 | 1.330 | 1.23 | 0.10 |
| 132 | 0.00E+00 | 1.85E+02 | 3.76E+00 | 1.510 | 1.35 | 0.17 |
| 134 | 0.00E+00 | 2.87E+02 | 3.76E+00 | 1.920 | 1.56 | 0.36 |
| 136 | 0.00E+00 | 2.17E+02 | 3.76E+00 | 1.600 | 1.41 | 0.19 |
| 137 | 0.00E+00 | 2.00E+02 | 3.72E+00 | 1.140 | 1.34 | -0.20 |
| 140 | 0.00E+00 | 1.30E+02 | 3.82E+00 | 1.240 | 1.28 | -0.04 |
| 139 | 0.00E+00 | 1.76E+02 | 3.76E+00 | 1.170 | 1.33 | -0.16 |
| 142 | 0.00E+00 | 1.35E+02 | 3.67E+00 | 1.140 | 1.16 | -0.02 |
| 143 | 0.00E+00 | 1.65E+02 | 3.82E+00 | 1.110 | 1.35 | -0.24 |
| 144 | 0.00E+00 | 1.74E+02 | 3.82E+00 | 1.360 | 1.37 | -0.01 |
| 145 | 0.00E+00 | 7.03E+01 | 3.82E+00 | 1.170 | 1.15 | 0.02 |
| 146 | 0.00E+00 | 6.74E+01 | 3.73E+00 | 1.170 | 1.07 | 0.10 |
| 147 | 0.00E+00 | 7.40E+01 | 3.79E+00 | 1.120 | 1.13 | -0.01 |
| 149 | 0.00E+00 | 8.60E+01 | 3.79E+00 | 1.150 | 1.16 | -0.01 |
| 150 | 0.00E+00 | 9.28E+01 | 3.78E+00 | 1.140 | 1.17 | -0.03 |
| 151 | 0.00E+00 | 1.11E+02 | 3.78E+00 | 1.140 | 1.20 | -0.06 |
| 153 | 0.00E+00 | 1.18E+02 | 3.82E+00 | 1.130 | 1.25 | -0.12 |
| 154 | 0.00E+00 | 1.56E+02 | 3.82E+00 | 1.120 | 1.33 | -0.21 |
| 157 | 0.00E+00 | 8.99E+01 | 3.82E+00 | 1.170 | 1.19 | -0.02 |
| 158 | 0.00E+00 | 5.62E+01 | 3.83E+00 | 1.120 | 1.13 | -0.01 |
| 159 | 0.00E+00 | 7.74E+01 | 3.80E+00 | 1.130 | 1.15 | -0.02 |
| 160 | 0.00E+00 | 5.98E+01 | 3.83E+00 | 1.130 | 1.14 | -0.01 |
| 45# | 2.00E+00 | 1.41E+02 | 3.83E+00 | 2.620 | 1.91 | 0.71 |
| 40* | 2.00E+00 | 1.30E+02 | 3.83E+00 | 2.060 | 1.89 | 0.18 |
| 41* | 2.00E+00 | 1.32E+02 | 3.72E+00 | 1.870 | 1.80 | 0.07 |
| 47* | 2.00E+00 | 1.73E+02 | 3.65E+00 | 1.970 | 1.82 | 0.15 |
| 54* | 4.00E+00 | 1.23E+02 | 3.83E+00 | 2.240 | 2.47 | -0.23 |
| 64* | 2.00E+00 | 1.34E+02 | 3.76E+00 | 1.850 | 1.83 | 0.02 |
| 73* | 2.00E+00 | 1.24E+02 | 3.79E+00 | 1.730 | 1.84 | -0.11 |
| 124* | 0.00E+00 | 9.18E+01 | 3.69E+00 | 1.150 | 1.09 | 0.06 |
| 127* | 0.00E+00 | 7.27E+01 | 3.76E+00 | 1.150 | 1.11 | 0.05 |
| 130* | 0.00E+00 | 9.60E+01 | 3.82E+00 | 1.170 | 1.21 | -0.04 |
| 135* | 0.00E+00 | 1.84E+02 | 3.67E+00 | 1.220 | 1.27 | -0.05 |
| 141* | 0.00E+00 | 1.76E+02 | 3.82E+00 | 1.150 | 1.38 | -0.23 |
| 148* | 0.00E+00 | 8.01E+01 | 3.79E+00 | 1.130 | 1.15 | -0.02 |
| 152* | 0.00E+00 | 9.10E+01 | 3.80E+00 | 1.120 | 1.18 | -0.06 |
| 155* | 0.00E+00 | 1.90E+02 | 3.82E+00 | 1.210 | 1.41 | -0.20 |
| 156* | 0.00E+00 | 1.01E+02 | 3.82E+00 | 1.190 | 1.22 | -0.03 |
| 138* | 0.00E+00 | 1.62E+02 | 3.71E+00 | 1.490 | 1.25 | 0.24 |

**Table S21: Descriptor, experimental and predicted pIC_50_ values and their residuals for test set 1 compounds in cell line based QSAR model against HL-60**

| **No.** | **RNH** | **RNO** | **MaVO** | **Exp.** | **Pred.** | **Res.** |
| --- | --- | --- | --- | --- | --- | --- |
| 123 | 4.87E-01 | 1.08E-01 | 2.16E+00 | 1.770 | 1.67 | 0.10 |
| 124 | 4.90E-01 | 1.43E-01 | 2.16E+00 | 1.770 | 1.56 | 0.21 |
| 125 | 4.88E-01 | 1.22E-01 | 2.16E+00 | 1.800 | 1.63 | 0.17 |
| 126 | 4.88E-01 | 1.22E-01 | 2.16E+00 | 1.310 | 1.63 | -0.32 |
| 127 | 5.00E-01 | 1.14E-01 | 2.16E+00 | 1.390 | 1.56 | -0.17 |
| 129 | 5.11E-01 | 8.51E-02 | 2.16E+00 | 1.350 | 1.55 | -0.20 |
| 130 | 5.20E-01 | 8.00E-02 | 2.16E+00 | 1.380 | 1.50 | -0.12 |
| 131 | 5.36E-01 | 7.14E-02 | 2.16E+00 | 1.620 | 1.40 | 0.22 |
| 132 | 4.52E-01 | 8.06E-02 | 2.16E+00 | 2.220 | 2.01 | 0.21 |
| 134 | 4.19E-01 | 8.06E-02 | 2.16E+00 | 2.520 | 2.26 | 0.26 |
| 135 | 4.12E-01 | 1.32E-01 | 2.16E+00 | 2.300 | 2.18 | 0.12 |
| 136 | 4.48E-01 | 7.46E-02 | 2.16E+00 | 2.150 | 2.06 | 0.09 |
| 137 | 4.57E-01 | 1.00E-01 | 2.16E+00 | 1.340 | 1.92 | -0.58 |
| 138 | 4.62E-01 | 1.15E-01 | 2.16E+00 | 2.220 | 1.84 | 0.38 |
| 139 | 4.52E-01 | 1.13E-01 | 2.26E+00 | 2.300 | 2.29 | 0.01 |
| 140 | 4.71E-01 | 9.80E-02 | 2.16E+00 | 2.050 | 1.82 | 0.23 |
| 143 | 4.73E-01 | 9.09E-02 | 2.15E+00 | 1.340 | 1.79 | -0.45 |
| 145 | 5.00E-01 | 1.25E-01 | 2.16E+00 | 1.300 | 1.53 | -0.23 |
| 147 | 5.22E-01 | 1.09E-01 | 2.16E+00 | 1.220 | 1.41 | -0.19 |
| 148 | 5.31E-01 | 1.02E-01 | 2.16E+00 | 1.740 | 1.36 | 0.38 |
| 149 | 5.39E-01 | 9.62E-02 | 2.16E+00 | 1.380 | 1.31 | 0.07 |
| 150 | 5.11E-01 | 1.28E-01 | 2.16E+00 | 1.360 | 1.44 | -0.08 |
| 151 | 5.26E-01 | 1.40E-01 | 2.15E+00 | 1.260 | 1.26 | 0.00 |
| 154 | 4.36E-01 | 1.03E-01 | 2.17E+00 | 1.890 | 2.11 | -0.22 |
| 155 | 4.39E-01 | 9.76E-02 | 2.20E+00 | 2.100 | 2.21 | -0.11 |
| 156 | 4.75E-01 | 1.25E-01 | 2.18E+00 | 1.920 | 1.79 | 0.13 |
| 157 | 4.74E-01 | 1.32E-01 | 2.16E+00 | 1.740 | 1.71 | 0.03 |
| 158 | 5.37E-01 | 9.76E-02 | 2.14E+00 | 1.320 | 1.25 | 0.07 |
| 159 | 5.22E-01 | 1.09E-01 | 2.14E+00 | 1.320 | 1.33 | -0.01 |
| 153* | 4.62E-01 | 1.03E-01 | 2.18E+00 | 1.920 | 1.95 | -0.03 |
| 128* | 5.11E-01 | 1.06E-01 | 2.16E+00 | 1.420 | 1.50 | -0.08 |
| 141* | 4.51E-01 | 9.80E-02 | 2.16E+00 | 1.770 | 1.97 | -0.20 |
| 144* | 4.51E-01 | 9.80E-02 | 2.16E+00 | 2.050 | 1.97 | 0.08 |
| 146* | 5.12E-01 | 1.16E-01 | 2.16E+00 | 1.490 | 1.46 | 0.03 |
| 152* | 5.11E-01 | 1.28E-01 | 2.15E+00 | 1.360 | 1.40 | -0.04 |
| 160* | 5.00E-01 | 8.33E-02 | 2.16E+00 | 1.800 | 1.64 | 0.16 |
| 142* | 4.44E-01 | 1.30E-01 | 2.16E+00 | 1.740 | 1.94 | -0.20 |

**Table S22: Descriptor, experimental and predicted pIC_50_ values and their residuals for test set 1 compounds in cell line based QSAR model against HS-638**

| **No.** | **MaVC** | **A1ERC** | **MiVO** | **Exp.** | **Pred.** | **Res.** |
| --- | --- | --- | --- | --- | --- | --- |
| 2 | 3.99E+00 | 9.75E-05 | 2.00E+00 | 2.4089 | 2.16 | 0.25 |
| 4 | 3.99E+00 | -3.16E-06 | 1.99E+00 |  | 2.36 |  |
| 1 | 3.99E+00 | -1.53E-05 | 2.01E+00 | 2.4318 | 2.32 | 0.11 |
| 6 | 3.99E+00 | 6.19E-05 | 1.98E+00 |  | 2.28 |  |
| 7 | 3.99E+00 | 7.94E-05 | 1.99E+00 |  | 2.22 |  |
| 20 | 4.03E+00 | 2.51E-04 | 1.99E+00 | 2.301 | 2.32 | -0.02 |
| 9 | 3.99E+00 | 7.56E-05 | 2.00E+00 |  | 2.20 |  |
| 10 | 4.00E+00 | -8.91E-06 | 1.99E+00 | 2.3768 | 2.46 | -0.09 |
| 11 | 4.06E+00 | 5.64E-05 | 2.00E+00 |  | 2.91 |  |
| 12 | 3.99E+00 | 5.29E-05 | 2.00E+00 | 2.0605 | 2.23 | -0.17 |
| 15 | 3.99E+00 | -5.68E-05 | 1.96E+00 | 2.5229 | 2.53 | -0.01 |
| 16 | 4.06E+00 | -4.57E-05 | 2.00E+00 | 3.0969 | 3.08 | 0.02 |
| 17 | 3.99E+00 | -6.29E-05 | 1.99E+00 | 2.5229 | 2.46 | 0.07 |
| 18 | 3.99E+00 | -7.04E-05 | 1.99E+00 | 2.4437 | 2.47 | -0.03 |
| 19 | 4.01E+00 | 7.65E-05 | 1.96E+00 | 2.5528 | 2.50 | 0.05 |
| 21 | 3.99E+00 | 7.94E-05 | 1.95E+00 | 2.3372 | 2.33 | 0.01 |
| 23 | 3.99E+00 | 1.45E-04 | 1.96E+00 | 2.2366 | 2.19 | 0.05 |
| 24 | 3.99E+00 | -6.48E-05 | 1.96E+00 | 2.585 | 2.54 | 0.04 |
| 25 | 3.99E+00 | -1.72E-05 | 1.97E+00 | 2.301 | 2.44 | -0.13 |
| 26 | 4.00E+00 | -5.38E-05 | 2.00E+00 |  | 2.51 |  |
| 27 | 4.00E+00 | -2.93E-05 | 1.92E+00 |  | 2.69 |  |
| 28 | 4.00E+00 | 4.15E-05 | 1.91E+00 |  | 2.60 |  |
| 29 | 4.00E+00 | -4.04E-05 | 2.00E+00 |  | 2.49 |  |
| 30 | 4.00E+00 | -8.67E-06 | 2.00E+00 |  | 2.43 |  |
| 31 | 4.00E+00 | -3.52E-05 | 2.00E+00 |  | 2.48 |  |
| 32 | 4.00E+00 | 1.93E-04 | 2.00E+00 | 2.0088 | 2.10 | -0.09 |
| 33 | 3.99E+00 | 9.64E-05 | 1.78E+00 |  | 2.78 |  |
| 34 | 4.00E+00 | 3.12E-05 | 2.00E+00 |  | 2.37 |  |
| 13 | 3.99E+00 | 1.69E-05 | 2.00E+00 | 2.2518 | 2.29 | -0.04 |
| 37 | 4.00E+00 | 4.58E-05 | 2.00E+00 |  | 2.34 |  |
| 37 | 4.01E+00 | -3.89E-05 | 2.00E+00 |  | 2.58 |  |
| 39 | 4.00E+00 | -4.17E-05 | 2.00E+00 |  | 2.49 |  |
| 5 | 3.99E+00 | -1.12E-05 | 2.00E+00 | 2.3188 | 2.34 | -0.02 |
| 22* | 3.99E+00 | -8.25E-05 | 1.96E+00 | 2.6778 | 2.57 | 0.11 |
| 3* | 3.99E+00 | -3.07E-05 | 2.00E+00 | 2.1612 | 2.37 | -0.21 |
| 14* | 3.99E+00 | 5.35E-06 | 1.98E+00 | 2.4815 | 2.37 | 0.11 |
| 35* | 4.00E+00 | 9.59E-05 | 2.00E+00 | 2.2596 | 2.26 | 0.00 |
| 36* | 3.99E+00 | -3.85E-05 | 2.00E+00 | 2.1938 | 2.39 | -0.19 |
| 8# | 4.01E+00 | -8.12E-06 | 1.99E+00 | 2.1549 | 2.56 | -0.40 |

**Table S23: Descriptor, experimental and predicted pIC_50_ values and their residuals for test set 1 compounds in cell line based QSAR model against KB**

| **No.** | **HC-2TZ** | **Mi1ERN** | **MiNACH** | **Exp.** | **Pred.** | **Res.** |
| --- | --- | --- | --- | --- | --- | --- |
| 103 | 1.86E-04 | -3.08E-04 | 0.195 | 2.9547 | 3.00 | -0.05 |
| 104 | 1.43E-04 | -3.44E-04 | 0.186 | 1.8586 | 2.02 | -0.16 |
| 110 | 1.36E-03 | -3.48E-04 | 0.200 | 3.9586 | 3.88 | 0.08 |
| 111 | 1.03E-03 | -4.56E-04 | 0.205 | 2.8996 | 2.97 | -0.07 |
| 114 | 0.00E+00 | -3.03E-04 | 0.190 | 2.8861 | 2.56 | 0.33 |
| 116 | 1.49E-03 | -3.11E-04 | 0.193 | 4.0969 | 3.84 | 0.26 |
| 118 | 1.47E-03 | -3.17E-04 | 0.190 | 3.7447 | 3.56 | 0.18 |
| 119 | 1.29E-03 | -2.97E-04 | 0.192 | 3.7447 | 3.74 | 0.00 |
| 122 | 3.21E-04 | -1.51E-04 | 0.197 | 4.4437 | 4.68 | -0.24 |
| 121 | 1.33E-03 | -3.21E-04 | 0.189 | 3.7447 | 3.35 | 0.40 |
| 105 | 7.48E-04 | -3.20E-04 | 0.186 | 2.061 | 2.71 | -0.64 |
| 102 | 6.30E-04 | -3.03E-04 | 0.191 | 3.1367 | 3.11 | 0.02 |
| 120 | 2.59E-04 | -3.11E-04 | 0.193 | 3.0458 | 2.89 | 0.16 |
| 109 | 8.70E-04 | -3.31E-04 | 0.195 | 2.9508 | 3.31 | -0.36 |
| 115 | 7.91E-04 | -3.14E-04 | 0.191 | 3.585 | 3.14 | 0.45 |
| 117 | 1.50E-03 | -3.42E-04 | 0.193 | 3.0809 | 3.56 | -0.48 |
| 112 | 0.00E+00 | -3.02E-04 | 0.197 | 3.1805 | 3.05 | 0.13 |
| 107* | 6.97E-04 | -3.14E-04 | 0.192 | 3.2596 | 3.13 | 0.13 |
| 106* | 2.17E-04 | -2.97E-04 | 0.197 | 3.0655 | 3.26 | -0.20 |
| 113* | 1.28E-04 | -3.19E-04 | 0.195 | 2.7122 | 2.85 | -0.14 |
| 108* | 0.00E+00 | -3.35E-04 | 1.94E-01 | 2.0315 | 2.54 | -0.51 |

**Table S24: Descriptor, experimental and predicted pIC_50_ values and their residuals for test set 1 compounds in cell line based QSAR model against KBvin**

| **No.** | **HC-1/T** | **PP/SD** | **MaNACC** | **Exp.** | **Pred.** | **Res.** |
| --- | --- | --- | --- | --- | --- | --- |
| 103 | 3.24E-04 | 1.92E-03 | 9.44E-01 | 3.025 | 3.03 | 0.00 |
| 104 | 1.93E-04 | 1.97E-03 | 9.46E-01 | 3.024 | 3.03 | 0.00 |
| 106 | 4.47E-04 | 1.64E-03 | 9.46E-01 | 3.024 | 3.03 | 0.00 |
| 107 | 2.61E-03 | 2.82E-03 | 3.17E-01 | 3.499 | 3.49 | 0.01 |
| 108 | 0.00E+00 | 2.38E-03 | 1.14E+00 | 2.943 | 2.88 | 0.07 |
| 110 | 9.60E-03 | 2.82E-03 | 5.77E-01 | 3.239 | 3.26 | -0.02 |
| 111 | 6.42E-03 | 2.10E-03 | 8.40E-01 | 3.076 | 3.08 | 0.00 |
| 112 | 0.00E+00 | 1.59E-03 | 3.18E-01 | 3.497 | 3.51 | -0.02 |
| 113 | 9.96E-05 | 2.05E-03 | 8.35E-01 | 3.078 | 3.11 | -0.03 |
| 114 | 0.00E+00 | 2.26E-03 | 8.34E-01 | 3.079 | 3.11 | -0.03 |
| 115 | 2.50E-03 | 2.82E-03 | 3.17E-01 | 3.499 | 3.50 | 0.01 |
| 116 | 8.05E-03 | 1.68E-03 | 3.17E-01 | 3.499 | 3.47 | 0.03 |
| 117 | 9.11E-03 | 2.81E-03 | 5.81E-01 | 3.236 | 3.26 | -0.02 |
| 119 | 6.16E-03 | 1.68E-03 | 3.17E-01 | 3.499 | 3.48 | 0.02 |
| 120 | 6.38E-04 | 1.58E-03 | 9.40E-01 | 3.027 | 3.03 | 0.00 |
| 122 | 5.60E-04 | 5.31E-03 | 3.16E-01 | 3.500 | 3.49 | 0.01 |
| 121 | 5.58E-03 | 1.48E-03 | 3.16E-01 | 3.500 | 3.49 | 0.01 |
| 102* | 2.14E-03 | 2.82E-03 | 3.17E-01 | 3.499 | 3.50 | 0.00 |
| 105* | 3.03E-03 | 2.81E-03 | 3.17E-01 | 3.499 | 3.49 | 0.01 |
| 109* | 5.93E-03 | 2.81E-03 | 3.18E-01 | 3.497 | 3.48 | 0.02 |
| 118* | 6.75E-03 | 1.52E-03 | 3.17E-01 | 3.499 | 3.48 | 0.02 |

**Table S25: Descriptor, experimental and predicted pIC_50_ values and their residuals for test set 1 compounds in cell line based QSAR model against LNCaP**

| **No.** | **ZXS** | **SIC0** | **RNN** | **Exp.** | **Pred.** | **Res.** |
| --- | --- | --- | --- | --- | --- | --- |
| 192 | 1.58E+02 | 1.96E+01 | 2.50E-02 | 2.377 | 2.02 | 0.36 |
| 193 | 1.58E+02 | 2.13E+01 | 3.70E-02 | 3.000 | 2.43 | 0.57 |
| 161 | 1.72E+02 | 2.34E+01 | 2.17E-02 | 2.796 | 3.11 | -0.32 |
| 199 | 1.10E+02 | 1.55E+01 | 1.56E-02 | 2.721 | 2.46 | 0.26 |
| 165 | 1.69E+02 | 2.23E+01 | 2.27E-02 | 2.620 | 2.75 | -0.13 |
| 167 | 1.70E+02 | 2.27E+01 | 2.33E-02 | 3.097 | 2.86 | 0.23 |
| 169 | 1.50E+02 | 2.04E+01 | 2.56E-02 | 2.301 | 2.65 | -0.35 |
| 170 | 1.56E+02 | 2.04E+01 | 2.56E-02 | 2.959 | 2.41 | 0.55 |
| 172 | 1.47E+02 | 2.13E+01 | 3.70E-02 | 2.482 | 2.86 | -0.38 |
| 174 | 1.75E+02 | 2.25E+01 | 3.33E-02 | 2.745 | 2.34 | 0.41 |
| 175 | 9.84E+01 | 1.84E+01 | 5.26E-02 | 2.244 | 3.21 | -0.97 |
| 176 | 1.03E+02 | 1.84E+01 | 5.26E-02 | 2.013 | 3.03 | -1.01 |
| 179 | 1.16E+02 | 1.72E+01 | 5.66E-02 | 2.456 | 1.92 | 0.54 |
| 180 | 9.56E+01 | 1.76E+01 | 8.51E-02 |  | 2.20 |  |
| 182 | 1.56E+02 | 2.27E+01 | 3.41E-02 | 2.699 | 3.15 | -0.46 |
| 188 | 1.47E+02 | 2.04E+01 | 3.66E-02 | 2.328 | 2.50 | -0.17 |
| 184 | 1.58E+02 | 1.99E+01 | 2.56E-02 | 2.854 | 2.13 | 0.73 |
| 185 | 1.64E+02 | 2.29E+01 | 3.49E-02 | 1.975 | 2.90 | -0.93 |
| 186 | 1.51E+02 | 2.17E+01 | 2.50E-02 |  | 3.16 |  |
| 187 | 1.35E+02 | 1.75E+01 | 2.67E-02 | 2.409 | 2.02 | 0.39 |
| 189 | 1.66E+02 | 2.28E+01 | 3.66E-02 | 2.319 | 2.74 | -0.42 |
| 190 | 1.63E+02 | 2.24E+01 | 3.70E-02 | 2.699 | 2.68 | 0.02 |
| 191 | 1.61E+02 | 2.17E+01 | 5.00E-02 | 2.745 | 2.15 | 0.59 |
| 194 | 4.16E+01 | 1.13E+01 | 2.61E-01 |  | -2.57 |  |
| 229 | 8.30E+01 | 1.78E+01 | 4.17E-02 |  | 3.84 |  |
| 230 | 1.06E+02 | 2.13E+01 | 3.33E-02 |  | 4.58 |  |
| 231 | 7.83E+01 | 1.77E+01 | 4.35E-02 |  | 3.94 |  |
| 233 | 7.98E+01 | 1.68E+01 | 4.55E-02 |  | 3.46 |  |
| 235 | 5.77E+01 | 9.20E+00 | 3.33E-02 |  | 1.50 |  |
| 236 | 6.49E+01 | 1.20E+01 | 2.94E-02 |  | 2.47 |  |
| 237 | 6.60E+01 | 1.20E+01 | 2.94E-02 |  | 2.42 |  |
| 238 | 7.49E+01 | 1.20E+01 | 2.94E-02 |  | 2.07 |  |
| 239 | 7.13E+01 | 1.39E+01 | 2.63E-02 |  | 3.07 |  |
| 234 | 7.17E+01 | 1.55E+01 | 2.38E-02 | 4.553 | 3.78 | 0.78 |
| 240 | 8.15E+01 | 1.39E+01 | 2.63E-02 | 3.491 | 2.67 | 0.82 |
| 241 | 5.51E+01 | 1.02E+01 | 3.33E-02 |  | 2.02 |  |
| 242 | 1.12E+02 | 1.82E+01 | 1.47E-02 |  | 3.52 |  |
| 243 | 7.70E+01 | 1.71E+01 | 2.13E-02 | 5.222 | 4.29 | 0.94 |
| 244 | 7.26E+01 | 1.75E+01 | 2.27E-02 | 4.276 | 4.59 | -0.32 |
| 248 | 7.94E+01 | 1.89E+01 | 4.35E-02 | 4.409 | 4.39 | 0.02 |
| 246 | 7.98E+01 | 1.89E+01 | 1.92E-02 | 5.222 | 4.97 | 0.25 |
| 247 | 7.98E+01 | 1.89E+01 | 1.92E-02 | 4.420 | 4.97 | -0.55 |
| 257 | 9.84E+01 | 1.62E+01 | 0.00E+00 |  | 3.59 |  |
| 258 | 1.03E+02 | 1.70E+01 | 0.00E+00 |  | 3.74 |  |
| 250 | 7.90E+01 | 1.94E+01 | 2.13E-02 | 4.027 | 5.16 | -1.13 |
| 251 | 7.87E+01 | 1.75E+01 | 2.27E-02 | 4.357 | 4.35 | 0.01 |
| 252 | 7.94E+01 | 1.78E+01 | 2.00E-02 | 4.222 | 4.51 | -0.29 |
| 253 | 6.86E+01 | 1.69E+01 | 4.65E-02 |  | 3.92 |  |
| 254 | 6.91E+01 | 1.72E+01 | 7.14E-02 | 2.551 | 3.42 | -0.86 |
| 255 | 6.95E+01 | 1.66E+01 | 2.44E-02 | 4.658 | 4.30 | 0.36 |
| 256 | 5.42E+01 | 1.45E+01 | 2.94E-02 |  | 3.92 |  |
| 214 | 8.66E+01 | 2.32E+01 | 5.80E-02 | 6.000 | 5.52 | 0.48 |
| 227 | 1.32E+02 | 2.13E+01 | 3.70E-02 | 3.000 | 3.46 | -0.46 |
| 228 | 9.33E+01 | 1.77E+01 | 5.56E-02 | 2.456 | 3.05 | -0.59 |
| 200 | 5.90E+01 | 1.39E+01 | 2.63E-02 |  | 3.56 |  |
| 201 | 7.00E+01 | 1.53E+01 | 2.08E-02 | 2.854 | 3.84 | -0.98 |
| 202 | 5.84E+01 | 1.47E+01 | 5.41E-02 | 2.187 | 3.23 | -1.05 |
| 203 | 6.90E+01 | 1.62E+01 | 4.26E-02 | 4.114 | 3.71 | 0.40 |
| 204 | 6.83E+01 | 1.55E+01 | 4.35E-02 | 3.699 | 3.43 | 0.27 |
| 205 | 6.76E+01 | 1.55E+01 | 4.35E-02 | 2.745 | 3.46 | -0.71 |
| 206 | 7.08E+01 | 1.92E+01 | 5.36E-02 | 5.398 | 4.61 | 0.79 |
| 208 | 7.62E+01 | 2.11E+01 | 4.92E-02 | 6.000 | 5.29 | 0.72 |
| 210 | 8.76E+01 | 2.14E+01 | 4.76E-02 | 5.097 | 5.00 | 0.10 |
| 212 | 7.87E+01 | 2.19E+01 | 6.15E-02 | 5.046 | 5.22 | -0.17 |
| 216 | 7.50E+01 | 1.92E+01 | 5.36E-02 | 3.222 | 4.44 | -1.22 |
| 217 | 8.11E+01 | 1.92E+01 | 5.36E-02 |  | 4.20 |  |
| 207 | 6.78E+01 | 2.13E+01 | 6.56E-02 | 6.000 | 5.30 | 0.70 |
| 209 | 8.30E+01 | 2.29E+01 | 6.06E-02 |  | 5.48 |  |
| 211 | 8.61E+01 | 2.34E+01 | 5.88E-02 | 6.000 | 5.61 | 0.39 |
| 213 | 8.30E+01 | 2.35E+01 | 7.14E-02 |  | 5.46 |  |
| 215 | 8.91E+01 | 2.47E+01 | 6.76E-02 | 6.000 | 5.81 | 0.19 |
| 219 | 6.20E+01 | 2.02E+01 | 7.27E-02 | 5.000 | 4.90 | 0.10 |
| 222 | 6.05E+01 | 1.76E+01 | 6.38E-02 |  | 4.11 |  |
| 223 | 6.11E+01 | 1.88E+01 | 5.88E-02 | 5.699 | 4.70 | 1.00 |
| 224 | 6.82E+01 | 1.88E+01 | 5.88E-02 | 5.699 | 4.42 | 1.28 |
| 225 | 6.03E+01 | 1.85E+01 | 7.84E-02 | 5.523 | 4.13 | 1.40 |
| 218 | 8.83E+01 | 2.39E+01 | 5.56E-02 | 5.398 | 5.80 | -0.41 |
| 220 | 6.28E+01 | 1.97E+01 | 7.14E-02 | 4.125 | 4.70 | -0.57 |
| 221 | 6.18E+01 | 1.91E+01 | 7.55E-02 | 3.620 | 4.39 | -0.77 |
| 226 | 6.22E+01 | 1.92E+01 | 7.69E-02 | 3.959 | 4.38 | -0.42 |
| 232# | 8.31E+01 | 1.77E+01 | 4.35E-02 | 1.7747 | 3.75 | -1.98 |
| 162* | 1.74E+02 | 2.38E+01 | 2.22E-02 | 3.1549 | 3.19 | -0.03 |
| 163* | 1.72E+02 | 2.38E+01 | 2.22E-02 | 3.2218 | 3.27 | -0.04 |
| 198* | 1.10E+02 | 1.73E+01 | 3.23E-02 | 2.699 | 2.79 | -0.09 |
| 197* | 1.00E+02 | 1.64E+01 | 3.57E-02 | 2.8539 | 2.74 | 0.12 |
| 196* | 9.89E+01 | 1.64E+01 | 3.57E-02 | 2.6576 | 2.78 | -0.12 |
| 195* | 8.99E+01 | 1.56E+01 | 4.00E-02 | 2.5686 | 2.70 | -0.13 |
| 166* | 1.69E+02 | 2.23E+01 | 2.27E-02 | 2.8861 | 2.75 | 0.13 |
| 168* | 1.56E+02 | 2.04E+01 | 2.56E-02 | 2.4437 | 2.41 | 0.03 |
| 173* | 1.51E+02 | 2.13E+01 | 3.70E-02 | 2.8539 | 2.70 | 0.15 |
| 178* | 9.74E+01 | 1.75E+01 | 5.45E-02 | 2.7696 | 2.83 | -0.06 |
| 183* | 1.45E+02 | 1.99E+01 | 2.56E-02 | 2.7959 | 2.64 | 0.15 |
| 245* | 7.29E+01 | 1.75E+01 | 2.27E-02 | 4.9586 | 4.58 | 0.38 |
| 249* | 7.23E+01 | 1.86E+01 | 4.44E-02 | 4.8239 | 4.53 | 0.30 |
| 181* | 1.50E+02 | 2.15E+01 | 3.80E-02 | 2.7447 | 2.80 | -0.06 |
| 177* | 9.59E+01 | 1.77E+01 | 5.56E-02 | 2.4559 | 2.95 | -0.49 |

**Table S26: Descriptor, experimental and predicted pIC_50_ values and their residuals for test set 1 compounds in cell line based QSAR model against LoVo**

| **No.** | **MaERC** | **MaBO** | **MaVH** | **Exp.** | **Pred.** | **Res.** |
| --- | --- | --- | --- | --- | --- | --- |
| 4 | 1.56E-02 | 1.67E+00 | 9.70E-01 |  | 2.39 |  |
| 1 | 2.43E-02 | 1.67E+00 | 9.69E-01 | 3.398 | 3.36 | 0.04 |
| 3 | 1.51E-02 | 1.66E+00 | 9.70E-01 | 2.328 | 2.28 | 0.05 |
| 5 | 1.55E-02 | 1.71E+00 | 9.79E-01 | 2.181 | 2.16 | 0.02 |
| 6 | 1.45E-02 | 1.70E+00 | 9.70E-01 |  | 2.43 |  |
| 7 | 1.55E-02 | 1.68E+00 | 9.70E-01 | 2.000 | 2.43 | -0.43 |
| 8 | 1.55E-02 | 1.68E+00 | 9.70E-01 | 2.319 | 2.43 | -0.11 |
| 9 | 1.55E-02 | 1.75E+00 | 9.70E-01 |  | 2.79 |  |
| 10 | 1.54E-02 | 1.68E+00 | 9.70E-01 | 2.886 | 2.42 | 0.47 |
| 12 | 1.55E-02 | 1.70E+00 | 9.70E-01 | 2.347 | 2.53 | -0.19 |
| 13 | 1.53E-02 | 1.69E+00 | 9.70E-01 | 2.432 | 2.46 | -0.03 |
| 14 | 1.59E-02 | 1.67E+00 | 9.70E-01 | 2.387 | 2.42 | -0.03 |
| 18 | 1.50E-02 | 1.68E+00 | 9.70E-01 | 2.114 | 2.38 | -0.26 |
| 16 | 1.63E-02 | 1.75E+00 | 9.69E-01 | 3.046 | 2.93 | 0.12 |
| 17 | 1.51E-02 | 1.70E+00 | 9.70E-01 | 2.678 | 2.49 | 0.19 |
| 20 | 1.68E-02 | 1.67E+00 | 9.69E-01 | 2.377 | 2.56 | -0.19 |
| 19 | 1.55E-02 | 1.67E+00 | 9.70E-01 | 2.699 | 2.38 | 0.32 |
| 22 | 1.56E-02 | 1.77E+00 | 9.70E-01 | 2.824 | 2.91 | -0.09 |
| 21 | 1.55E-02 | 1.67E+00 | 9.70E-01 | 2.745 | 2.38 | 0.37 |
| 26 | 1.57E-02 | 1.66E+00 | 9.71E-01 | 2.081 | 2.30 | -0.22 |
| 23 | 1.55E-02 | 1.67E+00 | 9.70E-01 | 2.357 | 2.38 | -0.02 |
| 24 | 1.55E-02 | 1.67E+00 | 9.70E-01 | 2.495 | 2.38 | 0.12 |
| 25 | 1.60E-02 | 1.67E+00 | 9.70E-01 | 2.553 | 2.43 | 0.12 |
| 27 | 1.63E-02 | 1.67E+00 | 9.72E-01 |  | 2.37 |  |
| 29 | 1.59E-02 | 1.66E+00 | 9.70E-01 | 2.301 | 2.37 | -0.07 |
| 30 | 1.63E-02 | 1.67E+00 | 9.70E-01 | 2.387 | 2.46 | -0.08 |
| 31 | 1.78E-02 | 1.67E+00 | 9.70E-01 |  | 2.62 |  |
| 32 | 1.61E-02 | 1.67E+00 | 9.70E-01 | 2.319 | 2.44 | -0.12 |
| 33 | 1.61E-02 | 1.66E+00 | 9.70E-01 |  | 2.39 |  |
| 35 | 1.58E-02 | 1.66E+00 | 9.70E-01 | 2.398 | 2.36 | 0.04 |
| 36 | 1.58E-02 | 1.67E+00 | 9.70E-01 | 2.398 | 2.41 | -0.01 |
| 37 | 1.57E-02 | 1.67E+00 | 9.70E-01 |  | 2.40 |  |
| 39 | 1.63E-02 | 1.67E+00 | 9.69E-01 |  | 2.51 |  |
| 2* | 1.70E-02 | 1.67E+00 | 9.70E-01 | 2.569 | 2.54 | 0.03 |
| 15* | 1.64E-02 | 1.75E+00 | 9.70E-01 | 2.538 | 2.89 | -0.35 |
| 28* | 1.52E-02 | 1.66E+00 | 9.70E-01 | 2.076 | 2.29 | -0.22 |
| 34* | 1.48E-02 | 1.67E+00 | 9.70E-01 | 2.092 | 2.30 | -0.21 |
| 32* | 1.57E-02 | 1.67E+00 | 9.70E-01 | 2.061 | 2.40 | -0.34 |
| 11* | 1.49E-02 | 1.69E+00 | 9.70E-01 | 2.194 | 2.42 | -0.22 |

**Table S27: Descriptor, experimental and predicted pIC_50_ values and their residuals for test set 1 compounds in cell line based QSAR model against MB231**

| **No.** | **YZS** | **FBCSQ** | **MaPBO** | **Exp.** | **Pred.** | **Res.** |
| --- | --- | --- | --- | --- | --- | --- |
| 40 | 4.61E+01 | 2.35E-01 | 9.91E-01 | 2.070 | 2.36 | -0.29 |
| 42 | 3.14E+01 | 2.14E-01 | 9.37E-01 |  | 2.64 |  |
| 43 | 3.86E+01 | 2.26E-01 | 9.42E-01 |  | 2.49 |  |
| 44 | 5.71E+01 | 2.37E-01 | 9.92E-01 | 1.630 | 2.02 | -0.39 |
| 45 | 5.10E+01 | 2.38E-01 | 9.91E-01 | 2.770 | 2.22 | 0.55 |
| 46 | 5.63E+01 | 2.28E-01 | 9.93E-01 |  | 2.00 |  |
| 47 | 5.24E+01 | 2.04E-01 | 9.92E-01 | 2.230 | 1.99 | 0.24 |
| 48 | 5.15E+01 | 2.29E-01 | 9.89E-01 | 2.120 | 2.15 | -0.03 |
| 49 | 5.93E+01 | 2.30E-01 | 9.89E-01 | 2.390 | 1.91 | 0.48 |
| 50 | 4.41E+01 | 2.39E-01 | 9.91E-01 | 2.390 | 2.44 | -0.05 |
| 53 | 5.01E+01 | 2.36E-01 | 9.88E-01 |  | 2.23 |  |
| 54 | 5.85E+01 | 2.36E-01 | 9.89E-01 | 2.490 | 1.97 | 0.52 |
| 55 | 5.08E+01 | 2.37E-01 | 9.89E-01 |  | 2.22 |  |
| 56 | 4.25E+01 | 2.40E-01 | 9.91E-01 | 2.170 | 2.50 | -0.33 |
| 58 | 5.56E+01 | 2.21E-01 | 9.89E-01 | 2.330 | 1.98 | 0.35 |
| 59 | 4.60E+01 | 2.52E-01 | 9.95E-01 | 2.240 | 2.45 | -0.21 |
| 60 | 4.58E+01 | 2.62E-01 | 9.95E-01 | 2.800 | 2.51 | 0.29 |
| 61 | 5.24E+01 | 3.11E-01 | 9.43E-01 |  | 2.51 |  |
| 62 | 5.45E+01 | 2.88E-01 | 9.40E-01 |  | 2.31 |  |
| 63 | 5.51E+01 | 2.01E-01 | 9.95E-01 | 1.870 | 1.90 | -0.03 |
| 64 | 5.47E+01 | 2.11E-01 | 9.94E-01 | 1.940 | 1.96 | -0.02 |
| 66 | 5.31E+01 | 2.08E-01 | 9.87E-01 | 1.660 | 1.99 | -0.33 |
| 68 | 6.10E+01 | 2.70E-01 | 9.96E-01 |  | 2.08 |  |
| 70 | 5.24E+01 | 2.95E-01 | 9.92E-01 | 1.650 | 2.48 | -0.83 |
| 71 | 5.25E+01 | 2.35E-01 | 9.91E-01 | 2.140 | 2.16 | -0.02 |
| 269 | 5.01E+01 | 3.54E-01 | 8.43E-01 |  | 2.69 |  |
| 275 | 4.39E+01 | 2.69E-01 | 8.43E-01 |  | 2.43 |  |
| 74 | 4.70E+01 | 1.59E-01 | 9.80E-01 | 1.680 | 1.91 | -0.23 |
| 261 | 4.01E+01 | 2.66E-01 | 8.51E-01 | 3.030 | 2.55 | 0.49 |
| 262 | 4.91E+01 | 4.87E-01 | 8.44E-01 | 3.550 | 3.43 | 0.12 |
| 263 | 5.17E+01 | 4.90E-01 | 8.35E-01 | 3.680 | 3.35 | 0.33 |
| 264 | 4.53E+01 | 3.59E-01 | 1.98E+00 | 4.150 | 4.20 | -0.05 |
| 265 | 4.80E+01 | 3.54E-01 | 1.98E+00 | 3.850 | 4.08 | -0.23 |
| 266 | 4.45E+01 | 3.93E-01 | 9.94E-01 | 3.920 | 3.25 | 0.67 |
| 267 | 4.72E+01 | 3.52E-01 | 8.51E-01 | 2.630 | 2.78 | -0.15 |
| 268 | 4.75E+01 | 4.77E-01 | 8.43E-01 | 3.640 | 3.42 | 0.22 |
| 270 | 5.15E+01 | 4.54E-01 | 8.45E-01 | 3.700 | 3.18 | 0.52 |
| 273 | 4.98E+01 | 4.04E-01 | 8.56E-01 |  | 2.98 |  |
| 276 | 5.18E+01 | 3.97E-01 | 8.43E-01 |  | 2.86 |  |
| 277 | 4.96E+01 | 3.86E-01 | 8.56E-01 |  | 2.89 |  |
| 276 | 5.13E+01 | 3.76E-01 | 8.43E-01 |  | 2.77 |  |
| 280 | 5.32E+01 | 4.71E-01 | 8.37E-01 | 2.450 | 3.20 | -0.75 |
| 281 | 4.84E+01 | 3.67E-01 | 8.43E-01 |  | 2.81 |  |
| 282 | 4.84E+01 | 4.70E-01 | 8.41E-01 | 3.640 | 3.35 | 0.29 |
| 283 | 4.83E+01 | 2.31E-01 | 8.43E-01 | 2.320 | 2.09 | 0.23 |
| 285 | 4.72E+01 | 2.36E-01 | 8.43E-01 | 2.020 | 2.15 | -0.13 |
| 287 | 4.73E+01 | 2.74E-01 | 9.97E-01 | 1.980 | 2.53 | -0.55 |
| 288 | 4.84E+01 | 3.85E-01 | 9.97E-01 | 3.770 | 3.09 | 0.68 |
| 289 | 4.49E+01 | 2.49E-01 | 1.99E+00 | 3.920 | 3.64 | 0.28 |
| 290 | 4.66E+01 | 4.96E-01 | 8.43E-01 |  | 3.55 |  |
| 291 | 5.61E+01 | 4.72E-01 | 8.45E-01 |  | 3.13 |  |
| 292 | 4.56E+01 | 4.01E-01 | 8.47E-01 |  | 3.08 |  |
| 293 | 4.73E+01 | 3.96E-01 | 8.38E-01 |  | 2.99 |  |
| 294 | 5.04E+01 | 3.50E-01 | 9.94E-01 |  | 2.83 |  |
| 295 | 5.00E+01 | 3.97E-01 | 8.56E-01 |  | 2.93 |  |
| 72 | 4.76E+01 | 2.72E-01 | 9.91E-01 | 1.930 | 2.51 | -0.58 |
| 278 | 5.09E+01 | 4.86E-01 | 8.47E-01 | 2.280 | 3.37 | -1.09 |
| 41 | 5.93E+01 | 2.44E-01 | 9.92E-01 | 2.010 | 1.99 | 0.02 |
| 274# | 5.02E+01 | 4.96E-01 | 8.44E-01 | 2.250 | 3.44 | -1.19 |
| 51* | 4.97E+01 | 2.53E-01 | 9.91E-01 | 2.490 | 2.34 | 0.15 |
| 57* | 5.37E+01 | 2.17E-01 | 9.88E-01 | 2.540 | 2.02 | 0.52 |
| 67* | 6.99E+01 | 2.39E-01 | 9.93E-01 | 1.800 | 1.63 | 0.17 |
| 69* | 5.60E+01 | 2.65E-01 | 9.92E-01 | 1.930 | 2.21 | -0.28 |
| 271* | 5.11E+01 | 4.67E-01 | 8.44E-01 | 3.640 | 3.26 | 0.38 |
| 284* | 4.87E+01 | 3.41E-01 | 8.42E-01 | 2.530 | 2.66 | -0.13 |
| 73* | 5.08E+01 | 2.43E-01 | 9.90E-01 | 1.840 | 2.25 | -0.41 |
| 52* | 4.47E+01 | 2.40E-01 | 9.91E-01 | 2.960 | 2.43 | 0.53 |
| 65* | 6.74E+01 | 2.22E-01 | 9.94E-01 | 1.970 | 1.62 | 0.35 |
| 272* | 4.42E+01 | 2.72E-01 | 8.56E-01 | 2.450 | 2.45 | 0.00 |
| 286* | 4.98E+01 | 3.49E-01 | 8.38E-01 | 2.390 | 2.67 | -0.28 |

**Table S28: Descriptor, experimental and predicted pIC_50_ values and their residuals for test set 1 compounds in cell line based QSAR model against MB468**

| **No.** | **RPCSZ** | **RNCSZ** | **MiBOH** | **Exp.** | **Pred.** | **Res.** |
| --- | --- | --- | --- | --- | --- | --- |
| 40 | 8.70E-02 | 1.65E+01 | 7.58E-01 | 1.980 | 2.37 | -0.39 |
| 41 | 0.00E+00 | 3.69E+00 | 7.58E-01 | 1.950 | 2.15 | -0.20 |
| 42 | 4.26E+00 | 4.62E+00 | 8.08E-01 |  | 1.21 |  |
| 43 | 3.87E+00 | 4.85E+00 | 8.08E-01 |  | 1.29 |  |
| 44 | 0.00E+00 | 3.39E+00 | 7.58E-01 |  | 2.14 |  |
| 45 | 1.66E-01 | 1.58E+01 | 7.58E-01 | 2.680 | 2.35 | 0.33 |
| 46 | 0.00E+00 | 7.95E-01 | 7.69E-01 |  | 2.06 |  |
| 47 | 0.00E+00 | 8.28E+00 | 7.54E-01 | 2.390 | 2.25 | 0.15 |
| 49 | 8.65E-02 | 1.34E+01 | 7.70E-01 | 2.640 | 2.28 | 0.36 |
| 51 | 1.61E-01 | 1.43E+01 | 7.57E-01 | 2.520 | 2.32 | 0.20 |
| 52 | 8.39E-02 | 1.50E+01 | 7.57E-01 | 2.470 | 2.35 | 0.12 |
| 53 | 1.69E-01 | 1.21E+01 | 7.66E-01 |  | 2.25 |  |
| 54 | 1.62E-01 | 1.17E+01 | 7.69E-01 | 2.330 | 2.23 | 0.10 |
| 55 | 2.36E-01 | 1.05E+01 | 7.68E-01 |  | 2.20 |  |
| 56 | 7.88E-02 | 1.11E+01 | 7.62E-01 | 1.970 | 2.26 | -0.29 |
| 57 | 1.93E-01 | 1.41E+01 | 7.67E-01 | 2.210 | 2.28 | -0.07 |
| 58 | 1.85E-01 | 1.37E+01 | 7.68E-01 | 1.930 | 2.27 | -0.34 |
| 61 | 1.98E+00 | 9.15E+00 | 7.69E-01 |  | 1.84 |  |
| 62 | 2.01E+00 | 8.36E+00 | 7.69E-01 |  | 1.82 |  |
| 63 | 0.00E+00 | 1.29E+01 | 8.96E-01 | 1.800 | 1.89 | -0.09 |
| 64 | 8.01E-02 | 1.22E+01 | 8.96E-01 | 1.970 | 1.86 | 0.11 |
| 65 | 0.00E+00 | 3.38E+00 | 8.96E-01 | 1.730 | 1.71 | 0.02 |
| 66 | 1.91E-01 | 3.12E+00 | 8.95E-01 | 1.680 | 1.67 | 0.01 |
| 67 | 0.00E+00 | 8.29E-01 | 8.75E-01 | 1.690 | 1.73 | -0.04 |
| 68 | 3.65E+00 | 6.01E+00 | 7.17E-01 |  | 1.63 |  |
| 70 | 2.65E+00 | 7.05E+00 | 7.40E-01 | 1.670 | 1.77 | -0.10 |
| 72 | 1.50E+00 | 8.88E+00 | 7.27E-01 |  | 2.06 |  |
| 73 | 8.53E-01 | 5.97E+00 | 7.62E-01 | 1.800 | 2.02 | -0.22 |
| 74 | 2.35E+00 | 9.32E-01 | 7.50E-01 | 1.710 | 1.68 | 0.03 |
| 71* | 0.00E+00 | 8.34E-01 | 7.59E-01 | 1.970 | 2.09 | -0.12 |
| 48* | 1.80E-01 | 1.38E+01 | 7.67E-01 | 2.210 | 2.28 | -0.07 |
| 50* | 8.40E-02 | 1.47E+01 | 7.57E-01 | 2.260 | 2.34 | -0.08 |
| 69* | 2.62E+00 | 3.59E+00 | 7.40E-01 | 1.840 | 1.71 | 0.13 |
| 59* | 0.00E+00 | 5.94E+00 | 7.60E-01 | 2.400 | 2.18 | 0.22 |
| 60* | 7.56E-02 | 5.60E+00 | 7.60E-01 | 2.380 | 2.16 | 0.22 |

**Table S29: Descriptor, experimental and predicted pIC_50_ values and their residuals for test set 1 compounds in cell line based QSAR model against MCF-7**

| **No.** | **ZXS/ZXR** | **MiNRO** | **Mi1ERC** | **Exp.** | **Pred.** | **Res.** |
| --- | --- | --- | --- | --- | --- | --- |
| 296 | 5.61E-01 | 3.37E-04 | -1.83E-02 | 5.000 | 4.43 | 0.57 |
| 297 | 6.05E-01 | 6.00E-07 | -2.38E-02 |  | 4.76 |  |
| 299 | 6.42E-01 | 5.69E-04 | -2.37E-02 | 5.000 | 4.50 | 0.50 |
| 300 | 7.96E-01 | 1.44E-02 | -1.69E-02 | 1.220 | 2.07 | -0.85 |
| 301 | 7.44E-01 | 6.31E-04 | -1.45E-02 |  | 3.00 |  |
| 303 | 6.39E-01 | 6.35E-04 | -1.52E-02 | 3.520 | 3.66 | -0.14 |
| 305 | 6.50E-01 | 2.42E-06 | -1.58E-02 | 3.520 | 3.70 | -0.18 |
| 306 | 6.44E-01 | 7.74E-04 | -1.60E-02 | 3.360 | 3.71 | -0.35 |
| 307 | 6.74E-01 | 1.03E-04 | -1.59E-02 | 3.570 | 3.57 | 0.00 |
| 308 | 6.62E-01 | 6.34E-04 | -1.60E-02 |  | 3.61 |  |
| 309 | 7.09E-01 | 3.58E-07 | -1.61E-02 |  | 3.40 |  |
| 310 | 7.16E-01 | 6.28E-04 | -1.62E-02 |  | 3.33 |  |
| 313 | 6.64E-01 | 3.10E-08 | -1.64E-02 | 4.220 | 3.68 | 0.54 |
| 314 | 6.44E-01 | 6.94E-04 | -1.66E-02 |  | 3.77 |  |
| 315 | 7.03E-01 | 6.50E-04 | -1.65E-02 | 2.980 | 3.43 | -0.45 |
| 316 | 6.55E-01 | 1.64E-03 | -1.62E-02 | 3.420 | 3.61 | -0.19 |
| 317 | 6.81E-01 | 2.35E-07 | -1.63E-02 | 3.550 | 3.58 | -0.03 |
| 318 | 6.42E-01 | 1.63E-03 | -1.63E-02 |  | 3.69 |  |
| 320 | 6.96E-01 | 1.84E-04 | -8.38E-03 | 3.320 | 2.69 | 0.63 |
| 321 | 7.06E-01 | 1.29E-03 | -1.46E-02 |  | 3.18 |  |
| 322 | 6.83E-01 | 1.05E-04 | -1.60E-02 |  | 3.53 |  |
| 323 | 6.45E-01 | 1.40E-03 | -1.52E-02 |  | 3.58 |  |
| 324 | 6.49E-01 | 1.44E-03 | -1.60E-02 |  | 3.64 |  |
| 325 | 6.49E-01 | 1.41E-03 | -1.61E-02 |  | 3.65 |  |
| 327 | 6.74E-01 | 1.07E-04 | -1.62E-02 |  | 3.60 |  |
| 328 | 6.93E-01 | 1.36E-02 | -1.63E-02 | 2.660 | 2.64 | 0.02 |
| 329 | 6.92E-01 | 2.73E-05 | -1.69E-02 | 2.790 | 3.57 | -0.78 |
| 330 | 7.02E-01 | 1.37E-02 | -1.66E-02 |  | 2.61 |  |
| 331 | 6.69E-01 | 1.35E-02 | -1.55E-02 |  | 2.70 |  |
| 332 | 6.51E-01 | 1.41E-02 | -1.56E-02 | 2.480 | 2.77 | -0.29 |
| 334 | 7.05E-01 | 1.25E-04 | -1.52E-02 | 3.640 | 3.32 | 0.32 |
| 335 | 5.74E-01 | 1.48E-02 | -1.41E-02 | 3.720 | 3.01 | 0.71 |
| 336 | 6.67E-01 | 1.40E-02 | -1.58E-02 | 2.780 | 2.71 | 0.07 |
| 337 | 6.40E-01 | 1.42E-02 | -1.59E-02 | 1.960 | 2.86 | -0.90 |
| 338 | 6.70E-01 | 1.04E-04 | -1.57E-02 |  | 3.57 |  |
| 339 | 6.33E-01 | 1.38E-02 | -1.50E-02 |  | 2.83 |  |
| 340 | 6.87E-01 | 1.40E-02 | -1.57E-02 | 3.000 | 2.59 | 0.41 |
| 341 | 6.60E-01 | 8.53E-05 | -1.57E-02 | 3.430 | 3.63 | -0.20 |
| 342 | 6.96E-01 | 1.47E-02 | -1.52E-02 | 3.260 | 2.44 | 0.82 |
| 2 | 6.34E-01 | 1.75E-04 | -9.37E-04 | 2.240 | 2.29 | -0.05 |
| 4 | 6.46E-01 | 1.12E-06 | -1.08E-03 |  | 2.25 |  |
| 1 | 6.55E-01 | 3.31E-07 | -1.25E-03 |  | 2.21 |  |
| 3 | 6.20E-01 | 1.88E-04 | -1.13E-03 | 2.000 | 2.39 | -0.39 |
| 5 | 6.37E-01 | 9.21E-07 | -1.11E-03 | 2.130 | 2.30 | -0.17 |
| 7 | 6.73E-01 | 2.17E-06 | -8.84E-04 |  | 2.08 |  |
| 9 | 6.63E-01 | 1.11E-07 | -8.77E-04 |  | 2.13 |  |
| 10 | 6.59E-01 | 2.68E-07 | -1.11E-03 | 2.660 | 2.18 | 0.48 |
| 11 | 6.46E-01 | 1.28E-06 | -8.46E-04 |  | 2.22 |  |
| 12 | 6.75E-01 | 9.97E-07 | -8.49E-04 | 2.020 | 2.06 | -0.04 |
| 13 | 6.09E-01 | 2.06E-06 | -1.08E-03 |  | 2.46 |  |
| 14 | 6.33E-01 | 2.31E-07 | -1.10E-03 |  | 2.32 |  |
| 16 | 6.11E-01 | 2.57E-08 | -1.20E-03 | 2.740 | 2.46 | 0.28 |
| 17 | 6.94E-01 | 1.84E-07 | -1.38E-03 | 2.440 | 2.01 | 0.43 |
| 18 | 6.81E-01 | 9.42E-08 | -1.38E-03 | 2.200 | 2.08 | 0.12 |
| 19 | 6.09E-01 | 5.29E-07 | -8.58E-04 | 2.620 | 2.43 | 0.19 |
| 21 | 6.60E-01 | 1.19E-06 | -8.68E-04 | 2.410 | 2.15 | 0.26 |
| 22 | 6.32E-01 | 2.59E-08 | -1.44E-03 | 2.510 | 2.36 | 0.15 |
| 23 | 6.25E-01 | 1.16E-05 | -2.98E-03 | 2.180 | 2.56 | -0.38 |
| 24 | 6.19E-01 | 2.81E-07 | -1.34E-03 | 2.190 | 2.43 | -0.24 |
| 25 | 4.86E-01 | 4.59E-09 | -1.15E-04 | 2.320 | 3.05 | -0.73 |
| 26 | 7.06E-01 | 4.60E-06 | -1.88E-03 |  | 1.99 |  |
| 27 | 6.65E-01 | 1.72E-06 | -1.58E-03 |  | 2.19 |  |
| 28 | 6.18E-01 | 1.84E-06 | -8.51E-04 |  | 2.38 |  |
| 29 | 6.19E-01 | 9.70E-08 | -1.61E-03 |  | 2.45 |  |
| 30 | 6.96E-01 | 1.81E-04 | -1.31E-03 | 2.130 | 1.98 | 0.15 |
| 31 | 6.75E-01 | 3.04E-07 | -1.56E-03 |  | 2.13 |  |
| 38 | 6.92E-01 | 4.21E-05 | -2.49E-03 |  | 2.13 |  |
| 33 | 6.17E-01 | 1.41E-09 | -9.78E-04 |  | 2.40 |  |
| 34 | 7.10E-01 | 2.38E-07 | -1.50E-03 |  | 1.93 |  |
| 35 | 6.30E-01 | 5.16E-07 | -1.68E-03 | 2.050 | 2.40 | -0.35 |
| 36 | 6.45E-01 | 8.17E-09 | -1.35E-03 | 2.320 | 2.28 | 0.04 |
| 37 | 6.31E-01 | 2.12E-06 | -8.47E-04 |  | 2.31 |  |
| 38 | 6.36E-01 | 1.80E-04 | -1.36E-03 |  | 2.32 |  |
| 39 | 5.68E-01 | 1.75E-04 | -1.36E-03 |  | 2.70 |  |
| 6 | 6.37E-01 | 7.74E-08 | -8.47E-04 |  | 2.27 |  |
| 298# | 6.42E-01 | 2.65E-06 | -2.36E-02 | 3.430 | 4.53 | -1.10 |
| 302* | 6.47E-01 | 6.34E-04 | -1.60E-02 | 3.520 | 3.70 | -0.18 |
| 311* | 6.91E-01 | 1.01E-08 | -1.65E-02 | 3.570 | 3.54 | 0.03 |
| 312* | 6.25E-01 | 7.09E-04 | -1.64E-02 | 3.720 | 3.86 | -0.14 |
| 326* | 6.58E-01 | 1.39E-02 | -1.64E-02 | 2.740 | 2.83 | -0.09 |
| 319* | 6.37E-01 | 1.78E-06 | -1.64E-02 | 4.150 | 3.84 | 0.32 |
| 333 | 6.28E-01 | 1.58E-06 | -1.60E-02 | 3.430 | 3.85 | -0.42 |
| 8* | 6.71E-01 | 1.31E-06 | -1.10E-03 | 2.320 | 2.11 | 0.21 |
| 15* | 6.55E-01 | 5.78E-09 | -1.23E-03 | 2.310 | 2.21 | 0.10 |
| 20* | 5.97E-01 | 1.10E-08 | -1.68E-03 | 2.510 | 2.58 | -0.07 |

**Table S30: Descriptor, experimental and predicted pIC_50_ values and their residuals for test set 1 compounds in cell line based QSAR model against OVCR-3**

| **No.** | **MSA** | **THCMD** | **MaVO** | **Exp.** | **Pred.** | **Res.** |
| --- | --- | --- | --- | --- | --- | --- |
| 326 | 4.27E+02 | 8.10E-01 | 1.97E+00 | 1.840 | 1.63 | 0.21 |
| 328 | 4.19E+02 | -1.00E+00 | 1.97E+00 | 2.330 | 2.22 | 0.11 |
| 329 | 4.17E+02 | 1.85E-01 | 1.96E+00 | 1.770 | 1.85 | -0.08 |
| 337 | 4.16E+02 | -6.30E-01 | 1.97E+00 | 1.820 | 2.12 | -0.30 |
| 334 | 4.10E+02 | -3.27E+00 | 1.96E+00 | 2.190 | 2.92 | -0.73 |
| 340 | 4.12E+02 | -1.38E+00 | 1.97E+00 | 2.380 | 2.37 | 0.01 |
| 341 | 4.17E+02 | -1.80E+00 | 1.97E+00 | 2.580 | 2.47 | 0.11 |
| 342 | 4.44E+02 | -2.00E+00 | 1.97E+00 | 2.310 | 2.39 | -0.08 |
| 312 | 4.86E+02 | -7.21E-01 | 2.11E+00 | 2.150 | 2.11 | 0.04 |
| 303 | 4.49E+02 | -1.19E+00 | 2.11E+00 | 1.730 | 2.44 | -0.71 |
| 320 | 4.73E+02 | -7.11E-01 | 2.10E+00 | 2.370 | 2.15 | 0.22 |
| 305 | 4.46E+02 | -1.50E+00 | 2.10E+00 | 2.770 | 2.53 | 0.24 |
| 297 | 4.69E+02 | -3.54E+00 | 2.11E+00 | 3.300 | 3.04 | 0.26 |
| 336 | 4.06E+02 | -1.11E+00 | 1.97E+00 | 2.690 | 2.32 | 0.37 |
| 335 | 4.07E+02 | -2.38E+00 | 1.97E+00 | 3.160 | 2.70 | 0.47 |
| 316 | 4.31E+02 | 6.45E-02 | 2.10E+00 | 1.790 | 2.14 | -0.35 |
| 302 | 4.28E+02 | -1.33E+00 | 2.10E+00 | 2.660 | 2.57 | 0.09 |
| 296 | 3.55E+02 | -2.73E+00 | 2.18E+00 | 3.660 | 3.56 | 0.10 |
| 338 | 4.18E+02 | -9.51E-01 | 1.97E+00 |  | 2.21 |  |
| 339 | 4.16E+02 | -1.34E+00 | 1.97E+00 |  | 2.34 |  |
| 318 | 4.12E+02 | 6.01E-01 | 2.10E+00 |  | 2.07 |  |
| 319 | 4.35E+02 | -6.67E-01 | 2.10E+00 |  | 2.33 |  |
| 321 | 4.10E+02 | -1.48E+00 | 2.12E+00 |  | 2.76 |  |
| 322 | 4.61E+02 | -1.04E-01 | 2.11E+00 |  | 2.05 |  |
| 323 | 4.55E+02 | -2.51E+00 | 2.12E+00 |  | 2.83 |  |
| 324 | 4.76E+02 | -1.14E+00 | 2.11E+00 |  | 2.28 |  |
| 325 | 5.03E+02 | -1.24E+00 | 2.12E+00 |  | 2.20 |  |
| 300 | 3.32E+02 | -1.11E+00 | 1.97E+00 |  | 2.71 |  |
| 301 | 4.00E+02 | -9.30E-01 | 2.11E+00 |  | 2.62 |  |
| 308 | 4.75E+02 | -1.12E+00 | 2.11E+00 |  | 2.28 |  |
| 309 | 5.05E+02 | -1.03E+00 | 2.10E+00 |  | 2.08 |  |
| 310 | 4.26E+02 | -8.68E-01 | 2.10E+00 |  | 2.44 |  |
| 314 | 4.55E+02 | -9.07E-02 | 2.10E+00 |  | 2.06 |  |
| 327 | 4.30E+02 | -1.28E+00 | 1.97E+00 |  | 2.24 |  |
| 330 | 3.78E+02 | 5.04E-01 | 1.97E+00 |  | 1.98 |  |
| 331 | 4.52E+02 | -1.88E+00 | 1.97E+00 |  | 2.31 |  |
| 306* | 4.28E+02 | -2.38E-01 | 2.10E+00 | 2.710 | 2.24 | 0.47 |
| 311* | 4.50E+02 | -7.75E-01 | 2.10E+00 | 2.740 | 2.29 | 0.45 |
| 313* | 4.55E+02 | -8.46E-01 | 2.10E+00 | 2.740 | 2.28 | 0.46 |
| 315* | 4.26E+02 | -8.63E-01 | 2.10E+00 | 2.710 | 2.44 | 0.27 |
| 317* | 4.18E+02 | -3.41E-01 | 2.10E+00 | 2.810 | 2.33 | 0.49 |
| 307* | 4.53E+02 | -1.47E+00 | 2.10E+00 | 2.740 | 2.48 | 0.26 |
| 332* | 4.21E+02 | -1.41E+00 | 1.97E+00 | 2.820 | 2.33 | 0.49 |
| 333* | 4.25E+02 | -1.63E+00 | 1.97E+00 | 2.730 | 2.38 | 0.36 |
| 298# | 4.89E+02 | -1.59E+00 | 2.12E+00 | 1.130 | 2.37 | -1.24 |
| 299# | 4.70E+02 | -1.68E+00 | 2.13E+00 | 4.070 | 2.52 | 1.55 |

**Table S31: Descriptor, experimental and predicted pIC_50_ values and their residuals for test set 1 compounds in cell line based QSAR model against PC-3**

| **No.** | **ZXS/ZXR** | **MaPCH** | **FS-2PZ** | **Exp.** | **Pred.** | **Res.** |
| --- | --- | --- | --- | --- | --- | --- |
| 192 | 6.25E-01 | 5.37E-02 | -1.86E-01 | 2.000 | 2.45 | -0.45 |
| 161 | 5.65E-01 | 5.37E-02 | -1.81E-01 | 2.310 | 2.63 | -0.32 |
| 163 | 5.56E-01 | 5.37E-02 | -1.92E-01 | 2.920 | 2.68 | 0.24 |
| 198 | 7.19E-01 | 5.37E-02 | -1.79E-01 | 2.590 | 2.14 | 0.45 |
| 196 | 7.15E-01 | 5.37E-02 | -1.90E-01 | 2.360 | 2.18 | 0.19 |
| 195 | 7.29E-01 | 5.37E-02 | -1.85E-01 |  | 2.12 |  |
| 199 | 6.66E-01 | 4.62E-02 | -9.87E-02 | 2.540 | 2.83 | -0.29 |
| 165 | 5.68E-01 | 5.37E-02 | -1.64E-01 | 2.390 | 2.58 | -0.19 |
| 166 | 5.68E-01 | 5.37E-02 | -1.64E-01 | 2.590 | 2.58 | 0.01 |
| 167 | 5.53E-01 | 5.37E-02 | -1.88E-01 | 2.700 | 2.68 | 0.02 |
| 168 | 5.90E-01 | 5.37E-02 | -1.82E-01 | 2.130 | 2.55 | -0.42 |
| 169 | 5.89E-01 | 5.37E-02 | -1.92E-01 | 3.000 | 2.58 | 0.42 |
| 175 | 7.36E-01 | 5.52E-02 | -2.65E-01 |  | 2.14 |  |
| 176 | 7.01E-01 | 5.52E-02 | -2.95E-01 |  | 2.33 |  |
| 178 | 5.41E-01 | 5.64E-02 | -3.99E-01 | 3.100 | 2.96 | 0.14 |
| 179 | 7.11E-01 | 5.63E-02 | -3.71E-01 | 2.120 | 2.37 | -0.25 |
| 180 | 6.18E-01 | 5.89E-02 | -4.07E-01 |  | 2.50 |  |
| 182 | 6.07E-01 | 5.52E-02 | -2.59E-01 | 2.680 | 2.54 | 0.14 |
| 183 | 6.04E-01 | 5.37E-02 | -1.99E-01 | 2.620 | 2.55 | 0.07 |
| 184 | 6.49E-01 | 5.37E-02 | -1.89E-01 | 2.680 | 2.38 | 0.30 |
| 185 | 5.71E-01 | 5.52E-02 | -3.17E-01 | 2.050 | 2.79 | -0.74 |
| 186 | 6.40E-01 | 5.52E-02 | -2.68E-01 |  | 2.45 |  |
| 187 | 6.90E-01 | 4.68E-02 | -1.26E-01 | 2.470 | 2.76 | -0.29 |
| 189 | 6.17E-01 | 6.39E-02 | -2.83E-01 |  | 1.73 |  |
| 190 | 6.11E-01 | 5.74E-02 | -3.29E-01 | 3.050 | 2.48 | 0.57 |
| 191 | 5.98E-01 | 5.84E-02 | -2.78E-01 | 2.600 | 2.30 | 0.30 |
| 194 | 6.81E-01 | 5.83E-02 | -1.98E-01 |  | 1.86 |  |
| 229 | 6.18E-01 | 5.80E-02 | -3.07E-01 |  | 2.35 |  |
| 230 | 6.62E-01 | 5.80E-02 | -2.85E-01 |  | 2.16 |  |
| 231 | 5.42E-01 | 5.83E-02 | -3.04E-01 |  | 2.55 |  |
| 232 | 6.08E-01 | 5.89E-02 | -3.04E-01 | 1.880 | 2.29 | -0.41 |
| 233 | 6.79E-01 | 5.91E-02 | -3.13E-01 |  | 2.07 |  |
| 235 | 7.01E-01 | 3.50E-02 | -1.89E-01 |  | 4.00 |  |
| 236 | 7.46E-01 | 3.50E-02 | -2.32E-01 |  | 3.96 |  |
| 237 | 5.66E-01 | 3.51E-02 | -2.25E-01 |  | 4.51 |  |
| 238 | 7.31E-01 | 3.52E-02 | -2.24E-01 |  | 3.97 |  |
| 239 | 6.92E-01 | 3.51E-02 | -2.37E-01 |  | 4.14 |  |
| 234 | 5.70E-01 | 3.52E-02 | -2.86E-01 | 4.680 | 4.63 | 0.05 |
| 240 | 6.23E-01 | 3.51E-02 | -2.17E-01 | 3.520 | 4.31 | -0.79 |
| 241 | 6.37E-01 | 3.54E-02 | -2.26E-01 |  | 4.26 |  |
| 242 | 8.62E-01 | 3.47E-02 | -1.43E-01 |  | 3.42 |  |
| 243 | 5.39E-01 | 3.57E-02 | -2.31E-01 | 5.300 | 4.55 | 0.75 |
| 245 | 6.37E-01 | 3.67E-02 | -2.83E-01 | 4.620 | 4.27 | 0.35 |
| 246 | 5.29E-01 | 3.66E-02 | -2.25E-01 | 4.890 | 4.48 | 0.41 |
| 247 | 5.29E-01 | 3.66E-02 | -2.25E-01 | 4.160 | 4.48 | -0.32 |
| 248 | 6.39E-01 | 3.62E-02 | -3.67E-01 | 4.250 | 4.51 | -0.26 |
| 249 | 6.34E-01 | 3.59E-02 | -3.05E-01 | 4.520 | 4.41 | 0.12 |
| 257 | 7.80E-01 | 9.76E-02 | -4.36E-01 |  | -1.64 |  |
| 258 | 7.54E-01 | 3.46E-02 | -3.81E-01 |  | 4.33 |  |
| 250 | 6.50E-01 | 3.62E-02 | -4.24E-01 | 4.240 | 4.61 | -0.37 |
| 251 | 5.07E-01 | 3.60E-02 | -3.26E-01 | 4.740 | 4.85 | -0.11 |
| 252 | 6.68E-01 | 3.57E-02 | -2.32E-01 | 4.570 | 4.14 | 0.43 |
| 253 | 5.48E-01 | 3.63E-02 | -2.26E-01 |  | 4.45 |  |
| 254 | 6.94E-01 | 4.15E-02 | -2.39E-01 | 2.580 | 3.52 | -0.94 |
| 255 | 6.91E-01 | 3.73E-02 | -2.84E-01 | 4.770 | 4.04 | 0.73 |
| 256 | 6.97E-01 | 3.35E-02 | -2.04E-01 |  | 4.20 |  |
| 259 | 7.41E-01 | 9.14E-02 | -3.95E-01 |  | -1.02 |  |
| 227 | 6.66E-01 | 5.52E-02 | -2.76E-01 | 2.920 | 2.39 | 0.53 |
| 228 | 5.52E-01 | 5.67E-02 | -3.51E-01 | 2.680 | 2.79 | -0.11 |
| 193* | 5.89E-01 | 5.52E-02 | -2.63E-01 | 2.920 | 2.60 | 0.32 |
| 162* | 5.31E-01 | 5.37E-02 | -1.90E-01 | 2.820 | 2.76 | 0.07 |
| 170* | 6.33E-01 | 5.37E-02 | -1.93E-01 | 2.570 | 2.44 | 0.13 |
| 172* | 6.24E-01 | 5.56E-02 | -2.45E-01 | 2.570 | 2.41 | 0.16 |
| 173* | 6.32E-01 | 5.52E-02 | -2.58E-01 | 2.800 | 2.46 | 0.35 |
| 174* | 6.66E-01 | 5.52E-02 | -2.54E-01 | 2.430 | 2.34 | 0.09 |
| 181* | 5.76E-01 | 5.52E-02 | -2.75E-01 | 2.600 | 2.67 | -0.07 |
| 188* | 5.78E-01 | 5.52E-02 | -2.07E-01 | 2.350 | 2.50 | -0.15 |
| 244* | 6.61E-01 | 3.87E-02 | -2.93E-01 | 4.090 | 4.02 | 0.07 |
| 197* | 7.25E-01 | 5.37E-02 | -1.87E-01 | 2.540 | 2.14 | 0.40 |
| 177* | 6.75E-01 | 5.64E-02 | -3.50E-01 | 2.680 | 2.42 | 0.26 |

**Table S32: Descriptor, experimental and predicted pIC_50_ values and their residuals for test set 1 compounds in cell line based QSAR model against PPC-1**

| **No.** | **PS-1Z** | **RPCGZ** | **MaNACH** | **Exp.** | **Pred.** | **Res.** |
| --- | --- | --- | --- | --- | --- | --- |
| 192 | 2.51E+02 | 5.47E-02 | 4.30E-01 | 3.400 | 3.10 | 0.31 |
| 161 | 1.51E+02 | 4.27E-02 | 4.18E-01 | 3.000 | 3.24 | -0.24 |
| 163 | 1.60E+02 | 4.33E-02 | 4.18E-01 | 3.520 | 3.24 | 0.28 |
| 198 | 1.89E+02 | 7.52E-02 | 4.09E-01 | 3.220 | 2.84 | 0.38 |
| 196 | 2.00E+02 | 8.12E-02 | 4.09E-01 | 2.680 | 2.76 | -0.08 |
| 195 | 1.92E+02 | 8.81E-02 | 4.09E-01 | 1.850 | 2.62 | -0.77 |
| 199 | 1.15E+02 | 6.83E-02 | 3.92E-01 | 3.150 | 3.01 | 0.14 |
| 165 | 1.48E+02 | 4.83E-02 | 4.18E-01 | 3.050 | 3.13 | -0.08 |
| 166 | 1.48E+02 | 4.83E-02 | 4.18E-01 | 3.220 | 3.13 | 0.09 |
| 167 | 1.72E+02 | 4.90E-02 | 4.18E-01 | 3.520 | 3.17 | 0.35 |
| 168 | 1.73E+02 | 5.74E-02 | 4.15E-01 | 2.770 | 3.06 | -0.29 |
| 169 | 1.95E+02 | 5.78E-02 | 4.18E-01 | 3.700 | 3.06 | 0.64 |
| 170 | 1.94E+02 | 5.78E-02 | 4.18E-01 | 3.400 | 3.06 | 0.34 |
| 173 | 2.44E+02 | 5.47E-02 | 4.30E-01 | 3.300 | 3.08 | 0.22 |
| 174 | 2.46E+02 | 5.07E-02 | 4.30E-01 | 3.100 | 3.16 | -0.06 |
| 175 | 2.21E+02 | 7.03E-02 | 4.32E-01 |  | 2.73 |  |
| 176 | 2.49E+02 | 6.99E-02 | 4.30E-01 |  | 2.82 |  |
| 177 | 3.24E+02 | 7.68E-02 | 4.32E-01 | 2.810 | 2.84 | -0.03 |
| 178 | 4.00E+02 | 7.62E-02 | 4.33E-01 | 3.150 | 3.00 | 0.16 |
| 180 | 3.48E+02 | 7.55E-02 | 4.33E-01 |  | 2.90 |  |
| 181 | 2.64E+02 | 5.56E-02 | 4.30E-01 | 2.960 | 3.11 | -0.15 |
| 184 | 2.06E+02 | 6.29E-02 | 4.19E-01 | 3.220 | 2.98 | 0.24 |
| 185 | 2.92E+02 | 5.51E-02 | 4.31E-01 | 2.170 | 3.16 | -0.99 |
| 186 | 2.55E+02 | 7.51E-02 | 4.31E-01 |  | 2.73 |  |
| 187 | 1.45E+02 | 5.54E-02 | 3.92E-01 | 2.600 | 3.30 | -0.70 |
| 188 | 2.05E+02 | 5.37E-02 | 4.30E-01 | 2.620 | 3.02 | -0.40 |
| 189 | 2.76E+02 | 5.72E-02 | 4.44E-01 | 2.960 | 2.94 | 0.02 |
| 190 | 3.13E+02 | 5.82E-02 | 4.35E-01 | 3.400 | 3.11 | 0.29 |
| 191 | 2.57E+02 | 6.38E-02 | 4.37E-01 | 3.300 | 2.87 | 0.44 |
| 194 | 1.45E+02 | 1.25E-01 | 4.40E-01 |  | 1.51 |  |
| 229 | 2.11E+02 | 6.35E-02 | 4.33E-01 |  | 2.82 |  |
| 230 | 1.59E+02 | 4.42E-02 | 4.32E-01 |  | 3.06 |  |
| 232 | 2.10E+02 | 7.25E-02 | 4.32E-01 | 2.470 | 2.67 | -0.20 |
| 233 | 2.21E+02 | 7.61E-02 | 4.33E-01 |  | 2.62 |  |
| 235 | 2.57E+02 | 1.38E-01 | 2.88E-01 |  | 3.28 |  |
| 236 | 2.63E+02 | 1.05E-01 | 2.87E-01 |  | 3.89 |  |
| 237 | 2.53E+02 | 1.05E-01 | 2.88E-01 |  | 3.86 |  |
| 238 | 2.32E+02 | 1.09E-01 | 2.68E-01 |  | 3.97 |  |
| 239 | 2.17E+02 | 8.26E-02 | 2.88E-01 |  | 4.18 |  |
| 241 | 2.42E+02 | 1.33E-01 | 2.90E-01 |  | 3.31 |  |
| 242 | 1.71E+02 | 6.35E-02 | 2.89E-01 |  | 4.41 |  |
| 243 | 1.76E+02 | 6.25E-02 | 2.76E-01 | 5.220 | 4.58 | 0.64 |
| 244 | 1.95E+02 | 6.39E-02 | 2.84E-01 | 4.280 | 4.51 | -0.23 |
| 245 | 1.91E+02 | 6.14E-02 | 2.76E-01 | 4.680 | 4.64 | 0.05 |
| 246 | 1.45E+02 | 4.87E-02 | 2.76E-01 | 5.100 | 4.76 | 0.34 |
| 247 | 1.45E+02 | 4.87E-02 | 2.76E-01 | 4.250 | 4.76 | -0.51 |
| 248 | 2.78E+02 | 6.02E-02 | 2.80E-01 | 4.470 | 4.79 | -0.32 |
| 249 | 2.33E+02 | 6.26E-02 | 2.79E-01 | 4.720 | 4.67 | 0.05 |
| 257 | 3.19E+02 | 9.96E-02 | 5.06E-01 |  | 1.56 |  |
| 258 | 2.80E+02 | 8.28E-02 | 2.81E-01 |  | 4.39 |  |
| 250 | 2.64E+02 | 1.60E-01 | 2.78E-01 | 3.120 | 3.02 | 0.10 |
| 251 | 2.46E+02 | 6.30E-02 | 2.77E-01 | 4.680 | 4.71 | -0.03 |
| 252 | 1.75E+02 | 6.06E-02 | 2.75E-01 | 4.660 | 4.63 | 0.03 |
| 253 | 1.64E+02 | 6.25E-02 | 2.78E-01 |  | 4.54 |  |
| 256 | 1.36E+02 | 7.07E-02 | 2.75E-01 |  | 4.37 |  |
| 259 | 2.97E+02 | 1.22E-01 | 5.11E-01 |  | 1.06 |  |
| 228 | 3.23E+02 | 7.71E-02 | 4.32E-01 | 2.800 | 2.83 | -0.03 |
| 254# | 1.59E+02 | 5.96E-02 | 2.90E-01 | 2.930 | 4.44 | -1.51 |
| 182* | 2.49E+02 | 5.14E-02 | 4.30E-01 | 3.150 | 3.15 | 0.00 |
| 183* | 2.15E+02 | 6.29E-02 | 4.19E-01 | 3.000 | 3.00 | 0.00 |
| 197* | 1.95E+02 | 8.12E-02 | 4.09E-01 | 3.000 | 2.75 | 0.25 |
| 172* | 2.29E+02 | 5.45E-02 | 4.24E-01 | 2.820 | 3.12 | -0.30 |
| 162* | 1.58E+02 | 4.33E-02 | 4.18E-01 | 3.300 | 3.24 | 0.06 |
| 231* | 2.13E+02 | 6.89E-02 | 4.30E-01 | 2.280 | 2.76 | -0.48 |
| 179* | 3.63E+02 | 7.66E-02 | 4.32E-01 | 2.540 | 2.92 | -0.38 |
| 255* | 2.13E+02 | 6.55E-02 | 2.77E-01 | 4.890 | 4.60 | 0.29 |
| 234* | 2.18E+02 | 6.79E-02 | 2.87E-01 | 4.370 | 4.45 | -0.08 |
| 227* | 2.62E+02 | 5.47E-02 | 4.31E-01 | 3.400 | 3.11 | 0.29 |
| 240* | 1.97E+02 | 8.33E-02 | 2.75E-01 | 3.620 | 4.27 | -0.65 |
| 192* | 2.07E+02 | 6.17E-02 | 4.18E-01 | 2.620 | 3.02 | -0.40 |

**Table S33: Descriptor, experimental and predicted pIC_50_ values and their residuals for test set 1 compounds in cell line based QSAR model against RH7777**

| **No.** | **MI-A** | **MaBOC** | **AERN** | **Exp.** | **Pred.** | **Res.** |
| --- | --- | --- | --- | --- | --- | --- |
| 193 | 2.41E-02 | 1.65E+00 | 2.74E-03 |  | 1.68 |  |
| 162 | 9.39E-03 | 1.92E+00 | 3.42E-03 | 2.076 | 1.93 | 0.15 |
| 198 | 1.16E-02 | 1.66E+00 | 3.28E-03 | 2.215 | 2.17 | 0.05 |
| 196 | 1.24E-02 | 1.66E+00 | 3.24E-03 | 2.222 | 2.13 | 0.09 |
| 195 | 1.38E-02 | 1.66E+00 | 3.29E-03 |  | 2.07 |  |
| 167 | 1.97E-02 | 1.65E+00 | 2.93E-03 | 1.765 | 1.85 | -0.09 |
| 168 | 1.75E-02 | 1.65E+00 | 2.65E-03 | 2.444 | 1.97 | 0.48 |
| 171 | 1.59E-02 | 1.66E+00 | 3.34E-03 | 1.879 | 1.98 | -0.10 |
| 172 | 1.26E-02 | 1.68E+00 | 8.13E-04 | 2.310 | 2.27 | 0.04 |
| 173 | 1.75E-02 | 1.68E+00 | 1.30E-03 | 2.036 | 2.02 | 0.01 |
| 174 | 9.89E-03 | 1.69E+00 | 3.64E-03 |  | 2.18 |  |
| 175 | 1.57E-02 | 1.68E+00 | 1.03E-03 |  | 2.12 |  |
| 176 | 6.59E-03 | 1.68E+00 | 7.46E-04 | 2.268 | 2.53 | -0.27 |
| 165 | 1.61E-02 | 1.65E+00 | 2.34E-03 | 2.000 | 2.05 | -0.05 |
| 179 | 1.29E-02 | 1.68E+00 | 1.52E-02 |  | 1.25 |  |
| 180 | 6.03E-03 | 1.92E+00 | 3.51E-03 | 2.119 | 2.07 | 0.05 |
| 187 | 1.69E-02 | 1.68E+00 | 6.94E-04 | 2.174 | 2.09 | 0.08 |
| 185 | 1.88E-02 | 1.73E+00 | 2.44E-03 |  | 1.83 |  |
| 183 | 2.16E-02 | 1.92E+00 | 2.56E-03 | 1.051 | 1.46 | -0.41 |
| 161 | 1.29E-02 | 1.65E+00 | 3.50E-03 | 1.854 | 2.11 | -0.25 |
| 190 | 1.44E-02 | 1.65E+00 | 1.40E-03 |  | 2.19 |  |
| 191 | 1.22E-02 | 1.68E+00 | 1.31E-03 | 2.027 | 2.25 | -0.23 |
| 184 | 1.13E-02 | 1.75E+00 | 3.20E-03 | 1.886 | 2.07 | -0.19 |
| 199 | 1.87E-02 | 1.44E+00 | 1.72E-03 | 2.237 | 2.24 | -0.01 |
| 194 | 1.76E-02 | 1.68E+00 | 9.53E-04 | 2.168 | 2.04 | 0.12 |
| 197 | 1.25E-02 | 1.66E+00 | 3.28E-03 | 2.187 | 2.13 | 0.06 |
| 181 | 3.72E-03 | 1.92E+00 | 3.53E-03 | 2.181 | 2.17 | 0.02 |
| 186 | 2.20E-02 | 1.44E+00 | 9.02E-04 | 2.208 | 2.16 | 0.05 |
| 177 | 7.72E-03 | 1.69E+00 | 1.99E-04 | 2.585 | 2.51 | 0.07 |
| 182 | 1.43E-02 | 1.92E+00 | 2.58E-03 | 2.081 | 1.77 | 0.31 |
| 163* | 1.20E-02 | 1.92E+00 | 3.52E-03 | 2.168 | 1.81 | 0.36 |
| 192* | 1.84E-02 | 1.65E+00 | 1.50E-03 | 2.174 | 2.01 | 0.17 |
| 166* | 1.51E-02 | 1.92E+00 | 2.35E-03 | 2.027 | 1.76 | 0.27 |
| 164* | 1.61E-02 | 1.65E+00 | 2.34E-03 | 1.928 | 2.05 | -0.12 |
| 169* | 2.34E-02 | 1.65E+00 | 2.17E-03 | 2.076 | 1.75 | 0.33 |
| 178* | 8.35E-03 | 1.69E+00 | 3.71E-03 | 2.022 | 2.24 | -0.22 |

**Table S34: Descriptor, experimental and predicted pIC_50_ values and their residuals for test set 1 compounds in cell line based QSAR model against SF-539**

| **No.** | **GI_AP_** | **TPCCMD** | **MaBOO** | **Exp.** | **Pred.** | **Res.** |
| --- | --- | --- | --- | --- | --- | --- |
| 296 | 5.82E+03 | 1.03E+01 | 1.77E+00 | 5.000 | 4.88 | 0.12 |
| 297 | 7.77E+03 | 9.57E+00 | 1.73E+00 | 4.400 | 4.74 | -0.34 |
| 300 | 4.43E+03 | 4.30E+00 | 1.63E+00 |  | 2.02 |  |
| 301 | 6.44E+03 | 6.51E+00 | 1.63E+00 |  | 2.75 |  |
| 302 | 6.76E+03 | 7.18E+00 | 1.62E+00 | 2.570 | 2.78 | -0.21 |
| 303 | 7.31E+03 | 6.63E+00 | 1.63E+00 | 2.360 | 2.98 | -0.62 |
| 306 | 6.76E+03 | 5.54E+00 | 1.62E+00 | 2.370 | 2.59 | -0.22 |
| 307 | 7.71E+03 | 6.80E+00 | 1.62E+00 | 3.310 | 2.96 | 0.35 |
| 308 | 8.30E+03 | 7.99E+00 | 1.63E+00 |  | 3.37 |  |
| 309 | 8.65E+03 | 6.82E+00 | 1.62E+00 |  | 3.19 |  |
| 310 | 6.82E+03 | 6.45E+00 | 1.62E+00 |  | 2.71 |  |
| 311 | 7.29E+03 | 6.94E+00 | 1.62E+00 | 3.310 | 2.88 | 0.44 |
| 312 | 7.71E+03 | 4.05E+00 | 1.62E+00 | 2.710 | 2.65 | 0.06 |
| 314 | 7.13E+03 | 5.37E+00 | 1.62E+00 |  | 2.66 |  |
| 318 | 6.49E+03 | 3.59E+00 | 1.62E+00 |  | 2.30 |  |
| 319 | 7.35E+03 | 4.57E+00 | 1.62E+00 | 3.220 | 2.62 | 0.60 |
| 320 | 8.09E+03 | 8.28E+00 | 1.60E+00 | 2.670 | 2.96 | -0.29 |
| 321 | 6.41E+03 | 7.38E+00 | 1.63E+00 |  | 2.85 |  |
| 322 | 7.68E+03 | 3.78E+00 | 1.62E+00 |  | 2.61 |  |
| 323 | 7.27E+03 | 9.12E+00 | 1.63E+00 |  | 3.25 |  |
| 324 | 7.87E+03 | 5.57E+00 | 1.62E+00 |  | 2.86 |  |
| 325 | 8.05E+03 | 7.14E+00 | 1.62E+00 |  | 3.08 |  |
| 327 | 7.07E+03 | 5.16E+00 | 1.62E+00 |  | 2.62 |  |
| 328 | 7.50E+03 | 4.59E+00 | 1.62E+00 | 2.960 | 2.66 | 0.30 |
| 329 | 6.40E+03 | 1.47E+00 | 1.62E+00 | 2.030 | 2.04 | -0.01 |
| 330 | 5.57E+03 | 1.55E+00 | 1.63E+00 |  | 1.98 |  |
| 331 | 7.35E+03 | 6.25E+00 | 1.63E+00 |  | 2.94 |  |
| 332 | 6.88E+03 | 2.75E+00 | 1.63E+00 | 2.800 | 2.43 | 0.37 |
| 335 | 6.60E+03 | 7.04E+00 | 1.63E+00 | 3.410 | 2.85 | 0.56 |
| 336 | 7.39E+03 | 4.63E+00 | 1.63E+00 | 2.730 | 2.77 | -0.04 |
| 337 | 6.85E+03 | 2.00E+00 | 1.63E+00 | 1.820 | 2.34 | -0.52 |
| 338 | 6.95E+03 | 4.49E+00 | 1.63E+00 |  | 2.65 |  |
| 339 | 6.55E+03 | 4.36E+00 | 1.63E+00 |  | 2.54 |  |
| 341 | 7.09E+03 | 4.36E+00 | 1.63E+00 | 2.710 | 2.67 | 0.05 |
| 316 | 7.34E+03 | 3.56E+00 | 1.62E+00 | 1.930 | 2.50 | -0.57 |
| 298 | 8.37E+03 | 4.12E+00 | 1.73E+00 | 4.400 | 4.26 | 0.14 |
| 305 | 7.65E+03 | 7.25E+00 | 1.62E+00 | 3.090 | 3.00 | 0.09 |
| 334 | 6.32E+03 | 6.33E+00 | 1.62E+00 | 2.360 | 2.57 | -0.21 |
| 315 | 6.64E+03 | 7.53E+00 | 1.62E+00 | 2.730 | 2.79 | -0.06 |
| 299* | 8.11E+03 | 3.02E+00 | 1.72E+00 | 4.430 | 3.94 | 0.49 |
| 340* | 7.09E+03 | 3.54E+00 | 1.63E+00 | 2.820 | 2.57 | 0.25 |
| 317* | 6.63E+03 | 5.08E+00 | 1.63E+00 | 2.870 | 2.64 | 0.23 |
| 326* | 6.98E+03 | 2.51E+00 | 1.63E+00 | 2.190 | 2.43 | -0.24 |
| 333* | 6.90E+03 | 3.38E+00 | 1.63E+00 | 2.900 | 2.51 | 0.39 |
| 313* | 7.95E+03 | 6.05E+00 | 1.62E+00 | 2.940 | 2.93 | 0.01 |

**Table S35: Descriptor, experimental and predicted pIC_50_ values and their residuals for test set 1 compounds in cell line based QSAR model against SN12C**

| **No.** | **NN** | **XYS/XYR** | **MiVC** | **Exp.** | **Pred.** | **Res.** |
| --- | --- | --- | --- | --- | --- | --- |
| 296 | 2.00E+00 | 6.71E-01 | 3.77E+00 | 2.700 | 2.24 | 0.46 |
| 298 | 2.00E+00 | 5.88E-01 | 3.66E+00 | 3.090 | 3.12 | -0.03 |
| 303 | 4.00E+00 | 6.19E-01 | 3.78E+00 | 1.090 | 1.99 | -0.90 |
| 306 | 3.00E+00 | 6.21E-01 | 3.78E+00 | 2.240 | 2.31 | -0.07 |
| 314 | 3.00E+00 | 5.42E-01 | 3.78E+00 |  | 2.98 |  |
| 311 | 3.00E+00 | 5.04E-01 | 3.78E+00 | 3.030 | 3.30 | -0.27 |
| 312 | 4.00E+00 | 6.25E-01 | 3.78E+00 | 1.900 | 1.94 | -0.04 |
| 297 | 2.00E+00 | 6.84E-01 | 3.67E+00 |  | 2.29 |  |
| 333 | 3.00E+00 | 5.85E-01 | 3.82E+00 |  | 2.55 |  |
| 315 | 3.00E+00 | 5.69E-01 | 3.78E+00 | 2.730 | 2.75 | -0.02 |
| 320 | 5.00E+00 | 5.77E-01 | 3.67E+00 | 2.230 | 2.18 | 0.05 |
| 326 | 4.00E+00 | 6.30E-01 | 3.82E+00 | 1.800 | 1.83 | -0.03 |
| 328 | 5.00E+00 | 5.12E-01 | 3.83E+00 | 2.740 | 2.48 | 0.26 |
| 318 | 3.00E+00 | 6.41E-01 | 3.67E+00 |  | 2.31 |  |
| 327 | 3.00E+00 | 6.17E-01 | 3.81E+00 |  | 2.30 |  |
| 334 | 3.00E+00 | 6.03E-01 | 3.82E+00 | 1.990 | 2.40 | -0.41 |
| 335 | 4.00E+00 | 5.81E-01 | 3.81E+00 | 2.810 | 2.26 | 0.55 |
| 336 | 5.00E+00 | 4.95E-01 | 3.83E+00 | 2.680 | 2.62 | 0.06 |
| 337 | 4.00E+00 | 6.09E-01 | 3.82E+00 | 1.720 | 2.01 | -0.29 |
| 340 | 4.00E+00 | 6.38E-01 | 3.82E+00 | 1.860 | 1.76 | 0.10 |
| 332 | 4.00E+00 | 5.88E-01 | 3.82E+00 | 2.750 | 2.19 | 0.56 |
| 342 | 5.00E+00 | 5.29E-01 | 3.82E+00 | 2.220 | 2.35 | -0.13 |
| 317 | 3.00E+00 | 5.76E-01 | 3.67E+00 | 2.720 | 2.87 | -0.15 |
| 319 | 3.00E+00 | 5.84E-01 | 3.67E+00 | 3.100 | 2.80 | 0.30 |
| 329# | 3.00E+00 | 5.82E-01 | 3.81E+00 | 1.690 | 2.60 | -0.91 |
| 302* | 3.00E+00 | 6.06E-01 | 3.78E+00 | 2.600 | 2.44 | 0.16 |
| 305* | 3.00E+00 | 5.81E-01 | 3.78E+00 | 2.590 | 2.65 | -0.06 |
| 307* | 3.00E+00 | 5.96E-01 | 3.78E+00 | 3.030 | 2.52 | 0.51 |
| 313* | 3.00E+00 | 5.30E-01 | 3.78E+00 | 2.950 | 3.08 | -0.13 |
| 316* | 4.00E+00 | 6.13E-01 | 3.67E+00 | 2.630 | 2.21 | 0.42 |
| 341* | 3.00E+00 | 6.20E-01 | 3.82E+00 | 2.430 | 2.26 | 0.17 |

**Table S36: Descriptor, experimental and predicted pIC_50_ values and their residuals for test set 1 compounds in cell line based QSAR model against U937**

| **No.** | **MiBOH** | **MiVN** | **H-HD-2/T** | **Exp.** | **Pred.** | **Res.** |
| --- | --- | --- | --- | --- | --- | --- |
| 75 | 7.14E-01 | 3.12E+00 | 2.14E-02 | 2.319 | 2.56 | -0.24 |
| 76 | 7.37E-01 | 3.12E+00 | 2.82E-02 | 1.815 | 1.70 | 0.12 |
| 78 | 7.68E-01 | 2.95E+00 | 2.94E-02 |  | -0.53 |  |
| 79 | 7.12E-01 | 3.12E+00 | 2.55E-02 |  | 2.39 |  |
| 80 | 7.69E-01 | 3.13E+00 | 2.06E-02 |  | 1.47 |  |
| 82 | 7.66E-01 | 3.12E+00 | 2.79E-02 |  | 1.07 |  |
| 83 | 7.73E-01 | 3.12E+00 | 2.16E-02 |  | 1.25 |  |
| 84 | 7.27E-01 | 3.06E+00 | 3.26E-02 |  | 1.17 |  |
| 87 | 7.14E-01 | 3.07E+00 | 2.69E-02 | 2.022 | 1.84 | 0.18 |
| 88 | 7.14E-01 | 3.07E+00 | 2.67E-02 | 1.854 | 1.85 | 0.00 |
| 89 | 7.14E-01 | 3.06E+00 | 2.65E-02 | 1.678 | 1.77 | -0.10 |
| 90 | 7.14E-01 | 3.12E+00 | 2.19E-02 | 2.699 | 2.53 | 0.17 |
| 91 | 7.14E-01 | 3.12E+00 | 1.93E-02 | 3.000 | 2.67 | 0.33 |
| 92 | 7.14E-01 | 3.12E+00 | 2.04E-02 | 2.569 | 2.61 | -0.04 |
| 93 | 7.13E-01 | 3.12E+00 | 2.00E-02 | 2.337 | 2.65 | -0.32 |
| 94 | 7.37E-01 | 3.12E+00 | 2.47E-02 | 1.921 | 1.88 | 0.04 |
| 95 | 7.29E-01 | 3.12E+00 | 1.80E-02 | 2.553 | 2.40 | 0.15 |
| 96 | 7.29E-01 | 3.11E+00 | 1.81E-02 | 2.187 | 2.31 | -0.13 |
| 97 | 7.30E-01 | 3.12E+00 | 1.75E-02 | 2.569 | 2.41 | 0.16 |
| 98 | 7.28E-01 | 2.97E+00 | 1.57E-02 | 1.229 | 1.25 | -0.02 |
| 99 | 7.35E-01 | 3.12E+00 | 2.37E-02 | 1.658 | 1.98 | -0.32 |
| 100 | 7.36E-01 | 3.12E+00 | 2.00E-02 |  | 2.15 |  |
| 101 | 7.39E-01 | 3.12E+00 | 2.22E-02 |  | 1.97 |  |
| 81* | 7.74E-01 | 3.12E+00 | 2.20E-02 | 1.244 | 1.20 | 0.04 |
| 86* | 7.63E-01 | 3.12E+00 | 1.86E-02 | 1.495 | 1.62 | -0.13 |
| 85* | 7.14E-01 | 3.12E+00 | 1.96E-02 | 2.745 | 2.65 | 0.09 |
| 77* | 7.67E-01 | 3.12E+00 | 2.57E-02 | 1.215 | 1.17 | 0.05 |

**Table S37: Descriptor, experimental and predicted pIC_50_ values and their residuals for test set 1 compounds in cell line based QSAR model against UACC-62**

| **No.** | **MaPCN** | **MaPC** | **MaVO** | **Exp.** | **Pred.** | **Res.** |
| --- | --- | --- | --- | --- | --- | --- |
| 296 | -8.93E-02 | 9.23E-02 | 2.18E+00 | 5.00E+00 | 4.61 | 0.40 |
| 297 | -9.27E-02 | 8.28E-02 | 2.11E+00 | 4.52E+00 | 4.89 | -0.37 |
| 298 | -9.26E-02 | 6.38E-02 | 2.12E+00 | 4.52E+00 | 4.67 | -0.15 |
| 302 | -8.65E-02 | 6.14E-02 | 2.10E+00 | 2.78E+00 | 2.94 | -0.16 |
| 305 | -8.64E-02 | 6.14E-02 | 2.10E+00 | 3.00E+00 | 2.91 | 0.09 |
| 306 | -8.65E-02 | 6.14E-02 | 2.10E+00 | 2.94E+00 | 2.94 | 0.01 |
| 307 | -8.64E-02 | 8.34E-02 | 2.10E+00 | 3.34E+00 | 3.21 | 0.13 |
| 308 | -8.64E-02 | 6.14E-02 | 2.11E+00 |  | 2.98 |  |
| 309 | -8.64E-02 | 6.14E-02 | 2.10E+00 |  | 2.91 |  |
| 310 | -8.67E-02 | 6.14E-02 | 2.10E+00 |  | 2.99 |  |
| 313 | -8.67E-02 | 6.14E-02 | 2.10E+00 | 2.94E+00 | 2.99 | -0.05 |
| 314 | -8.67E-02 | 6.14E-02 | 2.10E+00 |  | 2.99 |  |
| 315 | -8.67E-02 | 6.14E-02 | 2.10E+00 | 2.79E+00 | 2.99 | -0.20 |
| 317 | -8.62E-02 | 8.28E-02 | 2.10E+00 | 3.33E+00 | 3.15 | 0.18 |
| 318 | -8.63E-02 | 6.14E-02 | 2.10E+00 |  | 2.88 |  |
| 319 | -8.62E-02 | 8.34E-02 | 2.10E+00 | 3.46E+00 | 3.16 | 0.30 |
| 320 | -8.63E-02 | 6.14E-02 | 2.10E+00 | 2.47E+00 | 2.88 | -0.41 |
| 327 | -8.61E-02 | 8.34E-02 | 1.97E+00 | 1.79E+00 | 2.27 | -0.48 |
| 328 | -8.61E-02 | 6.14E-02 | 1.97E+00 |  | 1.97 |  |
| 329 | -8.61E-02 | 8.28E-02 | 1.96E+00 | 1.85E+00 | 2.20 | -0.35 |
| 332 | -8.79E-02 | 6.14E-02 | 1.97E+00 | 2.85E+00 | 2.43 | 0.42 |
| 334 | -8.79E-02 | 8.28E-02 | 1.96E+00 | 2.69E+00 | 2.66 | 0.03 |
| 335 | -8.79E-02 | 6.14E-02 | 1.97E+00 | 3.51E+00 | 2.43 | 1.08 |
| 336 | -8.71E-02 | 6.14E-02 | 1.97E+00 | 2.72E+00 | 2.23 | 0.49 |
| 337 | -8.71E-02 | 6.14E-02 | 1.97E+00 | 1.77E+00 | 2.23 | -0.46 |
| 340 | -8.71E-02 | 6.14E-02 | 1.97E+00 | 1.81E+00 | 2.23 | -0.42 |
| 342 | -8.71E-02 | 6.14E-02 | 1.97E+00 | 2.14E+00 | 2.23 | -0.09 |
| 303# | -8.64E-02 | 6.14E-02 | 2.11E+00 | 1.67E+00 | 2.98 | -1.31 |
| 311* | -8.67E-02 | 8.28E-02 | 2.10E+00 | 3.34E+00 | 3.28 | 0.06 |
| 312* | -8.67E-02 | 6.14E-02 | 2.11E+00 | 3.03E+00 | 3.05 | -0.02 |
| 333* | -8.79E-02 | 8.34E-02 | 1.97E+00 | 3.13E+00 | 2.74 | 0.40 |
| 341* | -8.71E-02 | 8.34E-02 | 1.97E+00 | 2.51E+00 | 2.53 | -0.02 |
| 316* | -8.62E-02 | 6.14E-02 | 2.10E+00 | 2.79E+00 | 2.86 | -0.07 |

**Table S38: Descriptor, experimental and predicted pIC_50_ values and their residuals for test set 1 compounds in cell line based QSAR model against WM-164**

| **No.** | **PS-3AZ** | **KHI3** | **MiNRN** | **Exp.** | **Pred.** | **Res.** |
| --- | --- | --- | --- | --- | --- | --- |
| 192 | 1.52E+01 | 7.80E+00 | 1.90E-04 | 2.3565 | 2.45 | -0.10 |
| 193 | 1.53E+01 | 7.73E+00 | 1.03E-04 | 2.9586 | 2.64 | 0.32 |
| 161 | 2.07E+01 | 8.69E+00 | 8.49E-05 | 2.5229 | 2.67 | -0.15 |
| 164 | 1.98E+01 | 8.19E+00 | 8.63E-05 | 3.1549 | 3.00 | 0.16 |
| 198 | 1.17E+01 | 6.30E+00 | 8.01E-04 |  | 2.56 |  |
| 197 | 1.06E+01 | 5.80E+00 | 7.99E-04 |  | 2.86 |  |
| 196 | 1.06E+01 | 5.80E+00 | 8.01E-04 |  | 2.86 |  |
| 195 | 9.80E+00 | 5.30E+00 | 7.96E-04 |  | 3.21 |  |
| 199 | 1.14E+01 | 6.70E+00 | 2.47E-03 |  | 0.11 |  |
| 166 | 1.87E+01 | 8.41E+00 | 1.20E-04 | 2.9208 | 2.55 | 0.37 |
| 169 | 1.63E+01 | 7.59E+00 | 1.84E-04 | 2.9208 | 2.85 | 0.07 |
| 168 | 1.55E+01 | 7.60E+00 | 1.22E-04 | 2.6198 | 2.78 | -0.16 |
| 172 | 1.45E+01 | 7.73E+00 | 3.37E-04 | 2.4089 | 2.22 | 0.19 |
| 173 | 1.50E+01 | 7.71E+00 | 2.46E-04 | 2.9586 | 2.44 | 0.52 |
| 174 | 1.62E+01 | 8.60E+00 | 1.43E-04 | 1.9706 | 1.91 | 0.06 |
| 175 | 1.15E+01 | 8.33E+00 | 5.33E-04 | 0.8945 | 0.89 | 0.01 |
| 176 | 1.08E+01 | 8.44E+00 | 1.25E-04 | 1.1911 | 1.15 | 0.04 |
| 177 | 1.09E+01 | 6.97E+00 | 6.56E-05 | 2.7696 | 2.66 | 0.11 |
| 178 | 1.07E+01 | 6.87E+00 | 5.20E-05 | 2.8539 | 2.74 | 0.11 |
| 179 | 1.09E+01 | 6.16E+00 | 8.48E-04 | 2.3872 | 2.50 | -0.12 |
| 180 | 1.08E+01 | 6.67E+00 | 9.26E-04 |  | 1.90 |  |
| 181 | 1.53E+01 | 7.39E+00 | 7.03E-05 | 2.699 | 3.01 | -0.31 |
| 182 | 1.68E+01 | 8.14E+00 | 7.09E-05 | 2.8861 | 2.54 | 0.34 |
| 183 | 1.46E+01 | 7.46E+00 | 2.26E-04 | 2.6198 | 2.63 | -0.01 |
| 185 | 1.51E+01 | 8.26E+00 | 4.81E-05 | 1.6904 | 2.16 | -0.47 |
| 187 | 1.46E+01 | 7.39E+00 | 4.87E-04 | 2.3098 | 2.39 | -0.08 |
| 188 | 1.56E+01 | 7.82E+00 | 1.25E-04 | 2.4949 | 2.58 | -0.09 |
| 189 | 1.61E+01 | 8.32E+00 | 1.42E-04 | 1.7305 | 2.16 | -0.43 |
| 190 | 1.60E+01 | 8.06E+00 | 1.38E-04 | 2.2441 | 2.40 | -0.16 |
| 191 | 1.72E+01 | 7.68E+00 | 1.04E-04 | 2.7696 | 3.02 | -0.25 |
| 194 | 1.01E+01 | 1.18E+00 | 8.28E-05 |  | 8.11 |  |
| 186# | 1.57E+01 | 7.61E+00 | 2.85E-03 | 1.7328 | -0.48 | 2.22 |
| 163* | 2.04E+01 | 8.34E+00 | 8.64E-05 | 2.9586 | 2.95 | 0.01 |
| 184* | 1.49E+01 | 7.46E+00 | 2.26E-04 | 2.6198 | 2.69 | -0.07 |
| 165* | 1.87E+01 | 8.41E+00 | 1.20E-04 | 2.5686 | 2.55 | 0.02 |
| 167* | 1.86E+01 | 8.06E+00 | 1.19E-04 | 2.9586 | 2.87 | 0.09 |
| 162* | 2.05E+01 | 8.34E+00 | 9.07E-05 | 2.8861 | 2.97 | -0.08 |
| 170* | 1.63E+01 | 7.61E+00 | 1.26E-04 | 3.0000 | 2.90 | 0.10 |

**Table S39: Descriptor, experimental and predicted pIC_50_ values and their residuals for test set 1 compounds in cell line based QSAR model against U373-MG**

| **No.** | **ANRN** | **H-1E** | **MaBON** | **Exp.** | **Pred.** | **Res.** |
| --- | --- | --- | --- | --- | --- | --- |
| 4 | 2.81E-02 | -6.18E+00 | 1.13E+00 | 2.0458 | 2.28 | -0.23 |
| 3 | 4.23E-02 | -6.52E+00 | 1.07E+00 | 2.1249 | 2.00 | 0.12 |
| 5 | 2.80E-02 | -6.29E+00 | 1.15E+00 | 2.3565 | 2.31 | 0.05 |
| 6 | 2.81E-02 | -6.34E+00 | 1.15E+00 |  | 2.32 |  |
| 7 | 2.76E-02 | -6.54E+00 | 1.14E+00 |  | 2.39 |  |
| 8 | 2.81E-02 | -6.17E+00 | 1.14E+00 | 2.3596 | 2.27 | 0.09 |
| 9 | 2.76E-02 | -6.56E+00 | 1.13E+00 |  | 2.40 |  |
| 14 | 2.80E-02 | -6.27E+00 | 1.11E+00 | 2.3768 | 2.31 | 0.07 |
| 15 | 2.05E-02 | -6.82E+00 | 1.11E+00 | 2.4815 | 2.68 | -0.19 |
| 16 | 2.06E-02 | -6.76E+00 | 1.12E+00 | 3.0458 | 2.65 | 0.39 |
| 17 | 2.08E-02 | -6.46E+00 | 1.12E+00 | 2.4685 | 2.56 | -0.09 |
| 18 | 2.08E-02 | -6.46E+00 | 1.13E+00 | 2.3872 | 2.56 | -0.17 |
| 19 | 2.07E-02 | -6.00E+00 | 1.09E+00 | 2.4437 | 2.43 | 0.02 |
| 20 | 2.23E-02 | -5.77E+00 | 1.59E+00 | 2.0458 | 2.22 | -0.17 |
| 21 | 2.07E-02 | -6.52E+00 | 1.10E+00 | 3 | 2.58 | 0.42 |
| 25 | 1.41E-02 | -5.75E+00 | 1.09E+00 | 2.4437 | 2.53 | -0.09 |
| 26 | 2.83E-02 | -5.77E+00 | 1.74E+00 |  | 2.03 |  |
| 27 | 2.84E-02 | -5.83E+00 | 1.74E+00 |  | 2.04 |  |
| 28 | 2.84E-02 | -5.67E+00 | 1.82E+00 | 2.1079 | 1.98 | 0.13 |
| 29 | 2.83E-02 | -6.00E+00 | 1.75E+00 | 2.1938 | 2.10 | 0.10 |
| 30 | 2.10E-02 | -6.38E+00 | 2.86E+00 | 2.3768 | 2.19 | 0.19 |
| 32 | 2.77E-02 | -5.53E+00 | 1.11E+00 |  | 2.10 |  |
| 33 | 2.85E-02 | -5.73E+00 | 1.09E+00 |  | 2.14 |  |
| 34 | 2.11E-02 | -5.61E+00 | 1.51E+00 |  | 2.22 |  |
| 35 | 2.84E-02 | -5.62E+00 | 1.12E+00 | 2.1487 | 2.10 | 0.05 |
| 36 | 2.08E-02 | -5.94E+00 | 1.14E+00 | 2.4437 | 2.40 | 0.05 |
| 37 | 2.07E-02 | -5.68E+00 | 1.20E+00 |  | 2.31 |  |
| 38 | 2.08E-02 | -5.90E+00 | 1.15E+00 |  | 2.39 |  |
| 39 | 1.66E-02 | -6.13E+00 | 2.86E+00 | 2.0269 | 2.23 | -0.21 |
| 23 | 3.94E-03 | -5.64E+00 | 1.10E+00 | 2.8539 | 2.77 | 0.08 |
| 24 | 2.06E-02 | -6.17E+00 | 1.09E+00 | 2.5086 | 2.48 | 0.03 |
| 11 | 2.78E-02 | -6.47E+00 | 1.16E+00 | 2.0506 | 2.36 | -0.31 |
| 12 | 2.77E-02 | -6.59E+00 | 1.17E+00 | 2.0915 | 2.40 | -0.31 |
| 2* | 2.83E-02 | -5.81E+00 | 1.14E+00 | 2.4559 | 2.16 | 0.30 |
| 10* | 2.83E-02 | -6.04E+00 | 1.13E+00 | 2.5686 | 2.23 | 0.34 |
| 31* | 2.11E-02 | -6.11E+00 | 1.74E+00 | 2.301 | 2.32 | -0.02 |
| 22* | 2.06E-02 | -6.30E+00 | 1.09E+00 | 2.6778 | 2.52 | 0.16 |
| 13* | 2.79E-02 | -6.26E+00 | 1.12E+00 | 2.2218 | 2.31 | -0.08 |
| 1* | 2.75E-02 | -7.10E+00 | 1.52E+00 | 2.8861 | 2.49 | 0.40 |

**Table S40: Descriptor, experimental and predicted pIC_50_ values and their residuals for test set 1 compounds in cell line based QSAR model against K562**

| **No.** | **H-HC-1Q** | **RPCSZ** | **MiERC** | **Exp.** | **Pred.** | **Res.** |
| --- | --- | --- | --- | --- | --- | --- |
| 41 | 1.99E+01 | 0.00E+00 | 5.07E-06 | 2.0223 | 2.20 | -0.18 |
| 42 | 2.88E+01 | 4.26E+00 | 1.95E-04 |  | -3.39 |  |
| 43 | 2.83E+01 | 3.87E+00 | 7.22E-05 |  | -0.24 |  |
| 44 | 1.83E+01 | 0.00E+00 | 2.48E-05 |  | 1.65 |  |
| 45 | 1.96E+01 | 1.66E-01 | 2.63E-06 | 2.6021 | 2.20 | 0.40 |
| 46 | 1.41E+01 | 0.00E+00 | 5.28E-06 |  | 2.00 |  |
| 50 | 2.59E+01 | 8.40E-02 | 6.02E-06 | 2.0862 | 2.36 | -0.27 |
| 51 | 2.62E+01 | 1.61E-01 | 1.97E-06 | 2.4089 | 2.45 | -0.04 |
| 52 | 2.63E+01 | 8.39E-02 | 2.72E-06 | 2.9208 | 2.45 | 0.47 |
| 53 | 2.58E+01 | 1.69E-01 | 3.61E-07 |  | 2.47 |  |
| 54 | 2.44E+01 | 1.62E-01 | 3.32E-07 | 2.2291 | 2.43 | -0.20 |
| 55 | 2.81E+01 | 2.36E-01 | 5.46E-06 |  | 2.41 |  |
| 56 | 2.38E+01 | 7.88E-02 | 1.33E-05 | 1.9957 | 2.11 | -0.11 |
| 59 | 1.86E+01 | 0.00E+00 | 3.31E-05 | 1.3768 | 1.46 | -0.08 |
| 61 | 1.89E+01 | 1.98E+00 | 8.96E-07 |  | 1.73 |  |
| 62 | 1.34E+01 | 2.01E+00 | 1.47E-05 |  | 1.19 |  |
| 66 | 1.32E+01 | 1.91E-01 | 6.00E-06 | 1.6253 | 1.90 | -0.27 |
| 67 | 7.00E+00 | 0.00E+00 | 1.51E-07 | 1.6946 | 1.88 | -0.19 |
| 68 | 2.08E+01 | 3.65E+00 | 3.07E-06 |  | 1.28 |  |
| 69 | 3.09E+01 | 2.62E+00 | 4.79E-06 | 1.8297 | 1.87 | -0.04 |
| 70 | 4.40E+01 | 2.65E+00 | 4.84E-06 |  | 2.30 |  |
| 71 | 1.82E+01 | 0.00E+00 | 7.54E-06 | 2.1367 | 2.08 | 0.06 |
| 72 | 2.62E+01 | 1.50E+00 | 5.64E-06 | 1.9245 | 1.99 | -0.07 |
| 73 | 9.33E+00 | 8.53E-01 | 1.07E-06 | 1.7167 | 1.71 | 0.01 |
| 74 | 1.87E+01 | 2.35E+00 | 2.92E-06 | 1.6402 | 1.57 | 0.07 |
| 40 | 1.83E+01 | 8.70E-02 | 3.71E-06 | 1.9318 | 2.16 | -0.22 |
| 60 | 1.73E+01 | 7.56E-02 | 5.84E-06 | 2.3768 | 2.07 | 0.31 |
| 64 | 1.27E+01 | 8.01E-02 | 1.47E-05 | 2.0362 | 1.69 | 0.34 |
| 47* | 1.91E+01 | 0.00E+00 | 5.82E-07 | 2.1249 | 2.28 | -0.16 |
| 48* | 2.02E+01 | 1.80E-01 | 5.96E-07 | 1.8761 | 2.27 | -0.40 |
| 57* | 2.43E+01 | 1.93E-01 | 1.10E-06 | 1.9957 | 2.40 | -0.40 |
| 58* | 1.74E+01 | 1.85E-01 | 1.83E-06 | 1.8386 | 2.14 | -0.31 |
| 65* | 9.40E+00 | 0.00E+00 | 7.85E-06 | 1.8097 | 1.77 | 0.04 |

**Table S41: Descriptor, experimental and predicted pIC_50_ values and their residuals for test set 1 compounds in test set 1 compounds in scaffold based QSAR model S5**

| **No.** | **ACI_2_** | **MiPCO** | **RNAB** | **Exp.** | **Pred.** | **Res.** |
| --- | --- | --- | --- | --- | --- | --- |
| 125 | 7.20E-01 | -1.77E-01 | 0.00E+00 | 1.1871 | 1.11 | 0.08 |
| 126 | 7.69E-01 | -1.67E-01 | 0.00E+00 | 1.1871 | 1.18 | 0.01 |
| 128 | 1.06E+00 | -1.67E-01 | 0.00E+00 | 1.1871 | 1.27 | -0.08 |
| 129 | 8.62E-01 | -1.74E-01 | 2.04E-02 | 1.1367 | 1.18 | -0.04 |
| 131 | 1.17E+00 | -1.74E-01 | 1.72E-02 | 1.3279 | 1.27 | 0.06 |
| 132 | 1.26E+00 | -1.64E-01 | 1.94E-01 | 1.5086 | 1.45 | 0.06 |
| 136 | 1.23E+00 | -1.64E-01 | 1.67E-01 | 1.6021 | 1.42 | 0.18 |
| 137 | 1.19E+00 | -1.82E-01 | 1.73E-01 | 1.1427 | 1.32 | -0.17 |
| 138 | 1.41E+00 | -1.77E-01 | 1.57E-01 | 1.4949 | 1.40 | 0.09 |
| 139 | 7.66E-01 | -1.75E-01 | 5.97E-02 | 1.2366 | 1.17 | 0.07 |
| 140 | 9.00E-01 | -1.75E-01 | 1.11E-01 | 1.1675 | 1.23 | -0.07 |
| 142 | 7.09E-01 | -1.74E-01 | 1.03E-01 | 1.1367 | 1.18 | -0.04 |
| 143 | 8.70E-01 | -1.75E-01 | 1.03E-01 | 1.1079 | 1.22 | -0.11 |
| 144 | 7.75E-01 | -1.72E-01 | 1.30E-01 | 1.3565 | 1.22 | 0.14 |
| 145 | 7.88E-01 | -1.73E-01 | 0.00E+00 | 1.1675 | 1.15 | 0.02 |
| 152 | 9.58E-01 | -1.81E-01 | 0.00E+00 | 1.1192 | 1.16 | -0.04 |
| 149 | 1.16E+00 | -1.81E-01 | 0.00E+00 | 1.1549 | 1.22 | -0.07 |
| 150 | 8.41E-01 | -1.77E-01 | 0.00E+00 | 1.1427 | 1.15 | 0.00 |
| 146 | 7.97E-01 | -1.82E-01 | 0.00E+00 | 1.1675 | 1.10 | 0.06 |
| 153 | 7.57E-01 | -1.75E-01 | 0.00E+00 | 1.1308 | 1.13 | 0.00 |
| 154 | 7.57E-01 | -1.71E-01 | 2.44E-02 | 1.1192 | 1.17 | -0.05 |
| 155 | 7.20E-01 | -1.72E-01 | 0.00E+00 | 1.2147 | 1.14 | 0.08 |
| 156 | 8.38E-01 | -1.75E-01 | 0.00E+00 | 1.1938 | 1.16 | 0.04 |
| 157 | 6.71E-01 | -1.72E-01 | 2.50E-02 | 1.1739 | 1.13 | 0.04 |
| 124 | 8.88E-01 | -1.76E-01 | 0.00E+00 | 1.1549 | 1.17 | -0.01 |
| 135 | 1.02E+00 | -1.63E-01 | 1.73E-01 | 1.2241 | 1.37 | -0.14 |
| 160 | 7.64E-01 | -1.56E-01 | 0.00E+00 | 1.1308 | 1.24 | -0.11 |
| 123* | 6.90E-01 | -1.75E-01 | 0.00E+00 | 1.2218 | 1.11 | 0.11 |
| 151* | 1.32E+00 | -1.81E-01 | 0.00E+00 | 1.1427 | 1.27 | -0.13 |
| 127* | 9.67E-01 | -1.67E-01 | 0.00E+00 | 1.1549 | 1.24 | -0.08 |
| 130* | 9.61E-01 | -1.74E-01 | 1.92E-02 | 1.1675 | 1.21 | -0.04 |
| 147* | 9.68E-01 | -1.81E-01 | 0.00E+00 | 1.1192 | 1.16 | -0.04 |
| 148* | 1.06E+00 | -1.81E-01 | 0.00E+00 | 1.1308 | 1.19 | -0.06 |
| 141* | 7.75E-01 | -1.74E-01 | 1.11E-01 | 1.1549 | 1.20 | -0.05 |
| 158* | 1.09E+00 | -1.76E-01 | 0.00E+00 | 1.1192 | 1.23 | -0.11 |
| 159* | 1.07E+00 | -1.75E-01 | 0.00E+00 | 1.1308 | 1.23 | -0.10 |
| 134# | 1.06E+00 | -1.64E-01 | 1.94E-01 | 1.9208 | 1.39 | 0.53 |

**Table S42: Descriptor, experimental and predicted pIC50 values and their residuals for test set 1 compounds in scaffold based QSAR model S6**

| **No.** | **KHI3** | **MV/X** | **MaPCH** | **Exp.** | **Pred.** | **Res.** |
| --- | --- | --- | --- | --- | --- | --- |
| 192 | 7.80E+00 | 2.53E-01 | 5.37E-02 | 1.81E+00 | 2.00 | -0.19 |
| 193 | 7.73E+00 | 2.35E-01 | 5.52E-02 | 2.66E+00 | 1.99 | 0.67 |
| 161 | 8.69E+00 | 1.89E-01 | 5.37E-02 | 2.23E+00 | 2.38 | -0.15 |
| 162 | 8.34E+00 | 1.79E-01 | 5.37E-02 | 2.33E+00 | 2.55 | -0.22 |
| 163 | 8.34E+00 | 1.85E-01 | 5.37E-02 | 2.49E+00 | 2.50 | -0.01 |
| 198 | 6.30E+00 | 2.40E-01 | 5.37E-02 |  | 2.48 |  |
| 197 | 5.80E+00 | 2.48E-01 | 5.37E-02 |  | 2.52 |  |
| 196 | 5.80E+00 | 2.46E-01 | 5.37E-02 |  | 2.54 |  |
| 195 | 5.30E+00 | 2.60E-01 | 5.37E-02 |  | 2.53 |  |
| 199 | 6.70E+00 | 2.46E-01 | 4.62E-02 |  | 3.29 |  |
| 169 | 7.59E+00 | 2.15E-01 | 5.37E-02 | 2.52E+00 | 2.40 | 0.12 |
| 170 | 7.61E+00 | 2.60E-01 | 5.37E-02 | 2.64E+00 | 1.98 | 0.66 |
| 172 | 7.73E+00 | 2.42E-01 | 5.56E-02 | 2.10E+00 | 1.87 | 0.23 |
| 173 | 7.71E+00 | 2.16E-01 | 5.52E-02 | 2.66E+00 | 2.17 | 0.49 |
| 174 | 8.60E+00 | 2.59E-01 | 5.52E-02 | 1.72E+00 | 1.56 | 0.16 |
| 175 | 8.33E+00 | 2.88E-01 | 5.52E-02 | 1.02E+00 | 1.36 | -0.34 |
| 176 | 8.44E+00 | 3.13E-01 | 5.52E-02 | 9.66E-01 | 1.10 | -0.14 |
| 178 | 6.87E+00 | 2.07E-01 | 5.64E-02 | 2.25E+00 | 2.30 | -0.05 |
| 179 | 6.16E+00 | 2.21E-01 | 5.63E-02 | 2.24E+00 | 2.35 | -0.11 |
| 180 | 6.67E+00 | 2.50E-01 | 5.89E-02 | 1.26E+00 | 1.63 | -0.37 |
| 181 | 7.39E+00 | 1.56E-01 | 5.52E-02 | 2.68E+00 | 2.80 | -0.12 |
| 182 | 8.14E+00 | 1.79E-01 | 5.52E-02 | 2.80E+00 | 2.41 | 0.39 |
| 183 | 7.46E+00 | 2.09E-01 | 5.37E-02 | 2.22E+00 | 2.49 | -0.27 |
| 184 | 7.46E+00 | 2.54E-01 | 5.37E-02 | 2.00E+00 | 2.07 | -0.07 |
| 185 | 8.26E+00 | 2.08E-01 | 5.52E-02 | 1.82E+00 | 2.11 | -0.29 |
| 186 | 7.61E+00 | 2.71E-01 | 5.52E-02 | 1.38E+00 | 1.69 | -0.31 |
| 188 | 7.82E+00 | 2.43E-01 | 5.52E-02 | 1.92E+00 | 1.90 | 0.03 |
| 189 | 8.32E+00 | 2.28E-01 | 6.39E-02 | 9.45E-01 | 0.80 | 0.15 |
| 190 | 8.06E+00 | 2.12E-01 | 5.74E-02 | 1.03E+00 | 1.84 | -0.81 |
| 191 | 7.68E+00 | 2.30E-01 | 5.84E-02 | 2.19E+00 | 1.64 | 0.55 |
| 194 | 1.18E+00 | 3.28E-01 | 5.83E-02 |  | 2.29 |  |
| 165* | 8.41E+00 | 2.05E-01 | 5.37E-02 | 1.84E+00 | 2.30 | -0.46 |
| 167* | 8.06E+00 | 2.04E-01 | 5.37E-02 | 2.49E+00 | 2.39 | 0.10 |
| 168* | 7.60E+00 | 2.35E-01 | 5.37E-02 | 2.37E+00 | 2.21 | 0.16 |
| 166* | 8.41E+00 | 2.05E-01 | 5.37E-02 | 2.48E+00 | 2.30 | 0.18 |
| 177* | 6.97E+00 | 2.50E-01 | 5.64E-02 | 2.41E+00 | 1.88 | 0.53 |
| 187* | 7.39E+00 | 2.77E-01 | 4.68E-02 | 2.33E+00 | 2.76 | -0.43 |

**Table S43: Descriptor, experimental and predicted pIC50 values and their residuals for test set 1 compounds in scaffold based QSAR model S7**

| **No.** | **RNC** | **PS-3AZ** | **HS-1/T** | **Exp.** | **Pred.** | **Res.** |
| --- | --- | --- | --- | --- | --- | --- |
| 200 | 4.74E-01 | 1.29E+01 | 1.82E-01 |  | 2.20 |  |
| 202 | 4.60E-01 | 1.37E+01 | 2.08E-01 | 2.190 | 1.83 | 0.36 |
| 204 | 5.00E-01 | 1.23E+01 | 1.11E-01 | 3.700 | 3.36 | 0.34 |
| 205 | 5.00E-01 | 1.26E+01 | 1.26E-01 | 2.740 | 3.01 | -0.27 |
| 206 | 4.64E-01 | 1.37E+01 | 5.29E-02 | 5.400 | 5.29 | 0.11 |
| 210 | 4.76E-01 | 1.62E+01 | 4.17E-02 | 5.100 | 5.20 | -0.10 |
| 212 | 4.46E-01 | 1.45E+01 | 4.24E-02 | 5.050 | 5.82 | -0.77 |
| 214 | 4.35E-01 | 1.71E+01 | 6.85E-02 | 6.000 | 5.31 | 0.69 |
| 216 | 4.64E-01 | 1.39E+01 | 1.13E-01 | 3.220 | 3.91 | -0.69 |
| 217 | 4.64E-01 | 1.29E+01 | 5.84E-02 |  | 5.20 |  |
| 207 | 4.10E-01 | 1.42E+01 | 9.76E-02 | 6.000 | 5.24 | 0.76 |
| 209 | 4.09E-01 | 1.68E+01 | 8.67E-02 |  | 5.39 |  |
| 211 | 4.27E-01 | 1.72E+01 | 4.10E-02 | 6.000 | 6.08 | -0.08 |
| 213 | 4.00E-01 | 1.60E+01 | 5.03E-02 |  | 6.42 |  |
| 219 | 4.18E-01 | 1.44E+01 | 1.17E-01 | 5.000 | 4.64 | 0.36 |
| 222 | 4.47E-01 | 1.39E+01 | 5.95E-02 |  | 5.44 |  |
| 223 | 4.51E-01 | 1.43E+01 | 5.70E-02 | 5.700 | 5.41 | 0.30 |
| 224 | 4.51E-01 | 1.36E+01 | 6.32E-02 | 5.700 | 5.30 | 0.40 |
| 225 | 4.31E-01 | 1.37E+01 | 6.68E-02 | 5.520 | 5.58 | -0.06 |
| 218 | 4.31E-01 | 1.72E+01 | 6.63E-02 | 5.400 | 5.43 | -0.03 |
| 220 | 4.11E-01 | 1.50E+01 | 1.20E-01 | 4.120 | 4.68 | -0.56 |
| 221 | 4.15E-01 | 1.48E+01 | 1.30E-01 | 3.620 | 4.39 | -0.77 |
| 203# | 4.89E-01 | 1.49E+01 | 1.63E-01 | 4.110 | 2.26 | 1.85 |
| 226* | 4.23E-01 | 1.60E+01 | 1.17E-01 | 3.960 | 4.48 | -0.52 |
| 201* | 5.00E-01 | 1.43E+01 | 1.40E-01 | 2.850 | 2.61 | 0.24 |
| 208* | 4.59E-01 | 1.52E+01 | 3.69E-02 | 6.000 | 5.67 | 0.33 |
| 215* | 3.92E-01 | 1.83E+01 | 8.50E-02 | 6.000 | 5.67 | 0.33 |

**Table S44: Descriptor, experimental and predicted pIC50 values and their residuals for test set 1 compounds in scaffold based QSAR model S8**

| **No.** | **Mi1ERS** | **RNN** | **MaERN** | **Exp.** | **Pred.** | **Res.** |
| --- | --- | --- | --- | --- | --- | --- |
| 229 | 4.27E-03 | 4.17E-02 | 3.96E-03 |  | 3.21 |  |
| 230 | 2.54E-03 | 3.33E-02 | 2.47E-03 |  | 3.91 |  |
| 231 | 8.75E-03 | 4.35E-02 | 2.16E-02 | 1.6421 | 1.69 | -0.05 |
| 233 | -6.89E-03 | 4.55E-02 | 1.45E-02 |  | 3.93 |  |
| 235 | 2.23E-02 | 3.33E-02 | 1.74E-03 |  | 1.44 |  |
| 236 | -1.91E-04 | 2.94E-02 | 3.08E-03 |  | 4.41 |  |
| 237 | -1.73E-03 | 2.94E-02 | 1.68E-03 |  | 4.68 |  |
| 238 | 2.25E-02 | 2.94E-02 | 1.11E-02 | 1.153 | 1.14 | 0.01 |
| 239 | -2.11E-03 | 2.63E-02 | 2.56E-03 |  | 4.83 |  |
| 240 | 3.42E-03 | 2.63E-02 | 9.65E-03 | 3.7696 | 3.78 | -0.01 |
| 241 | 2.31E-02 | 3.33E-02 | 4.54E-04 |  | 1.40 |  |
| 242 | 2.46E-02 | 1.47E-02 | 6.57E-03 | 1.7959 | 1.81 | -0.01 |
| 244 | 1.04E-03 | 2.27E-02 | 6.34E-03 | 4.5229 | 4.42 | 0.11 |
| 247 | 1.14E-03 | 1.92E-02 | 6.35E-03 | 4.4685 | 4.57 | -0.10 |
| 250 | -1.16E-04 | 2.13E-02 | 6.47E-03 | 4.6383 | 4.63 | 0.01 |
| 251 | -8.96E-04 | 2.27E-02 | 6.44E-03 | 4.8861 | 4.66 | 0.23 |
| 253 | -1.00E-03 | 4.65E-02 | 6.81E-03 |  | 3.51 |  |
| 254 | -3.13E-03 | 7.14E-02 | 7.19E-03 | 2.3872 | 2.56 | -0.18 |
| 256 | -6.82E-04 | 2.94E-02 | 6.04E-03 |  | 4.33 |  |
| 227 | 1.35E-02 | 3.70E-02 | 2.46E-03 | 2.6778 | 2.34 | 0.34 |
| 245 | 3.96E-04 | 2.27E-02 | 6.28E-03 | 3.5171 | 4.50 | -0.99 |
| 249 | -2.65E-04 | 4.44E-02 | 7.24E-03 | 4.2291 | 3.50 | 0.73 |
| 228 | 7.77E-04 | 5.56E-02 | 1.00E-03 | 2.6778 | 3.13 | -0.45 |
| 255 | -6.14E-05 | 2.44E-02 | 6.30E-03 | 4.699 | 4.48 | 0.22 |
| 248 | -9.85E-03 | 4.35E-02 | 2.10E-02 | 4.4202 | 4.08 | 0.34 |
| 232 | 2.38E-04 | 4.35E-02 | 3.59E-02 | 1.8665 | 2.07 | -0.20 |
| 234* | -1.03E-03 | 2.38E-02 | 1.49E-03 | 4.5528 | 4.87 | -0.31 |
| 252* | 6.31E-04 | 2.00E-02 | 5.88E-03 | 4.7696 | 4.62 | 0.15 |
| 243* | -7.91E-04 | 2.13E-02 | 6.30E-03 | 4.9586 | 4.72 | 0.24 |
| 246* | 1.14E-03 | 1.92E-02 | 6.35E-03 | 4.4815 | 4.57 | -0.09 |

**Table S45: Descriptor, experimental and predicted pIC50 values and their residuals for test set 1 compounds in scaffold based QSAR model S9**

| **No.** | **ABC** | **H-HD-2/T** | **HE** | **Exp.** | **Pred.** | **Res** |
| --- | --- | --- | --- | --- | --- | --- |
| 261 | 1.00E+00 | 1.77E-02 | -5.25E+00 | 3.0269 | 2.43 | 0.60 |
| 268 | 9.95E-01 | 6.84E-03 | -6.20E+00 | 3.6383 | 3.49 | 0.15 |
| 269 | 9.75E-01 | 7.25E-03 | -5.47E+00 |  | 2.43 |  |
| 273 | 1.03E+00 | 2.17E-02 | -5.35E+00 |  | 3.19 |  |
| 275 | 9.81E-01 | 1.56E-02 | -5.48E+00 |  | 2.08 |  |
| 276 | 9.80E-01 | 2.14E-02 | -5.66E+00 |  | 1.77 |  |
| 277 | 1.02E+00 | 1.12E-02 | -5.38E+00 |  | 3.57 |  |
| 276 | 9.76E-01 | 1.11E-02 | -5.69E+00 |  | 2.33 |  |
| 281 | 9.74E-01 | 7.67E-03 | -5.69E+00 |  | 2.49 |  |
| 283 | 9.76E-01 | 1.09E-02 | -5.50E+00 | 2.3242 | 2.24 | 0.09 |
| 288 | 9.99E-01 | 6.51E-03 | -6.17E+00 | 3.7696 | 3.63 | 0.14 |
| 289 | 1.05E+00 | 1.56E-02 | -5.34E+00 | 3.9202 | 4.22 | -0.30 |
| 290 | 1.00E+00 | 1.32E-02 | -5.98E+00 |  | 3.12 |  |
| 291 | 1.00E+00 | 1.22E-02 | -5.94E+00 |  | 3.17 |  |
| 292 | 1.00E+00 | 1.54E-02 | -5.87E+00 |  | 2.92 |  |
| 293 | 1.00E+00 | 1.46E-02 | -6.03E+00 |  | 3.06 |  |
| 294 | 1.03E+00 | 1.35E-02 | -5.81E+00 |  | 3.97 |  |
| 295 | 1.03E+00 | 1.49E-02 | -5.19E+00 |  | 3.54 |  |
| 280 | 9.75E-01 | 1.25E-02 | -6.22E+00 | 2.4535 | 2.49 | -0.04 |
| 262 | 1.00E+00 | 1.21E-02 | -6.19E+00 | 3.5528 | 3.31 | 0.25 |
| 278 | 9.96E-01 | 1.18E-02 | -6.06E+00 | 2.284 | 3.13 | -0.84 |
| 287 | 9.78E-01 | 7.45E-03 | -5.67E+00 | 1.983 | 2.62 | -0.63 |
| 285 | 9.76E-01 | 1.26E-02 | -5.31E+00 | 2.0223 | 2.02 | 0.00 |
| 267 | 9.96E-01 | 7.32E-03 | -5.24E+00 | 2.6253 | 2.97 | -0.34 |
| 286 | 9.76E-01 | 1.28E-02 | -5.39E+00 | 2.3947 | 2.05 | 0.34 |
| 264 | 1.02E+00 | 1.24E-02 | -6.21E+00 | 4.1487 | 3.94 | 0.21 |
| 274 | 1.00E+00 | 1.89E-02 | -6.24E+00 | 2.2518 | 2.89 | -0.64 |
| 266 | 1.01E+00 | 1.36E-02 | -6.23E+00 | 3.9208 | 3.55 | 0.37 |
| 270 | 9.96E-01 | 6.40E-03 | -5.92E+00 | 3.699 | 3.40 | 0.30 |
| 263 | 1.00E+00 | 1.15E-02 | -6.10E+00 | 3.6778 | 3.30 | 0.38 |
| 284* | 9.95E-01 | 1.21E-02 | -5.57E+00 | 2.5287 | 2.81 | -0.28 |
| 271* | 9.93E-01 | 6.38E-03 | -6.10E+00 | 3.6383 | 3.40 | 0.23 |
| 272# | 1.03E+00 | 1.60E-02 | -5.18E+00 | 2.4486 | 3.47 | -1.02 |
| 282* | 9.95E-01 | 6.53E-03 | -6.19E+00 | 3.6383 | 3.51 | 0.13 |
| 265* | 1.02E+00 | 1.21E-02 | -6.09E+00 | 3.8508 | 3.90 | -0.05 |

**Table S46: Descriptor, experimental and predicted pIC50 values and their residuals for test set 1 compounds in scaffold based QSAR model S10**

| **No.** | **MaBOC** | **TPCCMD** | **MiERO** | **Exp.** | **Pred.** | **Res.** |
| --- | --- | --- | --- | --- | --- | --- |
| 296 | 1.77E+00 | 1.03E+01 | 4.09E-05 | 5 | 5.12 | -0.12 |
| 297 | 1.73E+00 | 9.57E+00 | 7.66E-07 |  | 4.67 |  |
| 298 | 1.73E+00 | 4.12E+00 | 8.54E-07 | 3.8097 | 3.74 | 0.07 |
| 299 | 1.72E+00 | 3.02E+00 | 1.01E-04 |  | 3.25 |  |
| 303 | 1.63E+00 | 6.63E+00 | 1.98E-04 |  | 2.76 |  |
| 306 | 1.62E+00 | 5.54E+00 | 2.09E-04 | 2.6904 | 2.45 | 0.24 |
| 311 | 1.62E+00 | 6.94E+00 | 4.92E-06 | 2.9914 | 3.11 | -0.12 |
| 313 | 1.62E+00 | 6.05E+00 | 3.53E-06 | 3.1192 | 2.96 | 0.16 |
| 316 | 1.62E+00 | 3.56E+00 | 2.17E-04 | 1.2899 | 2.10 | -0.81 |
| 317 | 1.63E+00 | 5.08E+00 | 9.91E-05 | 2.7399 | 2.70 | 0.04 |
| 319 | 1.62E+00 | 4.57E+00 | 1.44E-05 |  | 2.69 |  |
| 320 | 1.60E+00 | 8.28E+00 | 2.07E-04 | 2.5901 | 2.73 | -0.14 |
| 326 | 1.63E+00 | 2.51E+00 | 1.96E-04 | 2.58 | 2.06 | 0.52 |
| 328 | 1.62E+00 | 4.59E+00 | 1.97E-04 | 2.2048 | 2.32 | -0.11 |
| 329 | 1.62E+00 | 1.47E+00 | 4.67E-05 | 1.9136 | 2.09 | -0.18 |
| 332 | 1.63E+00 | 2.75E+00 | 1.55E-04 | 2.5607 | 2.19 | 0.37 |
| 334 | 1.62E+00 | 6.33E+00 | 1.39E-05 |  | 2.99 |  |
| 335 | 1.63E+00 | 7.04E+00 | 1.43E-04 | 3.3188 | 2.95 | 0.37 |
| 336 | 1.63E+00 | 4.63E+00 | 1.81E-04 |  | 2.46 |  |
| 337 | 1.63E+00 | 2.00E+00 | 1.81E-04 | 1.6308 | 2.01 | -0.38 |
| 340 | 1.63E+00 | 3.54E+00 | 1.85E-04 |  | 2.26 |  |
| 341 | 1.63E+00 | 4.36E+00 | 1.04E-05 | 2.4001 | 2.76 | -0.36 |
| 302* | 1.62E+00 | 7.18E+00 | 1.79E-04 | 2.6596 | 2.80 | -0.14 |
| 305* | 1.62E+00 | 7.25E+00 | 1.45E-04 | 3.0315 | 2.88 | 0.16 |
| 307* | 1.62E+00 | 6.80E+00 | 5.31E-05 | 2.9914 | 2.99 | 0.00 |
| 312* | 1.62E+00 | 4.05E+00 | 1.95E-04 | 2.5406 | 2.23 | 0.31 |
| 315* | 1.62E+00 | 7.53E+00 | 1.92E-04 | 2.7399 | 2.83 | -0.09 |
| 333* | 1.63E+00 | 3.38E+00 | 1.36E-05 | 2.7799 | 2.59 | 0.20 |

**Table S47: Analysis of Inter-correlation of the descriptors along with correlation of activity for the test set (R^2^_pred_).**

| **No.** | **R^2^_pred_** | **D1** | **D2** | **D3** |  | **D1** | **D2** | **D3** |
| --- | --- | --- | --- | --- | --- | --- | --- | --- |
|  |  |  |  |  | D1 | 1.000 | 0.233 | -0.219 |
| M1 | 0.98 | MiVH | ZXS/ZXR | MiNRC | D2 | 0.233 | 1.000 | -0.150 |
|  |  |  |  |  | D3 | -0.219 | -0.150 | 1.000 |
|  |  |  |  |  |  |  |  |  |
|  |  |  |  |  | D1 | 1.000 | -0.135 | -0.188 |
| M2 | 0.85 | MaPCH | ZXS/ZXR | Ma1ERN | D2 | -0.135 | 1.000 | 0.199 |
|  |  |  |  |  | D3 | -0.188 | 0.199 | 1.000 |
|  |  |  |  |  |  |  |  |  |
|  |  |  |  |  | D1 | 1.000 | -0.248 | 0.023 |
| M4 | 0.94 | H-2/T_Z_ | Mi1ERN | MiNACH | D2 | -0.248 | 1.000 | 0.263 |
|  |  |  |  |  | D3 | 0.023 | 0.263 | 1.000 |
|  |  |  |  |  |  |  |  |  |
|  |  |  |  |  | D1 | 1.000 | -0.128 | -0.172 |
| M5 | 0.91 | PS3 | KHI3 | MiNRN | D2 | -0.128 | 1.000 | 0.133 |
|  |  |  |  |  | D3 | -0.172 | 0.133 | 1.000 |
|  |  |  |  |  |  |  |  |  |
|  |  |  |  |  | D1 | 1.000 | 0.122 | -0.010 |
| M6 | 0.94 | ZXS/ZXR | MaPCH | FS-2Pz | D2 | 0.122 | 1.000 | 0.054 |
|  |  |  |  |  | D3 | -0.010 | 0.054 | 1.000 |
|  |  |  |  |  |  |  |  |  |
|  |  |  |  |  | D1 | 1.000 | -0.128 | -0.231 |
| M8 | 0.82 | MaPCN | MaPC | MaVO | D2 | -0.128 | 1.000 | 0.198 |
|  |  |  |  |  | D3 | -0.231 | 0.198 | 1.000 |
|  |  |  |  |  |  |  |  |  |
|  |  |  |  |  | D1 | 1.000 | -0.053 | 0.327 |
| M9 | 0.95 | GI_AP_ | TPCCMD | MaBOO | D2 | -0.053 | 1.000 | 0.232 |
|  |  |  |  |  | D3 | 0.327 | 0.232 | 1.000 |
|  |  |  |  |  |  |  |  |  |
|  |  |  |  |  | D1 | 1.000 | 0.363 | 0.177 |
| M10 | 0.97 | ZXS | SIC_0_ | RNO | D2 | 0.363 | 1.000 | -0.123 |
|  |  |  |  |  | D3 | 0.177 | -0.123 | 1.000 |
|  |  |  |  |  |  |  |  |  |
|  |  |  |  |  | D1 | 1.000 | -0.196 | 0.238 |
| M11 | 0.91 | PS-1_Z_ | RPCG_Z_ | MaNACH | D2 | -0.196 | 1.000 | 0.325 |
|  |  |  |  |  | D3 | 0.238 | 0.325 | 1.000 |
|  |  |  |  |  |  |  |  |  |
|  |  |  |  |  | D1 | 1.000 | 0.291 | 0.111 |
| M12 | 0.49 | ZXS/ZXR | TPCCMD | RNCS | D2 | 0.291 | 1.000 | -0.037 |
|  |  |  |  |  | D3 | 0.111 | -0.037 | 1.000 |
|  |  |  |  |  |  |  |  |  |
|  |  |  |  |  |  |  |  |  |
|  |  |  |  |  |  |  |  |  |
|  |  |  |  |  | D1 | 1.000 | -0.144 | -0.125 |
| M15 | 0.79 | YZS | FBCS_Q_ | MaPBO | D2 | -0.144 | 1.000 | -0.194 |
|  |  |  |  |  | D3 | -0.125 | -0.194 | 1.000 |
|  |  |  |  |  |  |  |  |  |
|  |  |  |  |  | D1 | 1.000 | -0.135 | -0.188 |
| M17 | 0.95 | RPCS_Z_ | MiVO | MiBOO | D2 | -0.135 | 1.000 | 0.199 |
|  |  |  |  |  | D3 | -0.188 | 0.199 | 1.000 |
|  |  |  |  |  |  |  |  |  |
|  |  |  |  |  | D1 | 1.000 | -0.342 | -0.306 |
| M19 | 0.76 | ZXS/ZXR | RPCS_Q_ | NF | D2 | -0.342 | 1.000 | -0.363 |
|  |  |  |  |  | D3 | -0.306 | -0.363 | 1.000 |
|  |  |  |  |  |  |  |  |  |
|  |  |  |  |  | D1 | 1.000 | 0.000 | -0.295 |
| M20 | 0.53 | HC-1/T | PP/SD | MaNACC | D2 | 0.000 | 1.000 | -0.347 |
|  |  |  |  |  | D3 | -0.295 | -0.347 | 1.000 |
|  |  |  |  |  |  |  |  |  |
|  |  |  |  |  | D1 | 1.000 | -0.372 | -0.241 |
| M21 | 0.95 | ZXS/ZXR | MiNRO | Mi1ERC | D2 | -0.372 | 1.000 | 0.177 |
|  |  |  |  |  | D3 | -0.241 | 0.177 | 1.000 |
|  |  |  |  |  |  |  |  |  |
|  |  |  |  |  | D1 | 1.000 | -0.410 | -0.072 |
| M23 | 0.61 | NN | XYS/XYR | MiVC | D2 | -0.410 | 1.000 | 0.375 |
|  |  |  |  |  | D3 | -0.072 | 0.375 | 1.000 |
|  |  |  |  |  |  |  |  |  |
|  |  |  |  |  | D1 | 1.000 | -0.005 | 0.411 |
| M26 | 0.09 | MSA | THCMD | MaVO | D2 | -0.005 | 1.000 | -0.040 |
|  |  |  |  |  | D3 | 0.411 | -0.040 | 1.000 |
|  |  |  |  |  |  |  |  |  |
|  |  |  |  |  | D1 | 1.000 | -0.004 | 0.029 |
| M29 | 0.94 | RNO | HS-1_Z_ | H-HC2/ST | D2 | -0.004 | 1.000 | 0.403 |
|  |  |  |  |  | D3 | 0.029 | 0.403 | 1.000 |
|  |  |  |  |  |  |  |  |  |
|  |  |  |  |  | D1 | 1.000 | 0.121 | 0.138 |
| M3 | 0.94 | NN | W-1wP | MiVC | D2 | 0.121 | 1.000 | -0.158 |
|  |  |  |  |  | D3 | 0.138 | -0.158 | 1.000 |
|  |  |  |  |  |  |  |  |  |
|  |  |  |  |  | D1 | 1.000 | 0.102 | -0.050 |
| M7 | 0.99 | MiBOH | MiVN | H-HD-2/T | D2 | 0.102 | 1.000 | 0.103 |
|  |  |  |  |  | D3 | -0.050 | 0.103 | 1.000 |
|  |  |  |  |  |  |  |  |  |
|  |  |  |  |  | D1 | 1.000 | 0.140 | 0.172 |
| M13 | 0.73 | MaVC | A1ERC | MiVO | D2 | 0.140 | 1.000 | -0.066 |
|  |  |  |  |  | D3 | 0.172 | -0.066 | 1.000 |
|  |  |  |  |  |  |  |  |  |
|  |  |  |  |  |  |  |  |  |
|  |  |  |  |  |  |  |  |  |
|  |  |  |  |  | D1 | 1.000 | 0.380 | -0.331 |
| M14 | 0.95 | AVN | MiNACN | MaVC | D2 | 0.380 | 1.000 | -0.078 |
|  |  |  |  |  | D3 | -0.331 | -0.078 | 1.000 |
|  |  |  |  |  |  |  |  |  |
|  |  |  |  |  | D1 | 1.000 | -0.257 | -0.035 |
| M16 | 0.87 | RNH | RNO | MaVO | D2 | -0.257 | 1.000 | 0.083 |
|  |  |  |  |  | D3 | -0.035 | 0.083 | 1.000 |
|  |  |  |  |  |  |  |  |  |
|  |  |  |  |  | D1 | 1.000 | -0.342 | -0.306 |
| M18 | 0.75 | RPCS_Z_ | RNCS_Z_ | MiBOH | D2 | -0.342 | 1.000 | -0.363 |
|  |  |  |  |  | D3 | -0.306 | -0.363 | 1.000 |
|  |  |  |  |  |  |  |  |  |
|  |  |  |  |  | D1 | 1.000 | -0.054 | -0.118 |
| M22 | 0.82 | MaERC | MaBOO | MaVH | D2 | -0.054 | 1.000 | 0.131 |
|  |  |  |  |  | D3 | -0.118 | 0.131 | 1.000 |
|  |  |  |  |  |  |  |  |  |
|  |  |  |  |  | D1 | 1.000 | 0.330 | -0.142 |
| M24 | 0.21 | H-HC-1_Q_ | RPCS_Z_ | MiERC | D2 | 0.330 | 1.000 | -0.201 |
|  |  |  |  |  | D3 | -0.142 | -0.201 | 1.000 |
|  |  |  |  |  |  |  |  |  |
|  |  |  |  |  | D1 | 1.000 | -0.254 | -0.115 |
| M25 | 0.59 | ANRN | H-1E | MaBON | D2 | -0.254 | 1.000 | 0.113 |
|  |  |  |  |  | D3 | -0.115 | 0.113 | 1.000 |
|  |  |  |  |  |  |  |  |  |
|  |  |  |  |  | D1 | 1.000 | -0.084 | -0.273 |
| M27 | 0.87 | MiVN | L1E | THCMD | D2 | -0.084 | 1.000 | 0.269 |
|  |  |  |  |  | D3 | -0.273 | 0.269 | 1.000 |
|  |  |  |  |  |  |  |  |  |
|  |  |  |  |  | D1 | 1.000 | -0.325 | -0.135 |
| M28 | 0.31 | MI-A | MaBOC | AERN | D2 | -0.325 | 1.000 | 0.240 |
|  |  |  |  |  | D3 | -0.135 | 0.240 | 1.000 |
|  |  |  |  |  |  |  |  |  |
|  |  |  |  |  |  |  |  |  |
|  |  |  |  |  | D1 | 1.000 | 0.102 | -0.050 |
| S3 | 0.54 | MiBOH | MiVN | H-HD-2/T | D2 | 0.102 | 1.000 | 0.103 |
|  |  |  |  |  | D3 | -0.050 | 0.103 | 1.000 |
|  |  |  |  |  |  |  |  |  |
|  |  |  |  |  | D1 | 1.000 | 0.000 | -0.295 |
| S4 | 0.26 | HC-1/T | PP/SD | MaNACC | D2 | 0.000 | 1.000 | -0.347 |
|  |  |  |  |  | D3 | -0.295 | -0.347 | 1.000 |
|  |  |  |  |  |  |  |  |  |
|  |  |  |  |  |  |  |  |  |
|  |  |  |  |  |  |  |  |  |
|  |  |  |  |  | D1 | 1.000 | 0.263 | 0.220 |
| S7 | 0.95 | RNC | PS-3A_Z_ | HS-1/T | D2 | 0.263 | 1.000 | 0.275 |
|  |  |  |  |  | D3 | 0.220 | 0.275 | 1.000 |
|  |  |  |  |  |  |  |  |  |
|  |  |  |  |  |  |  |  |  |
|  |  |  |  |  | D1 | 1.000 | -0.249 | 0.029 |
| S8 | 0.23 | Mi1ERS | RNN | MaERN | D2 | -0.249 | 1.000 | 0.198 |
|  |  |  |  |  | D3 | 0.029 | 0.198 | 1.000 |
|  |  |  |  |  |  |  |  |  |
|  |  |  |  |  | D1 | 1.000 | 0.407 | 0.016 |
| S9 | 0.95 | ABC | H-HD-2/T | HE | D2 | 0.407 | 1.000 | 0.243 |
|  |  |  |  |  | D3 | 0.016 | 0.243 | 1.000 |
|  |  |  |  |  |  |  |  |  |
|  |  |  |  |  | D1 | 1.000 | 0.343 | -0.382 |
| S10 | 0.77 | MaBOC | TPCCMD | MiERO | D2 | 0.343 | 1.000 | -0.122 |
|  |  |  |  |  | D3 | -0.382 | -0.122 | 1.000 |
|  |  |  |  |  |  |  |  |  |
|  |  |  |  |  | D1 | 1.000 | 0.140 | 0.172 |
| S1 | 0.73 | MaVC | A1ERC | MiVO | D2 | 0.140 | 1.000 | -0.066 |
|  |  |  |  |  | D3 | 0.172 | -0.066 | 1.000 |
|  |  |  |  |  |  |  |  |  |
|  |  |  |  |  | D1 | 1.000 | 0.330 | -0.142 |
| S2 | 0.21 | H-HDC-1 | RPCS_Z_ | MiERC | D2 | 0.330 | 1.000 | -0.201 |
|  |  |  |  |  | D3 | -0.142 | -0.201 | 1.000 |
|  |  |  |  |  |  |  |  |  |
|  |  |  |  |  | D1 | 1.000 | -0.165 | 0.393 |
| S5 | 0.94 | ACI_2_ | MiPCO | RNAB | D2 | -0.165 | 1.000 | 0.167 |
|  |  |  |  |  | D3 | 0.393 | 0.167 | 1.000 |
|  |  |  |  |  |  |  |  |  |
|  |  |  |  |  | D1 | 1.000 | 0.371 | 0.133 |
| S6 | 0.85 | KHI_3_ | MV/X | MaPCH | D2 | 0.371 | 1.000 | -0.213 |
|  |  |  |  |  | D3 | 0.133 | -0.213 | 1.000 |

**Table S48: Descriptors, experimental and predicted pIC_50_ values and their residuals for test set 2 compounds in cell line based QSAR model against A375**

| **No.** | **MiVH** | **ZXS/ZXR** | **MiNRC** | **Exp.** | **Pred.** | **Res.** |
| --- | --- | --- | --- | --- | --- | --- |
| 191 | 8.29E-01 | 6.25E-01 | 0.00E+00 | 1.824 | 2.31 | -0.48 |
| 197 | 8.35E-01 | 7.19E-01 | 1.22E-08 |  | 1.90 |  |
| 196 | 8.35E-01 | 7.25E-01 | 5.03E-08 |  | 1.86 |  |
| 195 | 8.35E-01 | 7.15E-01 | 5.11E-08 |  | 1.92 |  |
| 194 | 8.35E-01 | 7.29E-01 | 2.09E-07 |  | 1.84 |  |
| 198 | 8.49E-01 | 6.66E-01 | 2.12E-10 |  | 2.49 |  |
| 165 | 8.30E-01 | 5.68E-01 | 0.00E+00 | 2.161 | 2.65 | -0.49 |
| 172 | 8.27E-01 | 6.24E-01 | 2.87E-10 | 2.032 | 2.27 | -0.24 |
| 173 | 8.19E-01 | 6.32E-01 | 1.73E-10 | 2.824 | 2.06 | 0.77 |
| 174 | 8.19E-01 | 6.66E-01 | 0.00E+00 | 1.686 | 1.86 | -0.18 |
| 177 | 8.17E-01 | 6.75E-01 | 5.37E-08 | 2.678 | 1.77 | 0.91 |
| 182 | 8.19E-01 | 6.07E-01 | 0.00E+00 | 2.569 | 2.20 | 0.37 |
| 186 | 8.18E-01 | 6.40E-01 | 0.00E+00 | 1.553 | 1.99 | -0.44 |
| 187 | 8.49E-01 | 6.90E-01 | 1.08E-09 | 1.886 | 2.36 | -0.47 |
| 188 | 8.19E-01 | 5.78E-01 | 2.26E-10 | 1.842 | 2.36 | -0.52 |
| 189 | 8.05E-01 | 6.17E-01 | 4.45E-11 | 1.296 | 1.85 | -0.55 |
| 190 | 8.15E-01 | 6.11E-01 | 4.37E-11 | 1.69 | 2.09 | -0.40 |
| 195 | 8.09E-01 | 6.81E-01 | 5.44E-04 |  | -3.64 |  |
| 229 | 8.17E-01 | 6.18E-01 | 1.39E-07 |  | 2.09 |  |
| 230 | 8.17E-01 | 6.62E-01 | 8.08E-08 |  | 1.84 |  |
| 231 | 8.19E-01 | 5.42E-01 | 4.76E-05 | 1.642 | 2.11 | -0.47 |
| 233 | 8.16E-01 | 6.79E-01 | 2.82E-05 |  | 1.46 |  |
| 235 | 9.19E-01 | 7.01E-01 | 1.47E-05 |  | 3.62 |  |
| 236 | 9.20E-01 | 7.46E-01 | 6.04E-07 |  | 3.52 |  |
| 237 | 9.19E-01 | 5.66E-01 | 2.86E-05 |  | 4.25 |  |
| 239 | 9.19E-01 | 6.92E-01 | 1.17E-05 |  | 3.70 |  |
| 241 | 9.18E-01 | 6.37E-01 | 5.60E-05 |  | 3.57 |  |
| 244 | 9.24E-01 | 6.61E-01 | 6.51E-07 | 4.523 | 4.09 | 0.44 |
| 242 | 9.19E-01 | 8.62E-01 | 1.38E-10 | 1.796 | 2.84 | -1.05 |
| 248 | 9.26E-01 | 6.39E-01 | 1.39E-05 | 4.42 | 4.13 | 0.29 |
| 257 | 7.48E-01 | 7.80E-01 | 8.13E-06 |  | -0.35 |  |
| 250 | 9.27E-01 | 6.50E-01 | 7.91E-06 | 4.638 | 4.14 | 0.50 |
| 252 | 9.28E-01 | 6.68E-01 | 1.45E-06 | 4.77 | 4.12 | 0.65 |
| 253 | 9.26E-01 | 5.48E-01 | 7.45E-06 |  | 4.71 |  |
| 255 | 9.27E-01 | 6.91E-01 | 3.64E-07 | 4.699 | 3.98 | 0.72 |
| 256 | 9.28E-01 | 6.97E-01 | 1.28E-06 |  | 3.96 |  |
| 259 | 7.40E-01 | 7.41E-01 | 3.29E-04 |  | -3.37 |  |
| 227 | 8.18E-01 | 6.66E-01 | 3.39E-10 | 2.678 | 1.84 | 0.84 |
| 163 | 8.30E-01 | 5.56E-01 | 0.00E+00 | 2.745 | 2.72 | 0.03 |
| 246 | 9.27E-01 | 5.29E-01 | 8.59E-08 | 4.482 | 4.91 | -0.42 |
| 183 | 8.29E-01 | 6.04E-01 | 0.00E+00 | 2.31 | 2.43 | -0.12 |
| 238 | 9.29E-01 | 7.31E-01 | 2.37E-04 | 1.153 | 1.53 | -0.38 |
| 232 | 8.17E-01 | 6.08E-01 | 8.84E-06 | 1.867 | 2.07 | -0.20 |
| 161 | 8.30E-01 | 5.65E-01 | 0.00E+00 | 2.337 | 2.67 | -0.33 |
| 179 | 8.18E-01 | 7.11E-01 | 4.04E-05 | 2.208 | 1.20 | 1.01 |
| 234 | 9.20E-01 | 5.70E-01 | 1.57E-05 | 4.553 | 4.38 | 0.18 |
| 184 | 8.29E-01 | 6.49E-01 | 0.00E+00 | 2.276 | 2.17 | 0.11 |
| 228 | 8.17E-01 | 5.52E-01 | 7.54E-07 | 2.678 | 2.46 | 0.22 |
| 168 | 8.32E-01 | 5.90E-01 | 9.16E-11 | 2.523 | 2.57 | -0.05 |
| 178 | 8.16E-01 | 5.41E-01 | 1.33E-06 | 2.509 | 2.50 | 0.01 |
| 169 | 8.30E-01 | 5.89E-01 | 4.49E-11 | 2.745 | 2.53 | 0.21 |
| 181 | 8.19E-01 | 5.76E-01 | 0.00E+00 | 2.721 | 2.38 | 0.35 |
| 251 | 9.27E-01 | 5.07E-01 | 2.27E-06 | 4.886 | 5.01 | -0.12 |
| 166 | 8.30E-01 | 5.68E-01 | 0.00E+00 | 2.796 | 2.65 | 0.15 |
| 243 | 9.28E-01 | 5.39E-01 | 1.59E-06 | 4.959 | 4.85 | 0.11 |
| 167 | 8.30E-01 | 5.53E-01 | 0.00E+00 | 2.854 | 2.74 | 0.12 |
| 162 | 8.30E-01 | 5.31E-01 | 0.00E+00 | 2.62 | 2.86 | -0.24 |
| 180 | 8.17E-01 | 6.18E-01 | 1.30E-04 | 1.338 | 0.85 | 0.49 |
| 245 | 9.27E-01 | 6.37E-01 | 1.46E-06 | 3.517 | 4.28 | -0.76 |
| 175 | 8.17E-01 | 7.36E-01 | 3.49E-06 | 0.862 | 1.39 | -0.53 |
| 192* | 8.19E-01 | 5.89E-01 | 1.49E-11 | 2.678 | 2.30 | 0.38 |
| 170* | 8.29E-01 | 6.33E-01 | 3.10E-11 | 2.824 | 2.26 | 0.56 |
| 176* | 8.19E-01 | 7.01E-01 | 6.08E-08 | 1.181 | 1.66 | -0.48 |
| 185* | 8.18E-01 | 5.71E-01 | 4.40E-11 | 1.9 | 2.38 | -0.48 |
| 247* | 9.27E-01 | 5.29E-01 | 8.59E-08 | 4.469 | 4.91 | -0.44 |
| 191* | 8.12E-01 | 5.98E-01 | 2.98E-11 | 2.444 | 2.10 | 0.34 |
| 249* | 9.26E-01 | 6.34E-01 | 3.10E-05 | 4.229 | 3.99 | 0.24 |
| 240* | 9.26E-01 | 6.23E-01 | 3.96E-05 | 3.77 | 3.97 | -0.20 |
| 254# | 9.19E-01 | 6.94E-01 | 2.46E-05 | 2.387 | 3.56 | -1.18 |

**Table S49: Descriptor, experimental and predicted pIC_50_ values and their residuals for test set 2 compounds in cell line based QSAR model against A549**

| **No.** | **RPCS Z** | **MiVO** | **MiBOO** | **Exp.** | **Pred.** | **Res.** |
| --- | --- | --- | --- | --- | --- | --- |
| 4 | 2.56E-01 | 1.99E+00 | 1.23E-01 |  | 2.41 |  |
| 1 | 3.05E-01 | 2.01E+00 | 1.27E-01 | 2.62 | 2.33 | 0.29 |
| 3 | 4.26E-01 | 2.00E+00 | 1.26E-01 | 2.252 | 2.30 | -0.05 |
| 5 | 2.60E-01 | 2.00E+00 | 1.24E-01 | 2.409 | 2.38 | 0.03 |
| 6 | 1.64E-01 | 1.98E+00 | 1.25E-01 |  | 2.50 |  |
| 7 | 2.77E-01 | 1.99E+00 | 1.25E-01 |  | 2.41 |  |
| 9 | 2.26E-01 | 2.00E+00 | 1.25E-01 |  | 2.40 |  |
| 10 | 1.99E-01 | 1.99E+00 | 1.24E-01 | 2.495 | 2.44 | 0.05 |
| 14 | 4.11E-01 | 1.98E+00 | 1.18E-01 | 2.229 | 2.36 | -0.13 |
| 15 | 2.93E-01 | 1.96E+00 | 1.05E-01 | 2.387 | 2.46 | -0.07 |
| 16 | 0.00E+00 | 2.00E+00 | 1.24E-01 | 3.046 | 2.50 | 0.55 |
| 18 | 7.22E-02 | 1.99E+00 | 1.08E-01 | 2.31 | 2.46 | -0.15 |
| 22 | 3.02E-01 | 1.96E+00 | 1.19E-01 | 2.796 | 2.49 | 0.31 |
| 23 | 2.86E-01 | 1.96E+00 | 1.18E-01 | 2.26 | 2.49 | -0.23 |
| 26 | 2.52E-01 | 2.00E+00 | 1.25E-01 |  | 2.38 |  |
| 27 | 2.26E+00 | 1.92E+00 | 1.25E-01 |  | 1.72 |  |
| 28 | 2.29E+00 | 1.91E+00 | 1.25E-01 | 2.041 | 1.75 | 0.30 |
| 30 | 1.31E-01 | 2.00E+00 | 1.25E-01 | 2.114 | 2.44 | -0.33 |
| 31 | 2.25E-01 | 2.00E+00 | 1.25E-01 |  | 2.40 |  |
| 33 | 1.21E+00 | 1.78E+00 | 1.24E-01 |  | 2.71 |  |
| 34 | 2.18E-01 | 2.00E+00 | 1.24E-01 |  | 2.40 |  |
| 37 | 2.62E-01 | 2.00E+00 | 1.25E-01 |  | 2.38 |  |
| 38 | 1.66E-01 | 2.00E+00 | 1.25E-01 |  | 2.42 |  |
| 39 | 1.69E-01 | 2.00E+00 | 1.25E-01 |  | 2.42 |  |
| 103 | 1.80E-01 | 1.96E+00 | 1.24E-01 | 2.925 | 2.56 | 0.37 |
| 107 | 1.82E+00 | 2.09E+00 | 9.02E-01 | 3.444 | 3.30 | 0.15 |
| 110 | 1.91E+00 | 2.02E+00 | 9.01E-01 | 3.959 | 3.50 | 0.46 |
| 111 | 5.30E-02 | 2.03E+00 | 1.65E-01 | 2.86 | 2.47 | 0.39 |
| 112 | 8.64E-01 | 1.61E+00 | 1.40E-01 | 2.994 | 3.52 | -0.53 |
| 113 | 0.00E+00 | 2.02E+00 | 1.62E-01 | 2.731 | 2.53 | 0.21 |
| 115 | 1.75E+00 | 2.09E+00 | 9.02E-01 | 3.31 | 3.33 | -0.02 |
| 116 | 2.02E+00 | 1.79E+00 | 7.66E-01 | 4.097 | 3.92 | 0.18 |
| 118 | 2.57E+00 | 1.78E+00 | 7.66E-01 | 3.886 | 3.70 | 0.19 |
| 120 | 5.81E-02 | 1.96E+00 | 1.24E-01 | 3.252 | 2.61 | 0.64 |
| 122 | 2.03E-01 | 2.05E+00 | 8.99E-01 | 4.658 | 4.19 | 0.47 |
| 105 | 1.85E+00 | 2.09E+00 | 9.02E-01 | 2.056 | 3.28 | -1.22 |
| 8 | 2.19E-01 | 1.99E+00 | 1.24E-01 | 2.328 | 2.43 | -0.10 |
| 29 | 1.67E-01 | 2.00E+00 | 1.25E-01 | 2.174 | 2.42 | -0.25 |
| 36 | 0.00E+00 | 2.00E+00 | 1.25E-01 | 2.367 | 2.50 | -0.14 |
| 20 | 1.80E-01 | 1.99E+00 | 1.02E-01 | 2.456 | 2.40 | 0.06 |
| 21 | 1.73E-01 | 1.95E+00 | 1.18E-01 | 2.523 | 2.58 | -0.06 |
| 121 | 2.65E+00 | 1.78E+00 | 7.62E-01 | 3.745 | 3.65 | 0.09 |
| 25 | 2.45E-01 | 1.97E+00 | 1.17E-01 | 2.444 | 2.47 | -0.03 |
| 119 | 2.74E+00 | 1.78E+00 | 7.66E-01 | 3.745 | 3.62 | 0.13 |
| 117 | 1.93E+00 | 2.03E+00 | 9.02E-01 | 3.097 | 3.46 | -0.36 |
| 104 | 0.00E+00 | 1.96E+00 | 1.24E-01 | 1.836 | 2.64 | -0.81 |
| 11 | 0.00E+00 | 2.00E+00 | 1.24E-01 | 2 | 2.50 | -0.50 |
| 12 | 0.00E+00 | 2.00E+00 | 1.24E-01 | 2.022 | 2.50 | -0.48 |
| 17 | 0.00E+00 | 1.99E+00 | 1.25E-01 | 2.699 | 2.54 | 0.16 |
| 114 | 1.11E-01 | 2.02E+00 | 1.56E-01 | 2.886 | 2.46 | 0.43 |
| 109* | 2.38E-01 | 2.00E+00 | 1.24E-01 | 2.071 | 2.39 | -0.32 |
| 35* | 1.88E-01 | 2.00E+00 | 1.24E-01 | 2.013 | 2.41 | -0.40 |
| 102* | 1.87E+00 | 2.09E+00 | 9.02E-01 | 3.208 | 3.27 | -0.06 |
| 106* | 1.20E-01 | 1.96E+00 | 1.24E-01 | 3.056 | 2.59 | 0.47 |
| 32* | 2.90E-01 | 2.00E+00 | 1.24E-01 | 2.071 | 2.36 | -0.29 |
| 2* | 3.71E-01 | 2.00E+00 | 1.24E-01 | 2.42 | 2.33 | 0.10 |
| 13* | 1.95E+00 | 2.09E+00 | 9.01E-01 | 3.056 | 3.23 | -0.18 |
| 12# | 0.00E+00 | 2.09E+00 | 9.01E-01 | 2.182 | 4.15 | -1.97 |
| 24* | 0.00E+00 | 1.96E+00 | 1.20E-01 | 2.509 | 2.63 | -0.12 |
| 19* | 2.92E-01 | 1.96E+00 | 1.20E-01 | 2.721 | 2.49 | 0.23 |

**Table S50: Descriptor, experimental and predicted pIC_50_ values and their residuals for test set 2 compounds in cell line based QSAR model against B16-F1**

| **No.** | **MaPCH** | **ZXS/ZXR** | **Mi1ERN** | **Exp.** | **Pred.** | **Res.** |
| --- | --- | --- | --- | --- | --- | --- |
| 165 | 5.37E-02 | 5.68E-01 | 5.18E-05 | 1.845 | 2.62 | -0.77 |
| 198 | 5.37E-02 | 7.19E-01 | 4.21E-05 |  | 1.91 |  |
| 197 | 5.37E-02 | 7.25E-01 | 4.21E-05 |  | 1.88 |  |
| 196 | 5.37E-02 | 7.15E-01 | 3.39E-05 |  | 1.93 |  |
| 195 | 5.37E-02 | 7.29E-01 | 4.26E-05 |  | 1.86 |  |
| 199 | 4.62E-02 | 6.66E-01 | -2.78E-03 |  | 3.07 |  |
| 173 | 5.52E-02 | 6.32E-01 | 2.42E-03 | 2.658 | 1.98 | 0.68 |
| 176 | 5.52E-02 | 7.01E-01 | 3.75E-03 | 0.966 | 1.54 | -0.57 |
| 177 | 5.64E-02 | 6.75E-01 | 2.06E-03 | 2.409 | 1.70 | 0.71 |
| 179 | 5.63E-02 | 7.11E-01 | 1.36E-03 | 2.244 | 1.60 | 0.65 |
| 182 | 5.52E-02 | 6.07E-01 | 8.57E-04 | 2.796 | 2.23 | 0.57 |
| 186 | 5.52E-02 | 6.40E-01 | 1.70E-03 | 1.378 | 2.00 | -0.62 |
| 187 | 4.68E-02 | 6.90E-01 | 2.64E-03 | 2.328 | 2.43 | -0.10 |
| 188 | 5.52E-02 | 5.78E-01 | 8.97E-04 | 1.925 | 2.36 | -0.44 |
| 189 | 6.39E-02 | 6.17E-01 | 1.65E-03 | 0.945 | 1.34 | -0.39 |
| 190 | 5.74E-02 | 6.11E-01 | 6.54E-03 | 1.03 | 1.52 | -0.49 |
| 191 | 5.84E-02 | 5.98E-01 | 6.88E-03 | 2.187 | 1.47 | 0.72 |
| 194 | 5.83E-02 | 6.81E-01 | 2.16E-02 |  | -0.19 |  |
| 229 | 5.80E-02 | 6.18E-01 | 1.38E-03 |  | 1.88 |  |
| 230 | 5.80E-02 | 6.62E-01 | 1.26E-03 |  | 1.69 |  |
| 231 | 5.83E-02 | 5.42E-01 | 2.50E-03 | 1.417 | 2.12 | -0.70 |
| 233 | 5.91E-02 | 6.79E-01 | 1.16E-03 |  | 1.52 |  |
| 235 | 3.50E-02 | 7.01E-01 | 2.70E-03 |  | 3.43 |  |
| 236 | 3.50E-02 | 7.46E-01 | -1.57E-03 |  | 3.58 |  |
| 239 | 3.51E-02 | 6.92E-01 | -1.81E-03 |  | 3.85 |  |
| 234 | 3.52E-02 | 5.70E-01 | 5.99E-04 | 4.26 | 4.20 | 0.06 |
| 240 | 3.51E-02 | 6.23E-01 | 8.49E-04 | 3.456 | 3.94 | -0.49 |
| 241 | 3.54E-02 | 6.37E-01 | 2.24E-03 |  | 3.73 |  |
| 243 | 3.57E-02 | 5.39E-01 | -2.77E-03 | 4.678 | 4.60 | 0.08 |
| 244 | 3.87E-02 | 6.61E-01 | -3.14E-03 | 4.569 | 3.79 | 0.78 |
| 245 | 3.67E-02 | 6.37E-01 | -2.89E-03 | 3.542 | 4.06 | -0.52 |
| 247 | 3.66E-02 | 5.29E-01 | 4.09E-03 | 3.793 | 3.97 | -0.18 |
| 248 | 3.62E-02 | 6.39E-01 | 8.07E-04 | 4.252 | 3.77 | 0.48 |
| 257 | 9.76E-02 | 7.80E-01 |  |  |  |  |
| 258 | 3.46E-02 | 7.54E-01 |  |  |  |  |
| 250 | 3.62E-02 | 6.50E-01 | 2.41E-03 | 4.036 | 3.58 | 0.45 |
| 252 | 3.57E-02 | 6.68E-01 | -3.39E-03 | 4.155 | 4.05 | 0.11 |
| 253 | 3.63E-02 | 5.48E-01 | 4.02E-04 |  | 4.23 |  |
| 254 | 4.15E-02 | 6.94E-01 | 9.81E-04 | 2.638 | 3.03 | -0.39 |
| 256 | 3.35E-02 | 6.97E-01 | 6.09E-04 |  | 3.76 |  |
| 259 | 9.14E-02 | 7.41E-01 |  |  |  |  |
| 161 | 5.37E-02 | 5.65E-01 | 4.29E-03 | 2.229 | 2.26 | -0.03 |
| 162 | 5.37E-02 | 5.31E-01 | 3.85E-03 | 2.328 | 2.46 | -0.13 |
| 192 | 5.37E-02 | 6.25E-01 | 4.08E-03 | 1.81 | 2.00 | -0.19 |
| 163 | 5.37E-02 | 5.56E-01 | 4.12E-03 | 2.495 | 2.32 | 0.18 |
| 166 | 5.37E-02 | 5.68E-01 | 5.18E-05 | 2.482 | 2.62 | -0.13 |
| 168 | 5.37E-02 | 5.90E-01 | 1.45E-04 | 2.367 | 2.50 | -0.14 |
| 167 | 5.37E-02 | 5.53E-01 | 3.77E-03 | 2.495 | 2.36 | 0.13 |
| 174 | 5.52E-02 | 6.66E-01 | 1.09E-03 | 1.724 | 1.93 | -0.21 |
| 180 | 5.89E-02 | 6.18E-01 | 7.49E-03 | 1.257 | 1.27 | -0.02 |
| 170 | 5.37E-02 | 6.33E-01 | 3.86E-04 | 2.638 | 2.28 | 0.36 |
| 172 | 5.56E-02 | 6.24E-01 | 9.83E-04 | 2.097 | 2.10 | -0.01 |
| 183 | 5.37E-02 | 6.04E-01 | 4.03E-04 | 2.222 | 2.42 | -0.19 |
| 169 | 5.37E-02 | 5.89E-01 | 4.23E-03 | 2.523 | 2.16 | 0.37 |
| 24 | 3.47E-02 | 8.62E-01 | 9.01E-03 | 1.731 | 2.15 | -0.42 |
| 175 | 5.52E-02 | 7.36E-01 | 4.00E-04 | 1.016 | 1.66 | -0.65 |
| 193 | 5.52E-02 | 5.89E-01 | 3.48E-03 | 2.658 | 2.09 | 0.57 |
| 227 | 5.52E-02 | 6.66E-01 | 3.02E-03 | 2.658 | 1.77 | 0.89 |
| 178* | 5.64E-02 | 5.41E-01 | 8.40E-05 | 2.252 | 2.50 | -0.25 |
| 181* | 5.52E-02 | 5.76E-01 | 8.77E-04 | 2.678 | 2.37 | 0.31 |
| 184* | 5.37E-02 | 6.49E-01 | 4.01E-03 | 1.996 | 1.89 | 0.10 |
| 185* | 5.52E-02 | 5.71E-01 | 4.82E-03 | 1.824 | 2.06 | -0.23 |
| 232* | 5.89E-02 | 6.08E-01 | 3.69E-03 | 1.517 | 1.65 | -0.13 |
| 246* | 3.66E-02 | 5.29E-01 | 4.09E-03 | 4.367 | 3.97 | 0.40 |
| 251* | 3.60E-02 | 5.07E-01 | -2.64E-03 | 4.495 | 4.71 | -0.21 |
| 238# | 3.52E-02 | 7.31E-01 | 7.88E-03 | 1.226 | 2.82 | -1.59 |
| 228* | 5.67E-02 | 5.52E-01 | 1.43E-03 | 2.409 | 2.30 | 0.11 |
| 255* | 3.73E-02 | 6.91E-01 | -3.76E-03 | 4.42 | 3.83 | 0.59 |
| 249* | 3.59E-02 | 6.34E-01 | 5.37E-04 | 4.276 | 3.85 | 0.43 |

**Table S51: Descriptor, experimental and predicted pIC_50_ values and their residuals for test set 2 compounds in cell line based QSAR model against DU145**

| No. | RNO | HS-1z | H-HC-2/ST | Exp. | Pred. | Res. |
| --- | --- | --- | --- | --- | --- | --- |
| 104 | 7.58E-02 | 1.83E+00 | 1.86E-02 | 1.764 | 2.78 | -1.02 |
| 108 | 5.77E-02 | 0.00E+00 | 1.08E-02 | 1.901 | 2.62 | -0.72 |
| 110 | 8.33E-02 | 4.95E+01 | 2.28E-02 | 3.959 | 3.41 | 0.55 |
| 111 | 1.11E-01 | 3.89E+01 | 2.14E-02 | 2.733 | 3.74 | -1.01 |
| 113 | 9.09E-02 | 6.09E-01 | 1.07E-02 | 2.572 | 3.16 | -0.58 |
| 114 | 9.09E-02 | 0.00E+00 | 1.12E-02 | 2.824 | 3.14 | -0.32 |
| 115 | 5.56E-02 | 1.40E+01 | 2.19E-02 | 3.456 | 2.55 | 0.91 |
| 116 | 8.00E-02 | 2.92E+01 | 2.65E-02 | 3.959 | 3.03 | 0.93 |
| 117 | 8.33E-02 | 4.78E+01 | 2.30E-02 | 2.971 | 3.38 | -0.41 |
| 118 | 7.55E-02 | 2.57E+01 | 2.83E-02 | 3.796 | 2.88 | 0.91 |
| 119 | 7.55E-02 | 2.27E+01 | 2.94E-02 | 3.585 | 2.82 | 0.76 |
| 122 | 5.77E-02 | 3.04E+00 | 6.34E-03 | 3.62 | 2.75 | 0.87 |
| 121 | 7.55E-02 | 2.15E+01 | 2.84E-02 | 4.602 | 2.83 | 1.77 |
| 192 | 1.25E-02 | 4.70E+01 | 1.34E-02 | 1.967 | 2.44 | -0.48 |
| 161 | 4.35E-02 | 4.77E+01 | 1.31E-02 | 2.387 | 2.95 | -0.56 |
| 196 | 1.79E-02 | 4.60E+01 | 1.47E-02 | 2.387 | 2.49 | -0.10 |
| 195 | 2.00E-02 | 4.65E+01 | 1.59E-02 | 1.762 | 2.51 | -0.74 |
| 199 | 0.00E+00 | 4.22E+01 | 1.06E-02 | 2.678 | 2.24 | 0.44 |
| 165 | 3.41E-02 | 4.83E+01 | 1.34E-02 | 2.26 | 2.80 | -0.54 |
| 166 | 3.41E-02 | 4.83E+01 | 1.34E-02 | 2.699 | 2.80 | -0.10 |
| 167 | 3.49E-02 | 5.05E+01 | 1.38E-02 | 2.824 | 2.83 | -0.01 |
| 168 | 2.56E-02 | 5.30E+01 | 1.23E-02 | 2.187 | 2.74 | -0.56 |
| 169 | 2.56E-02 | 4.98E+01 | 1.37E-02 | 2.959 | 2.68 | 0.28 |
| 172 | 2.47E-02 | 4.88E+01 | 1.34E-02 | 2.337 | 2.66 | -0.32 |
| 174 | 2.22E-02 | 5.15E+01 | 1.62E-02 | 2.409 | 2.60 | -0.19 |
| 175 | 3.51E-02 | 3.86E+01 | 2.49E-02 |  | 2.47 |  |
| 176 | 3.51E-02 | 5.00E+01 | 2.06E-02 |  | 2.70 |  |
| 178 | 3.64E-02 | 5.16E+01 | 2.27E-02 | 3.046 | 2.70 | 0.35 |
| 179 | 3.77E-02 | 5.21E+01 | 2.49E-02 | 2.301 | 2.68 | -0.38 |
| 180 | 4.26E-02 | 1.00E+02 | 3.95E-02 |  | 3.06 |  |
| 182 | 2.27E-02 | 4.76E+01 | 2.29E-02 | 2.745 | 2.43 | 0.32 |
| 184 | 1.28E-02 | 5.13E+01 | 1.39E-02 | 2.824 | 2.49 | 0.33 |
| 185 | 3.49E-02 | 4.68E+01 | 1.57E-02 | 1.827 | 2.75 | -0.92 |
| 186 | 3.75E-02 | 5.04E+01 | 1.50E-02 |  | 2.85 |  |
| 189 | 3.66E-02 | 4.81E+01 | 2.05E-02 |  | 2.70 |  |
| 190 | 2.47E-02 | 5.20E+01 | 2.20E-02 | 3.155 | 2.53 | 0.63 |
| 194 | 4.35E-02 | 5.41E+01 | 5.75E-02 |  | 2.16 |  |
| 229 | 8.33E-02 | 3.82E+01 | 1.48E-02 |  | 3.42 |  |
| 230 | 1.17E-01 | 3.76E+01 | 1.39E-02 |  | 3.96 |  |
| 231 | 8.70E-02 | 3.67E+01 | 1.87E-02 |  | 3.39 |  |
| 232 | 8.70E-02 | 4.99E+01 | 1.76E-02 |  | 3.57 |  |
| 233 | 9.09E-02 | 4.27E+01 | 2.15E-02 |  | 3.47 |  |
| 235 | 3.33E-02 | 4.40E+00 | 0.00E+00 |  | 2.50 |  |
| 236 | 5.88E-02 | 4.40E+00 | 7.09E-03 |  | 2.77 |  |
| 237 | 5.88E-02 | 4.40E+00 | 6.30E-03 |  | 2.78 |  |
| 238 | 5.88E-02 | 8.67E+01 | 1.68E-02 |  | 3.60 |  |
| 239 | 7.89E-02 | 3.77E+00 | 4.88E-03 |  | 3.12 |  |
| 240 | 7.89E-02 | 8.92E+01 | 1.96E-02 | 3.373 | 3.89 | -0.52 |
| 241 | 3.33E-02 | 3.79E+01 | 0.00E+00 |  | 2.92 |  |
| 242 | 1.47E-02 | 3.68E+01 | 6.16E-03 |  | 2.49 |  |
| 243 | 8.51E-02 | 9.03E+01 | 1.62E-02 | 5.155 | 4.07 | 1.09 |
| 244 | 9.09E-02 | 9.66E+01 | 1.71E-02 | 3.943 | 4.22 | -0.28 |
| 245 | 9.09E-02 | 9.60E+01 | 1.75E-02 | 4.456 | 4.20 | 0.25 |
| 246 | 1.15E-01 | 8.87E+01 | 1.56E-02 | 4.921 | 4.53 | 0.39 |
| 247 | 1.15E-01 | 8.87E+01 | 1.56E-02 | 3.991 | 4.53 | -0.54 |
| 248 | 1.30E-01 | 9.41E+01 | 1.65E-02 | 4.022 | 4.82 | -0.79 |
| 249 | 8.89E-02 | 1.58E+02 | 2.37E-02 | 4.284 | 4.82 | -0.53 |
| 257 | 1.30E-01 | 2.45E+01 | 1.73E-02 |  | 3.94 |  |
| 258 | 1.22E-01 | 0.00E+00 | 0.00E+00 |  | 3.85 |  |
| 250 | 8.51E-02 | 9.63E+01 | 1.66E-02 | 4.301 | 4.13 | 0.17 |
| 251 | 9.09E-02 | 8.87E+01 | 1.65E-02 | 4.678 | 4.13 | 0.55 |
| 252 | 8.00E-02 | 9.60E+01 | 1.66E-02 | 4.509 | 4.05 | 0.46 |
| 253 | 9.30E-02 | 1.14E+02 | 2.39E-02 |  | 4.34 |  |
| 256 | 1.18E-01 | 1.08E+02 | 1.94E-02 |  | 4.74 |  |
| 259 | 1.18E-01 | 8.72E+01 | 5.22E-02 |  | 3.85 |  |
| 227 | 2.47E-02 | 4.24E+01 | 2.04E-02 | 2.77 | 2.44 | 0.33 |
| 228 | 3.70E-02 | 4.51E+01 | 2.56E-02 | 2.721 | 2.57 | 0.15 |
| 261 | 6.25E-02 | 3.52E+01 | 3.58E-02 | 2.914 | 2.65 | 0.27 |
| 263 | 1.00E-01 | 8.52E+00 | 2.16E-02 | 3.678 | 3.19 | 0.49 |
| 264 | 8.77E-02 | 6.71E+01 | 2.30E-02 | 4.208 | 3.69 | 0.52 |
| 265 | 6.78E-02 | 6.65E+01 | 2.26E-02 | 4.268 | 3.38 | 0.89 |
| 266 | 9.80E-02 | 4.67E+01 | 2.40E-02 | 3.337 | 3.58 | -0.24 |
| 267 | 9.26E-02 | 1.55E+01 | 1.65E-02 | 2.377 | 3.26 | -0.88 |
| 268 | 1.19E-01 | 4.26E+00 | 1.29E-02 | 3.284 | 3.60 | -0.32 |
| 269 | 7.14E-02 | 2.27E+01 | 1.60E-02 | 2.253 | 3.02 | -0.77 |
| 271 | 7.94E-02 | 3.65E+00 | 1.22E-02 | 3.721 | 2.98 | 0.74 |
| 273 | 7.41E-02 | 3.29E+01 | 4.05E-02 | 1.232 | 2.71 | -1.48 |
| 274 | 1.02E-01 | 7.92E+00 | 3.49E-02 | 2.213 | 2.95 | -0.74 |
| 275 | 1.96E-02 | 3.79E+01 | 2.48E-02 | 1.216 | 2.22 | -1.00 |
| 276 | 5.36E-02 | 2.57E+01 | 3.98E-02 |  | 2.31 |  |
| 277 | 7.27E-02 | 2.87E+01 | 2.29E-02 |  | 2.98 |  |
| 278 | 1.00E-01 | 4.87E+00 | 2.23E-02 | 2.004 | 3.13 | -1.12 |
| 279 | 5.26E-02 | 2.21E+01 | 2.22E-02 |  | 2.60 |  |
| 281 | 5.45E-02 | 2.33E+01 | 1.65E-02 |  | 2.75 |  |
| 282 | 8.33E-02 | 4.26E+00 | 1.21E-02 | 3.959 | 3.05 | 0.91 |
| 283 | 1.59E-02 | 2.64E+01 | 1.81E-02 | 2.099 | 2.15 | -0.05 |
| 284 | 4.41E-02 | 9.74E+00 | 2.33E-02 | 2.451 | 2.29 | 0.16 |
| 285 | 1.67E-02 | 2.82E+01 | 1.95E-02 | 1.996 | 2.16 | -0.16 |
| 287 | 5.56E-02 | 2.21E+01 | 1.62E-02 | 1.914 | 2.76 | -0.85 |
| 288 | 8.47E-02 | 4.87E+00 | 1.22E-02 | 3.854 | 3.08 | 0.77 |
| 289 | 3.92E-02 | 9.03E+01 | 2.34E-02 | 3.31 | 3.20 | 0.11 |
| 291 | 9.52E-02 | 9.74E+00 | 2.35E-02 | 2.469 | 3.09 | -0.62 |
| 292 | 9.43E-02 | 2.34E+01 | 2.98E-02 | 3.721 | 3.12 | 0.60 |
| 293 | 7.27E-02 | 2.46E+01 | 2.85E-02 |  | 2.82 |  |
| 294 | 8.77E-02 | 4.18E+01 | 1.14E-02 |  | 3.60 |  |
| 295 | 9.43E-02 | 3.59E+01 | 2.98E-02 | 3.469 | 3.28 | 0.19 |
| 297 | 1.23E-01 | 2.09E+01 | 3.76E-02 |  | 3.39 |  |
| 299 | 1.09E-01 | 2.39E+01 | 2.57E-02 |  | 3.44 |  |
| 302 | 5.88E-02 | 1.69E+01 | 2.28E-02 | 2.66 | 2.62 | 0.04 |
| 303 | 6.00E-02 | 4.21E+01 | 3.00E-02 |  | 2.81 |  |
| 306 | 5.88E-02 | 1.63E+01 | 2.27E-02 | 2.69 | 2.61 | 0.08 |
| 311 | 6.90E-02 | 4.91E+01 | 3.69E-02 | 2.991 | 2.90 | 0.09 |
| 312 | 5.36E-02 | 4.27E+01 | 2.81E-02 | 2.541 | 2.75 | -0.21 |
| 315 | 5.88E-02 | 2.84E+01 | 2.43E-02 | 2.74 | 2.73 | 0.01 |
| 319 | 7.55E-02 | 3.58E+01 | 4.41E-02 |  | 2.70 |  |
| 320 | 5.17E-02 | 4.64E+01 | 3.26E-02 | 2.59 | 2.68 | -0.09 |
| 326 | 1.85E-02 | 2.42E+01 | 1.89E-02 | 2.58 | 2.15 | 0.43 |
| 329 | 4.08E-02 | 3.70E+01 | 3.46E-02 | 1.914 | 2.35 | -0.44 |
| 332 | 1.96E-02 | 2.23E+01 | 1.92E-02 | 2.561 | 2.14 | 0.43 |
| 334 | 4.35E-02 | 3.83E+01 | 2.83E-02 |  | 2.53 |  |
| 335 | 2.27E-02 | 4.02E+01 | 1.31E-02 | 3.319 | 2.52 | 0.80 |
| 336 | 1.82E-02 | 4.33E+01 | 2.07E-02 |  | 2.34 |  |
| 337 | 1.96E-02 | 2.60E+01 | 1.85E-02 | 1.631 | 2.20 | -0.56 |
| 340 | 1.96E-02 | 2.60E+01 | 1.75E-02 |  | 2.21 |  |
| 103 | 7.58E-02 | 3.04E+00 | 1.57E-02 | 2.963 | 2.85 | 0.11 |
| 105 | 5.88E-02 | 1.64E+01 | 2.28E-02 | 2.018 | 2.61 | -0.59 |
| 106 | 7.58E-02 | 4.26E+00 | 1.84E-02 | 3.032 | 2.81 | 0.22 |
| 112 | 8.77E-02 | 0.00E+00 | 1.85E-02 | 2.991 | 2.95 | 0.04 |
| 162 | 4.44E-02 | 4.77E+01 | 1.36E-02 | 2.921 | 2.95 | -0.03 |
| 193 | 2.47E-02 | 5.28E+01 | 2.06E-02 | 2.77 | 2.57 | 0.20 |
| 163 | 4.44E-02 | 4.77E+01 | 1.35E-02 | 3.046 | 2.95 | 0.10 |
| 197 | 1.79E-02 | 4.72E+01 | 1.47E-02 | 2.553 | 2.50 | 0.05 |
| 170 | 2.56E-02 | 4.77E+01 | 1.43E-02 | 2.658 | 2.64 | 0.02 |
| 173 | 2.47E-02 | 5.05E+01 | 2.03E-02 | 2.638 | 2.54 | 0.10 |
| 181 | 2.53E-02 | 4.82E+01 | 2.31E-02 | 2.62 | 2.47 | 0.15 |
| 188 | 1.22E-02 | 5.64E+01 | 2.06E-02 | 2.328 | 2.41 | -0.09 |
| 255 | 9.76E-02 | 1.42E+02 | 2.48E-02 | 4.658 | 4.74 | -0.08 |
| 262 | 1.21E-01 | 9.13E+00 | 2.28E-02 | 3.469 | 3.50 | -0.03 |
| 272 | 4.08E-02 | 4.21E+01 | 2.58E-02 | 2.578 | 2.59 | -0.01 |
| 280 | 8.06E-02 | 4.26E+00 | 2.37E-02 | 3.076 | 2.79 | 0.29 |
| 286 | 4.62E-02 | 7.31E+00 | 2.40E-02 | 2.516 | 2.28 | 0.24 |
| 340 | 4.00E-02 | 3.82E+01 | 2.88E-02 | 2.4 | 2.47 | -0.07 |
| 296 | 9.52E-02 | 3.42E+01 | 2.93E-02 | 5 | 3.28 | 1.72 |
| 316 | 5.56E-02 | 2.47E+01 | 3.21E-02 | 1.29 | 2.48 | -1.19 |
| 109 | 9.84E-02 | 4.26E+00 | 1.22E-02 | 3.244 | 3.29 | -0.05 |
| 102* | 5.56E-02 | 1.16E+01 | 2.30E-02 | 3.086 | 2.50 | 0.59 |
| 107* | 5.88E-02 | 1.40E+01 | 2.25E-02 | 3.108 | 2.59 | 0.52 |
| 270* | 6.00E-02 | 6.41E+01 | 2.20E-02 | 2.959 | 3.24 | -0.28 |
| 120* | 7.25E-02 | 6.09E+00 | 1.73E-02 | 3.076 | 2.81 | 0.27 |
| 198* | 1.61E-02 | 4.58E+01 | 1.37E-02 | 2.658 | 2.48 | 0.18 |
| 332* | 4.00E-02 | 3.89E+01 | 2.93E-02 | 2.78 | 2.47 | 0.31 |
| 327* | 1.72E-02 | 4.46E+01 | 2.06E-02 | 2.205 | 2.35 | -0.14 |
| 317* | 8.16E-02 | 4.36E+01 | 4.46E-02 | 2.74 | 2.88 | -0.14 |
| 312* | 6.45E-02 | 1.69E+01 | 2.14E-02 | 3.119 | 2.73 | 0.39 |
| 306* | 7.14E-02 | 2.10E+01 | 3.72E-02 | 2.991 | 2.59 | 0.41 |
| 304* | 7.14E-02 | 1.13E+01 | 2.23E-02 | 3.032 | 2.76 | 0.28 |
| 297* | 1.15E-01 | 6.09E-01 | 2.09E-02 | 3.81 | 3.34 | 0.47 |
| 290* | 1.05E-01 | 1.10E+01 | 2.46E-02 | 3.42 | 3.24 | 0.18 |
| 191* | 2.50E-02 | 6.16E+01 | 3.29E-02 | 2.495 | 2.44 | 0.06 |
| 187* | 0.00E+00 | 5.40E+01 | 1.44E-02 | 2.509 | 2.31 | 0.20 |
| 183* | 1.28E-02 | 4.78E+01 | 1.43E-02 | 2.745 | 2.44 | 0.31 |
| 177* | 3.70E-02 | 4.30E+01 | 2.56E-02 | 2.721 | 2.54 | 0.18 |
| 254# | 9.52E-02 | 1.24E+02 | 3.33E-02 | 2.551 | 4.31 | -1.76 |
| 234* | 9.52E-02 | 6.92E+00 | 5.93E-03 | 4.149 | 3.40 | 0.75 |

**Table S52: Descriptor, experimental and predicted pIC_50_ values and their residuals for test set 2 compounds in cell line based QSAR model against Fibroblast**

| **No.** | **MiVN** | **L1E** | **THCMD** | **Exp.** | **Pred.** | **Res.** |
| --- | --- | --- | --- | --- | --- | --- |
| 192 | 2.98E+00 | 7.73E-02 | -1.41E+00 | 1.526 | 1.90 | -0.37 |
| 162 | 2.99E+00 | 3.02E-01 | -2.00E+00 | 2.319 | 2.42 | -0.10 |
| 163 | 2.99E+00 | 3.04E-01 | -1.91E+00 | 2.398 | 2.40 | 0.00 |
| 165 | 2.98E+00 | 9.69E-02 | -6.70E-01 | 1.69 | 1.71 | -0.02 |
| 169 | 2.98E+00 | 1.51E-01 | -1.56E+00 | 2.602 | 2.00 | 0.61 |
| 170 | 2.98E+00 | 2.10E-01 | -2.09E+00 | 2.092 | 2.18 | -0.09 |
| 173 | 2.98E+00 | -2.66E-01 | -2.41E+00 | 2.201 | 1.91 | 0.29 |
| 174 | 2.98E+00 | -3.01E-01 | -1.32E+00 |  | 1.59 |  |
| 175 | 2.99E+00 | -4.03E-01 | -1.06E+00 |  | 1.63 |  |
| 176 | 2.98E+00 | -2.54E-01 | -1.09E+00 |  | 1.56 |  |
| 177 | 2.98E+00 | -8.68E-01 | -2.99E+00 | 1.539 | 1.61 | -0.07 |
| 181 | 2.98E+00 | -1.28E-01 | -2.32E+00 | 1.854 | 1.99 | -0.14 |
| 179 | 3.00E+00 | -8.17E-01 | -7.80E-01 | 1.476 | 1.43 | 0.04 |
| 180 | 2.98E+00 | -4.08E-01 | -2.49E+00 |  | 1.82 |  |
| 184 | 2.98E+00 | 7.54E-02 | -1.50E+00 | 1.907 | 1.92 | -0.02 |
| 185 | 2.98E+00 | -6.08E-01 | -1.45E+00 | 1.239 | 1.39 | -0.15 |
| 186 | 2.98E+00 | -4.34E-01 | -1.41E+00 |  | 1.51 |  |
| 187 | 2.96E+00 | 1.40E-01 | -2.09E-01 | 1.222 | 1.24 | -0.01 |
| 189 | 2.93E+00 | -2.67E-01 | -8.14E-01 |  | 0.51 |  |
| 191 | 2.96E+00 | -3.23E-01 | -1.16E+00 | 1.143 | 1.14 | 0.00 |
| 194 | 3.04E+00 | 5.25E-01 | -1.81E+00 |  | 3.50 |  |
| 161* | 2.99E+00 | 3.01E-01 | -1.99E+00 | 2.328 | 2.42 | -0.09 |
| 168* | 2.99E+00 | 3.52E-01 | -1.44E+00 | 2.143 | 2.31 | -0.17 |
| 178* | 3.00E+00 | -8.13E-01 | -2.76E+00 | 1.87 | 1.97 | -0.10 |
| 183* | 2.98E+00 | 7.35E-02 | -1.42E+00 | 1.936 | 1.90 | 0.04 |
| 188* | 2.96E+00 | -2.36E-01 | -1.84E+00 | 1.073 | 1.39 | -0.32 |
| 182* | 2.98E+00 | -1.26E-01 | -2.34E+00 | 2.155 | 2.00 | 0.16 |

**Table S53:** Descriptor, experimental and predicted pIC_50_ values and their residuals for test set 2 compounds in cell line based QSAR model against HCT-15

| **No.** | **AVN** | **MiNACN** | **MaVC** | **Exp.** | **Pred.** | **Res.** |
| --- | --- | --- | --- | --- | --- | --- |
| 1 | 3.50E+00 | -5.14E-01 | 3.99E+00 | 2.585 | 2.61 | -0.03 |
| 6 | 3.25E+00 | -6.40E-01 | 3.99E+00 |  | 2.24 |  |
| 7 | 3.25E+00 | -6.33E-01 | 3.99E+00 |  | 2.22 |  |
| 8 | 3.26E+00 | -6.21E-01 | 4.01E+00 | 2.26 | 2.43 | -0.17 |
| 9 | 3.25E+00 | -6.33E-01 | 3.99E+00 |  | 2.22 |  |
| 10 | 3.26E+00 | -6.22E-01 | 4.00E+00 | 2.553 | 2.33 | 0.23 |
| 11 | 3.26E+00 | -6.10E-01 | 4.06E+00 |  | 2.91 |  |
| 12 | 3.27E+00 | -6.07E-01 | 3.99E+00 |  | 2.22 |  |
| 16 | 3.23E+00 | -7.01E-01 | 4.06E+00 | 3.046 | 3.00 | 0.04 |
| 17 | 3.23E+00 | -7.06E-01 | 3.99E+00 | 2.469 | 2.31 | 0.16 |
| 18 | 3.22E+00 | -7.26E-01 | 3.99E+00 | 2.357 | 2.32 | 0.04 |
| 21 | 3.20E+00 | -6.90E-01 | 3.99E+00 | 2.252 | 2.21 | 0.05 |
| 22 | 3.23E+00 | -6.58E-01 | 3.99E+00 | 2.131 | 2.22 | -0.09 |
| 23 | 3.22E+00 | -6.60E-01 | 3.99E+00 | 2.114 | 2.20 | -0.09 |
| 24 | 3.22E+00 | -6.59E-01 | 3.99E+00 | 2.237 | 2.20 | 0.04 |
| 25 | 3.24E+00 | -6.57E-01 | 3.99E+00 | 2.337 | 2.24 | 0.10 |
| 26 | 3.21E+00 | -5.13E-01 | 4.00E+00 | 2.046 | 2.01 | 0.04 |
| 27 | 3.22E+00 | -5.13E-01 | 4.00E+00 |  | 2.03 |  |
| 28 | 3.22E+00 | -5.13E-01 | 4.00E+00 |  | 2.03 |  |
| 29 | 3.21E+00 | -5.13E-01 | 4.00E+00 |  | 2.01 |  |
| 30 | 3.18E+00 | -5.13E-01 | 4.00E+00 | 2.004 | 1.94 | 0.07 |
| 31 | 3.41E+00 | -5.13E-01 | 4.00E+00 |  | 2.49 |  |
| 32 | 3.22E+00 | -6.13E-01 | 4.00E+00 | 2.086 | 2.21 | -0.13 |
| 33 | 3.21E+00 | -6.61E-01 | 3.99E+00 |  | 2.18 |  |
| 34 | 3.40E+00 | -6.25E-01 | 4.00E+00 |  | 2.67 |  |
| 37 | 3.24E+00 | -6.25E-01 | 4.00E+00 |  | 2.28 |  |
| 38 | 3.24E+00 | -6.30E-01 | 4.01E+00 |  | 2.39 |  |
| 39 | 3.20E+00 | -6.28E-01 | 4.00E+00 |  | 2.19 |  |
| 2 | 3.16E+00 | -8.20E-01 | 3.99E+00 | 2.284 | 2.34 | -0.06 |
| 4 | 3.26E+00 | -6.17E-01 | 3.99E+00 | 2.108 | 2.22 | -0.11 |
| 3 | 3.26E+00 | -5.13E-01 | 3.99E+00 | 2.037 | 2.03 | 0.01 |
| 14 | 3.25E+00 | -6.43E-01 | 3.99E+00 | 2.143 | 2.24 | -0.10 |
| 20 | 3.00E+00 | -6.53E-01 | 4.03E+00 | 2.013 | 2.06 | -0.04 |
| 5 | 3.26E+00 | -6.22E-01 | 3.99E+00 | 2.367 | 2.23 | 0.14 |
| 35* | 3.22E+00 | -6.28E-01 | 4.00E+00 | 2.174 | 2.24 | -0.07 |
| 13* | 3.25E+00 | -6.29E-01 | 3.99E+00 | 2.076 | 2.22 | -0.14 |
| 15* | 3.23E+00 | -6.90E-01 | 3.99E+00 | 2.347 | 2.28 | 0.07 |
| 19* | 3.22E+00 | -6.59E-01 | 4.01E+00 | 2.509 | 2.40 | 0.11 |
| 36* | 3.24E+00 | -6.30E-01 | 3.99E+00 | 2.229 | 2.19 | 0.04 |

**Table S54:** Descriptor, experimental and predicted pIC_50_ values and their residuals for test set 2 compounds in cell line based QSAR model against HOP-62

| **No.** | **ZXS/ZXR** | **RPCSQ** | **NF** | **EXP** | **Pred.** | **Res.** |
| --- | --- | --- | --- | --- | --- | --- |
| 296 | 5.61E-01 | 1.63E-01 | 0.00E+00 | 5 | 4.72 | 0.28 |
| 297 | 6.05E-01 | 1.61E-01 | 0.00E+00 | 4.699 | 4.16 | 0.54 |
| 298 | 6.42E-01 | 2.13E-01 | 0.00E+00 | 2.75 | 3.47 | -0.72 |
| 302 | 6.47E-01 | 3.48E-01 | 0.00E+00 | 2.48 | 2.86 | -0.38 |
| 303 | 6.39E-01 | 4.44E-01 | 0.00E+00 | 1.71 | 2.57 | -0.86 |
| 306 | 6.44E-01 | 3.48E-01 | 0.00E+00 | 2.245 | 2.89 | -0.65 |
| 311 | 6.91E-01 | 2.92E-01 | 0.00E+00 | 2.996 | 2.51 | 0.49 |
| 315 | 7.03E-01 | 3.96E-01 | 0.00E+00 | 1.77 | 1.93 | -0.16 |
| 316 | 6.55E-01 | 3.83E-01 | 0.00E+00 | 3.181 | 2.61 | 0.57 |
| 317 | 6.81E-01 | 4.10E-01 | 0.00E+00 |  | 2.16 |  |
| 318 | 6.42E-01 | 3.61E-01 | 0.00E+00 | 3.319 | 2.87 | 0.45 |
| 319 | 6.37E-01 | 3.81E-01 | 0.00E+00 | 2.3 | 2.85 | -0.55 |
| 326 | 6.58E-01 | 3.85E-01 | 1.00E+00 | 1.697 | 1.82 | -0.12 |
| 327 | 6.74E-01 | 3.81E-01 | 1.00E+00 |  | 1.63 |  |
| 328 | 6.93E-01 | 3.01E-01 | 1.00E+00 | 1.435 | 1.70 | -0.27 |
| 329 | 6.92E-01 | 3.40E-01 | 1.00E+00 | 1.951 | 1.56 | 0.39 |
| 334 | 7.05E-01 | 3.90E-01 | 0.00E+00 | 2.14 | 1.93 | 0.21 |
| 333 | 6.28E-01 | 4.34E-01 | 0.00E+00 | 2.979 | 2.75 | 0.23 |
| 335 | 5.74E-01 | 5.37E-01 | 0.00E+00 | 3.337 | 3.04 | 0.30 |
| 341 | 6.60E-01 | 5.05E-01 | 0.00E+00 | 2.78 | 2.05 | 0.73 |
| 312 | 6.25E-01 | 3.20E-01 | 0.00E+00 | 3.244 | 3.26 | -0.01 |
| 332 | 6.51E-01 | 4.38E-01 | 0.00E+00 | 2.64 | 2.44 | 0.20 |
| 336 | 6.67E-01 | 4.06E-01 | 0.00E+00 | 2.699 | 2.36 | 0.34 |
| 314 | 6.44E-01 | 3.67E-01 | 0.00E+00 | 2.67 | 2.82 | -0.15 |
| 337 | 6.40E-01 | 4.35E-01 | 0.00E+00 | 1.742 | 2.59 | -0.85 |
| 307* | 6.74E-01 | 3.07E-01 | 0.00E+00 | 2.996 | 2.67 | 0.33 |
| 313* | 6.64E-01 | 2.88E-01 | 0.00E+00 | 3.268 | 2.88 | 0.39 |
| 340* | 6.87E-01 | 3.63E-01 | 0.00E+00 | 2.731 | 2.27 | 0.46 |
| 305* | 6.50E-01 | 3.91E-01 | 0.00E+00 | 2.42 | 2.64 | -0.22 |
| 342* | 6.96E-01 | 2.71E-01 | 0.00E+00 | 2.6 | 2.53 | 0.07 |

**Table S55:** Descriptor, experimental and predicted pIC_50_ values and their residuals for test set 2 compounds in cell line based QSAR model against HCT-116

| **No.** | **ZXS/ZXR** | **TPCCMD** | **RNCSQ** | **Exp.** | **Pred.** | **Res.** |
| --- | --- | --- | --- | --- | --- | --- |
| 296 | 5.61E-01 | 1.03E+01 | 2.09E+00 | 4.523 | 4.40 | 0.12 |
| 300 | 7.96E-01 | 4.30E+00 | 9.09E+00 | 1.242 | 1.29 | -0.05 |
| 301 | 7.44E-01 | 6.51E+00 | 6.95E+00 |  | 2.13 |  |
| 307 | 6.74E-01 | 6.80E+00 | 7.70E-01 | 3.086 | 3.04 | 0.05 |
| 308 | 6.62E-01 | 7.99E+00 | 7.13E-01 |  | 3.31 |  |
| 311 | 6.91E-01 | 6.94E+00 | 2.84E+00 | 3.086 | 2.82 | 0.26 |
| 314 | 6.44E-01 | 5.37E+00 | 5.02E+00 |  | 2.88 |  |
| 315 | 7.03E-01 | 7.53E+00 | 1.97E+00 | 2.71 | 2.85 | -0.14 |
| 316 | 6.55E-01 | 3.56E+00 | 1.19E+00 | 2.839 | 2.71 | 0.13 |
| 317 | 6.81E-01 | 5.08E+00 | 3.26E+00 | 3.022 | 2.62 | 0.40 |
| 318 | 6.42E-01 | 3.59E+00 | 5.92E+00 |  | 2.60 |  |
| 326 | 6.58E-01 | 2.51E+00 | 9.26E-01 | 2.745 | 2.55 | 0.20 |
| 327 | 6.74E-01 | 5.16E+00 | 7.97E-01 |  | 2.80 |  |
| 328 | 6.93E-01 | 4.59E+00 | 6.56E-01 | 2.516 | 2.57 | -0.06 |
| 329 | 6.92E-01 | 1.47E+00 | 2.70E+00 | 1.735 | 2.04 | -0.30 |
| 333 | 6.28E-01 | 3.38E+00 | 2.97E+00 | 2.979 | 2.82 | 0.16 |
| 334 | 7.05E-01 | 6.33E+00 | 3.03E+00 | 2.67 | 2.61 | 0.06 |
| 335 | 5.74E-01 | 7.04E+00 | 6.57E+00 | 3.398 | 3.62 | -0.22 |
| 337 | 6.40E-01 | 2.00E+00 | 1.41E+00 | 1.87 | 2.60 | -0.73 |
| 340 | 6.87E-01 | 3.54E+00 | 1.35E+00 | 2.851 | 2.44 | 0.41 |
| 341 | 6.60E-01 | 4.36E+00 | 2.97E+00 | 2.81 | 2.70 | 0.11 |
| 342 | 6.96E-01 | 8.36E+00 | 1.80E-01 | 2.801 | 3.11 | -0.31 |
| 298 | 6.42E-01 | 4.12E+00 | 3.05E+00 | 2.939 | 2.81 | 0.13 |
| 302 | 6.47E-01 | 7.18E+00 | 5.79E+00 | 2.932 | 3.08 | -0.15 |
| 313 | 6.64E-01 | 6.05E+00 | 4.73E+00 | 2.848 | 2.83 | 0.02 |
| 314 | 6.37E-01 | 4.57E+00 | 2.37E+00 | 3.071 | 2.95 | 0.12 |
| 320 | 6.96E-01 | 8.28E+00 | 2.65E-01 | 2.879 | 3.10 | -0.22 |
| 305* | 6.50E-01 | 7.25E+00 | 5.34E+00 | 2.876 | 3.09 | -0.21 |
| 306* | 6.44E-01 | 5.54E+00 | 5.64E+00 | 2.58 | 2.87 | -0.29 |
| 312* | 6.25E-01 | 4.05E+00 | 5.45E+00 | 2.71 | 2.82 | -0.11 |
| 336 | 6.67E-01 | 4.63E+00 | 1.20E-01 | 2.74 | 2.82 | -0.08 |
| 332* | 6.51E-01 | 2.75E+00 | 1.12E+00 | 3.824 | 2.63 | 1.19 |
| 297* | 6.05E-01 | 9.57E+00 | 1.69E+00 | 4 | 3.96 | 0.04 |

**Table S56:** Descriptor, experimental and predicted pIC_50_ values and their residuals for test set 2 compounds in cell line based QSAR model against HeLa

| **No.** | **NN** | **W-1wP** | **MiVC** | **Exp.** | **Pred.** | **Res.** |
| --- | --- | --- | --- | --- | --- | --- |
| 42 | 2.00E+00 | 6.01E+01 | 3.83E+00 |  | 1.71 |  |
| 43 | 2.00E+00 | 6.27E+01 | 3.83E+00 |  | 1.72 |  |
| 44 | 2.00E+00 | 1.32E+02 | 3.72E+00 |  | 1.82 |  |
| 46 | 2.00E+00 | 1.28E+02 | 3.72E+00 |  | 1.81 |  |
| 50 | 3.00E+00 | 1.33E+02 | 3.83E+00 | 2.24 | 2.18 | 0.06 |
| 52 | 3.00E+00 | 1.32E+02 | 3.83E+00 | 2.8 | 2.18 | 0.62 |
| 53 | 4.00E+00 | 1.24E+02 | 3.83E+00 |  | 2.47 |  |
| 55 | 4.00E+00 | 1.26E+02 | 3.75E+00 |  | 2.43 |  |
| 56 | 4.00E+00 | 1.36E+02 | 3.75E+00 | 1.95 | 2.45 | -0.50 |
| 57 | 3.00E+00 | 1.34E+02 | 3.83E+00 | 2.13 | 2.19 | -0.06 |
| 60 | 3.00E+00 | 1.19E+02 | 3.80E+00 | 2.6 | 2.14 | 0.47 |
| 61 | 2.00E+00 | 1.64E+02 | 3.83E+00 |  | 1.95 |  |
| 62 | 2.00E+00 | 1.71E+02 | 3.83E+00 |  | 1.97 |  |
| 65 | 2.00E+00 | 1.25E+02 | 3.72E+00 |  | 1.80 |  |
| 66 | 2.00E+00 | 1.30E+02 | 3.73E+00 | 1.66 | 1.82 | -0.16 |
| 68 | 2.00E+00 | 1.69E+02 | 3.81E+00 |  | 1.95 |  |
| 70 | 2.00E+00 | 1.43E+02 | 3.83E+00 |  | 1.90 |  |
| 72 | 2.00E+00 | 1.40E+02 | 3.83E+00 | 1.88 | 1.90 | -0.02 |
| 74 | 2.00E+00 | 1.39E+02 | 3.83E+00 | 1.73 | 1.89 | -0.16 |
| 125 | 0.00E+00 | 8.28E+01 | 3.80E+00 | 1.19 | 1.14 | 0.05 |
| 128 | 0.00E+00 | 7.55E+01 | 3.76E+00 | 1.19 | 1.10 | 0.09 |
| 131 | 0.00E+00 | 1.06E+02 | 3.82E+00 | 1.33 | 1.20 | 0.13 |
| 132 | 0.00E+00 | 1.85E+02 | 3.76E+00 | 1.51 | 1.35 | 0.16 |
| 51 | 3.00E+00 | 1.24E+02 | 3.83E+00 | 2.44 | 2.16 | 0.28 |
| 136 | 0.00E+00 | 2.17E+02 | 3.76E+00 | 1.6 | 1.43 | 0.17 |
| 137 | 0.00E+00 | 2.00E+02 | 3.72E+00 | 1.14 | 1.37 | -0.23 |
| 139 | 0.00E+00 | 1.76E+02 | 3.76E+00 | 1.17 | 1.33 | -0.16 |
| 142 | 0.00E+00 | 1.35E+02 | 3.67E+00 | 1.14 | 1.19 | -0.05 |
| 143 | 0.00E+00 | 1.65E+02 | 3.82E+00 | 1.11 | 1.34 | -0.23 |
| 144 | 0.00E+00 | 1.74E+02 | 3.82E+00 | 1.36 | 1.36 | 0.00 |
| 145 | 0.00E+00 | 7.03E+01 | 3.82E+00 | 1.17 | 1.12 | 0.05 |
| 147 | 0.00E+00 | 7.40E+01 | 3.79E+00 | 1.12 | 1.11 | 0.01 |
| 149 | 0.00E+00 | 8.60E+01 | 3.79E+00 | 1.15 | 1.14 | 0.01 |
| 150 | 0.00E+00 | 9.28E+01 | 3.78E+00 | 1.14 | 1.15 | -0.01 |
| 151 | 0.00E+00 | 1.11E+02 | 3.78E+00 | 1.14 | 1.19 | -0.05 |
| 153 | 0.00E+00 | 1.18E+02 | 3.82E+00 | 1.13 | 1.23 | -0.10 |
| 154 | 0.00E+00 | 1.56E+02 | 3.82E+00 | 1.12 | 1.32 | -0.20 |
| 157 | 0.00E+00 | 8.99E+01 | 3.82E+00 | 1.17 | 1.17 | 0.01 |
| 158 | 0.00E+00 | 5.62E+01 | 3.83E+00 | 1.12 | 1.09 | 0.03 |
| 134 | 0.00E+00 | 2.87E+02 | 3.76E+00 | 1.92 | 1.59 | 0.33 |
| 40 | 2.00E+00 | 1.30E+02 | 3.83E+00 | 2.06 | 1.87 | 0.19 |
| 41 | 2.00E+00 | 1.32E+02 | 3.72E+00 | 1.87 | 1.82 | 0.05 |
| 47 | 2.00E+00 | 1.73E+02 | 3.65E+00 | 1.97 | 1.87 | 0.10 |
| 54 | 4.00E+00 | 1.23E+02 | 3.83E+00 | 2.24 | 2.46 | -0.22 |
| 64 | 2.00E+00 | 1.34E+02 | 3.76E+00 | 1.85 | 1.84 | 0.01 |
| 73 | 2.00E+00 | 1.24E+02 | 3.79E+00 | 1.73 | 1.84 | -0.11 |
| 124 | 0.00E+00 | 9.18E+01 | 3.69E+00 | 1.15 | 1.10 | 0.05 |
| 127 | 0.00E+00 | 7.27E+01 | 3.76E+00 | 1.15 | 1.09 | 0.06 |
| 130 | 0.00E+00 | 9.60E+01 | 3.82E+00 | 1.17 | 1.18 | -0.01 |
| 135 | 0.00E+00 | 1.84E+02 | 3.67E+00 | 1.22 | 1.30 | -0.08 |
| 141 | 0.00E+00 | 1.76E+02 | 3.82E+00 | 1.15 | 1.37 | -0.22 |
| 148 | 0.00E+00 | 8.01E+01 | 3.79E+00 | 1.13 | 1.13 | 0.00 |
| 152 | 0.00E+00 | 9.10E+01 | 3.80E+00 | 1.12 | 1.16 | -0.04 |
| 155 | 0.00E+00 | 1.90E+02 | 3.82E+00 | 1.21 | 1.40 | -0.19 |
| 156 | 0.00E+00 | 1.01E+02 | 3.82E+00 | 1.19 | 1.19 | 0.00 |
| 138 | 0.00E+00 | 1.62E+02 | 3.71E+00 | 1.49 | 1.27 | 0.22 |
| 48 | 3.00E+00 | 1.26E+02 | 3.83E+00 | 1.84 | 2.17 | -0.33 |
| 49* | 3.00E+00 | 1.29E+02 | 3.83E+00 | 2.28 | 2.17 | 0.11 |
| 160* | 0.00E+00 | 5.98E+01 | 3.83E+00 | 1.13 | 1.10 | 0.03 |
| 58* | 3.00E+00 | 1.39E+02 | 3.83E+00 | 1.95 | 2.20 | -0.25 |
| 59* | 3.00E+00 | 1.27E+02 | 3.80E+00 | 2.33 | 2.15 | 0.18 |
| 63* | 2.00E+00 | 1.45E+02 | 3.76E+00 | 1.69 | 1.87 | -0.18 |
| 67* | 2.00E+00 | 1.70E+02 | 3.77E+00 | 1.65 | 1.93 | -0.28 |
| 69* | 2.00E+00 | 1.39E+02 | 3.72E+00 | 1.84 | 1.83 | 0.01 |
| 159* | 2.00E+00 | 1.28E+02 | 3.73E+00 | 1.97 | 1.81 | 0.16 |
| 123* | 0.00E+00 | 6.68E+01 | 3.82E+00 | 1.22 | 1.11 | 0.11 |
| 126* | 0.00E+00 | 6.73E+01 | 3.69E+00 | 1.19 | 1.04 | 0.15 |
| 129* | 0.00E+00 | 9.20E+01 | 3.82E+00 | 1.14 | 1.17 | -0.03 |
| 71* | 0.00E+00 | 7.74E+01 | 3.80E+00 | 1.13 | 1.13 | 0.00 |
| 45# | 2.00E+00 | 1.41E+02 | 3.83E+00 | 2.62 | 1.90 | 0.72 |
| 140* | 0.00E+00 | 1.30E+02 | 3.82E+00 | 1.24 | 1.26 | -0.02 |
| 146* | 0.00E+00 | 6.74E+01 | 3.73E+00 | 1.17 | 1.06 | 0.11 |

**Table S57:** Descriptor, experimental and predicted pIC_50_ values and their residuals for test set 2 compounds in cell line based QSAR model against HL-60

| **No.** | **RNH** | **RNO** | **MaVO** | **Exp.** | **Pred.** | **Res.** |
| --- | --- | --- | --- | --- | --- | --- |
| 123 | 4.87E-01 | 1.08E-01 | 2.16E+00 | 1.77 | 1.67 | 0.10 |
| 126 | 4.88E-01 | 1.22E-01 | 2.16E+00 | 1.31 | 1.62 | -0.31 |
| 127 | 5.00E-01 | 1.14E-01 | 2.16E+00 | 1.39 | 1.56 | -0.17 |
| 132 | 4.52E-01 | 8.06E-02 | 2.16E+00 | 2.22 | 2.02 | 0.21 |
| 135 | 4.12E-01 | 1.32E-01 | 2.16E+00 | 2.3 | 2.11 | 0.19 |
| 136 | 4.48E-01 | 7.46E-02 | 2.16E+00 | 2.15 | 2.06 | 0.09 |
| 138 | 4.62E-01 | 1.15E-01 | 2.16E+00 | 2.22 | 1.82 | 0.40 |
| 139 | 4.52E-01 | 1.13E-01 | 2.26E+00 | 2.3 | 2.31 | -0.01 |
| 140 | 4.71E-01 | 9.80E-02 | 2.16E+00 | 2.05 | 1.82 | 0.23 |
| 143 | 4.73E-01 | 9.09E-02 | 2.15E+00 | 1.34 | 1.79 | -0.45 |
| 147 | 5.22E-01 | 1.09E-01 | 2.16E+00 | 1.22 | 1.42 | -0.20 |
| 148 | 5.31E-01 | 1.02E-01 | 2.16E+00 | 1.74 | 1.39 | 0.35 |
| 154 | 4.36E-01 | 1.03E-01 | 2.17E+00 | 1.89 | 2.09 | -0.20 |
| 155 | 4.39E-01 | 9.76E-02 | 2.20E+00 | 2.1 | 2.21 | -0.11 |
| 156 | 4.75E-01 | 1.25E-01 | 2.18E+00 | 1.92 | 1.78 | 0.14 |
| 158 | 5.37E-01 | 9.76E-02 | 2.14E+00 | 1.32 | 1.28 | 0.04 |
| 159 | 5.22E-01 | 1.09E-01 | 2.14E+00 | 1.32 | 1.34 | -0.02 |
| 153 | 4.62E-01 | 1.03E-01 | 2.18E+00 | 1.92 | 1.95 | -0.03 |
| 128 | 5.11E-01 | 1.06E-01 | 2.16E+00 | 1.42 | 1.51 | -0.09 |
| 141 | 4.51E-01 | 9.80E-02 | 2.16E+00 | 1.77 | 1.96 | -0.19 |
| 144 | 4.51E-01 | 9.80E-02 | 2.16E+00 | 2.05 | 1.96 | 0.09 |
| 146 | 5.12E-01 | 1.16E-01 | 2.16E+00 | 1.49 | 1.47 | 0.02 |
| 152 | 5.11E-01 | 1.28E-01 | 2.15E+00 | 1.36 | 1.39 | -0.03 |
| 160 | 5.00E-01 | 8.33E-02 | 2.16E+00 | 1.8 | 1.67 | 0.13 |
| 142 | 4.44E-01 | 1.30E-01 | 2.16E+00 | 1.74 | 1.90 | -0.16 |
| 137 | 4.57E-01 | 1.00E-01 | 2.16E+00 | 1.34 | 1.91 | -0.57 |
| 134 | 4.19E-01 | 8.06E-02 | 2.16E+00 | 2.52 | 2.25 | 0.27 |
| 149 | 5.39E-01 | 9.62E-02 | 2.16E+00 | 1.38 | 1.35 | 0.03 |
| 124 | 4.90E-01 | 1.43E-01 | 2.16E+00 | 1.77 | 1.53 | 0.24 |
| 125* | 4.88E-01 | 1.22E-01 | 2.16E+00 | 1.8 | 1.62 | 0.18 |
| 129* | 5.11E-01 | 8.51E-02 | 2.16E+00 | 1.35 | 1.59 | -0.24 |
| 145* | 5.00E-01 | 1.25E-01 | 2.16E+00 | 1.3 | 1.52 | -0.22 |
| 150* | 5.11E-01 | 1.28E-01 | 2.16E+00 | 1.36 | 1.43 | -0.07 |
| 151* | 5.26E-01 | 1.40E-01 | 2.15E+00 | 1.26 | 1.25 | 0.02 |
| 130* | 5.20E-01 | 8.00E-02 | 2.16E+00 | 1.38 | 1.54 | -0.16 |
| 157* | 4.74E-01 | 1.32E-01 | 2.16E+00 | 1.74 | 1.68 | 0.06 |
| 131* | 5.36E-01 | 7.14E-02 | 2.16E+00 | 1.62 | 1.46 | 0.16 |

**Table S58:** Descriptor, experimental and predicted pIC_50_ values and their residuals for test set 2 compounds in cell line based QSAR model against Hs468

| No. | MaVC | A1ERC | MiVO | Exp. | Pred. | Res. |
| --- | --- | --- | --- | --- | --- | --- |
| 4 | 3.99E+00 | -3.16E-06 | 1.99E+00 |  | 2.35 |  |
| 6 | 3.99E+00 | 6.19E-05 | 1.98E+00 |  | 2.29 |  |
| 7 | 3.99E+00 | 7.94E-05 | 1.99E+00 |  | 2.22 |  |
| 20 | 4.03E+00 | 2.51E-04 | 1.99E+00 | 2.301 | 2.35 | -0.05 |
| 9 | 3.99E+00 | 7.56E-05 | 2.00E+00 |  | 2.18 |  |
| 11 | 4.06E+00 | 5.64E-05 | 2.00E+00 |  | 2.90 |  |
| 16 | 4.06E+00 | -4.57E-05 | 2.00E+00 | 3.0969 | 3.06 | 0.04 |
| 18 | 3.99E+00 | -7.04E-05 | 1.99E+00 | 2.4437 | 2.46 | -0.01 |
| 19 | 4.01E+00 | 7.65E-05 | 1.96E+00 | 2.5528 | 2.55 | 0.00 |
| 21 | 3.99E+00 | 7.94E-05 | 1.95E+00 | 2.3372 | 2.40 | -0.06 |
| 24 | 3.99E+00 | -6.48E-05 | 1.96E+00 | 2.585 | 2.58 | 0.01 |
| 26 | 4.00E+00 | -5.38E-05 | 2.00E+00 |  | 2.48 |  |
| 27 | 4.00E+00 | -2.93E-05 | 1.92E+00 |  | 2.80 |  |
| 28 | 4.00E+00 | 4.15E-05 | 1.91E+00 |  | 2.73 |  |
| 29 | 4.00E+00 | -4.04E-05 | 2.00E+00 |  | 2.46 |  |
| 30 | 4.00E+00 | -8.67E-06 | 2.00E+00 |  | 2.41 |  |
| 31 | 4.00E+00 | -3.52E-05 | 2.00E+00 |  | 2.45 |  |
| 17 | 3.99E+00 | -6.29E-05 | 1.99E+00 | 2.5229 | 2.44 | 0.08 |
| 33 | 3.99E+00 | 9.64E-05 | 1.78E+00 |  | 3.12 |  |
| 34 | 4.00E+00 | 3.12E-05 | 2.00E+00 |  | 2.35 |  |
| 12 | 3.99E+00 | 5.29E-05 | 2.00E+00 | 2.0605 | 2.22 | -0.16 |
| 37 | 4.00E+00 | 4.58E-05 | 2.00E+00 |  | 2.33 |  |
| 37 | 4.01E+00 | -3.89E-05 | 2.00E+00 |  | 2.56 |  |
| 39 | 4.00E+00 | -4.17E-05 | 2.00E+00 |  | 2.47 |  |
| 5 | 3.99E+00 | -1.12E-05 | 2.00E+00 | 2.3188 | 2.32 | 0.00 |
| 22 | 3.99E+00 | -8.25E-05 | 1.96E+00 | 2.6778 | 2.61 | 0.07 |
| 3 | 3.99E+00 | -3.07E-05 | 2.00E+00 | 2.1612 | 2.35 | -0.19 |
| 14 | 3.99E+00 | 5.35E-06 | 1.98E+00 | 2.4815 | 2.38 | 0.10 |
| 35 | 4.00E+00 | 9.59E-05 | 2.00E+00 | 2.2596 | 2.25 | 0.01 |
| 36 | 3.99E+00 | -3.85E-05 | 2.00E+00 | 2.1938 | 2.36 | -0.17 |
| 2 | 3.99E+00 | 9.75E-05 | 2.00E+00 | 2.4089 | 2.15 | 0.26 |
| 10 | 4.00E+00 | -8.91E-06 | 1.99E+00 | 2.3768 | 2.46 | -0.08 |
| 1 | 3.99E+00 | -1.53E-05 | 2.01E+00 | 2.4318 | 2.28 | 0.15 |
| 15* | 3.99E+00 | -5.68E-05 | 1.96E+00 | 2.5229 | 2.57 | -0.04 |
| 23* | 3.99E+00 | 1.45E-04 | 1.96E+00 | 2.2366 | 2.25 | -0.02 |
| 25* | 3.99E+00 | -1.72E-05 | 1.97E+00 | 2.301 | 2.46 | -0.16 |
| 13* | 3.99E+00 | 1.69E-05 | 2.00E+00 | 2.2518 | 2.28 | -0.02 |
| 8# | 4.01E+00 | -8.12E-06 | 1.99E+00 | 2.1549 | 2.55 | -0.40 |
| 32* | 4.00E+00 | 1.93E-04 | 2.00E+00 | 2.0088 | 2.10 | -0.09 |

**Table S59:** Descriptor, experimental and predicted pIC_50_ values and their residuals for test set 2 compounds in cell line based QSAR model against KB

| **No.** | **HC-2TZ** | **Mi1ERN** | **MiNACH** | **Exp.** | **Pred.** | **Res.** |
| --- | --- | --- | --- | --- | --- | --- |
| 103 | 1.86E-04 | -3.08E-04 | 0.195 | 2.9547 | 2.94 | 0.02 |
| 104 | 1.43E-04 | -3.44E-04 | 0.186 | 1.8586 | 2.14 | -0.28 |
| 111 | 1.03E-03 | -4.56E-04 | 0.205 | 2.8996 | 2.80 | 0.10 |
| 118 | 1.47E-03 | -3.17E-04 | 0.19 | 3.7447 | 3.71 | 0.03 |
| 122 | 3.21E-04 | -1.51E-04 | 0.197 | 4.4437 | 4.58 | -0.14 |
| 121 | 1.33E-03 | -3.21E-04 | 0.189 | 3.7447 | 3.51 | 0.24 |
| 114 | 0.00E+00 | -3.03E-04 | 0.19 | 2.8861 | 2.58 | 0.31 |
| 102 | 6.30E-04 | -3.03E-04 | 0.191 | 3.1367 | 3.17 | -0.03 |
| 120 | 2.59E-04 | -3.11E-04 | 0.193 | 3.0458 | 2.88 | 0.17 |
| 116 | 1.49E-03 | -3.11E-04 | 0.193 | 4.0969 | 3.93 | 0.17 |
| 115 | 7.91E-04 | -3.14E-04 | 0.191 | 3.585 | 3.21 | 0.38 |
| 117 | 1.50E-03 | -3.42E-04 | 0.193 | 3.0809 | 3.66 | -0.58 |
| 108 | 0.00E+00 | -3.35E-04 | 1.94E-01 | 2.0315 | 2.48 | -0.45 |
| 107 | 6.97E-04 | -3.14E-04 | 0.192 | 3.2596 | 3.18 | 0.08 |
| 106 | 2.17E-04 | -2.97E-04 | 0.197 | 3.0655 | 3.16 | -0.10 |
| 113 | 1.28E-04 | -3.19E-04 | 0.195 | 2.7122 | 2.79 | -0.08 |
| 112 | 0.00E+00 | -3.02E-04 | 0.197 | 3.1805 | 2.93 | 0.25 |
| 110* | 1.36E-03 | -3.48E-04 | 0.2 | 3.9586 | 3.82 | 0.14 |
| 105* | 7.48E-04 | -3.20E-04 | 0.186 | 2.061 | 2.87 | -0.81 |
| 109* | 8.70E-04 | -3.31E-04 | 0.195 | 2.9508 | 3.31 | -0.36 |
| 119* | 1.29E-03 | -2.97E-04 | 0.192 | 3.7447 | 3.84 | -0.09 |

**Table S60:** Descriptor, experimental and predicted pIC_50_ values and their residuals for test set 2 compounds in cell line based QSAR model against Kbvin

| **No.** | **HC-1/T** | **PP/SD** | **MaNACC** | **Exp.** | **Pred.** | **Res.** |
| --- | --- | --- | --- | --- | --- | --- |
| 104 | 1.93E-04 | 1.97E-03 | 9.46E-01 | 3.024 | 3.01 | 0.01 |
| 107 | 2.61E-03 | 2.82E-03 | 3.17E-01 | 3.499 | 3.50 | 0.00 |
| 110 | 9.60E-03 | 2.82E-03 | 5.77E-01 | 3.239 | 3.26 | -0.02 |
| 111 | 6.42E-03 | 2.10E-03 | 8.40E-01 | 3.076 | 3.07 | 0.01 |
| 112 | 0.00E+00 | 1.59E-03 | 3.18E-01 | 3.497 | 3.52 | -0.03 |
| 114 | 0.00E+00 | 2.26E-03 | 8.34E-01 | 3.079 | 3.10 | -0.02 |
| 115 | 2.50E-03 | 2.82E-03 | 3.17E-01 | 3.499 | 3.50 | 0.00 |
| 116 | 8.05E-03 | 1.68E-03 | 3.17E-01 | 3.499 | 3.48 | 0.02 |
| 117 | 9.11E-03 | 2.81E-03 | 5.81E-01 | 3.236 | 3.26 | -0.02 |
| 119 | 6.16E-03 | 1.68E-03 | 3.17E-01 | 3.499 | 3.49 | 0.01 |
| 120 | 6.38E-04 | 1.58E-03 | 9.40E-01 | 3.027 | 3.02 | 0.01 |
| 122 | 5.60E-04 | 5.31E-03 | 3.16E-01 | 3.5 | 3.49 | 0.01 |
| 121 | 5.58E-03 | 1.48E-03 | 3.16E-01 | 3.5 | 3.50 | 0.00 |
| 102 | 2.14E-03 | 2.82E-03 | 3.17E-01 | 3.499 | 3.50 | 0.00 |
| 105 | 3.03E-03 | 2.81E-03 | 3.17E-01 | 3.499 | 3.50 | 0.00 |
| 109 | 5.93E-03 | 2.81E-03 | 3.18E-01 | 3.497 | 3.48 | 0.01 |
| 118 | 6.75E-03 | 1.52E-03 | 3.17E-01 | 3.499 | 3.49 | 0.01 |
| 103* | 3.24E-04 | 1.92E-03 | 9.44E-01 | 3.025 | 3.01 | 0.01 |
| 106* | 4.47E-04 | 1.64E-03 | 9.46E-01 | 3.024 | 3.01 | 0.01 |
| 108* | 0.00E+00 | 2.38E-03 | 1.14E+00 | 2.943 | 2.85 | 0.09 |
| 113* | 9.96E-05 | 2.05E-03 | 8.35E-01 | 3.078 | 3.10 | -0.02 |

**Table S61:** Descriptor, experimental and predicted pIC_50_ values and their residuals for test set 2 compounds in cell line based QSAR model against LNCaP

| **No.** | **ZXS** | **SIC0** | **RNN** | **Exp.** | **Pred.** | **Res.** |
| --- | --- | --- | --- | --- | --- | --- |
| 193 | 1.58E+02 | 2.13E+01 | 3.70E-02 | 3 | 2.42 | 0.58 |
| 161 | 1.72E+02 | 2.34E+01 | 2.17E-02 | 2.796 | 3.17 | -0.37 |
| 167 | 1.70E+02 | 2.27E+01 | 2.33E-02 | 3.097 | 2.90 | 0.19 |
| 169 | 1.50E+02 | 2.04E+01 | 2.56E-02 | 2.301 | 2.66 | -0.36 |
| 170 | 1.56E+02 | 2.04E+01 | 2.56E-02 | 2.959 | 2.42 | 0.54 |
| 172 | 1.47E+02 | 2.13E+01 | 3.70E-02 | 2.482 | 2.87 | -0.38 |
| 199 | 1.75E+02 | 2.25E+01 | 3.33E-02 | 2.745 | 2.34 | 0.40 |
| 175 | 9.84E+01 | 1.84E+01 | 5.26E-02 | 2.244 | 3.17 | -0.93 |
| 176 | 1.03E+02 | 1.84E+01 | 5.26E-02 | 2.013 | 2.98 | -0.97 |
| 179 | 1.16E+02 | 1.72E+01 | 5.66E-02 | 2.456 | 1.82 | 0.63 |
| 180 | 9.56E+01 | 1.76E+01 | 8.51E-02 |  | 2.06 |  |
| 182 | 1.56E+02 | 2.27E+01 | 3.41E-02 | 2.699 | 3.18 | -0.48 |
| 184 | 1.58E+02 | 1.99E+01 | 2.56E-02 | 2.854 | 2.12 | 0.74 |
| 185 | 1.64E+02 | 2.29E+01 | 3.49E-02 | 1.975 | 2.92 | -0.95 |
| 186 | 1.51E+02 | 2.17E+01 | 2.50E-02 |  | 3.20 |  |
| 187 | 1.35E+02 | 1.75E+01 | 2.67E-02 | 2.409 | 1.99 | 0.42 |
| 189 | 1.66E+02 | 2.28E+01 | 3.66E-02 | 2.319 | 2.75 | -0.43 |
| 190 | 1.63E+02 | 2.24E+01 | 3.70E-02 | 2.699 | 2.69 | 0.01 |
| 191 | 1.61E+02 | 2.17E+01 | 5.00E-02 | 2.745 | 2.12 | 0.63 |
| 194 | 4.16E+01 | 1.13E+01 | 2.61E-01 |  | -3.21 |  |
| 229 | 8.30E+01 | 1.78E+01 | 4.17E-02 |  | 3.83 |  |
| 230 | 1.06E+02 | 2.13E+01 | 3.33E-02 |  | 4.64 |  |
| 231 | 7.83E+01 | 1.77E+01 | 4.35E-02 |  | 3.93 |  |
| 233 | 7.98E+01 | 1.68E+01 | 4.55E-02 |  | 3.43 |  |
| 235 | 5.77E+01 | 9.20E+00 | 3.33E-02 |  | 1.36 |  |
| 236 | 6.49E+01 | 1.20E+01 | 2.94E-02 |  | 2.39 |  |
| 237 | 6.60E+01 | 1.20E+01 | 2.94E-02 |  | 2.34 |  |
| 238 | 7.49E+01 | 1.20E+01 | 2.94E-02 |  | 1.98 |  |
| 239 | 7.13E+01 | 1.39E+01 | 2.63E-02 |  | 3.03 |  |
| 234 | 7.17E+01 | 1.55E+01 | 2.38E-02 | 4.553 | 3.78 | 0.78 |
| 240 | 8.15E+01 | 1.39E+01 | 2.63E-02 | 3.491 | 2.62 | 0.87 |
| 241 | 5.51E+01 | 1.02E+01 | 3.33E-02 |  | 1.90 |  |
| 242 | 1.12E+02 | 1.82E+01 | 1.47E-02 |  | 3.55 |  |
| 243 | 7.70E+01 | 1.71E+01 | 2.13E-02 | 5.222 | 4.32 | 0.90 |
| 247 | 7.98E+01 | 1.89E+01 | 1.92E-02 | 4.42 | 5.05 | -0.63 |
| 257 | 9.84E+01 | 1.62E+01 | 0.00E+00 |  | 3.64 |  |
| 258 | 1.03E+02 | 1.70E+01 | 0.00E+00 |  | 3.79 |  |
| 250 | 7.90E+01 | 1.94E+01 | 2.13E-02 | 4.027 | 5.24 | -1.21 |
| 251 | 7.87E+01 | 1.75E+01 | 2.27E-02 | 4.357 | 4.39 | -0.03 |
| 253 | 6.86E+01 | 1.69E+01 | 4.65E-02 |  | 3.90 |  |
| 254 | 6.91E+01 | 1.72E+01 | 7.14E-02 | 2.551 | 3.34 | -0.79 |
| 255 | 6.95E+01 | 1.66E+01 | 2.44E-02 | 4.658 | 4.33 | 0.33 |
| 256 | 5.42E+01 | 1.45E+01 | 2.94E-02 |  | 3.91 |  |
| 214 | 8.66E+01 | 2.32E+01 | 5.80E-02 | 6 | 5.59 | 0.42 |
| 228 | 9.33E+01 | 1.77E+01 | 5.56E-02 | 2.456 | 2.99 | -0.54 |
| 200 | 5.90E+01 | 1.39E+01 | 2.63E-02 |  | 3.54 |  |
| 201 | 7.00E+01 | 1.53E+01 | 2.08E-02 | 2.854 | 3.84 | -0.99 |
| 202 | 5.84E+01 | 1.47E+01 | 5.41E-02 | 2.187 | 3.16 | -0.97 |
| 205 | 6.76E+01 | 1.55E+01 | 4.35E-02 | 2.745 | 3.41 | -0.67 |
| 206 | 7.08E+01 | 1.92E+01 | 5.36E-02 | 5.398 | 4.61 | 0.78 |
| 208 | 7.62E+01 | 2.11E+01 | 4.92E-02 | 6 | 5.34 | 0.66 |
| 216 | 7.50E+01 | 1.92E+01 | 5.36E-02 | 3.222 | 4.44 | -1.22 |
| 217 | 8.11E+01 | 1.92E+01 | 5.36E-02 |  | 4.19 |  |
| 207 | 6.78E+01 | 2.13E+01 | 6.56E-02 | 6 | 5.32 | 0.68 |
| 209 | 8.30E+01 | 2.29E+01 | 6.06E-02 |  | 5.53 |  |
| 211 | 8.61E+01 | 2.34E+01 | 5.88E-02 | 6 | 5.67 | 0.33 |
| 213 | 8.30E+01 | 2.35E+01 | 7.14E-02 |  | 5.50 |  |
| 219 | 6.20E+01 | 2.02E+01 | 7.27E-02 | 5 | 4.89 | 0.11 |
| 222 | 6.05E+01 | 1.76E+01 | 6.38E-02 |  | 4.07 |  |
| 223 | 6.11E+01 | 1.88E+01 | 5.88E-02 | 5.699 | 4.70 | 1.00 |
| 224 | 6.82E+01 | 1.88E+01 | 5.88E-02 | 5.699 | 4.41 | 1.29 |
| 225 | 6.03E+01 | 1.85E+01 | 7.84E-02 | 5.523 | 4.07 | 1.45 |
| 218 | 8.83E+01 | 2.39E+01 | 5.56E-02 | 5.398 | 5.88 | -0.49 |
| 220 | 6.28E+01 | 1.97E+01 | 7.14E-02 | 4.125 | 4.68 | -0.55 |
| 221 | 6.18E+01 | 1.91E+01 | 7.55E-02 | 3.62 | 4.35 | -0.73 |
| 226 | 6.22E+01 | 1.92E+01 | 7.69E-02 | 3.959 | 4.34 | -0.38 |
| 162 | 1.74E+02 | 2.38E+01 | 2.22E-02 | 3.1549 | 3.25 | -0.09 |
| 163 | 1.72E+02 | 2.38E+01 | 2.22E-02 | 3.2218 | 3.33 | -0.11 |
| 198 | 1.10E+02 | 1.73E+01 | 3.23E-02 | 2.699 | 2.77 | -0.07 |
| 197 | 1.00E+02 | 1.64E+01 | 3.57E-02 | 2.8539 | 2.69 | 0.16 |
| 196 | 9.89E+01 | 1.64E+01 | 3.57E-02 | 2.6576 | 2.74 | -0.08 |
| 195 | 8.99E+01 | 1.56E+01 | 4.00E-02 | 2.5686 | 2.64 | -0.07 |
| 166 | 1.69E+02 | 2.23E+01 | 2.27E-02 | 2.8861 | 2.79 | 0.10 |
| 168 | 1.56E+02 | 2.04E+01 | 2.56E-02 | 2.4437 | 2.42 | 0.03 |
| 173 | 1.51E+02 | 2.13E+01 | 3.70E-02 | 2.8539 | 2.70 | 0.15 |
| 178 | 9.74E+01 | 1.75E+01 | 5.45E-02 | 2.7696 | 2.77 | 0.00 |
| 183 | 1.45E+02 | 1.99E+01 | 2.56E-02 | 2.7959 | 2.65 | 0.15 |
| 245 | 7.29E+01 | 1.75E+01 | 2.27E-02 | 4.9586 | 4.63 | 0.33 |
| 249 | 7.23E+01 | 1.86E+01 | 4.44E-02 | 4.8239 | 4.54 | 0.28 |
| 181 | 1.50E+02 | 2.15E+01 | 3.80E-02 | 2.7447 | 2.80 | -0.06 |
| 177 | 9.59E+01 | 1.77E+01 | 5.56E-02 | 2.4559 | 2.89 | -0.43 |
| 192* | 1.58E+02 | 1.96E+01 | 2.50E-02 | 2.377 | 2.00 | 0.37 |
| 174* | 1.10E+02 | 1.55E+01 | 1.56E-02 | 2.721 | 2.44 | 0.28 |
| 165* | 1.69E+02 | 2.23E+01 | 2.27E-02 | 2.62 | 2.79 | -0.17 |
| 188* | 1.47E+02 | 2.04E+01 | 3.66E-02 | 2.328 | 2.49 | -0.16 |
| 244* | 7.26E+01 | 1.75E+01 | 2.27E-02 | 4.276 | 4.64 | -0.36 |
| 248* | 7.94E+01 | 1.89E+01 | 4.35E-02 | 4.409 | 4.41 | 0.00 |
| 246* | 7.98E+01 | 1.89E+01 | 1.92E-02 | 5.222 | 5.05 | 0.18 |
| 252 | 7.94E+01 | 1.78E+01 | 2.00E-02 | 4.222 | 4.56 | -0.34 |
| 227* | 1.32E+02 | 2.13E+01 | 3.70E-02 | 3 | 3.48 | -0.48 |
| 203* | 6.90E+01 | 1.62E+01 | 4.26E-02 | 4.114 | 3.68 | 0.43 |
| 204* | 6.83E+01 | 1.55E+01 | 4.35E-02 | 3.699 | 3.39 | 0.31 |
| 210* | 8.76E+01 | 2.14E+01 | 4.76E-02 | 5.097 | 5.04 | 0.05 |
| 212* | 7.87E+01 | 2.19E+01 | 6.15E-02 | 5.046 | 5.25 | -0.20 |
| 215* | 8.91E+01 | 2.47E+01 | 6.76E-02 | 6 | 5.87 | 0.13 |
| 232# | 8.31E+01 | 1.77E+01 | 4.35E-02 | 1.7747 | 3.74 | -1.96 |

**Table S62:** Descriptor, experimental and predicted pIC_50_ values and their residuals for test set 2 compounds in cell line based QSAR model against LoVo

| **No.** | **MaERC** | **MaBO** | **MaVH** | **Exp.** | **Pred** | **Res.** |
| --- | --- | --- | --- | --- | --- | --- |
| 4 | 1.56E-02 | 1.67E+00 | 9.70E-01 |  | 2.33 |  |
| 1 | 2.43E-02 | 1.67E+00 | 9.69E-01 | 3.398 | 3.39 | 0.00 |
| 6 | 1.45E-02 | 1.70E+00 | 9.70E-01 |  | 2.28 |  |
| 8 | 1.55E-02 | 1.68E+00 | 9.70E-01 | 2.319 | 2.34 | -0.02 |
| 9 | 1.55E-02 | 1.75E+00 | 9.70E-01 |  | 2.51 |  |
| 12 | 1.55E-02 | 1.70E+00 | 9.70E-01 | 2.347 | 2.39 | -0.04 |
| 13 | 1.53E-02 | 1.69E+00 | 9.70E-01 | 2.432 | 2.35 | 0.09 |
| 14 | 1.59E-02 | 1.67E+00 | 9.70E-01 | 2.387 | 2.37 | 0.02 |
| 18 | 1.50E-02 | 1.68E+00 | 9.70E-01 | 2.114 | 2.29 | -0.17 |
| 17 | 1.51E-02 | 1.70E+00 | 9.70E-01 | 2.678 | 2.35 | 0.33 |
| 20 | 1.68E-02 | 1.67E+00 | 9.69E-01 | 2.377 | 2.54 | -0.17 |
| 19 | 1.55E-02 | 1.67E+00 | 9.70E-01 | 2.699 | 2.32 | 0.38 |
| 21 | 1.55E-02 | 1.67E+00 | 9.70E-01 | 2.745 | 2.32 | 0.43 |
| 26 | 1.57E-02 | 1.66E+00 | 9.71E-01 | 2.081 | 2.24 | -0.16 |
| 23 | 1.55E-02 | 1.67E+00 | 9.70E-01 | 2.357 | 2.32 | 0.04 |
| 24 | 1.55E-02 | 1.67E+00 | 9.70E-01 | 2.495 | 2.32 | 0.18 |
| 27 | 1.63E-02 | 1.67E+00 | 9.72E-01 |  | 2.26 |  |
| 29 | 1.59E-02 | 1.66E+00 | 9.70E-01 | 2.301 | 2.34 | -0.04 |
| 30 | 1.63E-02 | 1.67E+00 | 9.70E-01 | 2.387 | 2.41 | -0.02 |
| 31 | 1.78E-02 | 1.67E+00 | 9.70E-01 |  | 2.58 |  |
| 32 | 1.61E-02 | 1.67E+00 | 9.70E-01 | 2.319 | 2.39 | -0.07 |
| 33 | 1.61E-02 | 1.66E+00 | 9.70E-01 |  | 2.36 |  |
| 35 | 1.58E-02 | 1.66E+00 | 9.70E-01 | 2.398 | 2.33 | 0.07 |
| 36 | 1.58E-02 | 1.67E+00 | 9.70E-01 | 2.398 | 2.35 | 0.05 |
| 37 | 1.57E-02 | 1.67E+00 | 9.70E-01 |  | 2.34 |  |
| 39 | 1.63E-02 | 1.67E+00 | 9.69E-01 |  | 2.49 |  |
| 2 | 1.70E-02 | 1.67E+00 | 9.70E-01 | 2.569 | 2.49 | 0.08 |
| 15 | 1.64E-02 | 1.75E+00 | 9.70E-01 | 2.538 | 2.61 | -0.08 |
| 28 | 1.52E-02 | 1.66E+00 | 9.70E-01 | 2.076 | 2.26 | -0.19 |
| 34 | 1.48E-02 | 1.67E+00 | 9.70E-01 | 2.092 | 2.24 | -0.15 |
| 32 | 1.57E-02 | 1.67E+00 | 9.70E-01 | 2.061 | 2.34 | -0.28 |
| 11 | 1.49E-02 | 1.69E+00 | 9.70E-01 | 2.194 | 2.30 | -0.11 |
| 7 | 1.55E-02 | 1.68E+00 | 9.70E-01 | 2 | 2.34 | -0.34 |
| 25 | 1.60E-02 | 1.67E+00 | 9.70E-01 | 2.553 | 2.38 | 0.18 |
| 3* | 1.51E-02 | 1.66E+00 | 9.70E-01 | 2.328 | 2.25 | 0.08 |
| 5* | 1.55E-02 | 1.71E+00 | 9.79E-01 | 2.181 | 1.73 | 0.45 |
| 16* | 1.63E-02 | 1.75E+00 | 9.69E-01 | 3.046 | 2.68 | 0.37 |
| 22* | 1.56E-02 | 1.77E+00 | 9.70E-01 | 2.824 | 2.57 | 0.25 |
| 10# | 1.54E-02 | 1.68E+00 | 9.70E-01 | 2.886 | 2.33 | 0.55 |

**Table S63:** Descriptor, experimental and predicted pIC_50_ values and their residuals for test set 2 compounds in cell line based QSAR model against MB231

| **No.** | **YZS** | **FBCSQ** | **MaPBO** | **Exp.** | **Pred.** | **Res.** |
| --- | --- | --- | --- | --- | --- | --- |
| 42 | 3.14E+01 | 2.14E-01 | 9.37E-01 |  | 2.63 |  |
| 43 | 3.86E+01 | 2.26E-01 | 9.42E-01 |  | 2.48 |  |
| 46 | 5.63E+01 | 2.28E-01 | 9.93E-01 |  | 2.02 |  |
| 47 | 5.24E+01 | 2.04E-01 | 9.92E-01 | 2.23 | 2.01 | 0.22 |
| 48 | 5.15E+01 | 2.29E-01 | 9.89E-01 | 2.12 | 2.17 | -0.05 |
| 50 | 4.41E+01 | 2.39E-01 | 9.91E-01 | 2.39 | 2.44 | -0.05 |
| 53 | 5.01E+01 | 2.36E-01 | 9.88E-01 |  | 2.24 |  |
| 54 | 5.85E+01 | 2.36E-01 | 9.89E-01 | 2.49 | 1.99 | 0.50 |
| 55 | 5.08E+01 | 2.37E-01 | 9.89E-01 |  | 2.23 |  |
| 56 | 4.25E+01 | 2.40E-01 | 9.91E-01 | 2.17 | 2.50 | -0.33 |
| 59 | 4.60E+01 | 2.52E-01 | 9.95E-01 | 2.24 | 2.46 | -0.22 |
| 61 | 5.24E+01 | 3.11E-01 | 9.43E-01 |  | 2.52 |  |
| 62 | 5.45E+01 | 2.88E-01 | 9.40E-01 |  | 2.33 |  |
| 63 | 5.51E+01 | 2.01E-01 | 9.95E-01 | 1.87 | 1.92 | -0.05 |
| 66 | 5.31E+01 | 2.08E-01 | 9.87E-01 | 1.66 | 2.00 | -0.34 |
| 68 | 6.10E+01 | 2.70E-01 | 9.96E-01 |  | 2.11 |  |
| 70 | 5.24E+01 | 2.95E-01 | 9.92E-01 | 1.65 | 2.49 | -0.84 |
| 71 | 5.25E+01 | 2.35E-01 | 9.91E-01 | 2.14 | 2.17 | -0.03 |
| 269 | 5.01E+01 | 3.54E-01 | 8.43E-01 |  | 2.70 |  |
| 275 | 4.39E+01 | 2.69E-01 | 8.43E-01 |  | 2.44 |  |
| 262 | 4.91E+01 | 4.87E-01 | 8.44E-01 | 3.55 | 3.43 | 0.12 |
| 263 | 5.17E+01 | 4.90E-01 | 8.35E-01 | 3.68 | 3.36 | 0.32 |
| 264 | 4.53E+01 | 3.59E-01 | 1.98E+00 | 4.15 | 4.19 | -0.04 |
| 265 | 4.80E+01 | 3.54E-01 | 1.98E+00 | 3.85 | 4.09 | -0.24 |
| 266 | 4.45E+01 | 3.93E-01 | 9.94E-01 | 3.92 | 3.25 | 0.67 |
| 268 | 4.75E+01 | 4.77E-01 | 8.43E-01 | 3.64 | 3.43 | 0.21 |
| 270 | 5.15E+01 | 4.54E-01 | 8.45E-01 | 3.7 | 3.19 | 0.51 |
| 273 | 4.98E+01 | 4.04E-01 | 8.56E-01 |  | 2.99 |  |
| 276 | 5.18E+01 | 3.97E-01 | 8.43E-01 |  | 2.88 |  |
| 277 | 4.96E+01 | 3.86E-01 | 8.56E-01 |  | 2.90 |  |
| 276 | 5.13E+01 | 3.76E-01 | 8.43E-01 |  | 2.78 |  |
| 280 | 5.32E+01 | 4.71E-01 | 8.37E-01 | 2.45 | 3.22 | -0.77 |
| 281 | 4.84E+01 | 3.67E-01 | 8.43E-01 |  | 2.82 |  |
| 282 | 4.84E+01 | 4.70E-01 | 8.41E-01 | 3.64 | 3.36 | 0.28 |
| 283 | 4.83E+01 | 2.31E-01 | 8.43E-01 | 2.32 | 2.10 | 0.22 |
| 285 | 4.72E+01 | 2.36E-01 | 8.43E-01 | 2.02 | 2.16 | -0.14 |
| 287 | 4.73E+01 | 2.74E-01 | 9.97E-01 | 1.98 | 2.54 | -0.56 |
| 288 | 4.84E+01 | 3.85E-01 | 9.97E-01 | 3.77 | 3.09 | 0.68 |
| 289 | 4.49E+01 | 2.49E-01 | 1.99E+00 | 3.92 | 3.64 | 0.28 |
| 290 | 4.66E+01 | 4.96E-01 | 8.43E-01 |  | 3.56 |  |
| 291 | 5.61E+01 | 4.72E-01 | 8.45E-01 |  | 3.14 |  |
| 292 | 4.56E+01 | 4.01E-01 | 8.47E-01 |  | 3.09 |  |
| 293 | 4.73E+01 | 3.96E-01 | 8.38E-01 |  | 3.00 |  |
| 294 | 5.04E+01 | 3.50E-01 | 9.94E-01 |  | 2.84 |  |
| 295 | 5.00E+01 | 3.97E-01 | 8.56E-01 |  | 2.94 |  |
| 72 | 4.76E+01 | 2.72E-01 | 9.91E-01 | 1.93 | 2.51 | -0.58 |
| 278 | 5.09E+01 | 4.86E-01 | 8.47E-01 | 2.28 | 3.38 | -1.10 |
| 51 | 4.97E+01 | 2.53E-01 | 9.91E-01 | 2.49 | 2.35 | 0.14 |
| 57 | 5.37E+01 | 2.17E-01 | 9.88E-01 | 2.54 | 2.04 | 0.51 |
| 67 | 6.99E+01 | 2.39E-01 | 9.93E-01 | 1.8 | 1.67 | 0.13 |
| 69 | 5.60E+01 | 2.65E-01 | 9.92E-01 | 1.93 | 2.22 | -0.29 |
| 271 | 5.11E+01 | 4.67E-01 | 8.44E-01 | 3.64 | 3.27 | 0.37 |
| 284 | 4.87E+01 | 3.41E-01 | 8.42E-01 | 2.53 | 2.67 | -0.14 |
| 73 | 5.08E+01 | 2.43E-01 | 9.90E-01 | 1.84 | 2.26 | -0.42 |
| 52 | 4.47E+01 | 2.40E-01 | 9.91E-01 | 2.96 | 2.43 | 0.53 |
| 65 | 6.74E+01 | 2.22E-01 | 9.94E-01 | 1.97 | 1.66 | 0.31 |
| 272 | 4.42E+01 | 2.72E-01 | 8.56E-01 | 2.45 | 2.46 | -0.01 |
| 286 | 4.98E+01 | 3.49E-01 | 8.38E-01 | 2.39 | 2.68 | -0.29 |
| 261 | 4.01E+01 | 2.66E-01 | 8.51E-01 | 3.03 | 2.54 | 0.49 |
| 40* | 4.61E+01 | 2.35E-01 | 9.91E-01 | 2.07 | 2.36 | -0.29 |
| 44* | 5.71E+01 | 2.37E-01 | 9.92E-01 | 1.63 | 2.04 | -0.41 |
| 45* | 5.10E+01 | 2.38E-01 | 9.91E-01 | 2.77 | 2.23 | 0.54 |
| 49* | 5.93E+01 | 2.30E-01 | 9.89E-01 | 2.39 | 1.94 | 0.45 |
| 58* | 5.56E+01 | 2.21E-01 | 9.89E-01 | 2.33 | 2.00 | 0.33 |
| 60* | 4.58E+01 | 2.62E-01 | 9.95E-01 | 2.8 | 2.52 | 0.28 |
| 64* | 5.47E+01 | 2.11E-01 | 9.94E-01 | 1.94 | 1.98 | -0.04 |
| 74* | 4.70E+01 | 1.59E-01 | 9.80E-01 | 1.68 | 1.92 | -0.24 |
| 41* | 5.93E+01 | 2.44E-01 | 9.92E-01 | 2.01 | 2.01 | 0.00 |
| 267* | 4.72E+01 | 3.52E-01 | 8.51E-01 | 2.63 | 2.79 | -0.16 |
| 274# | 5.02E+01 | 4.96E-01 | 8.44E-01 | 2.25 | 3.45 | -1.20 |

**Table S64:** Descriptor, experimental and predicted pIC_50_ values and their residuals for test set 2 compounds in cell line based QSAR model against MB468

| **No.** | **RPCSZ** | **RNCSZ** | **MiBOH** | **Exp.** | **Pred.** | **Res.** |
| --- | --- | --- | --- | --- | --- | --- |
| 40 | 8.70E-02 | 1.65E+01 | 7.58E-01 | 1.98 | 2.38 | -0.40 |
| 42 | 4.26E+00 | 4.62E+00 | 8.08E-01 |  | 1.19 |  |
| 43 | 3.87E+00 | 4.85E+00 | 8.08E-01 |  | 1.27 |  |
| 44 | 0.00E+00 | 3.39E+00 | 7.58E-01 |  | 2.20 |  |
| 46 | 0.00E+00 | 7.95E-01 | 7.69E-01 |  | 2.12 |  |
| 49 | 8.65E-02 | 1.34E+01 | 7.70E-01 | 2.64 | 2.29 | 0.35 |
| 51 | 1.61E-01 | 1.43E+01 | 7.57E-01 | 2.52 | 2.34 | 0.18 |
| 52 | 8.39E-02 | 1.50E+01 | 7.57E-01 | 2.47 | 2.37 | 0.11 |
| 53 | 1.69E-01 | 1.21E+01 | 7.66E-01 |  | 2.27 |  |
| 54 | 1.62E-01 | 1.17E+01 | 7.69E-01 | 2.33 | 2.25 | 0.08 |
| 55 | 2.36E-01 | 1.05E+01 | 7.68E-01 |  | 2.22 |  |
| 57 | 1.93E-01 | 1.41E+01 | 7.67E-01 | 2.21 | 2.29 | -0.08 |
| 58 | 1.85E-01 | 1.37E+01 | 7.68E-01 | 1.93 | 2.28 | -0.35 |
| 61 | 1.98E+00 | 9.15E+00 | 7.69E-01 |  | 1.85 |  |
| 62 | 2.01E+00 | 8.36E+00 | 7.69E-01 |  | 1.84 |  |
| 63 | 0.00E+00 | 1.29E+01 | 8.96E-01 | 1.8 | 1.83 | -0.03 |
| 65 | 0.00E+00 | 3.38E+00 | 8.96E-01 | 1.73 | 1.69 | 0.05 |
| 66 | 1.91E-01 | 3.12E+00 | 8.95E-01 | 1.68 | 1.65 | 0.03 |
| 68 | 3.65E+00 | 6.01E+00 | 7.17E-01 |  | 1.67 |  |
| 67 | 0.00E+00 | 8.29E-01 | 8.75E-01 | 1.69 | 1.72 | -0.03 |
| 72 | 1.50E+00 | 8.88E+00 | 7.27E-01 |  | 2.10 |  |
| 73 | 8.53E-01 | 5.97E+00 | 7.62E-01 | 1.8 | 2.06 | -0.26 |
| 74 | 2.35E+00 | 9.32E-01 | 7.50E-01 | 1.71 | 1.73 | -0.02 |
| 71 | 0.00E+00 | 8.34E-01 | 7.59E-01 | 1.97 | 2.16 | -0.19 |
| 48 | 1.80E-01 | 1.38E+01 | 7.67E-01 | 2.21 | 2.29 | -0.08 |
| 50 | 8.40E-02 | 1.47E+01 | 7.57E-01 | 2.26 | 2.36 | -0.10 |
| 69 | 2.62E+00 | 3.59E+00 | 7.40E-01 | 1.84 | 1.75 | 0.09 |
| 59 | 0.00E+00 | 5.94E+00 | 7.60E-01 | 2.4 | 2.23 | 0.17 |
| 60 | 7.56E-02 | 5.60E+00 | 7.60E-01 | 2.38 | 2.21 | 0.17 |
| 45 | 1.66E-01 | 1.58E+01 | 7.58E-01 | 2.68 | 2.36 | 0.32 |
| 41* | 0.00E+00 | 3.69E+00 | 7.58E-01 | 1.95 | 2.20 | -0.25 |
| 47* | 0.00E+00 | 8.28E+00 | 7.54E-01 | 2.39 | 2.29 | 0.10 |
| 56* | 7.88E-02 | 1.11E+01 | 7.62E-01 | 1.97 | 2.29 | -0.32 |
| 64* | 8.01E-02 | 1.22E+01 | 8.96E-01 | 1.97 | 1.81 | 0.17 |
| 70* | 2.65E+00 | 7.05E+00 | 7.40E-01 | 1.67 | 1.80 | -0.13 |

**Table S65:** Descriptor, experimental and predicted pIC_50_ values and their residuals for test set 2 compounds in cell line based QSAR model against MCF-7

| **No.** | **ZXS/ZXR** | **MiNRO** | **Mi1ERC** | **Exp.** | **Pred.** | **Res.** |
| --- | --- | --- | --- | --- | --- | --- |
| 296 | 5.61E-01 | 3.37E-04 | -1.83E-02 | 5 | 4.36 | 0.65 |
| 297 | 6.05E-01 | 6.00E-07 | -2.38E-02 |  | 4.69 |  |
| 300 | 7.96E-01 | 1.44E-02 | -1.69E-02 | 1.22 | 2.16 | -0.94 |
| 301 | 7.44E-01 | 6.31E-04 | -1.45E-02 |  | 3.04 |  |
| 306 | 6.44E-01 | 7.74E-04 | -1.60E-02 | 3.36 | 3.69 | -0.33 |
| 307 | 6.74E-01 | 1.03E-04 | -1.59E-02 | 3.57 | 3.57 | 0.00 |
| 308 | 6.62E-01 | 6.34E-04 | -1.60E-02 |  | 3.60 |  |
| 309 | 7.09E-01 | 3.58E-07 | -1.61E-02 |  | 3.42 |  |
| 310 | 7.16E-01 | 6.28E-04 | -1.62E-02 |  | 3.35 |  |
| 313 | 6.64E-01 | 3.10E-08 | -1.64E-02 | 4.22 | 3.67 | 0.55 |
| 314 | 6.44E-01 | 6.94E-04 | -1.66E-02 |  | 3.75 |  |
| 317 | 6.81E-01 | 2.35E-07 | -1.63E-02 | 3.55 | 3.58 | -0.03 |
| 318 | 6.42E-01 | 1.63E-03 | -1.63E-02 |  | 3.67 |  |
| 320 | 6.96E-01 | 1.84E-04 | -8.38E-03 | 3.32 | 2.72 | 0.60 |
| 321 | 7.06E-01 | 1.29E-03 | -1.46E-02 |  | 3.21 |  |
| 322 | 6.83E-01 | 1.05E-04 | -1.60E-02 |  | 3.53 |  |
| 323 | 6.45E-01 | 1.40E-03 | -1.52E-02 |  | 3.57 |  |
| 324 | 6.49E-01 | 1.44E-03 | -1.60E-02 |  | 3.62 |  |
| 325 | 6.49E-01 | 1.41E-03 | -1.61E-02 |  | 3.63 |  |
| 327 | 6.74E-01 | 1.07E-04 | -1.62E-02 |  | 3.60 |  |
| 329 | 6.92E-01 | 2.73E-05 | -1.69E-02 | 2.79 | 3.58 | -0.79 |
| 330 | 7.02E-01 | 1.37E-02 | -1.66E-02 |  | 2.65 |  |
| 331 | 6.69E-01 | 1.35E-02 | -1.55E-02 |  | 2.73 |  |
| 334 | 7.05E-01 | 1.25E-04 | -1.52E-02 | 3.64 | 3.34 | 0.30 |
| 335 | 5.74E-01 | 1.48E-02 | -1.41E-02 | 3.72 | 2.99 | 0.73 |
| 336 | 6.67E-01 | 1.40E-02 | -1.58E-02 | 2.78 | 2.73 | 0.05 |
| 337 | 6.40E-01 | 1.42E-02 | -1.59E-02 | 1.96 | 2.87 | -0.91 |
| 338 | 6.70E-01 | 1.04E-04 | -1.57E-02 |  | 3.57 |  |
| 339 | 6.33E-01 | 1.38E-02 | -1.50E-02 |  | 2.84 |  |
| 340 | 6.87E-01 | 1.40E-02 | -1.57E-02 | 3 | 2.62 | 0.38 |
| 341 | 6.60E-01 | 8.53E-05 | -1.57E-02 | 3.43 | 3.62 | -0.19 |
| 342 | 6.96E-01 | 1.47E-02 | -1.52E-02 | 3.26 | 2.49 | 0.77 |
| 2 | 6.34E-01 | 1.75E-04 | -9.37E-04 | 2.24 | 2.32 | -0.08 |
| 4 | 6.46E-01 | 1.12E-06 | -1.08E-03 |  | 2.28 |  |
| 1 | 6.55E-01 | 3.31E-07 | -1.25E-03 |  | 2.25 |  |
| 3 | 6.20E-01 | 1.88E-04 | -1.13E-03 | 2 | 2.41 | -0.41 |
| 7 | 6.73E-01 | 2.17E-06 | -8.84E-04 |  | 2.13 |  |
| 9 | 6.63E-01 | 1.11E-07 | -8.77E-04 |  | 2.18 |  |
| 10 | 6.59E-01 | 2.68E-07 | -1.11E-03 | 2.66 | 2.22 | 0.44 |
| 11 | 6.46E-01 | 1.28E-06 | -8.46E-04 |  | 2.26 |  |
| 12 | 6.75E-01 | 9.97E-07 | -8.49E-04 | 2.02 | 2.11 | -0.09 |
| 13 | 6.09E-01 | 2.06E-06 | -1.08E-03 |  | 2.47 |  |
| 14 | 6.33E-01 | 2.31E-07 | -1.10E-03 |  | 2.35 |  |
| 16 | 6.11E-01 | 2.57E-08 | -1.20E-03 | 2.74 | 2.47 | 0.27 |
| 18 | 6.81E-01 | 9.42E-08 | -1.38E-03 | 2.2 | 2.13 | 0.07 |
| 19 | 6.09E-01 | 5.29E-07 | -8.58E-04 | 2.62 | 2.45 | 0.17 |
| 21 | 6.60E-01 | 1.19E-06 | -8.68E-04 | 2.41 | 2.19 | 0.22 |
| 23 | 6.25E-01 | 1.16E-05 | -2.98E-03 | 2.18 | 2.57 | -0.39 |
| 24 | 6.19E-01 | 2.81E-07 | -1.34E-03 | 2.19 | 2.44 | -0.25 |
| 25 | 4.86E-01 | 4.59E-09 | -1.15E-04 | 2.32 | 3.00 | -0.68 |
| 26 | 7.06E-01 | 4.60E-06 | -1.88E-03 |  | 2.05 |  |
| 27 | 6.65E-01 | 1.72E-06 | -1.58E-03 |  | 2.23 |  |
| 28 | 6.18E-01 | 1.84E-06 | -8.51E-04 |  | 2.40 |  |
| 29 | 6.19E-01 | 9.70E-08 | -1.61E-03 |  | 2.47 |  |
| 30 | 6.96E-01 | 1.81E-04 | -1.31E-03 | 2.13 | 2.04 | 0.09 |
| 31 | 6.75E-01 | 3.04E-07 | -1.56E-03 |  | 2.18 |  |
| 38 | 6.92E-01 | 4.21E-05 | -2.49E-03 |  | 2.18 |  |
| 33 | 6.17E-01 | 1.41E-09 | -9.78E-04 |  | 2.42 |  |
| 34 | 7.10E-01 | 2.38E-07 | -1.50E-03 |  | 2.00 |  |
| 35 | 6.30E-01 | 5.16E-07 | -1.68E-03 | 2.05 | 2.42 | -0.37 |
| 36 | 6.45E-01 | 8.17E-09 | -1.35E-03 | 2.32 | 2.31 | 0.01 |
| 37 | 6.31E-01 | 2.12E-06 | -8.47E-04 |  | 2.33 |  |
| 38 | 6.36E-01 | 1.80E-04 | -1.36E-03 |  | 2.35 |  |
| 39 | 5.68E-01 | 1.75E-04 | -1.36E-03 |  | 2.69 |  |
| 6 | 6.37E-01 | 7.74E-08 | -8.47E-04 |  | 2.30 |  |
| 302 | 6.47E-01 | 6.34E-04 | -1.60E-02 | 3.52 | 3.68 | -0.16 |
| 311 | 6.91E-01 | 1.01E-08 | -1.65E-02 | 3.57 | 3.54 | 0.03 |
| 312 | 6.25E-01 | 7.09E-04 | -1.64E-02 | 3.72 | 3.83 | -0.11 |
| 326 | 6.58E-01 | 1.39E-02 | -1.64E-02 | 2.74 | 2.84 | -0.10 |
| 319 | 6.37E-01 | 1.78E-06 | -1.64E-02 | 4.15 | 3.81 | 0.34 |
| 333 | 6.28E-01 | 1.58E-06 | -1.60E-02 | 3.43 | 3.82 | -0.39 |
| 8 | 6.71E-01 | 1.31E-06 | -1.10E-03 | 2.32 | 2.16 | 0.16 |
| 15 | 6.55E-01 | 5.78E-09 | -1.23E-03 | 2.31 | 2.25 | 0.06 |
| 20 | 5.97E-01 | 1.10E-08 | -1.68E-03 | 2.51 | 2.59 | -0.08 |
| 17 | 6.94E-01 | 1.84E-07 | -1.38E-03 | 2.44 | 2.07 | 0.37 |
| 303* | 6.39E-01 | 6.35E-04 | -1.52E-02 | 3.52 | 3.64 | -0.12 |
| 305* | 6.50E-01 | 2.42E-06 | -1.58E-02 | 3.52 | 3.68 | -0.16 |
| 315* | 7.03E-01 | 6.50E-04 | -1.65E-02 | 2.98 | 3.44 | -0.46 |
| 316* | 6.55E-01 | 1.64E-03 | -1.62E-02 | 3.42 | 3.60 | -0.18 |
| 328* | 6.93E-01 | 1.36E-02 | -1.63E-02 | 2.66 | 2.68 | -0.02 |
| 332* | 6.51E-01 | 1.41E-02 | -1.56E-02 | 2.48 | 2.79 | -0.31 |
| 299* | 6.37E-01 | 9.21E-07 | -1.11E-03 | 2.13 | 2.33 | -0.20 |
| 5* | 6.42E-01 | 5.69E-04 | -2.37E-02 | 5 | 4.45 | 0.55 |
| 22* | 6.32E-01 | 2.59E-08 | -1.44E-03 | 2.51 | 2.39 | 0.12 |
| 298# | 6.42E-01 | 2.65E-06 | -2.36E-02 | 3.43 | 4.48 | -1.05 |

**Table S66:** Descriptor, experimental and predicted pIC_50_ values and their residuals for test set 2 compounds in cell line based QSAR model against OVCR-3

| **No.** | **MSA** | **THCMD** | **MaVO** | **Exp.** | **Pred.** | **Res.** |
| --- | --- | --- | --- | --- | --- | --- |
| 326 | 4.27E+02 | 8.10E-01 | 1.97E+00 | 1.84 | 1.86 | -0.02 |
| 328 | 4.19E+02 | -1.00E+00 | 1.97E+00 | 2.33 | 2.32 | 0.01 |
| 329 | 4.17E+02 | 1.85E-01 | 1.96E+00 | 1.77 | 2.03 | -0.26 |
| 334 | 4.10E+02 | -3.27E+00 | 1.96E+00 | 2.19 | 2.87 | -0.68 |
| 340 | 4.12E+02 | -1.38E+00 | 1.97E+00 | 2.38 | 2.45 | -0.07 |
| 341 | 4.17E+02 | -1.80E+00 | 1.97E+00 | 2.58 | 2.52 | 0.06 |
| 312 | 4.86E+02 | -7.21E-01 | 2.11E+00 | 2.15 | 2.31 | -0.16 |
| 303 | 4.49E+02 | -1.19E+00 | 2.11E+00 | 1.73 | 2.61 | -0.88 |
| 305 | 4.46E+02 | -1.50E+00 | 2.10E+00 | 2.77 | 2.66 | 0.11 |
| 297 | 4.69E+02 | -3.54E+00 | 2.11E+00 | 3.3 | 3.05 | 0.25 |
| 336 | 4.06E+02 | -1.11E+00 | 1.97E+00 | 2.69 | 2.41 | 0.28 |
| 316 | 4.31E+02 | 6.45E-02 | 2.10E+00 | 1.79 | 2.38 | -0.59 |
| 338 | 4.18E+02 | -9.51E-01 | 1.97E+00 |  | 2.32 |  |
| 339 | 4.16E+02 | -1.34E+00 | 1.97E+00 |  | 2.42 |  |
| 318 | 4.12E+02 | 6.01E-01 | 2.10E+00 |  | 2.35 |  |
| 319 | 4.35E+02 | -6.67E-01 | 2.10E+00 |  | 2.53 |  |
| 321 | 4.10E+02 | -1.48E+00 | 2.12E+00 |  | 2.90 |  |
| 322 | 4.61E+02 | -1.04E-01 | 2.11E+00 |  | 2.29 |  |
| 323 | 4.55E+02 | -2.51E+00 | 2.12E+00 |  | 2.91 |  |
| 324 | 4.76E+02 | -1.14E+00 | 2.11E+00 |  | 2.46 |  |
| 325 | 5.03E+02 | -1.24E+00 | 2.12E+00 |  | 2.37 |  |
| 300 | 3.32E+02 | -1.11E+00 | 1.97E+00 |  | 2.79 |  |
| 301 | 4.00E+02 | -9.30E-01 | 2.11E+00 |  | 2.79 |  |
| 308 | 4.75E+02 | -1.12E+00 | 2.11E+00 |  | 2.46 |  |
| 309 | 5.05E+02 | -1.03E+00 | 2.10E+00 |  | 2.26 |  |
| 310 | 4.26E+02 | -8.68E-01 | 2.10E+00 |  | 2.62 |  |
| 314 | 4.55E+02 | -9.07E-02 | 2.10E+00 |  | 2.29 |  |
| 327 | 4.30E+02 | -1.28E+00 | 1.97E+00 |  | 2.33 |  |
| 330 | 3.78E+02 | 5.04E-01 | 1.97E+00 |  | 2.18 |  |
| 331 | 4.52E+02 | -1.88E+00 | 1.97E+00 |  | 2.36 |  |
| 306 | 4.28E+02 | -2.38E-01 | 2.10E+00 | 2.71 | 2.46 | 0.25 |
| 311 | 4.50E+02 | -7.75E-01 | 2.10E+00 | 2.74 | 2.48 | 0.27 |
| 313 | 4.55E+02 | -8.46E-01 | 2.10E+00 | 2.74 | 2.47 | 0.27 |
| 315 | 4.26E+02 | -8.63E-01 | 2.10E+00 | 2.71 | 2.62 | 0.09 |
| 302 | 4.28E+02 | -1.33E+00 | 2.10E+00 | 2.66 | 2.72 | -0.06 |
| 307 | 4.53E+02 | -1.47E+00 | 2.10E+00 | 2.74 | 2.62 | 0.12 |
| 332 | 4.21E+02 | -1.41E+00 | 1.97E+00 | 2.82 | 2.41 | 0.41 |
| 333 | 4.25E+02 | -1.63E+00 | 1.97E+00 | 2.73 | 2.44 | 0.29 |
| 296 | 3.55E+02 | -2.73E+00 | 2.18E+00 | 3.66 | 3.64 | 0.02 |
| 317 | 4.18E+02 | -3.41E-01 | 2.10E+00 | 2.81 | 2.54 | 0.27 |
| 337* | 4.16E+02 | -6.30E-01 | 1.97E+00 | 1.82 | 2.25 | -0.43 |
| 342* | 4.44E+02 | -2.00E+00 | 1.97E+00 | 2.31 | 2.43 | -0.12 |
| 320* | 4.73E+02 | -7.11E-01 | 2.10E+00 | 2.37 | 2.34 | 0.03 |
| 298# | 4.89E+02 | -1.59E+00 | 2.12E+00 | 1.13 | 2.52 | -1.39 |
| 299# | 4.70E+02 | -1.68E+00 | 2.13E+00 | 4.07 | 2.67 | 1.40 |
| 335* | 4.07E+02 | -2.38E+00 | 1.97E+00 | 3.16 | 2.70 | 0.46 |

**Table S67:** Descriptor, experimental and predicted pIC_50_ values and their residuals for test set 2 compounds in cell line based QSAR model against PC-3

| **No.** | **ZXS/ZXR** | **MaPCH** | **FS-2PZ** | **Exp.** | **Pred.** | **Res.** |
| --- | --- | --- | --- | --- | --- | --- |
| 192 | 6.25E-01 | 5.37E-02 | -1.86E-01 | 2 | 2.47 | -0.47 |
| 198 | 7.19E-01 | 5.37E-02 | -1.79E-01 | 2.59 | 2.22 | 0.37 |
| 195 | 7.29E-01 | 5.37E-02 | -1.85E-01 |  | 2.20 |  |
| 199 | 6.66E-01 | 4.62E-02 | -9.87E-02 | 2.54 | 2.89 | -0.35 |
| 165 | 5.68E-01 | 5.37E-02 | -1.64E-01 | 2.39 | 2.57 | -0.18 |
| 166 | 5.68E-01 | 5.37E-02 | -1.64E-01 | 2.59 | 2.57 | 0.02 |
| 168 | 5.90E-01 | 5.37E-02 | -1.82E-01 | 2.13 | 2.55 | -0.42 |
| 169 | 5.89E-01 | 5.37E-02 | -1.92E-01 | 3 | 2.58 | 0.42 |
| 175 | 7.36E-01 | 5.52E-02 | -2.65E-01 |  | 2.22 |  |
| 176 | 7.01E-01 | 5.52E-02 | -2.95E-01 |  | 2.38 |  |
| 179 | 7.11E-01 | 5.63E-02 | -3.71E-01 | 2.12 | 2.42 | -0.30 |
| 180 | 6.18E-01 | 5.89E-02 | -4.07E-01 |  | 2.49 |  |
| 182 | 6.07E-01 | 5.52E-02 | -2.59E-01 | 2.68 | 2.54 | 0.14 |
| 184 | 6.49E-01 | 5.37E-02 | -1.89E-01 | 2.68 | 2.42 | 0.26 |
| 185 | 5.71E-01 | 5.52E-02 | -3.17E-01 | 2.05 | 2.76 | -0.71 |
| 186 | 6.40E-01 | 5.52E-02 | -2.68E-01 |  | 2.47 |  |
| 187 | 6.90E-01 | 4.68E-02 | -1.26E-01 | 2.47 | 2.83 | -0.36 |
| 189 | 6.17E-01 | 6.39E-02 | -2.83E-01 |  | 1.74 |  |
| 191 | 5.98E-01 | 5.84E-02 | -2.78E-01 | 2.6 | 2.30 | 0.30 |
| 194 | 6.81E-01 | 5.83E-02 | -1.98E-01 |  | 1.92 |  |
| 229 | 6.18E-01 | 5.80E-02 | -3.07E-01 |  | 2.35 |  |
| 230 | 6.62E-01 | 5.80E-02 | -2.85E-01 |  | 2.19 |  |
| 231 | 5.42E-01 | 5.83E-02 | -3.04E-01 |  | 2.51 |  |
| 232 | 6.08E-01 | 5.89E-02 | -3.04E-01 | 1.88 | 2.28 | -0.40 |
| 233 | 6.79E-01 | 5.91E-02 | -3.13E-01 |  | 2.10 |  |
| 235 | 7.01E-01 | 3.50E-02 | -1.89E-01 |  | 4.06 |  |
| 236 | 7.46E-01 | 3.50E-02 | -2.32E-01 |  | 4.04 |  |
| 237 | 5.66E-01 | 3.51E-02 | -2.25E-01 |  | 4.48 |  |
| 238 | 7.31E-01 | 3.52E-02 | -2.24E-01 |  | 4.04 |  |
| 239 | 6.92E-01 | 3.51E-02 | -2.37E-01 |  | 4.18 |  |
| 240 | 6.23E-01 | 3.51E-02 | -2.17E-01 | 3.52 | 4.31 | -0.79 |
| 241 | 6.37E-01 | 3.54E-02 | -2.26E-01 |  | 4.27 |  |
| 242 | 8.62E-01 | 3.47E-02 | -1.43E-01 |  | 3.58 |  |
| 243 | 5.39E-01 | 3.57E-02 | -2.31E-01 | 5.3 | 4.50 | 0.80 |
| 245 | 6.37E-01 | 3.67E-02 | -2.83E-01 | 4.62 | 4.27 | 0.35 |
| 246 | 5.29E-01 | 3.66E-02 | -2.25E-01 | 4.89 | 4.43 | 0.46 |
| 248 | 6.39E-01 | 3.62E-02 | -3.67E-01 | 4.25 | 4.50 | -0.25 |
| 257 | 7.80E-01 | 9.76E-02 | -4.36E-01 |  | -1.54 |  |
| 258 | 7.54E-01 | 3.46E-02 | -3.81E-01 |  | 4.39 |  |
| 252 | 6.68E-01 | 3.57E-02 | -2.32E-01 | 4.57 | 4.18 | 0.40 |
| 253 | 5.48E-01 | 3.63E-02 | -2.26E-01 |  | 4.41 |  |
| 254 | 6.94E-01 | 4.15E-02 | -2.39E-01 | 2.58 | 3.57 | -0.99 |
| 255 | 6.91E-01 | 3.73E-02 | -2.84E-01 | 4.77 | 4.08 | 0.69 |
| 256 | 6.97E-01 | 3.35E-02 | -2.04E-01 |  | 4.25 |  |
| 259 | 7.41E-01 | 9.14E-02 | -3.95E-01 |  | -0.94 |  |
| 227 | 6.66E-01 | 5.52E-02 | -2.76E-01 | 2.92 | 2.42 | 0.50 |
| 193 | 5.89E-01 | 5.52E-02 | -2.63E-01 | 2.92 | 2.59 | 0.33 |
| 162 | 5.31E-01 | 5.37E-02 | -1.90E-01 | 2.82 | 2.72 | 0.10 |
| 170 | 6.33E-01 | 5.37E-02 | -1.93E-01 | 2.57 | 2.47 | 0.10 |
| 172 | 6.24E-01 | 5.56E-02 | -2.45E-01 | 2.57 | 2.43 | 0.15 |
| 173 | 6.32E-01 | 5.52E-02 | -2.58E-01 | 2.8 | 2.47 | 0.33 |
| 174 | 6.66E-01 | 5.52E-02 | -2.54E-01 | 2.43 | 2.38 | 0.05 |
| 181 | 5.76E-01 | 5.52E-02 | -2.75E-01 | 2.6 | 2.65 | -0.05 |
| 188 | 5.78E-01 | 5.52E-02 | -2.07E-01 | 2.35 | 2.50 | -0.15 |
| 244 | 6.61E-01 | 3.87E-02 | -2.93E-01 | 4.09 | 4.04 | 0.05 |
| 197 | 7.25E-01 | 5.37E-02 | -1.87E-01 | 2.54 | 2.22 | 0.32 |
| 177 | 6.75E-01 | 5.64E-02 | -3.50E-01 | 2.68 | 2.45 | 0.23 |
| 161 | 5.65E-01 | 5.37E-02 | -1.81E-01 | 2.31 | 2.62 | -0.31 |
| 247 | 5.29E-01 | 3.66E-02 | -2.25E-01 | 4.16 | 4.43 | -0.27 |
| 250 | 6.50E-01 | 3.62E-02 | -4.24E-01 | 4.24 | 4.60 | -0.36 |
| 163* | 5.56E-01 | 5.37E-02 | -1.92E-01 | 2.92 | 2.66 | 0.26 |
| 196* | 7.15E-01 | 5.37E-02 | -1.90E-01 | 2.36 | 2.25 | 0.11 |
| 167* | 5.53E-01 | 5.37E-02 | -1.88E-01 | 2.7 | 2.66 | 0.04 |
| 178* | 5.41E-01 | 5.64E-02 | -3.99E-01 | 3.1 | 2.90 | 0.20 |
| 183* | 6.04E-01 | 5.37E-02 | -1.99E-01 | 2.62 | 2.56 | 0.07 |
| 234* | 5.70E-01 | 3.52E-02 | -2.86E-01 | 4.68 | 4.59 | 0.09 |
| 249* | 6.34E-01 | 3.59E-02 | -3.05E-01 | 4.52 | 4.40 | 0.12 |
| 251* | 5.07E-01 | 3.60E-02 | -3.26E-01 | 4.74 | 4.77 | -0.03 |
| 228* | 5.52E-01 | 5.67E-02 | -3.51E-01 | 2.68 | 2.74 | -0.06 |
| 190* | 6.11E-01 | 5.74E-02 | -3.29E-01 | 3.05 | 2.47 | 0.58 |

**Table S68:** Descriptor, experimental and predicted pIC_50_ values and their residuals for test set 2 compounds in cell line based QSAR model against PPC-1

| **No.** | **PS-1Z** | **RPCGZ** | **MaNACH** | **Exp.** | **Pred.** | **Res.** |
| --- | --- | --- | --- | --- | --- | --- |
| 161 | 1.51E+02 | 4.27E-02 | 4.18E-01 | 3 | 3.23 | -0.23 |
| 163 | 1.60E+02 | 4.33E-02 | 4.18E-01 | 3.52 | 3.24 | 0.28 |
| 195 | 1.92E+02 | 8.81E-02 | 4.09E-01 | 1.85 | 2.56 | -0.71 |
| 199 | 1.15E+02 | 6.83E-02 | 3.92E-01 | 3.15 | 2.98 | 0.17 |
| 166 | 1.48E+02 | 4.83E-02 | 4.18E-01 | 3.22 | 3.12 | 0.10 |
| 167 | 1.72E+02 | 4.90E-02 | 4.18E-01 | 3.52 | 3.15 | 0.37 |
| 169 | 1.95E+02 | 5.78E-02 | 4.18E-01 | 3.7 | 3.03 | 0.67 |
| 170 | 1.94E+02 | 5.78E-02 | 4.18E-01 | 3.4 | 3.03 | 0.37 |
| 173 | 2.44E+02 | 5.47E-02 | 4.30E-01 | 3.3 | 3.04 | 0.26 |
| 175 | 2.21E+02 | 7.03E-02 | 4.32E-01 |  | 2.68 |  |
| 176 | 2.49E+02 | 6.99E-02 | 4.30E-01 |  | 2.76 |  |
| 178 | 4.00E+02 | 7.62E-02 | 4.33E-01 | 3.15 | 2.90 | 0.26 |
| 180 | 3.48E+02 | 7.55E-02 | 4.33E-01 |  | 2.81 |  |
| 181 | 2.64E+02 | 5.56E-02 | 4.30E-01 | 2.96 | 3.06 | -0.10 |
| 184 | 2.06E+02 | 6.29E-02 | 4.19E-01 | 3.22 | 2.94 | 0.28 |
| 185 | 2.92E+02 | 5.51E-02 | 4.31E-01 | 2.17 | 3.11 | -0.94 |
| 186 | 2.55E+02 | 7.51E-02 | 4.31E-01 |  | 2.67 |  |
| 187 | 1.45E+02 | 5.54E-02 | 3.92E-01 | 2.6 | 3.28 | -0.68 |
| 188 | 2.05E+02 | 5.37E-02 | 4.30E-01 | 2.62 | 2.99 | -0.37 |
| 190 | 3.13E+02 | 5.82E-02 | 4.35E-01 | 3.4 | 3.05 | 0.35 |
| 191 | 2.57E+02 | 6.38E-02 | 4.37E-01 | 3.3 | 2.81 | 0.49 |
| 194 | 1.45E+02 | 1.25E-01 | 4.40E-01 |  | 1.41 |  |
| 229 | 2.11E+02 | 6.35E-02 | 4.33E-01 |  | 2.78 |  |
| 230 | 1.59E+02 | 4.42E-02 | 4.32E-01 |  | 3.06 |  |
| 232 | 2.10E+02 | 7.25E-02 | 4.32E-01 | 2.47 | 2.62 | -0.15 |
| 233 | 2.21E+02 | 7.61E-02 | 4.33E-01 |  | 2.56 |  |
| 235 | 2.57E+02 | 1.38E-01 | 2.88E-01 |  | 3.14 |  |
| 236 | 2.63E+02 | 1.05E-01 | 2.87E-01 |  | 3.79 |  |
| 237 | 2.53E+02 | 1.05E-01 | 2.88E-01 |  | 3.76 |  |
| 238 | 2.32E+02 | 1.09E-01 | 2.68E-01 |  | 3.88 |  |
| 239 | 2.17E+02 | 8.26E-02 | 2.88E-01 |  | 4.11 |  |
| 241 | 2.42E+02 | 1.33E-01 | 2.90E-01 |  | 3.19 |  |
| 242 | 1.71E+02 | 6.35E-02 | 2.89E-01 |  | 4.37 |  |
| 243 | 1.76E+02 | 6.25E-02 | 2.76E-01 | 5.22 | 4.55 | 0.67 |
| 244 | 1.95E+02 | 6.39E-02 | 2.84E-01 | 4.28 | 4.47 | -0.19 |
| 246 | 1.45E+02 | 4.87E-02 | 2.76E-01 | 5.1 | 4.76 | 0.35 |
| 247 | 1.45E+02 | 4.87E-02 | 2.76E-01 | 4.25 | 4.76 | -0.51 |
| 248 | 2.78E+02 | 6.02E-02 | 2.80E-01 | 4.47 | 4.74 | -0.27 |
| 257 | 3.19E+02 | 9.96E-02 | 5.06E-01 |  | 1.45 |  |
| 258 | 2.80E+02 | 8.28E-02 | 2.81E-01 |  | 4.31 |  |
| 250 | 2.64E+02 | 1.60E-01 | 2.78E-01 | 3.12 | 2.86 | 0.26 |
| 251 | 2.46E+02 | 6.30E-02 | 2.77E-01 | 4.68 | 4.67 | 0.02 |
| 252 | 1.75E+02 | 6.06E-02 | 2.75E-01 | 4.66 | 4.60 | 0.06 |
| 253 | 1.64E+02 | 6.25E-02 | 2.78E-01 |  | 4.51 |  |
| 256 | 1.36E+02 | 7.07E-02 | 2.75E-01 |  | 4.34 |  |
| 259 | 2.97E+02 | 1.22E-01 | 5.11E-01 |  | 0.93 |  |
| 228 | 3.23E+02 | 7.71E-02 | 4.32E-01 | 2.8 | 2.74 | 0.06 |
| 182 | 2.49E+02 | 5.14E-02 | 4.30E-01 | 3.15 | 3.11 | 0.04 |
| 183 | 2.15E+02 | 6.29E-02 | 4.19E-01 | 3 | 2.96 | 0.04 |
| 197 | 1.95E+02 | 8.12E-02 | 4.09E-01 | 3 | 2.69 | 0.31 |
| 172 | 2.29E+02 | 5.45E-02 | 4.24E-01 | 2.82 | 3.09 | -0.27 |
| 162 | 1.58E+02 | 4.33E-02 | 4.18E-01 | 3.3 | 3.23 | 0.07 |
| 231 | 2.13E+02 | 6.89E-02 | 4.30E-01 | 2.28 | 2.72 | -0.44 |
| 179 | 3.63E+02 | 7.66E-02 | 4.32E-01 | 2.54 | 2.83 | -0.29 |
| 255 | 2.13E+02 | 6.55E-02 | 2.77E-01 | 4.89 | 4.56 | 0.34 |
| 234 | 2.18E+02 | 6.79E-02 | 2.87E-01 | 4.37 | 4.40 | -0.03 |
| 227 | 2.62E+02 | 5.47E-02 | 4.31E-01 | 3.4 | 3.06 | 0.34 |
| 240 | 1.97E+02 | 8.33E-02 | 2.75E-01 | 3.62 | 4.21 | -0.59 |
| 192 | 2.07E+02 | 6.17E-02 | 4.18E-01 | 2.62 | 2.98 | -0.36 |
| 192* | 2.51E+02 | 5.47E-02 | 4.30E-01 | 3.4 | 3.05 | 0.35 |
| 198* | 1.89E+02 | 7.52E-02 | 4.09E-01 | 3.22 | 2.80 | 0.43 |
| 196* | 2.00E+02 | 8.12E-02 | 4.09E-01 | 2.68 | 2.70 | -0.02 |
| 165* | 1.48E+02 | 4.83E-02 | 4.18E-01 | 3.05 | 3.12 | -0.07 |
| 168* | 1.73E+02 | 5.74E-02 | 4.15E-01 | 2.77 | 3.03 | -0.26 |
| 174* | 2.46E+02 | 5.07E-02 | 4.30E-01 | 3.1 | 3.12 | -0.02 |
| 177* | 3.24E+02 | 7.68E-02 | 4.32E-01 | 2.81 | 2.75 | 0.06 |
| 189* | 2.76E+02 | 5.72E-02 | 4.44E-01 | 2.96 | 2.89 | 0.07 |
| 245* | 1.91E+02 | 6.14E-02 | 2.76E-01 | 4.68 | 4.60 | 0.08 |
| 249* | 2.33E+02 | 6.26E-02 | 2.79E-01 | 4.72 | 4.62 | 0.10 |
| 254# | 1.59E+02 | 5.96E-02 | 2.90E-01 | 2.93 | 4.41 | -1.48 |

**Table S69:** Descriptor, experimental and predicted pIC_50_ values and their residuals for test set 2 compounds in cell line based QSAR model against RH7777

| **No.** | **MI-A** | **MaBOC** | **AERN** | **Exp.** | **Pred.** | **Res.** |
| --- | --- | --- | --- | --- | --- | --- |
| 193 | 2.41E-02 | 1.65E+00 | 2.74E-03 |  | 1.73 |  |
| 195 | 1.38E-02 | 1.66E+00 | 3.29E-03 |  | 2.03 |  |
| 167 | 1.97E-02 | 1.65E+00 | 2.93E-03 | 1.765 | 1.86 | -0.10 |
| 171 | 1.59E-02 | 1.66E+00 | 3.34E-03 | 1.879 | 1.95 | -0.07 |
| 173 | 1.75E-02 | 1.68E+00 | 1.30E-03 | 2.036 | 2.10 | -0.06 |
| 174 | 9.89E-03 | 1.69E+00 | 3.64E-03 |  | 2.11 |  |
| 175 | 1.57E-02 | 1.68E+00 | 1.03E-03 |  | 2.19 |  |
| 165 | 1.61E-02 | 1.65E+00 | 2.34E-03 | 2 | 2.06 | -0.06 |
| 179 | 1.29E-02 | 1.68E+00 | 1.52E-02 |  | 0.75 |  |
| 180 | 6.03E-03 | 1.92E+00 | 3.51E-03 | 2.119 | 2.08 | 0.04 |
| 187 | 1.69E-02 | 1.68E+00 | 6.94E-04 | 2.174 | 2.18 | -0.01 |
| 185 | 1.88E-02 | 1.73E+00 | 2.44E-03 |  | 1.89 |  |
| 183 | 2.16E-02 | 1.92E+00 | 2.56E-03 | 1.051 | 1.62 | -0.57 |
| 161 | 1.29E-02 | 1.65E+00 | 3.50E-03 | 1.854 | 2.05 | -0.19 |
| 190 | 1.44E-02 | 1.65E+00 | 1.40E-03 |  | 2.22 |  |
| 191 | 1.22E-02 | 1.68E+00 | 1.31E-03 | 2.027 | 2.29 | -0.26 |
| 184 | 1.13E-02 | 1.75E+00 | 3.20E-03 | 1.886 | 2.06 | -0.17 |
| 199 | 1.87E-02 | 1.44E+00 | 1.72E-03 | 2.237 | 2.20 | 0.04 |
| 194 | 1.76E-02 | 1.68E+00 | 9.53E-04 | 2.168 | 2.13 | 0.04 |
| 197 | 1.25E-02 | 1.66E+00 | 3.28E-03 | 2.187 | 2.08 | 0.11 |
| 181 | 3.72E-03 | 1.92E+00 | 3.53E-03 | 2.181 | 2.16 | 0.02 |
| 177 | 7.72E-03 | 1.69E+00 | 1.99E-04 | 2.585 | 2.56 | 0.02 |
| 182 | 1.43E-02 | 1.92E+00 | 2.58E-03 | 2.081 | 1.88 | 0.20 |
| 163 | 1.20E-02 | 1.92E+00 | 3.52E-03 | 2.168 | 1.86 | 0.31 |
| 192 | 1.84E-02 | 1.65E+00 | 1.50E-03 | 2.174 | 2.07 | 0.11 |
| 166 | 1.51E-02 | 1.92E+00 | 2.35E-03 | 2.027 | 1.88 | 0.15 |
| 164 | 1.61E-02 | 1.65E+00 | 2.34E-03 | 1.928 | 2.06 | -0.13 |
| 169 | 2.34E-02 | 1.65E+00 | 2.17E-03 | 2.076 | 1.81 | 0.26 |
| 178 | 8.35E-03 | 1.69E+00 | 3.71E-03 | 2.022 | 2.16 | -0.14 |
| 168 | 1.75E-02 | 1.65E+00 | 2.65E-03 | 2.444 | 1.97 | 0.47 |
| 162* | 9.39E-03 | 1.92E+00 | 3.42E-03 | 2.076 | 1.97 | 0.11 |
| 198* | 1.16E-02 | 1.66E+00 | 3.28E-03 | 2.215 | 2.11 | 0.10 |
| 196* | 1.24E-02 | 1.66E+00 | 3.24E-03 | 2.222 | 2.09 | 0.14 |
| 172* | 1.26E-02 | 1.68E+00 | 8.13E-04 | 2.31 | 2.33 | -0.02 |
| 176* | 6.59E-03 | 1.68E+00 | 7.46E-04 | 2.268 | 2.55 | -0.28 |
| 186* | 2.20E-02 | 1.44E+00 | 9.02E-04 | 2.208 | 2.17 | 0.04 |

**Table S70: Descriptor, experimental and predicted pIC_50_ values and their residuals for cell line based QSAR model against SF-549**

| **No.** | **GIAP** | **TPCCMD** | **MaBOO** | **Exp.** | **Pred.** | **Res.** |
| --- | --- | --- | --- | --- | --- | --- |
| 296 | 5.82E+03 | 1.03E+01 | 1.77E+00 | 5 | 5.12 | -0.12 |
| 300 | 4.43E+03 | 4.30E+00 | 1.63E+00 |  | 2.02 |  |
| 301 | 6.44E+03 | 6.51E+00 | 1.63E+00 |  | 2.77 |  |
| 303 | 7.31E+03 | 6.63E+00 | 1.63E+00 | 2.36 | 3.01 | -0.65 |
| 306 | 6.76E+03 | 5.54E+00 | 1.62E+00 | 2.37 | 2.60 | -0.23 |
| 308 | 8.30E+03 | 7.99E+00 | 1.63E+00 |  | 3.41 |  |
| 309 | 8.65E+03 | 6.82E+00 | 1.62E+00 |  | 3.23 |  |
| 310 | 6.82E+03 | 6.45E+00 | 1.62E+00 |  | 2.72 |  |
| 311 | 7.29E+03 | 6.94E+00 | 1.62E+00 | 3.31 | 2.89 | 0.42 |
| 314 | 7.13E+03 | 5.37E+00 | 1.62E+00 |  | 2.68 |  |
| 318 | 6.49E+03 | 3.59E+00 | 1.62E+00 |  | 2.32 |  |
| 319 | 7.35E+03 | 4.57E+00 | 1.62E+00 | 3.22 | 2.65 | 0.57 |
| 320 | 8.09E+03 | 8.28E+00 | 1.60E+00 | 2.67 | 2.94 | -0.27 |
| 321 | 6.41E+03 | 7.38E+00 | 1.63E+00 |  | 2.86 |  |
| 322 | 7.68E+03 | 3.78E+00 | 1.62E+00 |  | 2.65 |  |
| 323 | 7.27E+03 | 9.12E+00 | 1.63E+00 |  | 3.27 |  |
| 324 | 7.87E+03 | 5.57E+00 | 1.62E+00 |  | 2.89 |  |
| 325 | 8.05E+03 | 7.14E+00 | 1.62E+00 |  | 3.11 |  |
| 327 | 7.07E+03 | 5.16E+00 | 1.62E+00 |  | 2.64 |  |
| 328 | 7.50E+03 | 4.59E+00 | 1.62E+00 | 2.96 | 2.69 | 0.27 |
| 330 | 5.57E+03 | 1.55E+00 | 1.63E+00 |  | 2.02 |  |
| 331 | 7.35E+03 | 6.25E+00 | 1.63E+00 |  | 2.98 |  |
| 332 | 6.88E+03 | 2.75E+00 | 1.63E+00 | 2.8 | 2.49 | 0.32 |
| 335 | 6.60E+03 | 7.04E+00 | 1.63E+00 | 3.41 | 2.87 | 0.54 |
| 337 | 6.85E+03 | 2.00E+00 | 1.63E+00 | 1.82 | 2.40 | -0.58 |
| 338 | 6.95E+03 | 4.49E+00 | 1.63E+00 |  | 2.69 |  |
| 339 | 6.55E+03 | 4.36E+00 | 1.63E+00 |  | 2.57 |  |
| 341 | 7.09E+03 | 4.36E+00 | 1.63E+00 | 2.71 | 2.71 | 0.00 |
| 316 | 7.34E+03 | 3.56E+00 | 1.62E+00 | 1.93 | 2.54 | -0.61 |
| 298 | 8.37E+03 | 4.12E+00 | 1.73E+00 | 4.4 | 4.52 | -0.12 |
| 305 | 7.65E+03 | 7.25E+00 | 1.62E+00 | 3.09 | 3.02 | 0.07 |
| 334 | 6.32E+03 | 6.33E+00 | 1.62E+00 | 2.36 | 2.57 | -0.21 |
| 315 | 6.64E+03 | 7.53E+00 | 1.62E+00 | 2.73 | 2.79 | -0.06 |
| 299 | 8.11E+03 | 3.02E+00 | 1.72E+00 | 4.43 | 4.18 | 0.25 |
| 340 | 7.09E+03 | 3.54E+00 | 1.63E+00 | 2.82 | 2.62 | 0.20 |
| 317 | 6.63E+03 | 5.08E+00 | 1.63E+00 | 2.87 | 2.67 | 0.20 |
| 326 | 6.98E+03 | 2.51E+00 | 1.63E+00 | 2.19 | 2.49 | -0.30 |
| 333 | 6.90E+03 | 3.38E+00 | 1.63E+00 | 2.9 | 2.56 | 0.34 |
| 313 | 7.95E+03 | 6.05E+00 | 1.62E+00 | 2.94 | 2.97 | -0.03 |
| 297* | 7.77E+03 | 9.57E+00 | 1.73E+00 | 4.4 | 4.95 | -0.55 |
| 302* | 6.76E+03 | 7.18E+00 | 1.62E+00 | 2.57 | 2.78 | -0.21 |
| 307* | 7.71E+03 | 6.80E+00 | 1.62E+00 | 3.31 | 2.98 | 0.33 |
| 312* | 7.71E+03 | 4.05E+00 | 1.62E+00 | 2.71 | 2.69 | 0.02 |
| 329* | 6.40E+03 | 1.47E+00 | 1.62E+00 | 2.03 | 2.07 | -0.04 |
| 336* | 7.39E+03 | 4.63E+00 | 1.63E+00 | 2.73 | 2.82 | -0.09 |

**Table S71:** Descriptor, experimental and predicted pIC_50_ values and their residuals for test set 2 compounds in cell line based QSAR model against SN12C

| **No.** | **NN** | **XYS/XYR** | **MiVC** | **Exp.** | **Pred.** | **Res.** |
| --- | --- | --- | --- | --- | --- | --- |
| 298 | 2.00E+00 | 5.88E-01 | 3.66E+00 | 3.09 | 3.14 | -0.05 |
| 314 | 3.00E+00 | 5.42E-01 | 3.78E+00 |  | 2.87 |  |
| 311 | 3.00E+00 | 5.04E-01 | 3.78E+00 | 3.03 | 3.12 | -0.09 |
| 297 | 2.00E+00 | 6.84E-01 | 3.67E+00 |  | 2.47 |  |
| 333 | 3.00E+00 | 5.85E-01 | 3.82E+00 |  | 2.46 |  |
| 320 | 5.00E+00 | 5.77E-01 | 3.67E+00 | 2.23 | 2.57 | -0.34 |
| 326 | 4.00E+00 | 6.30E-01 | 3.82E+00 | 1.8 | 1.95 | -0.15 |
| 328 | 5.00E+00 | 5.12E-01 | 3.83E+00 | 2.74 | 2.50 | 0.24 |
| 318 | 3.00E+00 | 6.41E-01 | 3.67E+00 |  | 2.55 |  |
| 327 | 3.00E+00 | 6.17E-01 | 3.81E+00 |  | 2.28 |  |
| 334 | 3.00E+00 | 6.03E-01 | 3.82E+00 | 1.99 | 2.34 | -0.35 |
| 335 | 4.00E+00 | 5.81E-01 | 3.81E+00 | 2.81 | 2.31 | 0.50 |
| 337 | 4.00E+00 | 6.09E-01 | 3.82E+00 | 1.72 | 2.09 | -0.37 |
| 340 | 4.00E+00 | 6.38E-01 | 3.82E+00 | 1.86 | 1.90 | -0.04 |
| 332 | 4.00E+00 | 5.88E-01 | 3.82E+00 | 2.75 | 2.23 | 0.52 |
| 319 | 3.00E+00 | 5.84E-01 | 3.67E+00 | 3.1 | 2.93 | 0.17 |
| 302 | 3.00E+00 | 6.06E-01 | 3.78E+00 | 2.6 | 2.44 | 0.16 |
| 305 | 3.00E+00 | 5.81E-01 | 3.78E+00 | 2.59 | 2.61 | -0.02 |
| 307 | 3.00E+00 | 5.96E-01 | 3.78E+00 | 3.03 | 2.51 | 0.52 |
| 313 | 3.00E+00 | 5.30E-01 | 3.78E+00 | 2.95 | 2.95 | 0.01 |
| 316 | 4.00E+00 | 6.13E-01 | 3.67E+00 | 2.63 | 2.53 | 0.10 |
| 341 | 3.00E+00 | 6.20E-01 | 3.82E+00 | 2.43 | 2.22 | 0.21 |
| 329 | 3.00E+00 | 5.82E-01 | 3.81E+00 | 1.69 | 2.51 | -0.82 |
| 342 | 5.00E+00 | 5.29E-01 | 3.82E+00 | 2.22 | 2.42 | -0.20 |
| 306* | 3.00E+00 | 6.21E-01 | 3.78E+00 | 2.24 | 2.34 | -0.10 |
| 312* | 4.00E+00 | 6.25E-01 | 3.78E+00 | 1.9 | 2.11 | -0.21 |
| 315* | 3.00E+00 | 5.69E-01 | 3.78E+00 | 2.73 | 2.69 | 0.04 |
| 336* | 5.00E+00 | 4.95E-01 | 3.83E+00 | 2.68 | 2.62 | 0.07 |
| 317* | 3.00E+00 | 5.76E-01 | 3.67E+00 | 2.72 | 2.98 | -0.26 |
| 296* | 2.00E+00 | 6.71E-01 | 3.77E+00 | 2.7 | 2.24 | 0.46 |
| 303# | 4.00E+00 | 6.19E-01 | 3.78E+00 | 1.09 | 2.15 | -1.06 |

**Table S72:** Descriptor, experimental and predicted pIC_50_ values and their residuals for test set 2 compounds in cell line based QSAR model against U937

| **No.** | **MiBOH** | **MiVN** | **H-HD-2/T** | **Exp.** | **Pred.** | **Res.** |
| --- | --- | --- | --- | --- | --- | --- |
| 76 | 7.37E-01 | 3.12E+00 | 2.82E-02 | 1.815 | 1.70 | 0.11 |
| 78 | 7.68E-01 | 2.95E+00 | 2.94E-02 |  | -0.61 |  |
| 79 | 7.12E-01 | 3.12E+00 | 2.55E-02 |  | 2.42 |  |
| 80 | 7.69E-01 | 3.13E+00 | 2.06E-02 |  | 1.46 |  |
| 82 | 7.66E-01 | 3.12E+00 | 2.79E-02 |  | 1.05 |  |
| 83 | 7.73E-01 | 3.12E+00 | 2.16E-02 |  | 1.22 |  |
| 84 | 7.27E-01 | 3.06E+00 | 3.26E-02 |  | 1.16 |  |
| 88 | 7.14E-01 | 3.07E+00 | 2.67E-02 | 1.854 | 1.86 | 0.00 |
| 90 | 7.14E-01 | 3.12E+00 | 2.19E-02 | 2.699 | 2.56 | 0.14 |
| 91 | 7.14E-01 | 3.12E+00 | 1.93E-02 | 3 | 2.70 | 0.30 |
| 92 | 7.14E-01 | 3.12E+00 | 2.04E-02 | 2.569 | 2.64 | -0.07 |
| 93 | 7.13E-01 | 3.12E+00 | 2.00E-02 | 2.337 | 2.69 | -0.35 |
| 95 | 7.29E-01 | 3.12E+00 | 1.80E-02 | 2.553 | 2.43 | 0.13 |
| 96 | 7.29E-01 | 3.11E+00 | 1.81E-02 | 2.187 | 2.33 | -0.14 |
| 97 | 7.30E-01 | 3.12E+00 | 1.75E-02 | 2.569 | 2.43 | 0.14 |
| 98 | 7.28E-01 | 2.97E+00 | 1.57E-02 | 1.229 | 1.22 | 0.01 |
| 99 | 7.35E-01 | 3.12E+00 | 2.37E-02 | 1.658 | 1.99 | -0.33 |
| 100 | 7.36E-01 | 3.12E+00 | 2.00E-02 |  | 2.16 |  |
| 101 | 7.39E-01 | 3.12E+00 | 2.22E-02 |  | 1.97 |  |
| 81 | 7.74E-01 | 3.12E+00 | 2.20E-02 | 1.244 | 1.18 | 0.06 |
| 86 | 7.63E-01 | 3.12E+00 | 1.86E-02 | 1.495 | 1.61 | -0.12 |
| 85 | 7.14E-01 | 3.12E+00 | 1.96E-02 | 2.745 | 2.69 | 0.06 |
| 77 | 7.67E-01 | 3.12E+00 | 2.57E-02 | 1.215 | 1.15 | 0.07 |
| 75* | 7.14E-01 | 3.12E+00 | 2.14E-02 | 2.319 | 2.59 | -0.27 |
| 87* | 7.14E-01 | 3.07E+00 | 2.69E-02 | 2.022 | 1.85 | 0.17 |
| 89* | 7.14E-01 | 3.06E+00 | 2.65E-02 | 1.678 | 1.78 | -0.10 |
| 94* | 7.37E-01 | 3.12E+00 | 2.47E-02 | 1.921 | 1.89 | 0.03 |

**Table S73:** Descriptor, experimental and predicted pIC_50_ values and their residuals for test set 2 compounds in cell line based QSAR model against UACC-62

| No. | MaPCN | MaPC | MaVO | Exp. | Pred. | Res. |
| --- | --- | --- | --- | --- | --- | --- |
| 297 | -9.27E-02 | 8.28E-02 | 2.11E+00 | 4.52 | 4.78 | -0.26 |
| 298 | -9.26E-02 | 6.38E-02 | 2.12E+00 | 4.52 | 4.62 | -0.10 |
| 305 | -8.64E-02 | 6.14E-02 | 2.10E+00 | 3 | 2.91 | 0.10 |
| 306 | -8.65E-02 | 6.14E-02 | 2.10E+00 | 2.94 | 2.93 | 0.01 |
| 307 | -8.64E-02 | 8.34E-02 | 2.10E+00 | 3.34 | 3.13 | 0.22 |
| 308 | -8.64E-02 | 6.14E-02 | 2.11E+00 |  | 2.96 |  |
| 309 | -8.64E-02 | 6.14E-02 | 2.10E+00 |  | 2.91 |  |
| 310 | -8.67E-02 | 6.14E-02 | 2.10E+00 |  | 2.98 |  |
| 314 | -8.67E-02 | 6.14E-02 | 2.10E+00 |  | 2.98 |  |
| 318 | -8.63E-02 | 6.14E-02 | 2.10E+00 |  | 2.88 |  |
| 319 | -8.62E-02 | 8.34E-02 | 2.10E+00 | 3.46 | 3.07 | 0.39 |
| 320 | -8.63E-02 | 6.14E-02 | 2.10E+00 | 2.47 | 2.88 | -0.41 |
| 327 | -8.61E-02 | 8.34E-02 | 1.97E+00 | 1.79 | 2.28 | -0.49 |
| 328 | -8.61E-02 | 6.14E-02 | 1.97E+00 |  | 2.07 |  |
| 329 | -8.61E-02 | 8.28E-02 | 1.96E+00 | 1.85 | 2.22 | -0.37 |
| 332 | -8.79E-02 | 6.14E-02 | 1.97E+00 | 2.85 | 2.52 | 0.33 |
| 334 | -8.79E-02 | 8.28E-02 | 1.96E+00 | 2.69 | 2.68 | 0.01 |
| 335 | -8.79E-02 | 6.14E-02 | 1.97E+00 | 3.51 | 2.52 | 0.99 |
| 336 | -8.71E-02 | 6.14E-02 | 1.97E+00 | 2.72 | 2.32 | 0.40 |
| 337 | -8.71E-02 | 6.14E-02 | 1.97E+00 | 1.77 | 2.32 | -0.55 |
| 340 | -8.71E-02 | 6.14E-02 | 1.97E+00 | 1.81 | 2.32 | -0.51 |
| 342 | -8.71E-02 | 6.14E-02 | 1.97E+00 | 2.14 | 2.32 | -0.18 |
| 311 | -8.67E-02 | 8.28E-02 | 2.10E+00 | 3.34 | 3.20 | 0.15 |
| 312 | -8.67E-02 | 6.14E-02 | 2.11E+00 | 3.03 | 3.04 | -0.01 |
| 333 | -8.79E-02 | 8.34E-02 | 1.97E+00 | 3.13 | 2.74 | 0.39 |
| 341 | -8.71E-02 | 8.34E-02 | 1.97E+00 | 2.51 | 2.54 | -0.03 |
| 316 | -8.62E-02 | 6.14E-02 | 2.10E+00 | 2.79 | 2.85 | -0.06 |
| 296* | -8.93E-02 | 9.23E-02 | 2.18E+00 | 5 | 4.42 | 0.58 |
| 302* | -8.65E-02 | 6.14E-02 | 2.10E+00 | 2.78 | 2.93 | -0.15 |
| 313* | -8.67E-02 | 6.14E-02 | 2.10E+00 | 2.94 | 2.98 | -0.04 |
| 315* | -8.67E-02 | 6.14E-02 | 2.10E+00 | 2.79 | 2.98 | -0.19 |
| 317* | -8.62E-02 | 8.28E-02 | 2.10E+00 | 3.33 | 3.07 | 0.26 |
| 303# | -8.64E-02 | 6.14E-02 | 2.11E+00 | 1.67 | 2.96 | -1.29 |

**Table S74:** Descriptor, experimental and predicted pIC_50_ values and their residuals for test set 2 compounds in cell line based QSAR model against WM164

| No. | PS-3AZ | KHI3 | MiNRN | Exp. | Pred. | Res. |
| --- | --- | --- | --- | --- | --- | --- |
| 192 | 1.52E+01 | 7.80E+00 | 1.90E-04 | 2.3565 | 2.46 | -0.10 |
| 193 | 1.53E+01 | 7.73E+00 | 1.03E-04 | 2.9586 | 2.65 | 0.31 |
| 161 | 2.07E+01 | 8.69E+00 | 8.49E-05 | 2.5229 | 2.65 | -0.13 |
| 198 | 1.17E+01 | 6.30E+00 | 8.01E-04 |  | 2.56 |  |
| 197 | 1.06E+01 | 5.80E+00 | 7.99E-04 |  | 2.87 |  |
| 196 | 1.06E+01 | 5.80E+00 | 8.01E-04 |  | 2.86 |  |
| 195 | 9.80E+00 | 5.30E+00 | 7.96E-04 |  | 3.22 |  |
| 199 | 1.14E+01 | 6.70E+00 | 2.47E-03 |  | 0.04 |  |
| 166 | 1.87E+01 | 8.41E+00 | 1.20E-04 | 2.9208 | 2.54 | 0.38 |
| 172 | 1.45E+01 | 7.73E+00 | 3.37E-04 | 2.4089 | 2.22 | 0.19 |
| 173 | 1.50E+01 | 7.71E+00 | 2.46E-04 | 2.9586 | 2.44 | 0.52 |
| 175 | 1.15E+01 | 8.33E+00 | 5.33E-04 | 0.8945 | 0.88 | 0.01 |
| 176 | 1.08E+01 | 8.44E+00 | 1.25E-04 | 1.1911 | 1.17 | 0.03 |
| 177 | 1.09E+01 | 6.97E+00 | 6.56E-05 | 2.7696 | 2.69 | 0.08 |
| 178 | 1.07E+01 | 6.87E+00 | 5.20E-05 | 2.8539 | 2.77 | 0.08 |
| 179 | 1.09E+01 | 6.16E+00 | 8.48E-04 | 2.3872 | 2.51 | -0.12 |
| 180 | 1.08E+01 | 6.67E+00 | 9.26E-04 |  | 1.89 |  |
| 181 | 1.53E+01 | 7.39E+00 | 7.03E-05 | 2.699 | 3.02 | -0.32 |
| 182 | 1.68E+01 | 8.14E+00 | 7.09E-05 | 2.8861 | 2.55 | 0.34 |
| 183 | 1.46E+01 | 7.46E+00 | 2.26E-04 | 2.6198 | 2.64 | -0.02 |
| 185 | 1.51E+01 | 8.26E+00 | 4.81E-05 | 1.6904 | 2.17 | -0.48 |
| 187 | 1.46E+01 | 7.39E+00 | 4.87E-04 | 2.3098 | 2.38 | -0.07 |
| 188 | 1.56E+01 | 7.82E+00 | 1.25E-04 | 2.4949 | 2.59 | -0.09 |
| 189 | 1.61E+01 | 8.32E+00 | 1.42E-04 | 1.7305 | 2.16 | -0.43 |
| 191 | 1.72E+01 | 7.68E+00 | 1.04E-04 | 2.7696 | 3.02 | -0.25 |
| 194 | 1.01E+01 | 1.18E+00 | 8.28E-05 |  | 8.19 |  |
| 163 | 2.04E+01 | 8.34E+00 | 8.64E-05 | 2.9586 | 2.94 | 0.02 |
| 184 | 1.49E+01 | 7.46E+00 | 2.26E-04 | 2.6198 | 2.69 | -0.07 |
| 165 | 1.87E+01 | 8.41E+00 | 1.20E-04 | 2.5686 | 2.54 | 0.03 |
| 167 | 1.86E+01 | 8.06E+00 | 1.19E-04 | 2.9586 | 2.87 | 0.09 |
| 162 | 2.05E+01 | 8.34E+00 | 9.07E-05 | 2.8861 | 2.95 | -0.07 |
| 170 | 1.63E+01 | 7.61E+00 | 1.26E-04 | 3 | 2.91 | 0.09 |
| 164* | 1.98E+01 | 8.19E+00 | 8.63E-05 | 3.1549 | 2.99 | 0.17 |
| 169* | 1.63E+01 | 7.59E+00 | 1.84E-04 | 2.9208 | 2.86 | 0.07 |
| 168* | 1.55E+01 | 7.60E+00 | 1.22E-04 | 2.6198 | 2.79 | -0.17 |
| 174* | 1.62E+01 | 8.60E+00 | 1.43E-04 | 1.9706 | 1.90 | 0.07 |
| 190* | 1.60E+01 | 8.06E+00 | 1.38E-04 | 2.2441 | 2.40 | -0.16 |
| 186# | 1.57E+01 | 7.61E+00 | 2.85E-03 | 1.7328 | -0.59 | 2.33 |

**Table S75:** Descriptor, experimental and predicted pIC_50_ values and their residuals for test set 2 compounds in cell line based QSAR model against U373-MG

| **No.** | **ANRN** | **H-1E** | **MaBON** | **Exp.** | **Pred.** | **Res.** |
| --- | --- | --- | --- | --- | --- | --- |
| 4 | 2.81E-02 | -6.18E+00 | 1.13E+00 | 2.0458 | 2.31 | -0.27 |
| 6 | 2.81E-02 | -6.34E+00 | 1.15E+00 |  | 2.38 |  |
| 7 | 2.76E-02 | -6.54E+00 | 1.14E+00 |  | 2.47 |  |
| 8 | 2.81E-02 | -6.17E+00 | 1.14E+00 | 2.3596 | 2.31 | 0.05 |
| 9 | 2.76E-02 | -6.56E+00 | 1.13E+00 |  | 2.48 |  |
| 14 | 2.80E-02 | -6.27E+00 | 1.11E+00 | 2.3768 | 2.35 | 0.02 |
| 16 | 2.06E-02 | -6.76E+00 | 1.12E+00 | 3.0458 | 2.75 | 0.30 |
| 17 | 2.08E-02 | -6.46E+00 | 1.12E+00 | 2.4685 | 2.62 | -0.15 |
| 18 | 2.08E-02 | -6.46E+00 | 1.13E+00 | 2.3872 | 2.62 | -0.23 |
| 20 | 2.23E-02 | -5.77E+00 | 1.59E+00 | 2.0458 | 2.25 | -0.20 |
| 21 | 2.07E-02 | -6.52E+00 | 1.10E+00 | 3 | 2.65 | 0.35 |
| 25 | 1.41E-02 | -5.75E+00 | 1.09E+00 | 2.4437 | 2.52 | -0.07 |
| 26 | 2.83E-02 | -5.77E+00 | 1.74E+00 |  | 2.07 |  |
| 27 | 2.84E-02 | -5.83E+00 | 1.74E+00 |  | 2.09 |  |
| 28 | 2.84E-02 | -5.67E+00 | 1.82E+00 | 2.1079 | 2.02 | 0.09 |
| 29 | 2.83E-02 | -6.00E+00 | 1.75E+00 | 2.1938 | 2.16 | 0.03 |
| 30 | 2.10E-02 | -6.38E+00 | 2.86E+00 | 2.3768 | 2.37 | 0.01 |
| 32 | 2.77E-02 | -5.53E+00 | 1.11E+00 |  | 2.06 |  |
| 33 | 2.85E-02 | -5.73E+00 | 1.09E+00 |  | 2.13 |  |
| 34 | 2.11E-02 | -5.61E+00 | 1.51E+00 |  | 2.22 |  |
| 35 | 2.84E-02 | -5.62E+00 | 1.12E+00 | 2.1487 | 2.08 | 0.07 |
| 37 | 2.07E-02 | -5.68E+00 | 1.20E+00 |  | 2.30 |  |
| 38 | 2.08E-02 | -5.90E+00 | 1.15E+00 |  | 2.39 |  |
| 24 | 2.06E-02 | -6.17E+00 | 1.09E+00 | 2.5086 | 2.51 | 0.00 |
| 11 | 2.78E-02 | -6.47E+00 | 1.16E+00 | 2.0506 | 2.43 | -0.38 |
| 12 | 2.77E-02 | -6.59E+00 | 1.17E+00 | 2.0915 | 2.48 | -0.39 |
| 2 | 2.83E-02 | -5.81E+00 | 1.14E+00 | 2.4559 | 2.16 | 0.30 |
| 10 | 2.83E-02 | -6.04E+00 | 1.13E+00 | 2.5686 | 2.25 | 0.32 |
| 31 | 2.11E-02 | -6.11E+00 | 1.74E+00 | 2.301 | 2.40 | -0.10 |
| 22 | 2.06E-02 | -6.30E+00 | 1.09E+00 | 2.6778 | 2.57 | 0.11 |
| 13 | 2.79E-02 | -6.26E+00 | 1.12E+00 | 2.2218 | 2.35 | -0.13 |
| 1 | 2.75E-02 | -7.10E+00 | 1.52E+00 | 2.8861 | 2.65 | 0.24 |
| 36 | 2.08E-02 | -5.94E+00 | 1.14E+00 | 2.4437 | 2.41 | 0.04 |
| 3* | 4.23E-02 | -6.52E+00 | 1.07E+00 | 2.1249 | 2.08 | 0.05 |
| 5* | 2.80E-02 | -6.29E+00 | 1.15E+00 | 2.3565 | 2.36 | 0.00 |
| 15* | 2.05E-02 | -6.82E+00 | 1.11E+00 | 2.4815 | 2.77 | -0.29 |
| 19* | 2.07E-02 | -6.00E+00 | 1.09E+00 | 2.4437 | 2.44 | 0.00 |
| 23* | 3.94E-03 | -5.64E+00 | 1.10E+00 | 2.8539 | 2.74 | 0.11 |
| 39* | 1.66E-02 | -6.13E+00 | 2.86E+00 | 2.0269 | 2.39 | -0.36 |

**Table S76:** Descriptor, experimental and predicted pIC_50_ values and their residuals for test set 2 compounds in cell line based QSAR model against K562

| **No.** | **H-HC-1Q** | **RPCSZ** | **MiERC** | **Exp.** | **Pred.** | **Res.** |
| --- | --- | --- | --- | --- | --- | --- |
| 42 | 2.88E+01 | 4.26E+00 | 1.95E-04 |  | 3.02 |  |
| 43 | 2.83E+01 | 3.87E+00 | 7.22E-05 |  | 2.06 |  |
| 44 | 1.83E+01 | 0.00E+00 | 2.48E-05 |  | 2.31 |  |
| 45 | 1.96E+01 | 1.66E-01 | 2.63E-06 | 2.6021 | 2.12 | 0.48 |
| 46 | 1.41E+01 | 0.00E+00 | 5.28E-06 |  | 2.02 |  |
| 50 | 2.59E+01 | 8.40E-02 | 6.02E-06 | 2.0862 | 2.37 | -0.28 |
| 52 | 2.63E+01 | 8.39E-02 | 2.72E-06 | 2.9208 | 2.35 | 0.57 |
| 53 | 2.58E+01 | 1.69E-01 | 3.61E-07 |  | 2.30 |  |
| 54 | 2.44E+01 | 1.62E-01 | 3.32E-07 | 2.2291 | 2.25 | -0.03 |
| 55 | 2.81E+01 | 2.36E-01 | 5.46E-06 |  | 2.39 |  |
| 61 | 1.89E+01 | 1.98E+00 | 8.96E-07 |  | 1.63 |  |
| 62 | 1.34E+01 | 2.01E+00 | 1.47E-05 |  | 1.57 |  |
| 67 | 7.00E+00 | 0.00E+00 | 1.51E-07 | 1.6946 | 1.75 | -0.06 |
| 68 | 2.08E+01 | 3.65E+00 | 3.07E-06 |  | 1.29 |  |
| 69 | 3.09E+01 | 2.62E+00 | 4.79E-06 | 1.8297 | 1.88 | -0.05 |
| 70 | 4.40E+01 | 2.65E+00 | 4.84E-06 |  | 2.28 |  |
| 71 | 1.82E+01 | 0.00E+00 | 7.54E-06 | 2.1367 | 2.16 | -0.03 |
| 72 | 2.62E+01 | 1.50E+00 | 5.64E-06 | 1.9245 | 2.02 | -0.10 |
| 74 | 1.87E+01 | 2.35E+00 | 2.92E-06 | 1.6402 | 1.55 | 0.09 |
| 40 | 1.83E+01 | 8.70E-02 | 3.71E-06 | 1.9318 | 2.11 | -0.18 |
| 60 | 1.73E+01 | 7.56E-02 | 5.84E-06 | 2.3768 | 2.10 | 0.28 |
| 64 | 1.27E+01 | 8.01E-02 | 1.47E-05 | 2.0362 | 2.03 | 0.00 |
| 47 | 1.91E+01 | 0.00E+00 | 5.82E-07 | 2.1249 | 2.13 | -0.01 |
| 48 | 2.02E+01 | 1.80E-01 | 5.96E-07 | 1.8761 | 2.12 | -0.25 |
| 57 | 2.43E+01 | 1.93E-01 | 1.10E-06 | 1.9957 | 2.25 | -0.25 |
| 58 | 1.74E+01 | 1.85E-01 | 1.83E-06 | 1.8386 | 2.04 | -0.21 |
| 65 | 9.40E+00 | 0.00E+00 | 7.85E-06 | 1.8097 | 1.89 | -0.08 |
| 73 | 9.33E+00 | 8.53E-01 | 1.07E-06 | 1.7167 | 1.62 | 0.10 |
| 41* | 1.99E+01 | 0.00E+00 | 5.07E-06 | 2.0223 | 2.20 | -0.17 |
| 51* | 2.62E+01 | 1.61E-01 | 1.97E-06 | 2.4089 | 2.32 | 0.09 |
| 59# | 1.86E+01 | 0.00E+00 | 3.31E-05 | 1.3768 | 2.39 | -1.02 |
| 66* | 1.32E+01 | 1.91E-01 | 6.00E-06 | 1.6253 | 1.95 | -0.32 |
| 56* | 2.38E+01 | 7.88E-02 | 1.33E-05 | 1.9957 | 2.37 | -0.37 |

**Table S77:** Descriptor, experimental and predicted pIC_50_ values and their residuals for test set 2 compounds in cell line based QSAR model against S5

| **No.** | **ACI2** | **MiPCO** | **RNAB** | **Exp.** | **Pred.** | **Res.** |
| --- | --- | --- | --- | --- | --- | --- |
| 126 | 7.69E-01 | -1.67E-01 | 0.00E+00 | 1.1871 | 1.21 | -0.02 |
| 128 | 1.06E+00 | -1.67E-01 | 0.00E+00 | 1.1871 | 1.26 | -0.07 |
| 132 | 1.26E+00 | -1.64E-01 | 1.94E-01 | 1.5086 | 1.41 | 0.10 |
| 136 | 1.23E+00 | -1.64E-01 | 1.67E-01 | 1.6021 | 1.39 | 0.21 |
| 137 | 1.19E+00 | -1.82E-01 | 1.73E-01 | 1.1427 | 1.21 | -0.07 |
| 142 | 7.09E-01 | -1.74E-01 | 1.03E-01 | 1.1367 | 1.18 | -0.04 |
| 143 | 8.70E-01 | -1.75E-01 | 1.03E-01 | 1.1079 | 1.19 | -0.09 |
| 145 | 7.88E-01 | -1.73E-01 | 0.00E+00 | 1.1675 | 1.15 | 0.02 |
| 149 | 1.16E+00 | -1.81E-01 | 0.00E+00 | 1.1549 | 1.14 | 0.02 |
| 150 | 8.41E-01 | -1.77E-01 | 0.00E+00 | 1.1427 | 1.12 | 0.02 |
| 146 | 7.97E-01 | -1.82E-01 | 0.00E+00 | 1.1675 | 1.07 | 0.10 |
| 153 | 7.57E-01 | -1.75E-01 | 0.00E+00 | 1.1308 | 1.13 | 0.00 |
| 154 | 7.57E-01 | -1.71E-01 | 2.44E-02 | 1.1192 | 1.18 | -0.06 |
| 155 | 7.20E-01 | -1.72E-01 | 0.00E+00 | 1.2147 | 1.15 | 0.06 |
| 156 | 8.38E-01 | -1.75E-01 | 0.00E+00 | 1.1938 | 1.14 | 0.05 |
| 157 | 6.71E-01 | -1.72E-01 | 2.50E-02 | 1.1739 | 1.15 | 0.02 |
| 124 | 8.88E-01 | -1.76E-01 | 0.00E+00 | 1.1549 | 1.14 | 0.02 |
| 135 | 1.02E+00 | -1.63E-01 | 1.73E-01 | 1.2241 | 1.37 | -0.14 |
| 123 | 6.90E-01 | -1.75E-01 | 0.00E+00 | 1.2218 | 1.12 | 0.11 |
| 151 | 1.32E+00 | -1.81E-01 | 0.00E+00 | 1.1427 | 1.17 | -0.02 |
| 127 | 9.67E-01 | -1.67E-01 | 0.00E+00 | 1.1549 | 1.24 | -0.09 |
| 130* | 9.61E-01 | -1.74E-01 | 1.92E-02 | 1.1675 | 1.18 | -0.01 |
| 147 | 9.68E-01 | -1.81E-01 | 0.00E+00 | 1.1192 | 1.11 | 0.01 |
| 148 | 1.06E+00 | -1.81E-01 | 0.00E+00 | 1.1308 | 1.12 | 0.01 |
| 141 | 7.75E-01 | -1.74E-01 | 1.11E-01 | 1.1549 | 1.19 | -0.04 |
| 158 | 1.09E+00 | -1.76E-01 | 0.00E+00 | 1.1192 | 1.17 | -0.06 |
| 159 | 1.07E+00 | -1.75E-01 | 0.00E+00 | 1.1308 | 1.18 | -0.05 |
| 125* | 7.20E-01 | -1.77E-01 | 0.00E+00 | 1.1871 | 1.10 | 0.09 |
| 131* | 1.17E+00 | -1.74E-01 | 1.72E-02 | 1.3279 | 1.22 | 0.11 |
| 139* | 7.66E-01 | -1.75E-01 | 5.97E-02 | 1.2366 | 1.16 | 0.08 |
| 140* | 9.00E-01 | -1.75E-01 | 1.11E-01 | 1.1675 | 1.20 | -0.04 |
| 152* | 9.58E-01 | -1.81E-01 | 0.00E+00 | 1.1192 | 1.10 | 0.02 |
| 160* | 7.64E-01 | -1.56E-01 | 0.00E+00 | 1.1308 | 1.31 | -0.18 |
| 144* | 7.75E-01 | -1.72E-01 | 1.30E-01 | 1.3565 | 1.22 | 0.14 |
| 138* | 1.41E+00 | -1.77E-01 | 1.57E-01 | 1.4949 | 1.29 | 0.20 |
| 129* | 8.62E-01 | -1.74E-01 | 2.04E-02 | 1.1367 | 1.16 | -0.03 |
| 134# | 1.06E+00 | -1.64E-01 | 1.94E-01 | 1.9208 | 1.37 | 0.55 |

**Table S78:** Descriptor, experimental and predicted pIC_50_ values and their residuals for test set 2 compounds in cell line based QSAR model against S6

| **No.** | **KHI3** | **MV/X** | **MaPCH** | **Exp.** | **Pred.** | **Res.** |
| --- | --- | --- | --- | --- | --- | --- |
| 192 | 7.80E+00 | 2.53E-01 | 5.37E-02 | 1.81 | 2.00 | -0.19 |
| 193 | 7.73E+00 | 2.35E-01 | 5.52E-02 | 2.66 | 2.01 | 0.65 |
| 162 | 8.34E+00 | 1.79E-01 | 5.37E-02 | 2.33 | 2.53 | -0.20 |
| 198 | 6.30E+00 | 2.40E-01 | 5.37E-02 |  | 2.48 |  |
| 197 | 5.80E+00 | 2.48E-01 | 5.37E-02 |  | 2.53 |  |
| 196 | 5.80E+00 | 2.46E-01 | 5.37E-02 |  | 2.55 |  |
| 195 | 5.30E+00 | 2.60E-01 | 5.37E-02 |  | 2.54 |  |
| 199 | 6.70E+00 | 2.46E-01 | 4.62E-02 |  | 3.13 |  |
| 170 | 7.61E+00 | 2.60E-01 | 5.37E-02 | 2.64 | 1.98 | 0.66 |
| 172 | 7.73E+00 | 2.42E-01 | 5.56E-02 | 2.1 | 1.91 | 0.19 |
| 173 | 7.71E+00 | 2.16E-01 | 5.52E-02 | 2.66 | 2.19 | 0.47 |
| 174 | 8.60E+00 | 2.59E-01 | 5.52E-02 | 1.72 | 1.59 | 0.13 |
| 176 | 8.44E+00 | 3.13E-01 | 5.52E-02 | 0.966 | 1.14 | -0.18 |
| 178 | 6.87E+00 | 2.07E-01 | 5.64E-02 | 2.25 | 2.35 | -0.10 |
| 179 | 6.16E+00 | 2.21E-01 | 5.63E-02 | 2.24 | 2.40 | -0.16 |
| 180 | 6.67E+00 | 2.50E-01 | 5.89E-02 | 1.26 | 1.74 | -0.48 |
| 183 | 7.46E+00 | 2.09E-01 | 5.37E-02 | 2.22 | 2.47 | -0.25 |
| 182 | 8.14E+00 | 1.79E-01 | 5.52E-02 | 2.8 | 2.41 | 0.39 |
| 184 | 7.46E+00 | 2.54E-01 | 5.37E-02 | 2 | 2.07 | -0.07 |
| 185 | 8.26E+00 | 2.08E-01 | 5.52E-02 | 1.82 | 2.13 | -0.31 |
| 186 | 7.61E+00 | 2.71E-01 | 5.52E-02 | 1.38 | 1.72 | -0.34 |
| 188 | 7.82E+00 | 2.43E-01 | 5.52E-02 | 1.92 | 1.92 | 0.00 |
| 189 | 8.32E+00 | 2.28E-01 | 6.39E-02 | 0.945 | 1.00 | -0.06 |
| 190 | 8.06E+00 | 2.12E-01 | 5.74E-02 | 1.03 | 1.90 | -0.87 |
| 191 | 7.68E+00 | 2.30E-01 | 5.84E-02 | 2.19 | 1.73 | 0.46 |
| 194 | 1.18E+00 | 3.28E-01 | 5.83E-02 |  | 2.45 |  |
| 165 | 8.41E+00 | 2.05E-01 | 5.37E-02 | 1.84 | 2.28 | -0.44 |
| 167 | 8.06E+00 | 2.04E-01 | 5.37E-02 | 2.49 | 2.37 | 0.12 |
| 168 | 7.60E+00 | 2.35E-01 | 5.37E-02 | 2.37 | 2.21 | 0.17 |
| 166 | 8.41E+00 | 2.05E-01 | 5.37E-02 | 2.48 | 2.28 | 0.20 |
| 177 | 6.97E+00 | 2.50E-01 | 5.64E-02 | 2.41 | 1.94 | 0.47 |
| 187 | 7.39E+00 | 2.77E-01 | 4.68E-02 | 2.33 | 2.62 | -0.29 |
| 161* | 8.69E+00 | 1.89E-01 | 5.37E-02 | 2.23 | 2.35 | -0.12 |
| 169* | 7.59E+00 | 2.15E-01 | 5.37E-02 | 2.52 | 2.39 | 0.13 |
| 163* | 8.34E+00 | 1.85E-01 | 5.37E-02 | 2.49 | 2.47 | 0.02 |
| 175* | 8.33E+00 | 2.88E-01 | 5.52E-02 | 1.02 | 1.39 | -0.37 |
| 181* | 7.39E+00 | 1.56E-01 | 5.52E-02 | 2.68 | 2.80 | -0.12 |

**Table S79:** Descriptor, experimental and predicted pIC_50_ values and their residuals for test set 2 compounds in cell line based QSAR model against S7

| **No.** | **RNC** | **PS-3AZ** | **HS-1/T** | **Exp.** | **Pred.** | **Res.** |
| --- | --- | --- | --- | --- | --- | --- |
| 200 | 4.74E-01 | 1.29E+01 | 1.82E-01 |  | 2.07 |  |
| 202 | 4.60E-01 | 1.37E+01 | 2.08E-01 | 2.190 | 1.72 | 0.47 |
| 205 | 5.00E-01 | 1.26E+01 | 1.26E-01 | 2.740 | 2.94 | -0.20 |
| 214 | 4.35E-01 | 1.71E+01 | 6.85E-02 | 6.000 | 5.47 | 0.53 |
| 212 | 4.46E-01 | 1.45E+01 | 4.24E-02 | 5.050 | 5.85 | -0.80 |
| 216 | 4.64E-01 | 1.39E+01 | 1.13E-01 | 3.220 | 3.88 | -0.66 |
| 217 | 4.64E-01 | 1.29E+01 | 5.84E-02 |  | 5.14 |  |
| 207 | 4.10E-01 | 1.42E+01 | 9.76E-02 | 6.000 | 5.16 | 0.84 |
| 209 | 4.09E-01 | 1.68E+01 | 8.67E-02 |  | 5.48 |  |
| 213 | 4.00E-01 | 1.60E+01 | 5.03E-02 |  | 6.47 |  |
| 219 | 4.18E-01 | 1.44E+01 | 1.17E-01 | 5.000 | 4.57 | 0.43 |
| 222 | 4.47E-01 | 1.39E+01 | 5.95E-02 |  | 5.42 |  |
| 223 | 4.51E-01 | 1.43E+01 | 5.70E-02 | 5.700 | 5.42 | 0.28 |
| 224 | 4.51E-01 | 1.36E+01 | 6.32E-02 | 5.700 | 5.26 | 0.44 |
| 225 | 4.31E-01 | 1.37E+01 | 6.68E-02 | 5.520 | 5.52 | 0.00 |
| 218 | 4.31E-01 | 1.72E+01 | 6.63E-02 | 5.400 | 5.59 | -0.19 |
| 220 | 4.11E-01 | 1.50E+01 | 1.20E-01 | 4.120 | 4.63 | -0.51 |
| 221 | 4.15E-01 | 1.48E+01 | 1.30E-01 | 3.620 | 4.33 | -0.71 |
| 226 | 4.23E-01 | 1.60E+01 | 1.17E-01 | 3.960 | 4.52 | -0.56 |
| 201 | 5.00E-01 | 1.43E+01 | 1.40E-01 | 2.850 | 2.64 | 0.21 |
| 208 | 4.59E-01 | 1.52E+01 | 3.69E-02 | 6.000 | 5.77 | 0.23 |
| 215 | 3.92E-01 | 1.83E+01 | 8.50E-02 | 6.000 | 5.83 | 0.17 |
| 206* | 4.64E-01 | 1.37E+01 | 5.29E-02 | 5.400 | 5.28 | 0.12 |
| 210* | 4.76E-01 | 1.62E+01 | 4.17E-02 | 5.100 | 5.38 | -0.28 |
| 211* | 4.27E-01 | 1.72E+01 | 4.10E-02 | 6.000 | 6.25 | -0.25 |
| 204* | 5.00E-01 | 1.23E+01 | 1.11E-01 | 3.700 | 3.29 | 0.41 |
| 203# | 4.89E-01 | 1.49E+01 | 1.63E-01 | 4.110 | 2.30 | 1.81 |

**Table S80:** Descriptor, experimental and predicted pIC_50_ values and their residuals for test set 2 compounds in cell line based QSAR model against S8

| **No.** | **Mi1ERS** | **RNN** | **MaERN** | **Exp.** | **Pred.** | **Res.** |
| --- | --- | --- | --- | --- | --- | --- |
| 229 | 4.27E-03 | 4.17E-02 | 3.96E-03 |  | 3.20 |  |
| 230 | 2.54E-03 | 3.33E-02 | 0.002 |  | 3.89 |  |
| 231 | 8.75E-03 | 4.35E-02 | 0.022 | 1.6421 | 1.70 | -0.06 |
| 233 | -6.89E-03 | 4.55E-02 | 0.015 |  | 3.92 |  |
| 235 | 2.23E-02 | 3.33E-02 | 0.002 |  | 1.43 |  |
| 236 | -1.91E-04 | 2.94E-02 | 0.003 |  | 4.39 |  |
| 237 | -1.73E-03 | 2.94E-02 | 0.002 |  | 4.65 |  |
| 239 | -2.11E-03 | 2.63E-02 | 0.003 |  | 4.81 |  |
| 241 | 2.31E-02 | 3.33E-02 | 0.000 |  | 1.39 |  |
| 242 | 2.46E-02 | 1.47E-02 | 0.007 | 1.7959 | 1.80 | -0.01 |
| 247 | 1.14E-03 | 1.92E-02 | 0.006 | 4.4685 | 4.56 | -0.09 |
| 251 | -8.96E-04 | 2.27E-02 | 0.006 | 4.8861 | 4.64 | 0.24 |
| 253 | -1.00E-03 | 4.65E-02 | 0.007 |  | 3.50 |  |
| 254 | -3.13E-03 | 7.14E-02 | 0.007 | 2.3872 | 2.55 | -0.16 |
| 256 | -6.82E-04 | 2.94E-02 | 0.006 |  | 4.31 |  |
| 227 | 1.35E-02 | 3.70E-02 | 0.002 | 2.6778 | 2.33 | 0.35 |
| 245 | 3.96E-04 | 2.27E-02 | 0.006 | 3.5171 | 4.49 | -0.97 |
| 249 | -2.65E-04 | 4.44E-02 | 0.007 | 4.2291 | 3.48 | 0.75 |
| 228 | 7.77E-04 | 5.56E-02 | 0.001 | 2.6778 | 3.11 | -0.43 |
| 255 | -6.14E-05 | 2.44E-02 | 0.006 | 4.699 | 4.46 | 0.24 |
| 248 | -9.85E-03 | 4.35E-02 | 0.021 | 4.4202 | 4.08 | 0.34 |
| 232 | 2.38E-04 | 4.35E-02 | 0.036 | 1.8665 | 2.10 | -0.23 |
| 234 | -1.03E-03 | 2.38E-02 | 0.001 | 4.5528 | 4.84 | -0.29 |
| 252 | 6.31E-04 | 2.00E-02 | 5.88E-03 | 4.7696 | 4.61 | 0.16 |
| 243 | -7.91E-04 | 2.13E-02 | 6.30E-03 | 4.9586 | 4.71 | 0.25 |
| 246 | 1.14E-03 | 1.92E-02 | 6.35E-03 | 4.4815 | 4.56 | -0.08 |
| 238* | 2.25E-02 | 2.94E-02 | 1.11E-02 | 1.153 | 1.15 | 0.01 |
| 240* | 3.42E-03 | 2.63E-02 | 9.65E-03 | 3.7696 | 3.77 | 0.00 |
| 244* | 1.04E-03 | 2.27E-02 | 6.34E-03 | 4.5229 | 4.41 | 0.12 |
| 250* | -1.16E-04 | 2.13E-02 | 6.47E-03 | 4.6383 | 4.61 | 0.03 |

**Table S81:** Descriptor, experimental and predicted pIC_50_ values and their residuals for test set 2 compounds in cell line based QSAR model against S9

| **No.** | **ABC** | **H-HD-2/T** | **HE** | **Exp.** | **Pred.** | **Res.** |
| --- | --- | --- | --- | --- | --- | --- |
| 261 | 1.00E+00 | 1.77E-02 | -5.25E+00 | 3.0269 | 2.21 | 0.81 |
| 268 | 9.95E-01 | 6.84E-03 | -6.20E+00 | 3.6383 | 3.61 | 0.03 |
| 269 | 9.75E-01 | 7.25E-03 | -5.47E+00 |  | 2.66 |  |
| 273 | 1.03E+00 | 2.17E-02 | -5.35E+00 |  | 2.64 |  |
| 275 | 9.81E-01 | 1.56E-02 | -5.48E+00 |  | 2.08 |  |
| 276 | 9.80E-01 | 2.14E-02 | -5.66E+00 |  | 1.65 |  |
| 277 | 1.02E+00 | 1.12E-02 | -5.38E+00 |  | 3.34 |  |
| 276 | 9.76E-01 | 1.11E-02 | -5.69E+00 |  | 2.48 |  |
| 281 | 9.74E-01 | 7.67E-03 | -5.69E+00 |  | 2.73 |  |
| 288 | 9.99E-01 | 6.51E-03 | -6.17E+00 | 3.7696 | 3.71 | 0.06 |
| 289 | 1.05E+00 | 1.56E-02 | -5.34E+00 | 3.9202 | 3.65 | 0.27 |
| 290 | 1.00E+00 | 1.32E-02 | -5.98E+00 |  | 3.04 |  |
| 291 | 1.00E+00 | 1.22E-02 | -5.94E+00 |  | 3.10 |  |
| 292 | 1.00E+00 | 1.54E-02 | -5.87E+00 |  | 2.78 |  |
| 293 | 1.00E+00 | 1.46E-02 | -6.03E+00 |  | 2.95 |  |
| 294 | 1.03E+00 | 1.35E-02 | -5.81E+00 |  | 3.63 |  |
| 295 | 1.03E+00 | 1.49E-02 | -5.19E+00 |  | 3.15 |  |
| 262 | 1.00E+00 | 1.21E-02 | -6.19E+00 | 3.5528 | 3.26 | 0.30 |
| 278 | 9.96E-01 | 1.18E-02 | -6.06E+00 | 2.284 | 3.11 | -0.83 |
| 285 | 9.76E-01 | 1.26E-02 | -5.31E+00 | 2.0223 | 2.12 | -0.10 |
| 267 | 9.96E-01 | 7.32E-03 | -5.24E+00 | 2.6253 | 3.02 | -0.40 |
| 286 | 9.76E-01 | 1.28E-02 | -5.39E+00 | 2.3947 | 2.15 | 0.24 |
| 274 | 1.00E+00 | 1.89E-02 | -6.24E+00 | 2.2518 | 2.69 | -0.44 |
| 270 | 9.96E-01 | 6.40E-03 | -5.92E+00 | 3.699 | 3.50 | 0.20 |
| 263 | 1.00E+00 | 1.15E-02 | -6.10E+00 | 3.6778 | 3.26 | 0.42 |
| 284 | 9.95E-01 | 1.21E-02 | -5.57E+00 | 2.5287 | 2.77 | -0.25 |
| 271 | 9.93E-01 | 6.38E-03 | -6.10E+00 | 3.6383 | 3.54 | 0.10 |
| 272 | 1.03E+00 | 1.60E-02 | -5.18E+00 | 2.4486 | 3.04 | -0.59 |
| 282 | 9.95E-01 | 6.53E-03 | -6.19E+00 | 3.6383 | 3.63 | 0.01 |
| 265 | 1.02E+00 | 1.21E-02 | -6.09E+00 | 3.8508 | 3.68 | 0.17 |
| 283* | 9.76E-01 | 1.09E-02 | -5.50E+00 | 2.3242 | 2.38 | -0.06 |
| 280* | 9.75E-01 | 1.25E-02 | -6.22E+00 | 2.4535 | 2.64 | -0.19 |
| 264* | 1.02E+00 | 1.24E-02 | -6.21E+00 | 4.1487 | 3.72 | 0.43 |
| 266* | 1.01E+00 | 1.36E-02 | -6.23E+00 | 3.9208 | 3.39 | 0.53 |
| 287# | 9.78E-01 | 7.45E-03 | -5.67E+00 | 1.983 | 2.83 | -0.85 |

**Table S82:** Descriptor, experimental and predicted pIC_50_ values and their residuals for test set 2 compounds in cell line based QSAR model against S10

| **No.** | **MaBOC** | **TPCCMD** | **MiERO** | **Exp.** | **Pred.** | **Res.** |
| --- | --- | --- | --- | --- | --- | --- |
| 296 | 1.77E+00 | 1.03E+01 | 4.09E-05 | 5 | 5.09 | -0.09 |
| 297 | 1.730 | 9.57E+00 | 7.66E-07 |  | 4.67 |  |
| 298 | 1.730 | 4.12E+00 | 8.54E-07 | 3.8097 | 3.75 | 0.06 |
| 299 | 1.720 | 3.02E+00 | 1.01E-04 |  | 3.24 |  |
| 303 | 1.630 | 6.63E+00 | 1.98E-04 |  | 2.74 |  |
| 316 | 1.620 | 3.56E+00 | 2.17E-04 | 1.2899 | 2.08 | -0.79 |
| 319 | 1.620 | 4.57E+00 | 1.44E-05 |  | 2.73 |  |
| 326 | 1.630 | 2.51E+00 | 1.96E-04 | 2.58 | 2.05 | 0.53 |
| 328 | 1.620 | 4.59E+00 | 1.97E-04 | 2.2048 | 2.30 | -0.10 |
| 332 | 1.630 | 2.75E+00 | 1.55E-04 | 2.5607 | 2.19 | 0.37 |
| 334 | 1.620 | 6.33E+00 | 1.39E-05 |  | 3.03 |  |
| 335 | 1.630 | 7.04E+00 | 1.43E-04 | 3.3188 | 2.94 | 0.38 |
| 336 | 1.630 | 4.63E+00 | 1.81E-04 |  | 2.45 |  |
| 337 | 1.630 | 2.00E+00 | 1.81E-04 | 1.6308 | 2.00 | -0.37 |
| 340 | 1.630 | 3.54E+00 | 1.85E-04 |  | 2.25 |  |
| 302 | 1.62E+00 | 7.18E+00 | 1.79E-04 | 2.6596 | 2.78 | -0.12 |
| 305 | 1.62E+00 | 7.25E+00 | 1.45E-04 | 3.0315 | 2.87 | 0.16 |
| 307 | 1.62E+00 | 6.80E+00 | 5.31E-05 | 2.9914 | 3.01 | -0.02 |
| 312 | 1.62E+00 | 4.05E+00 | 1.95E-04 | 2.5406 | 2.22 | 0.32 |
| 315 | 1.62E+00 | 7.53E+00 | 1.92E-04 | 2.7399 | 2.81 | -0.07 |
| 333 | 1.63E+00 | 3.38E+00 | 1.36E-05 | 2.7799 | 2.63 | 0.15 |
| 341 | 1.63E+00 | 4.36E+00 | 1.04E-05 | 2.4001 | 2.80 | -0.40 |
| 306* | 1.62E+00 | 5.54E+00 | 2.09E-04 | 2.6904 | 2.44 | 0.26 |
| 311* | 1.62E+00 | 6.94E+00 | 4.92E-06 | 2.9914 | 3.15 | -0.16 |
| 313* | 1.62E+00 | 6.05E+00 | 3.53E-06 | 3.1192 | 3.01 | 0.11 |
| 317* | 1.63E+00 | 5.08E+00 | 9.91E-05 | 2.7399 | 2.71 | 0.03 |
| 329* | 1.62E+00 | 1.47E+00 | 4.67E-05 | 1.9136 | 2.13 | -0.22 |
| 320* | 1.60E+00 | 8.28E+00 | 2.07E-04 | 2.5901 | 2.71 | -0.12 |

**Please note: In the entire experimental and predicted activity tables (Table S12-S46 and S48-S82) symbol “*” indicated the molecules of test set and “#” indicated the outliers.**

**Table S83a:** Cell line with type of cancer in parenthesis, scaffolds involved, regression summary (regression equation, correlation coefficient R^2^, cross validation coefficient R_cv_^2^, average residual AE and number of outliers O) and number of compounds (training set TR, test set TS and predicted set PD) in various cell lines based QSAR models for the second test set.

| **No** | **Cell line**  **(Type)** | **Scf** | **Regression equation** | **R^2^** | **R_cv_^2^** | **AE** | **O** | **# Comp.** | | |
| --- | --- | --- | --- | --- | --- | --- | --- | --- | --- | --- |
|  |  |  |  |  |  |  |  | TR | TS | PD |
| M1 | A375  (melanoma) | **S6**, **S8** | =20.951* MiVH -5.68817* ZXS /ZXR -9570.43* MiNRC -11.5072 | 0.82 | 0.76 | 0.40 | 1 | 42 | 8 | 19 |
| M2 | B16-F1  (melanoma) | **S6**, **S8** | =-88.9688* MaPCH -4.6946* ZXS /ZXR -86.4736* Ma1ERN +10.0637 | 0.79 | 0.74 | 0.37 | 1 | 40 | 10 | 19 |
| M4 | KB  (nasopharyngeal) | **S4** | =856.577* HC-2/Tz +9104.4* Mi1ERN +48.6093* MiNACH -3.8961 | 0.85 | 0.65 | 0.22 | 0 | 17 | 4 | 0 |
| M5 | WM-164  (melanoma) | **S6** | =0.169805* PS-3A_Z_ -0.976014* KHI_3_-1248.43* MiNRN +7.7253 | 0.82 | 0.79 | 0.16 | 1 | 25 | 5 | 7 |
| M6 | PC-3  (prostate) | **S6**, **S8** | =-2.56168* ZXS/ZXR -95.0094* MaPCH -2.21046* FS-2P_z_ +8.76486 | 0.80 | 0.78 | 0.30 | 0 | 31 | 10 | 23 |
| M8 | UACC-62  (melanoma) | **S10** | =-253.987* MaPCN +9.98818* MaPC +5.87792* MaVO -31.9965 | 0.77 | 0.70 | 0.28 | 1 | 21 | 5 | 6 |
| M9 | SF-539  (CNS) | **S10** | =0.000258544* GI_AP_ +0.107011* TPCCMD +15.0021* MaBOO -24.0412 | 0.80 | 0.75 | 0.27 | 0 | 21 | 6 | 3 |
| M10 | LNCaP  (prostate) | **S6**, **S7**, **S8** | =-0.0407709* ZXS +0.433344* SIC_0_-26.9721* RNN +0.626274 | 0.74 | 0.70 | 0.46 | 1 | 59 | 14 | 24 |
| M11 | PPC-1  (prostate) | **S6**, **S8** | =0.00188434* PS-1_Z_ -18.8529* RPCG_Z_ -11.6095* MaNACH +8.60453 | 0.78 | 0.72 | 0.28 | 1 | 39 | 10 | 20 |
| M12 | HCT-116  (colon) | **S10** | =-8.17055* ZXS/ZXR +0.143642* TPCCMD -0.0472899* RNCS_Q_ +7.60682 | 0.83 | 0.62 | 0.19 | 1 | 28 | 5 | 5 |
| M15 | MB-231  (breast) | **S2**, **S9** | =-0.030101* YZS +5.28796* FBCS_q_ +1.16494* MaPBO +1.35277 | 0.70 | 0.63 | 0.33 | 1 | 38 | 10 | 21 |
| M17 | A549  (lung) | **S1**, **S4** | =-0.469808* RPCS_Z_ -3.55041* MiVO +2.53191* MiBOO +9.2862 | 0.68 | 0.52 | 0.28 | 1 | 38 | 9 | 12 |
| M19 | HOP-62  (lung) | **S10** | =-13.0148* ZXS/ZXR -4.0511* RPCS_Q_ -0.742786* NF +12.6857 | 0.71 | 0.66 | 0.39 | 0 | 23 | 5 | 2 |
|  |  |  |  |  |  |  |  |  |  |  |
| M20 | KBvin  (nasopharyngeal) | **S4** | =-4.64711* HC-1/T -8.38899* PP/SD -0.807779* MaNACC +3.7918 | 0.99 | 0.99 | 0.02 | 0 | 17 | 4 | 0 |
| M21 | MCF-7  (breast) | **S1, S10** | =-5.05669* ZXS/ZXR -61.7216* MiNRO -96.7425* Mi1ERC +5.44236 | 0.71 | 0.67 | 0.31 | 1 | 39 | 9 | 36 |
| M23 | SN12C  (renal) | **S10** | =-0.204007* NN -6.64019* XYS/ XYR -3.1063* MiVC +18.8186 | 0.52 | 0.46 | 0.24 | 1 | 19 | 6 | 5 |
| M26 | OVCR-3  (ovarian) | **S1, S10** | =-0.00508733* MSA -0.232483* THCMD +2.79319* MaVO -1.28126 | 0.52 | 0.32 | 0.25 | 2 | 22 | 4 | 18 |
| M29 | DU 145  (prostate) | **S4**, **S6**, **S8**, **S9**, **S10** | =15.7416* RNO +0.0123841* HS-1_Z_ -19.3274* H-HC-2/ST +1.92184 | 0.52 | 0.50 | 0.45 | 1 | 99 | 18 | 36 |
| M3 | HeLa  (cervical) | **S2**, **S5** | =0.304206* NN +0.0023195* W-1wP +0.542243* MiVC -1.11444 | 0.82 | 0.78 | 0.13 | 1 | 46 | 14 | 11 |
| M7 | U937  (lymphoma) | **S3** | =-22.9736* MiBOH +9.01894* MiVN -53.0154* H-HD-2/T -8.01112 | 0.92 | 0.87 | 0.14 | 0 | 15 | 4 | 8 |
| M13 | Hs-638  (glioblastoma) | **S1** | =9.81424* MaVC -1555.83* A1ERC -4.39169* MiVO -28.0739 | 0.76 | 0.56 | 0.08 | 1 | 17 | 5 | 16 |
| M14 | HCT-15  (colon) | **S1** | 2.44684* AVN -1.81367* MiNACN +9.9683* MaVC -46.6449 | 0.83 | 0.73 | 0.09 | 0 | 20 | 5 | 14 |
| M16 | HL-60  (blood) | **S5** | =-7.00232* RNH -3.5503* RNO +4.05237* MaVO -3.2871 | 0.64 | 0.59 | 0.17 | 0 | 29 | 8 | 0 |
| M18 | MB468  (breast) | **S2** | =-0.198363* RPCS_Z_ +0.0153976* RNCS_Z_ -3.71988* MiBOH +4.96641 | 0.65 | 0.53 | 0.16 | 0 | 20 | 5 | 10 |
| M22 | LoVo  (colon) | **S1** | =113.504* MaERC +2.40404* MaBOO -75.7008* MaVH +69.9749 | 0.58 | 0.53 | 0.17 | 1 | 26 | 4 | 8 |
| M24 | K562  (blood) | **S2** | =0.0310103* H-HC-1_Q_ -0.250668* RPCS_Z_ +8496.67* MiERC +1.53502 | 0.48 | 0.35 | 0.18 | 1 | 20 | 4 | 10 |
| M25 | U373-MG  (glioblastoma) | **S1** | =-26.5118* ANRN -0.402577* H-1E -0.121583* MaBON +0.707112 | 0.45 | 0.32 | 0.16 | 0 | 23 | 6 | 10 |
| M27 | Fibroblast  (fibroblast) | **S6** | =19.2657* MiVN +0.741562* L1E -0.287682* THCMD -55.9964 | 0.77 | 0.70 | 0.14 | 0 | 18 | 7 | 7 |
| M28 | RH7777  (prostate) | **S6** | =-36.2559* MI-A -0.797723* MaBOC -108.708* AERN +4.21245 | 0.44 | 0.27 | 0.14 | 0 | 23 | 6 | 7 |

**Table S83b:** Cell line with type of cancer in parenthesis, scaffolds involved, regression summary (regression equation, correlation coefficient R^2^, cross validation coefficient R_cv_^2^, average residual AE and number of outliers O) and number of compounds (training set TR, test set TS and predicted set PD) in various scaffolds based QSAR models for the second test set.

| **No** | **Cell lines**  **(Type)** | **Regression equation** | **R^2^** | **R_CV_^2^** | **AE** | **O** | **# Comp.** | | |
| --- | --- | --- | --- | --- | --- | --- | --- | --- | --- |
|  |  |  |  |  |  |  | **TR** | **TE** | **PD** |
| S3 | U937  (lymphoma) | =-22.9736* MiBOH +9.01894* MiVN -53.0154* H-HD-2/T -8.01112 | 0.92 | 0.87 | 0.14 | 0 | 15 | 4 | 8 |
| S4 | KBvin  (nasopharyngeal) | =-4.64711* HC-1/T -8.38899* PP/SD -0.807779* MaNACC +3.7918 | 0.99 | 0.99 | 0.02 | 0 | 17 | 4 | 0 |
| S7 | LNCaP  (prostate) | =-16.9788* RNC +0.0166138* PS-3A_z_ -23.4348* HS-1/T +14.1728 | 0.86 | 0.79 | 0.39 | 1 | 17 | 4 | 5 |
| S8 | A375  (melanoma) | =-126.706* -48.08* -49.070* +5.9514  =-126.312* Mi1ERS -48.104* RNN -47.5663* MaERN +5.9295 | 0.90 | 0.80 | 0.24 | 0 | 16 | 4 | 13 |
| S9 | MB231  (breast) | =23.9923* ABC -87.8407* H-HD-2/T -0.586919* HE -23.3044 | 0.65 | 0.47 | 0.31 | 1 | 17 | 4 | 13 |
| S10 | DU-145  (prostate) | =9.68973* MaBOC +0.16818* TPCCMD -2357.76* MiERO -13.7013 | 0.84 | 0.80 | 0.23 | 0 | 15 | 6 | 7 |
| S1 | Hs-638  (glioblastoma) | =9.81424* MaVC -1555.83* A1ERC -4.39169* MiVO -28.0739 | 0.76 | 0.56 | 0.08 | 1 | 17 | 5 | 16 |
| S2 | K562  (blood) | =0.0310103* H-HC-1_Q_ -0.250668* RPCS_Z_ +8496.67* MiERC +1.53502 | 0.48 | 0.35 | 0.18 | 1 | 20 | 4 | 10 |
| S5 | HeLa  (cervical) | =0.168827* ACI_2_+ 9.60262* MiPCO +0.458874* RNAB +2.68046 | 0.56 | 0.38 | 0.07 | 1 | 27 | 9 | 0 |
| S6 | B16-F1  (melanoma) | =-0.244252* KHI_3_-8.95526* MV/X -106.734* MaPCH +11.8973 | 0.52 | 0.47 | 0.28 | 0 | 26 | 5 | 6 |

**Table S84: Comparative statistical significance (correlation coefficient R^2^, cross validation coefficient R_cv_^2^) of various type of cancer involved in the cell line based QSAR study**

| **Cancer type** | **a** | |  | **b** | |  | **c** | |  | **d** | |  | **e** | |  | **Average** | |
| --- | --- | --- | --- | --- | --- | --- | --- | --- | --- | --- | --- | --- | --- | --- | --- | --- | --- |
|  | **R^2^** | **R_CV_^2^** |  | **R^2^** | **R_CV_^2^** |  | **R^2^** | **R_CV_^2^** |  | **R^2^** | **R_CV_^2^** |  | **R^2^** | **R_CV_^2^** |  | **R^2^** | **R_CV_^2^** |
| Nasopharyngeal^1^ | 0.8 | 0.71 |  | 0.99 | 0.97 |  | - | - |  | - | - |  | - | - |  | 0.90 | 0.84 |
| Lymphoma^2^ | 0.84 | 0.75 |  | - | - |  | - | - |  | - | - |  | - | - |  | 0.84 | 0.75 |
| Cervical^3^ | 0.83 | 0.76 |  | - | - |  | - | - |  | - | - |  | - | - |  | 0.83 | 0.76 |
| Melanoma^4^ | 0.79 | 0.76 |  | 0.83 | 0.8 |  | 0.81 | 0.77 |  | 0.81 | 0.74 |  | - | - |  | 0.81 | 0.77 |
| CNS^5^ | 0.81 | 0.75 |  | - | - |  | - | - |  | - | - |  | - | - |  | 0.81 | 0.75 |
| Fibroblast^6^ | 0.79 | 0.72 |  | - | - |  | - | - |  | - | - |  | - | - |  | 0.79 | 0.72 |
| Colon^7^ | 0.86 | 0.77 |  | 0.86 | 0.77 |  | 0.6 | 0.53 |  | - | - |  | - | - |  | 0.77 | 0.69 |
| Glioblastoma^8^ | 0.83 | 0.7 |  | 0.55 | 0.46 |  | - | - |  | - | - |  | - | - |  | 0.69 | 0.58 |
| Prostate^9^ | 0.83 | 0.79 |  | 0.75 | 0.74 |  | 0.8 | 0.77 |  | 0.46 | 0.43 |  | 0.58 | 0.38 |  | 0.68 | 0.62 |
| Breast^10^ | 0.7 | 0.66 |  | 0.72 | 0.65 |  | 0.64 | 0.5 |  | - | - |  | - | - |  | 0.67 | 0.60 |
| Lung^11^ | 0.64 | 0.56 |  | 0.7 | 0.66 |  | - | - |  | - | - |  | - | - |  | 0.67 | 0.61 |
| Blood^12^ | 0.65 | 0.61 |  | 0.62 | 0.54 |  | - | - |  | - | - |  | - | - |  | 0.63 | 0.58 |
| Ovarian^13^ | 0.63 | 0.51 |  | - | - |  | - | - |  | - | - |  | - | - |  | 0.63 | 0.51 |
| Renal^14^ | 0.6 | 0.51 |  | - | - |  | - | - |  | - | - |  | - | - |  | 0.60 | 0.51 |

^1^ a=KB, b=KBvin; ^2^ a=U937; ^3^ a=HeLa; ^4^ a=HCT-116, b=HCT-1; ^5^ a=SF-539; ^6^ a=Fibroblast; ^7^ a=HCT-116, b=HCT-15 c=LoVo;

^8^ a=Hs 638, b=U373-MG; ^9^ a=PC3, b=LNCaP, c=PPC1, d=DU145, e=RH7777; ^10^ a=MB231, b=MCF-7, c=MB468; ^11^ a=A549, b=HOP-62;

^12^ a=HL-60, b=K562; ^13^ a=OVCR-3; ^14^ a=SN12C.

**Figure S1a:** Plot between experimental and predicted IC_50_ values for 11-cell line based QSAR models with name of cell lines, correlation coefficient, cross validation coefficient, average residual of training set and average residual of test set of molecules

**Figure S1b:** Plot between experimental and predicted IC_50_ values for 4 scaffold based QSAR models with name of cell lines, correlation coefficient, cross validation coefficient, average residual of training set and average residual of test set of molecules
